# Disrupted fear regulation in insomnia disorder revealed by arousal estimation from skin conductance

**DOI:** 10.1371/journal.pmen.0000720

**Published:** 2026-10-14

**Authors:** Tianyu Hwang, Saman Khazaei, Edward F. Pace-Schott, Ryan Bottary, Rose T. Faghih

**Affiliations:** 1 Department of Mathematics, Tandon School of Engineering, New York University, New York City, New York, United States of America; 2 Department of Biomedical Engineering, Tandon School of Engineering, New York University, New York City, New York, United States of America; 3 Department of Psychiatry, Massachusetts General Hospital, Harvard Medical School, Boston, Massachusetts, United States of America; Seoul National University College of Medicine, KOREA, REPUBLIC OF

## Abstract

Insomnia disorder (ID) is a common sleep-wake disorder marked by persistent dissatisfaction with sleep quality or quantity. Previous studies have shown that sleep plays a critical role in emotion regulation, particularly in fear extinction. This study aims to characterize ID using estimates of the sympathetic arousal state derived from skin conductance (SC)-based observations, and to compare two observation models: the marked point process (MPP) model and the MPP coupled with continuous (MPP+Cont) model. We utilize SC data from a fear conditioning and extinction procedure involving 24 individuals with ID and 24 good sleepers (GS). The SC measurements are deconvolved to extract autonomic nervous system (ANS) activations and the tonic component. The MPP observation model uses only the extracted ANS activations, while the MPP+Cont model incorporates both ANS activations and the tonic component. Both models are employed to estimate the underlying arousal state over time and compute a high arousal index (HAI). We compare the estimated arousal states and HAI between the ID and GS groups to assess alterations in arousal associated with ID. Our results reveal that ID may be associated with impaired fear extinction learning and recall. Notably, the distinction between ID and GS emerges only under the MPP+Cont model, indicating the critical role of the tonic component in arousal estimation. Overall, this study highlights the potential of arousal state estimation for understanding autonomic dysregulation in ID, thereby supporting interventions for fear-centered comorbidities and improving quality of life.

## Introduction

Insomnia disorder (ID) is a common sleep-wake disorder that affects approximately 10% of the population [1,2]. According to the DSM-5-TR [1] and ICSD-3-TR [2], ID is marked by persistent dissatisfaction with sleep quality or quantity, such as difficulty initiating or maintaining sleep, or early morning awakenings with an inability to return to sleep. ID is often associated with significant daytime impairments, including fatigue, cognitive dysfunction, and mood disturbances, as well as an increased risk of mental health disorders such as anxiety and depression [1–4]. Therefore, early detection and intervention of ID are crucial for preventing the development of comorbid conditions and improving overall quality of life. Characterizing physiological and behavioral features of ID during wakefulness may aid differential diagnosis, as a range of other conditions can manifest with sleep disturbance.

ID has been widely conceptualized as a disorder of chronic hyperarousal, which contributes to emotional and autonomic dysregulation [5–11]. Consistent with this view, previous studies have shown that sleep plays a critical role in emotion regulation [12]. In particular, growing evidence highlights that sleep helps consolidate fear extinction—a form of emotion regulation that involves encoding a new inhibitory memory that indicates a previously learned threat no longer signals danger [6,13–30]. For example, Seo et al. [28] demonstrated that individuals with ID exhibit delayed engagement of neuronal structures that support extinction learning compared to good sleepers (GS), while Bottary et al. [29] reported a negative association between fear extinction memory and rapid eye movement (REM) sleep in individuals with ID. These findings suggest that disrupted fear regulation may be a potential risk factor for ID. These studies of fear regulation employ the fear conditioning and extinction procedure [31], which involves learning to associate a conditioned stimulus (CS), such as a colored image, with a shock, followed by extinction through repeated presentations of the CS without the shock.

Skin conductance (SC), or electrodermal activity (EDA), has been well-established as a biomarker of emotional arousal and affective regulation [32], and is widely utilized in fear conditioning and extinction procedures [14–18,20–22,24,26,27,29–31,33–36]. Prior studies examining the relationship between sleep and SC have primarily relied on simple SC response (SCR) metrics, such as peak-to-peak [14,16,18,20] or peak-to-mean differences [15,17,21,24,26,27]. More recent approaches have introduced advanced methods that decompose SC signals into phasic and tonic components [34,37–45]. Faghih et al. [34] proposed a deconvolution method for recovering underlying emotional stimulus responses to study fear regulation. Building on this, Amin et al. [45] developed BayesianEDA, a deconvolution algorithm that estimates autonomic nervous system (ANS) activations and the tonic component of SC. Compared to other deconvolution approaches, BayesianEDA achieves a more balanced estimation of activation sparsity while maintaining high computational scalability.

Rather than relying solely on SC as an indirect measure of emotional arousal, Wickramasuriya et al. [46] introduced a state-space approach to estimate the underlying sympathetic arousal state over time from physiological signals. To quantify arousal levels, they further defined the High Arousal Index (HAI)—the probability that the arousal state exceeds a specified threshold [47]. Specifically, sympathetic arousal is the activation of the sympathetic nervous system in response to emotional stimuli; the arousal state is a mathematical representation of this sympathetic arousal; and HAI is an index based on the arousal state that quantifies the arousal levels. Wickramasuriya et al. have developed several observation models to relate the arousal state to physiological signals such as SC [46–52]. Notably, their marked point process (MPP) model [50] relates the arousal state to the occurrence of ANS activations through a logistic function, and models activation amplitudes linearly. This model has demonstrated improved robustness to noisy activations compared to earlier models that either omitted amplitude information [46,47] or incorporated interpolated amplitudes and the tonic component [48]. The MPP model was later extended to include a linear relationship with the continuous tonic component [52], resulting in the MPP coupled with continuous (MPP+Cont) model. This extension further improves arousal state estimation by capturing the arousal baseline via tonic component.

In this study, we aim to characterize ID using estimates of the underlying sympathetic arousal state, rather than relying on traditional SCR metrics. We also compare two observation models: the MPP model and the MPP+Cont model, to investigate the impact of the tonic component on arousal state estimation and assess the association between the tonic component and ID. For this purpose, we utilize the SC dataset from Bottary et al. [27], in which individuals with ID and GS participated in the fear conditioning and extinction procedure [31]. The SC measurements are first deconvolved to extract ANS activations and the tonic component. These components are then used to estimate the underlying arousal state over time using the MPP and MPP+Cont models. Finally, we compare the estimated arousal states and HAI between the ID and GS groups to assess alterations in arousal dynamics associated with ID. We hypothesize that individuals with ID exhibit distinct patterns of arousal states compared to GS subjects and test this hypothesis using statistical analyses. Because disrupted sleep can potentially interfere with extinction learning, our findings may also be relevant for post-traumatic stress disorder (PTSD) and anxiety disorders, where problems with fear extinction make both recovery and treatment more challenging [6,25,30].

## Materials and methods

### Ethics statement

All study procedures were conducted in accordance with the Declaration of Helsinki and were approved by the Partners Healthcare Institutional Review Board. Participant recruitment ran from February 10, 2014 to February 4, 2017. All participants provided written informed consent.

### Skin conductance dataset

The SC dataset, originally presented by Bottary et al. [27], consists of SC recordings from 24 individuals with ID and 24 GS (ID age: 30.38±13.60 years; GS age: 30.79±13.79 years). Each group consists of 18 female and 6 male subjects. All participants were selected from an initial telephone screening. Exclusion criteria included: shift work; sleep/psychotropic medication use (or positive urine toxicology screen); evidence of sleep disorder (except insomnia in ID group); history of DSM-IV-TR Axis I psychiatric disorder [53] (except insomnia in ID group) including substance abuse disorders; history of major medical/neurologic disorders; pregnancy; and MRI contraindications.

Participants were clinically evaluated using the Pittsburgh Structured Clinical Interview for Sleep Disorders (PSCI-SD) [54,55]. Based on PSCI-SD responses, diagnoses were determined according to criteria for DSM-5 Insomnia Disorder [56] and ICSD-3 Chronic Insomnia Disorder [57]. Participants assigned to the ID group met Pittsburgh Sleep Quality Index (PSQI) ≥5 [58] and Insomnia Severity Index (ISI) ≥13 [59], whereas GS participants had PSQI < 3 and ISI < 4. Furthermore, a trained clinical psychologist administered the Structured Clinical Interview for DSM-IV for Non-Patients (SCID-I/NP) [60] to exclude participants with any current psychiatric disorders (except insomnia in ID group).

Participants underwent the fear conditioning and extinction procedure [31], which includes three phases: conditioning, extinction learning, and extinction recall. SC measurements were recorded using the MP150 system with AcqKnowledge 4.3 software (BIOPAC Systems, Inc., Goleta, CA) at a sampling rate of 200 Hz. The SC measuring itself was passive and causes no discomfort. However, stimulating electrodes were attached to the right index and middle fingers to deliver mild electrical shocks. To manage discomfort, the shock level was individualized, where participants were asked to select a level which they perceived to be “highly annoying but not painful”, from increasing intensities of shock. The procedure was conducted during fMRI scanning.

The SC data exhibiting irregular fluctuations, likely due to poor measurements, were visually inspected and excluded. The remaining SC data were then deconvolved to extract ANS activations and the tonic component using a modified BayesianEDA algorithm [61]. The sparsity parameter λ in the deconvolution algorithm is estimated empirically as described in [45]. To ensure meaningful arousal estimation, subjects with an estimated ANS activation density below 0.1% were excluded. The number of subjects retained in each phase after data exclusion is summarized in Table 1. The preprocessed SC data, downsampled to 4 Hz, along with the deconvolved ANS activations and the tonic component, are provided in S1 Data.

**Table 1:** Number of subjects retained after data exclusion in each phase.

Phase	ID	GS
Conditioning	16	17
Extinction	11	14
Recall	17	15

### Estimation of arousal state

The sympathetic arousal state is modeled as a hidden state that governs SC dynamics and ANS activations. Primarily, we aim to decode this hidden state over time using the MPP+Cont model [52], based on observations derived from SC measurements. Specifically, given observations $\{(n_{k},r_{k},s_{k}){\}}_{k=1}^{K}$, where $n_{k}\in \{0,1\}$ denotes the occurrence of an ANS activation, $r_{k}\geq 0$ denotes the corresponding amplitude, and $s_{k}\geq 0$ denotes the tonic component of SC, our objective is to estimate the underlying arousal states $\{x_{k}{\}}_{k=1}^{K}$. Note that the arousal state is expressed in arbitrary units (a.u.).

First, we assume that the arousal state follows a first-order Gaussian random walk model [52] parameterized by an unknown process noise variance $\sigma_{x}^{2}$:


$$\begin{array}{c} x_{k}=x_{k-1}+\epsilon_{x,k},\;\epsilon_{x,k}\sim 𝒩(0,\sigma_{x}^{2}) \end{array}$$

with initial state *x*_0_ = 0. Next, we assume that the occurrence of ANS activation $n_{k}$ follows a Bernoulli distribution [52] with probability $p_{k}$, and we relate the arousal state $x_{k}$ to $p_{k}$ using a logistic model [52] parameterized by unknowns $\beta_{0}$ and $\beta_{1}$:


$$\begin{array}{c} p_{k}=\frac{1}{1+e^{-(\beta_{0}+\beta_{1}x_{k})}} \end{array}$$

Moreover, we model the corresponding activation amplitude $r_{k}$ using a Gaussian linear model [52] parameterized by unknowns $\gamma_{0}$, $\gamma_{1}$, and sensor noise variance $\sigma_{r}^{2}$:


$$\begin{array}{c} r_{k}=\gamma_{0}+\gamma_{1}x_{k}+\epsilon_{r,k},\;\epsilon_{r,k}\sim 𝒩(0,\sigma_{r}^{2}),\;n_{k}=1 \end{array}$$

Similarly, we relate the arousal state $x_{k}$ to the tonic component $s_{k}$ using another Gaussian linear model [52], parameterized by unknowns $\delta_{0}$, $\delta_{1}$, and sensor noise variance $\sigma_{s}^{2}$:


$$\begin{array}{c} s_{k}=\delta_{0}+\delta_{1}x_{k}+\epsilon_{s,k},\;\epsilon_{s,k}\sim 𝒩(0,\sigma_{s}^{2}) \end{array}$$

Note that activation amplitude $r_{k}$ only applies when ANS activation occurs, i.e., $n_{k}=1$, whereas the tonic component $s_{k}$ is always observed. Therefore, the posterior of the arousal states $\{x_{k}{\}}_{k=1}^{K}$ is given by:


$$\begin{array}{cc} & p(\{x_{k}{\}}_{k=1}^{K}|\{(n_{k},r_{k},s_{k}){\}}_{k=1}^{K},\Theta )\\ & \propto \prod_{k=1}^{K}p_{k}^{n_{k}}(1-p_{k}{)}^{1-n_{k}}𝒩(r_{k}|\gamma_{0}+\gamma_{1}x_{k},\sigma_{r}^{2}{)}^{n_{k}}𝒩(s_{k}|\delta_{0}+\delta_{1}x_{k},\sigma_{s}^{2})𝒩(x_{k}|x_{k-1},\sigma_{x}^{2}) \end{array}$$

where $\Theta =\{\sigma_{x}^{2},\beta_{0},\beta_{1},\gamma_{0},\gamma_{1},\sigma_{r}^{2},\delta_{0},\delta_{1},\sigma_{s}^{2}\}$ denotes the unknown model parameters. The sensor noise variances, $\sigma_{r}^{2}$ and $\sigma_{s}^{2}$, are estimated empirically from the observations to ensure robustness against noise and artifact contamination in the SC data.

Based on this posterior, we now estimate the arousal states $\{x_{k}{\}}_{k=1}^{K}$ and model parameters Θ using the Expectation-Maximization (EM) algorithm [52]. The E- and M-steps are iterated until convergence, which is defined as the point at which the relative change in all parameters within Θ falls below 0.1%, or until a maximum of 500 iterations is reached. Based on preliminary empirical tuning, the parameters are initialized as follows: $\sigma_{x}^{2}=0.005$, $\gamma_{0}=0$, $\gamma_{1}=10$, $\sigma_{r}^{2}=0.1\cdot \text{var}(\{r_{k}{\}}_{n_{k}=1})$, $\delta_{0}=s_{1}$, $\delta_{1}=0.1$, and $\sigma_{s}^{2}=10\cdot \text{var}(\{s_{k}{\}}_{k=1}^{K})$. We fix $\beta_{1}=1$ and empirically estimate $\beta_{0}=\log\left(\frac{{\hat{p}}_{0}}{1-{\hat{p}}_{0}}\right)$, where ${\hat{p}}_{0}=\frac{1}{K}\sum_{k=1}^{K}n_{k}$ represents the empirical probability of ANS activation. To mitigate potential overfitting to the tonic component, the parameters $\delta_{0}$, $\delta_{1}$, and $\sigma_{s}^{2}$ are updated using an exponential smoothing factor of 0.1, and their updates are terminated early when $\sigma_{s}^{2}<0.1\cdot \text{var}(\{s_{k}{\}}_{k=1}^{K})$. Full mathematical details of the E-step and M-step are provided in S1 File.

Furthermore, we also consider the MPP model [50] for arousal state estimation, which excludes the tonic component $s_{k}$. This model can be viewed as a special case of the MPP+Cont model, where $\delta_{0}=\delta_{1}=0$ and $\sigma_{s}^{2}=1$ are fixed.

### Statistical analysis

The primary objective of this study was to compare arousal levels between the ID and GS groups during the fear conditioning and extinction procedures. Specifically, we compared the estimated arousal states and HAI between groups (ID vs. GS) within each phase (conditioning, extinction, and recall), as well as individual phase-to-phase changes between groups. The primary features extracted for comparison were the mean arousal state $\bar{x}$ and the mean HAI $\bar{h}$. As introduced in [47], the HAI is computed as follows:


$$\begin{array}{c} h_{k}=P(X_{k}>\text{median}(\{x_{j}{\}}_{j=1}^{K})),\;X_{k}\sim 𝒩(x_{k},\sigma_{k}^{2}), \end{array}$$

where $x_{k}$ and $\sigma_{k}^{2}$ denote the estimated arousal state and its corresponding variance. Note that the HAI $h_{k}$ represents a probability and is expressed in arbitrary units (a.u.). Statistical comparisons were performed at two levels using two-sample Wilcoxon rank-sum tests:

- **Between-group comparisons for each phase:** Assess if the primary features differed significantly between the ID and GS groups within the same phase.
- **Between-group comparisons of phase-to-phase changes:** Assess if the changes in primary features across phases differ significantly between the ID and GS groups.

To control for overestimating the evidence for group differences from multiple testing, the reported *p*-values for these primary analyses were adjusted using the Bonferroni correction across the two primary features within each phase. To evaluate whether the sample size limited the statistical power to detect meaningful group differences, we report non-parametric effect sizes (*r*) for all comparisons. Effect sizes were calculated as $r=Z/\sqrt{N}$, where *Z* is the standardized test statistic and *N* is the total sample size, to indicate medium effect sizes (|r|≥0.3) [62]. All statistical analyses were performed using SciPy 1.18.0 [63].

As an exploratory secondary analysis, we also compared the standard deviation of the arousal state ${\hat{\sigma }}_{x}$ and the standard deviation of the HAI ${\hat{\sigma }}_{h}$ between groups and across phases. These results are provided in S2 File.

## Results

### Estimated arousal state from SC

Fig 1 (left) shows the MPP+Cont model estimates for a representative ID subject during the extinction phase. The estimated arousal states ($x_{est}$) capture both the ANS activations ($r_{obs}$) and the tonic component ($s_{obs}$). However, the estimated amplitudes ($r_{est}$) of the discrete ANS activations exhibit notably large variance. For comparison, the MPP model estimates for the same subject are shown in Fig 1 (right). Unlike the MPP+Cont model, which incorporates the tonic component, the MPP model estimates display substantially larger variability in the arousal states ($x_{est}$). Estimation results for all subjects are provided in S3 File.

**Fig 1:**
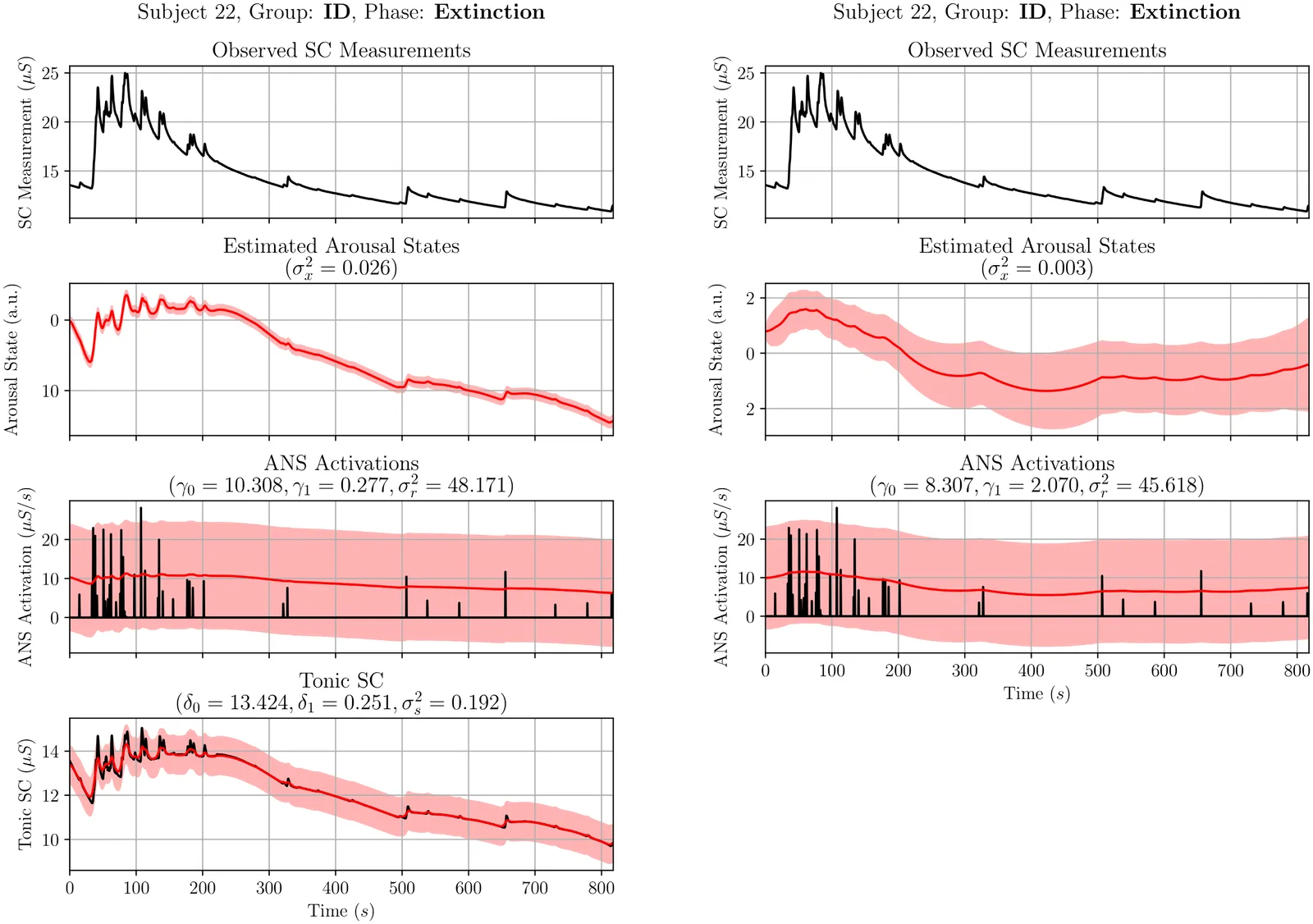
Estimation results under both models for a representative ID subject during the extinction phase. Shown for the MPP+Cont model (left) and the MPP model (right). The first panels show the observed SC measurements, *y* (black). The second panels show the estimated arousal states, $x_{est}$ (red). The third panels show the ANS activations, $r_{obs}$ (black) and the estimated amplitudes, $r_{est}$ (red). The fourth panel shows the tonic component, $s_{obs}$ (black) and the estimated tonic component, $s_{est}$ (red). Estimates are shown with 95% confidence intervals (CI) (red shaded area). Estimated parameters are reported in the title of each panel. Note that the MPP+Cont model provides higher confidence estimates by incorporating the tonic component.

Fig 2 further compares the estimated arousal states for a representative ID subject and a representative GS subject across the conditioning, extinction, and recall phases. Overall, the estimated arousal states ($x_{est}$) appear more spiky during the conditioning phase. Additionally, the ID participant shows elevated arousal states ($x_{est}$) at the beginning of the extinction phase.

**Fig 2:**
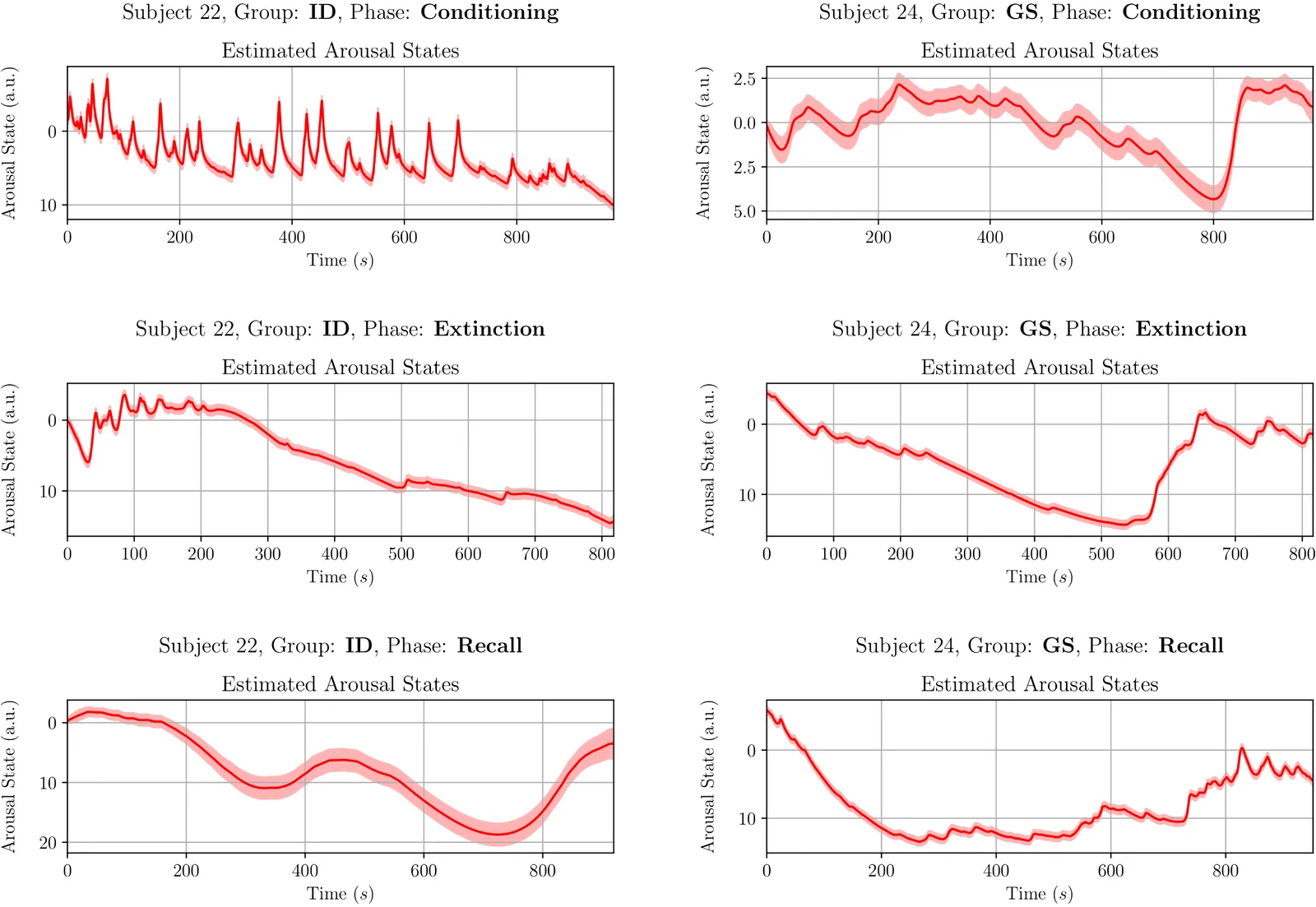
Estimated arousal states under the MPP+Cont model for representative ID and GS subjects. Shown for ID (left) and GS (right) subject during the conditioning (top), extinction (middle), and recall (bottom) phases. The panels show the estimated arousal states, $x_{est}$ (red). Estimates are shown with 95% confidence intervals (CI) (red shaded area).

### Between-group comparisons for each phase

Tables 2 and 3 summarize the extracted primary features ($\bar{x}$, $\bar{h}$) under the MPP+Cont and MPP models, respectively. For each model, the median, lower quartile, and upper quartile of these features are reported for the ID and GS groups across the conditioning, extinction, and recall phases. The *p*-values and effect sizes *r* from Wilcoxon rank-sum tests comparing feature distributions between the ID and GS groups are also provided. Box plots of the corresponding features under the MPP+Cont and MPP models are shown in Figs 3 and 4, respectively.

**Table 2:** Features under the MPP+Cont model for ID and GS groups during each phase. Median, lower quartile, and upper quartile values of the features are reported. *p*-values and effect sizes *r* from Wilcoxon rank-sum tests comparing features between the ID and GS groups are also provided. Significant differences (*p* < 0.05) and medium effect sizes (|r|≥0.3) are highlighted in bold.

Conditioning				
Feature	ID (*n* = 16)	GS (*n* = 17)	*p*-value	Effect Size (*r*)
$\bar{x}$ (a.u.)	-0.329 [-4.513, 0.326]	-3.257 [-5.704, -0.740]	0.4148	0.2195
$\bar{h}$ (a.u.)	0.498 [0.489, 0.504]	0.504 [0.500, 0.507]	0.0734	**-0.3637**
**Extinction**				
**Feature**	**ID (*n* = 11)**	**GS (*n* = 14)**	***p*-value**	**Effect Size (*r*)**
$\bar{x}$ (a.u.)	-4.034 [-8.190, -3.350]	-4.800 [-9.126, -2.045]	1.0000	-0.0985
$\bar{h}$ (a.u.)	0.499 [0.497, 0.513]	0.500 [0.500, 0.504]	1.0000	-0.0219
**Recall**				
**Feature**	**ID (*n* = 17)**	**GS (*n* = 15)**	***p*-value**	**Effect Size (*r*)**
$\bar{x}$ (a.u.)	-3.918 [-7.581, -2.128]	-7.287 [-9.126, -5.351]	0.0722	**0.3705**
$\bar{h}$ (a.u.)	0.500 [0.493, 0.501]	0.501 [0.496, 0.507]	0.7497	-0.1569

**Table 3:** Features under the MPP model for ID and GS groups during each phase. Median, lower quartile, and upper quartile values of the features are reported. *p*-values and effect sizes *r* from Wilcoxon rank-sum tests comparing features between the ID and GS groups are also provided. Significant differences (*p* < 0.05) and medium effect sizes (|r|≥0.3) are highlighted in bold.

Conditioning				
Feature	ID (*n* = 16)	GS (*n* = 17)	*p*-value	Effect Size (*r*)
$\bar{x}$ (a.u.)	-0.121 [-0.324, -0.043]	-0.082 [-0.318, -0.032]	1.0000	-0.0878
$\bar{h}$ (a.u.)	0.530 [0.508, 0.558]	0.544 [0.523, 0.560]	0.7746	-0.1505
**Extinction**				
**Feature**	**ID (*n* = 11)**	**GS (*n* = 14)**	***p*-value**	**Effect Size (*r*)**
$\bar{x}$ (a.u.)	-0.225 [-0.557, -0.071]	-0.170 [-0.258, -0.116]	1.0000	-0.0876
$\bar{h}$ (a.u.)	0.575 [0.524, 0.594]	0.564 [0.521, 0.587]	1.0000	0.0985
**Recall**				
**Feature**	**ID (*n* = 17)**	**GS (*n* = 15)**	***p*-value**	**Effect Size (*r*)**
$\bar{x}$ (a.u.)	-0.221 [-0.542, -0.092]	-0.159 [-0.379, -0.063]	0.5306	-0.1969
$\bar{h}$ (a.u.)	0.528 [0.494, 0.563]	0.535 [0.505, 0.546]	1.0000	0.0367

**Fig 3:**
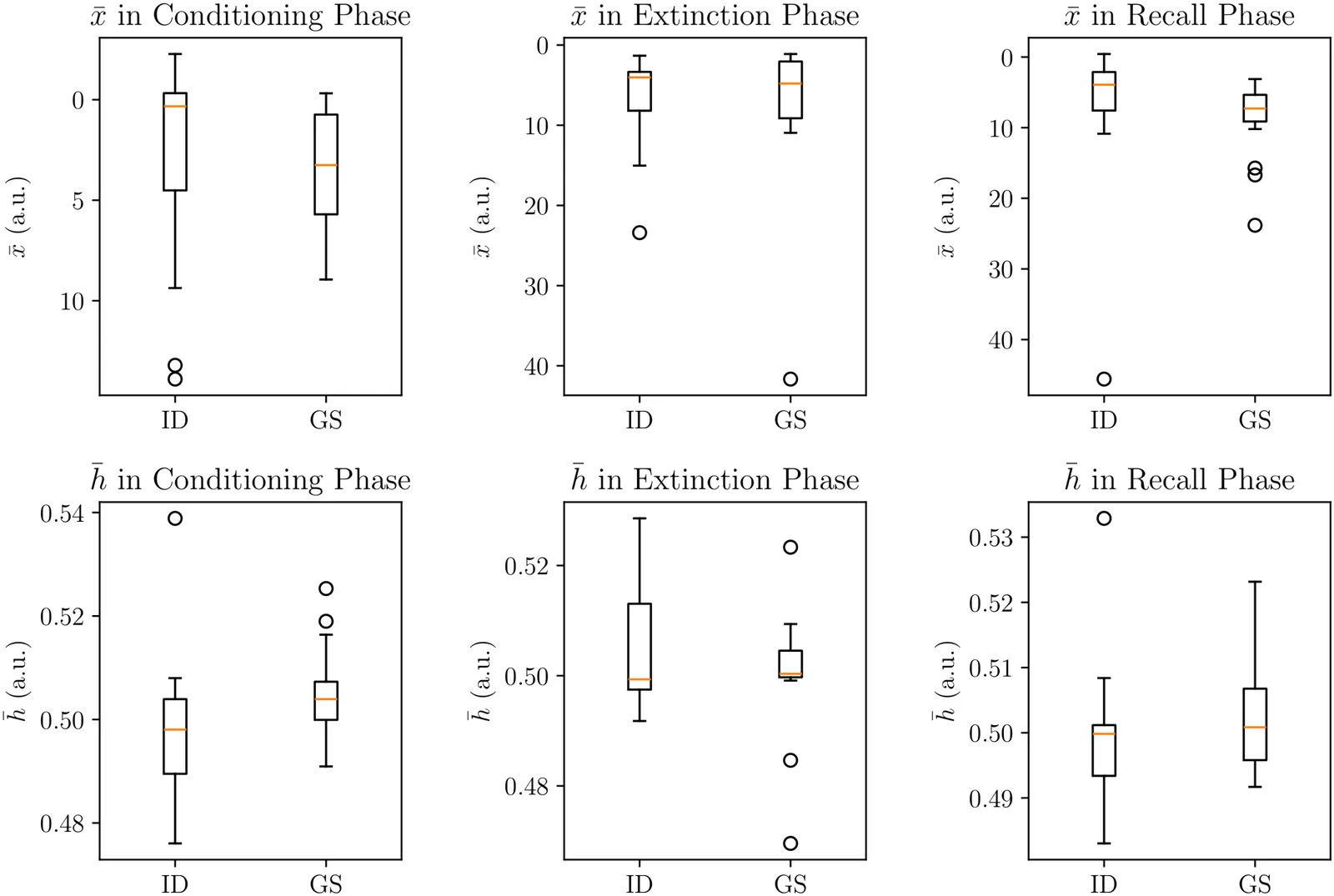
Box plots of features under the MPP+Cont model. Shown for ID and GS groups during the conditioning (left), extinction (middle), and recall (right) phases.

**Fig 4:**
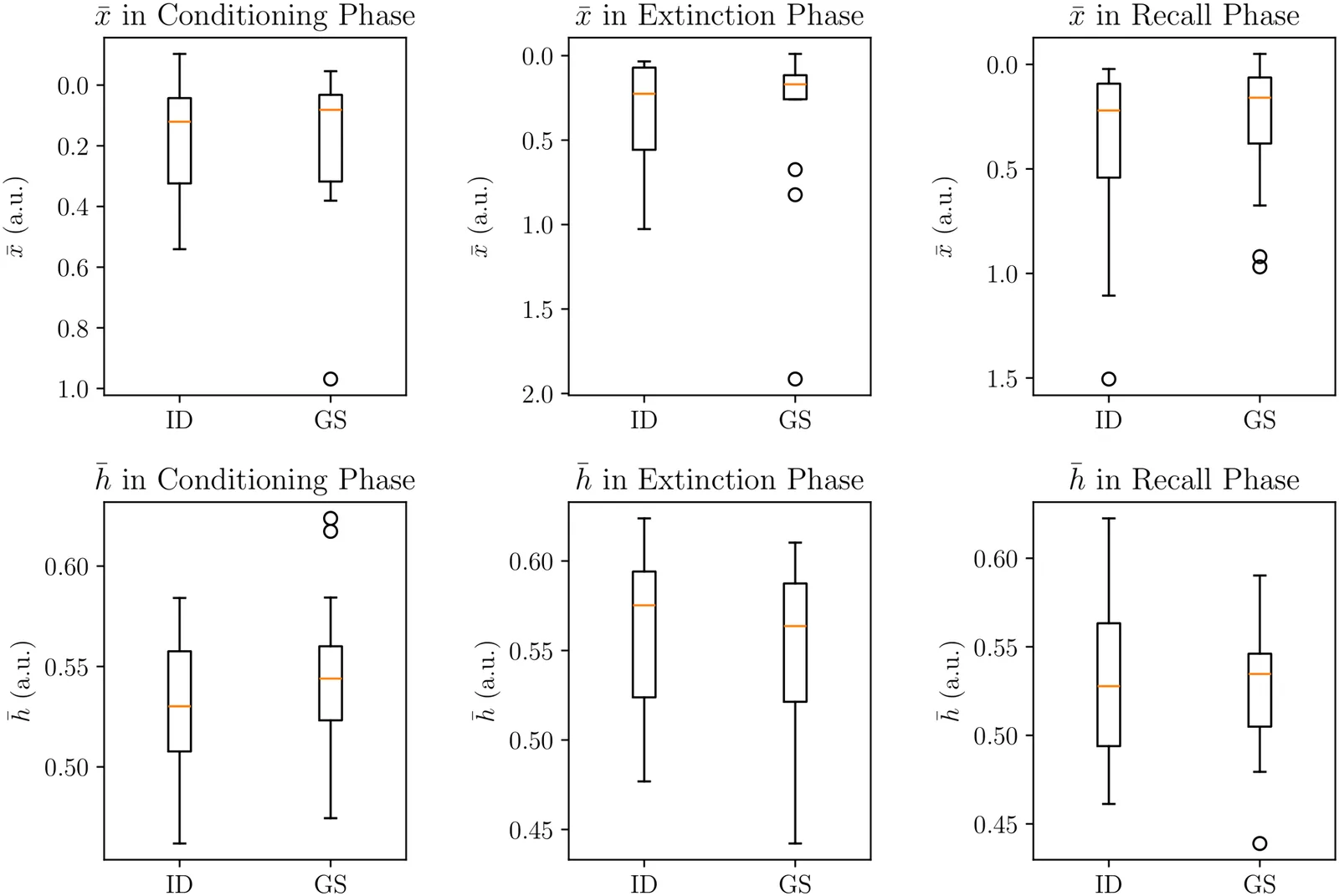
Box plots of features under the MPP model. Shown for ID and GS groups during the conditioning (left), extinction (middle), and recall (right) phases.

In Table 2, under the MPP+Cont model, we observe that the mean HAI ($\bar{h}$) is lower with a medium effect size (*p* = 0.0734, r=−0.3637) in the ID group compared to the GS group during the conditioning phase. Conversely, the mean arousal state ($\bar{x}$) is higher with a medium effect size (*p* = 0.0722, *r* = 0.3705) in the ID group compared to the GS group during the recall phase.

### Between-group comparisons of phase-to-phase changes

Tables 4 and 5 summarize individual phase-to-phase changes in primary features ($\Delta \bar{x}$, $\Delta \bar{h}$) under the MPP+Cont and MPP models, respectively. Only subjects with retained data in both phases after exclusion are included in the analysis. For each model, the median, lower quartile, and upper quartile of feature changes are reported for the ID and GS groups across the conditioning-to-extinction, conditioning-to-recall, and extinction-to-recall phase transitions. *p*-values and effect sizes *r* from Wilcoxon rank-sum tests are also provided.

**Table 4:** Changes in features under the MPP+Cont model for ID and GS groups between each phase. Median, lower quartile, and upper quartile values of the feature changes are reported. *p*-values and effect sizes *r* from Wilcoxon rank-sum tests comparing changes between the ID and GS groups are also provided. Significant differences (*p* < 0.05) and medium effect sizes (|r|≥0.3) are highlighted in bold.

Conditioning to Extinction				
ΔFeature	ID (*n* = 10)	GS (*n* = 14)	*p*-value	Effect Size (*r*)
$\Delta \bar{x}$ (a.u.)	-2.893 [-7.021, 0.136]	-2.334 [-5.082, 0.377]	1.0000	-0.0478
$\Delta \bar{h}$ (a.u.)	0.006 [0.001, 0.025]	-0.002 [-0.013, 0.003]	**0.0236**	**0.5139**
**Conditioning to Recall**				
**ΔFeature**	**ID (*n* = 14)**	**GS (*n* = 14)**	***p*-value**	**Effect Size (*r*)**
$\Delta \bar{x}$ (a.u.)	-2.020 [-4.392, 0.121]	-3.632 [-6.308, -1.868]	0.2156	**0.3039**
$\Delta \bar{h}$ (a.u.)	0.002 [-0.004, 0.011]	-0.004 [-0.011, 0.001]	0.3361	0.2605
**Extinction to Recall**				
**ΔFeature**	**ID (*n* = 10)**	**GS (*n* = 12)**	***p*-value**	**Effect Size (*r*)**
$\Delta \bar{x}$ (a.u.)	-0.001 [-1.721, 3.647]	0.336 [-3.076, 1.292]	1.0000	0.0843
$\Delta \bar{h}$ (a.u.)	-0.000 [-0.007, 0.003]	0.004 [-0.007, 0.010]	0.7119	-0.1968

**Table 5:** Changes in features under the MPP model for ID and GS groups between each phase. Median, lower quartile, and upper quartile values of the feature changes are reported. *p*-values and effect sizes *r* from Wilcoxon rank-sum tests comparing changes between the ID and GS groups are also provided. Significant differences (*p* < 0.05) and medium effect sizes (|r|≥0.3) are highlighted in bold.

Conditioning to Extinction				
ΔFeature	ID (*n* = 10)	GS (*n* = 14)	*p*-value	Effect Size (*r*)
$\Delta \bar{x}$ (a.u.)	-0.185 [-0.306, 0.007]	-0.102 [-0.150, -0.035]	1.0000	-0.1315
$\Delta \bar{h}$ (a.u.)	0.040 [0.010, 0.061]	0.018 [-0.034, 0.078]	1.0000	0.0717
Conditioning to Recall				
$\begin{array}{c} \Delta \end{array}$Feature	ID (*n* = 14)	GS (*n* = 14)	*p*-value	Effect Size (*r*)
$\Delta \bar{x}$ (a.u.)	-0.090 [-0.304, 0.029]	-0.081 [-0.170, 0.037]	0.9245	-0.1389
$\Delta \bar{h}$ (a.u.)	-0.000 [-0.017, 0.036]	-0.047 [-0.066, 0.026]	0.4645	0.2258
Extinction to Recall				
$\begin{array}{c} \Delta \end{array}$Feature	ID (*n* = 10)	GS (*n* = 12)	*p*-value	Effect Size (*r*)
$\Delta \bar{x}$ (a.u.)	0.061 [-0.117, 0.476]	0.088 [-0.053, 0.301]	1.0000	-0.0843
$\Delta \bar{h}$ (a.u.)	-0.059 [-0.082, 0.024]	-0.011 [-0.057, 0.001]	0.8576	-0.1687

In Table 4, under the MPP+Cont model, the increase in mean HAI ($\Delta \bar{h}$) in the ID group is significantly larger (*p* = 0.0236, *r* = 0.5139) than that of the GS group during the conditioning-to-extinction phase. The decrease in mean arousal state ($\Delta \bar{x}$) in the ID group is also smaller with a medium effect size (*p* = 0.2156, *r* = 0.3039) than that of the GS group during the conditioning-to-recall phase.

## Discussion

### Estimated arousal state from SC

Due to the discrete nature of ANS activations, the amplitudes used to fit the arousal states are inherently sparse. This sparsity contributes to a higher estimated sensor noise variance for the estimated amplitudes ($r_{est}$), as shown in Fig 1. Since the MPP model does not incorporate the tonic component, it does not to account for baseline fluctuations, resulting in greater variability in the estimated arousal states ($x_{est}$) compared to the MPP+Cont model. In contrast, the MPP+Cont model captures both discrete and continuous components, allowing it to detect more subtle changes in arousal states ($x_{est}$) and thereby demonstrating improved sensitivity, consistent with the findings in [52].

Additionally, as shown in Fig 2, the estimated arousal states ($x_{est}$) appear more spiky during the conditioning phase, which aligns with the fact that the CS is paired with shock only during this phase. Moreover, the ID subject exhibits elevated arousal states ($x_{est}$) in the early extinction phase, consistent with previous findings that individuals with ID demonstrate delayed extinction learning [26].

### Between-group comparisons of arousal dynamics across phases

From Table 2, ID subjects exhibit a lower mean HAI ($\bar{h}$) during the conditioning phase compared to GS subjects, suggesting a muted physiological response upon initial exposure to fear. However, as shown in Table 4, the ID group shows a significantly greater increase in the mean HAI ($\Delta \bar{h}$) from the conditioning to the extinction phase than that observed in the GS group. This indicates that individuals with ID experience heightened arousal during extinction, which is potentially associated with impaired fear extinction learning—specifically, a difficulty in learning that the CS is no longer associated with danger.

Furthermore, ID subjects exhibit a higher mean arousal state ($\bar{x}$) during the recall phase compared to GS subjects (Table 2), alongside a smaller phase-to-phase decrease in the mean arousal state ($\Delta \bar{x}$) from conditioning to recall (Table 4), suggesting a heightened fear response during recall. Collectively, these findings suggest that ID may be linked to difficulties for extinction memories to oppose fear memories, resulting in weaker fear extinction recall and a reduced capacity to extinguish physiological responses to past trauma.

### Characterization of ID and its applications

Overall, our findings suggest that ID may potentially be associated with disrupted fear regulation. This builds upon previous studies showing different physiological responses during fear extinction in individuals with ID [26,27]. Futhermore, this study provides supporting evidence that sleep disturbances could serve as a risk factor for the development of mental health disorders such as PTSD and anxiety disorders, in line with previous research [1,2,6,25,30]. This line of research frames disrupted sleep as potentially interfering with extinction learning, and our results are consistent with this view. This highlights the potential broader clinical implications for fear-centered conditions like PTSD and anxiety, where impaired extinction can hinder both recovery and treatment.

By estimating the arousal state, we gain deeper insight into disrupted fear regulation in individuals with ID. This understanding holds potential for identifying physiological features to aid in the diagnosis and assessment of ID. For example, arousal state metrics could potentially be used to evaluate alterations in emotion regulation, identify individuals at risk for ID, and assess sleep quality. Such applications may potentially support the development and monitoring of personalized treatment strategies, ultimately reducing the risk of comorbid mental health disorders and improving overall quality of life. Furthermore, research on arousal state estimation can be extended to other sleep-related metrics, such as REM sleep duration, as well as to the study of other mental health conditions.

### Impact of tonic component on arousal estimation

From the estimation results, we observe that the MPP+Cont model captures more subtle fluctuations in arousal states compared to the MPP model, consistent with the findings in [52]. This results in a clearer distinction in arousal states between the ID and GS groups under the MPP+Cont model, whereas the MPP model fails to reveal such group differences. These results highlight the importance of the tonic component in capturing differences in arousal levels between individuals with ID and GS. Since the tonic component represents the general changes in autonomic arousal [64], it may reflect sustained arousal levels over a longer time scale. By capturing this baseline, the MPP+Cont model may be better suited for quantifying long-term arousal dynamics. In contrast, the MPP model, which excludes the tonic component, may be better suited for estimating short-term or momentary arousal state. For individuals with ID, who may struggle to consolidate extinction memories, the tonic component remains elevated. This explains why group differences in arousal states are observed only when using the MPP+Cont model, which includes the tonic component.

## Conclusion

In this study, we estimate the underlying sympathetic arousal state to characterize alterations in fear regulation among individuals with ID. Using Bayesian filtering, we infer the arousal state over time from ANS activations and the tonic component of SC measurements. Statistical analyses reveal that individuals with ID exhibit a significantly greater increase in mean HAI from the conditioning to the extinction phase, suggesting an association with impaired fear extinction learning. Additionally, individuals with ID show a smaller decrease in the mean arousal state from the conditioning to the recall phase, suggesting a link to weaker fear extinction recall. Collectively, these altered arousal dynamics reveal the association between ID and disrupted fear regulation. Moreover, we demonstrate that the tonic component of SC may contain critical information about arousal levels and is essential for capturing differences between ID and GS individuals. This study provides a deeper understanding of disrupted fear regulation in ID and highlights the potential of arousal state estimation as a physiological feature for diagnosing and assessing ID. Beyond ID, our results align with the view that sleep disruption may impair extinction learning and consolidation, highlighting broader implications for the recovery and treatment of conditions such as PTSD and anxiety.

### Limitations and future work

A limitation of this study is the relatively small sample size, especially after phase-specific exclusions. This reduces statistical power required to reliably detect small group differences (|*r*| < 0.3). Consequently, for findings where non-significant *p*-values coincide with medium effect sizes, we cannot definitively rule out the presence of a true physiological effect, which may limit the stability of the reported findings. Given these limitations, this work serves primarily as a methodological proof-of-concept for utilizing MPP+Cont model in characterizing physiological arousal dynamics. Future research should consider large-scale validation to achieve the robust statistical power necessary to confirm these effects.

Moreover, while this study focuses on SC measurements, other physiological signals, such as heart rate, may offer complementary insights into arousal. Future work could explore the integration of multiple physiological modalities to enhance the robustness and reliability of arousal state estimation.

## Supporting information

S1 Data SC dataset including deconvolved ANS activations and the tonic component. (CSV)

S1 File Details of the Expectation-Maximization algorithm. (PDF)

S2 File Secondary analysis results. (PDF)

S3 File Estimated arousal states for all subjects. (PDF)
