# Supplementary material for: Disrupted fear regulation in insomnia disorder revealed by arousal estimation from skin conductance
Source: PLOS Ment Health. 2026 Oct 14;3(10):e0000720. doi: 10.1371/journal.pmen.0000720 (PMC13667829; doi:10.1371/journal.pmen.0000720)
Supplement: Supplementary file 1 [file pmen.0000720.s002.pdf]

S1 File. Details of the Expectation-Maximization algorithm

E-step

Here, we describe the E-step in detail based on the MPP+Cont model [1]. In the E-step, we apply Bayesian filtering and smoothing to estimate the arousal states $\{x_k\}_{k=0}^K$ and their corresponding variances $\{\sigma_k^2\}_{k=0}^K$, given the observations $\{(n_k, r_k, s_k)\}_{k=1}^K$ and the model parameters $\boldsymbol{\Theta}$ estimated from the previous M-step. The equations for Bayesian filtering are given by:

$$x_{k|k-1} = x_{k-1|k-1} \qquad \text{(predicted state estimate)}$$

$$\sigma^2_{k|k-1} = \sigma^2_{k-1|k-1} + \sigma_x^2 \qquad \text{(predicted estimate variance)}$$

$$p_{k|k} = \frac{1}{1 + e^{-(\beta_0 + \beta_1 x_{k|k})}}$$

$$C_k = \begin{cases} \frac{\sigma^2_{k|k-1}}{(\gamma_1^2\sigma_s + \delta_1^2\sigma_r)\sigma^2_{k|k-1} + \sigma_r^2\sigma_s^2}, & n_k = 1 \\ \frac{\sigma^2_{k|k-1}}{\delta_1^2\sigma^2_{k|k-1} + \sigma_s^2}, & n_k = 0 \end{cases}$$

$$x_{k|k} = \begin{cases} x_{k|k-1} + C_k[\sigma_r^2\sigma_s^2\beta_1(1 - p_{k|k}) + \gamma_1\sigma_s^2(r_k - \gamma_0 - \gamma_1 x_{k|k-1}) \\ \quad +\delta_1\sigma_r^2(s_k - \delta_0 - \delta_1 x_{k|k-1})], & n_k = 1 \\ x_{k|k-1} + C_k[-\sigma_s^2\beta_1 p_{k|k} + \delta_1(s_k - \delta_0 - \delta_1 x_{k|k-1})], & n_k = 0 \end{cases} \qquad \text{(updated state estimate)}$$

$$\sigma^2_{k|k} = \begin{cases} [\sigma^{-2}_{k|k-1} + \beta_1^2 p_{k|k}(1 - p_{k|k}) + \gamma_1^2\sigma_r^{-2} + \delta_1^2\sigma_s^{-2}]^{-1}, & n_k = 1 \\ [\sigma^{-2}_{k|k-1} + \beta_1^2 p_{k|k}(1 - p_{k|k}) + \delta_1^2\sigma_s^{-2}]^{-1}, & n_k = 0 \end{cases} \qquad \text{(updated estimate variance)}$$

while the equations for smoothing are given by:

$$x_{k|K} = x_{k|k} + \frac{\sigma^2_{k|k}}{\sigma^2_{k+1|k}}(x_{k+1} - x_{k+1|k}) \qquad \text{(smoothed state estimate)}$$

$$\sigma^2_{k|K} = \sigma^2_{k|k} + \left(\frac{\sigma^2_{k|k}}{\sigma^2_{k+1|k}}\right)^2(\sigma^2_{k+1} - \sigma^2_{k+1|k}) \qquad \text{(smoothed estimate variance)}$$

$$x_k = x_{k|K} \qquad \text{(estimated state)}$$

$$\sigma_k^2 = \sigma^2_{k|K} \qquad \text{(estimated state variance)}$$

Note that when estimating the updated state $x_{k|k}$, the state appears on both sides of the equation since $p_{k|k}$ also depends on $x_{k|k}$. As a result, $x_{k|k}$ is computed iteratively using Newton's method. The initial state and variance are set as $x_{0|0} = 0$ and $\sigma^2_{0|0} = \sigma_x^2$.

M-step

Here, we describe the M-step in detail based on the MPP+Cont model [1]. In the M-step, we estimate the model parameters $\boldsymbol{\Theta}$ by maximizing the expected log-posterior, given the observations $\{(n_k, r_k, s_k)\}_{k=1}^K$ as well as the states $\{x_k\}_{k=0}^K$ and their variances $\{\sigma_k^2\}_{k=0}^K$ estimated from the previous E-step. The expected

log-posterior is given by:

$$
\begin{aligned}
&\mathbb{E}[\log p(\{x_k\}_{k=1}^K | \{(n_k, r_k, s_k)\}_{k=1}^K, \mathbf{\Theta})] \\
&= \sum_{k=1}^{K} \mathbb{E}\left[n_k \log(p_k) + (1-n_k)\log(1-p_k)\right] \\
&\quad - \frac{\sum_{k=1}^K n_k}{2}\log(2\pi\sigma_r^2) - \frac{1}{2\sigma_r^2}\sum_{k=1}^{K} n_k \mathbb{E}\left[(r_k - \gamma_0 - \gamma_1 x_k)^2\right] \\
&\quad - \frac{K}{2}\log(2\pi\sigma_s^2) - \frac{1}{2\sigma_s^2}\sum_{k=1}^{K} \mathbb{E}\left[(s_k - \delta_0 - \delta_1 x_k)^2\right] \\
&\quad - \frac{K}{2}\log(2\pi\sigma_x^2) - \frac{1}{2\sigma_x^2}\sum_{k=1}^{K} \mathbb{E}\left[(x_k - x_{k-1})^2\right] \\
&\quad + c
\end{aligned}
$$

where c is a constant independent of $\mathbf{\Theta}$, and the expectations and covariances are given by:

$$
\begin{aligned}
\mathbb{E}[x_k] &= x_k \\
\mathbb{E}[x_k^2] &= x_k^2 + \sigma_k^2 \\
\mathbb{E}[x_k x_{k-1}] &= x_k x_{k-1} + \frac{\sigma_{k-1|k-1}^2}{\sigma_{k|k-1}^2}\sigma_k^2
\end{aligned}
$$

The equations for the model parameters that maximize the expected log-posterior are given by:

$$
\begin{aligned}
\sigma_x^2 &= \frac{1}{K}\sum_{k=1}^{K}[(x_k^2 + \sigma_k^2) - 2(x_k x_{k-1} + \frac{\sigma_{k-1|k-1}^2}{\sigma_{k|k-1}^2}\sigma_k^2) + (x_{k-1}^2 + \sigma_{k-1}^2)] \\
\begin{bmatrix}\gamma_0 \\ \gamma_1\end{bmatrix} &= \begin{bmatrix}\sum_{k=1}^K n_k & \sum_{k=1}^K n_k x_k \\ \sum_{k=1}^K n_k x_k & \sum_{k=1}^K n_k (x_k^2 + \sigma_k^2)\end{bmatrix}^{-1} \begin{bmatrix}\sum_{k=1}^K n_k r_k \\ \sum_{k=1}^K n_k r_k x_k\end{bmatrix} \\
\sigma_r^2 &= \frac{1}{\sum_{k=1}^K n_k}\sum_{k=1}^{K} n_k[r_k^2 + \gamma_0^2 + \gamma_1^2(x_k^2 + \sigma_k^2) - 2\gamma_0 r_k - 2\gamma_1 r_k x_k + 2\gamma_0\gamma_1 x_k] \\
\begin{bmatrix}\delta_0 \\ \delta_1\end{bmatrix} &= \begin{bmatrix}K & \sum_{k=1}^K x_k \\ \sum_{k=1}^K x_k & \sum_{k=1}^K (x_k^2 + \sigma_k^2)\end{bmatrix}^{-1} \begin{bmatrix}\sum_{k=1}^K s_k \\ \sum_{k=1}^K s_k x_k\end{bmatrix} \\
\sigma_s^2 &= \frac{1}{K}\sum_{k=1}^{K}[s_k^2 + \delta_0^2 + \delta_1^2(x_k^2 + \sigma_k^2) - 2\delta_0 s_k - 2\delta_1 s_k x_k + 2\delta_0\delta_1 x_k]
\end{aligned}
$$

References

[1] Wickramasuriya DS, Khazaei S, Kiani R, Faghih RT. A Bayesian filtering approach for tracking sympathetic arousal and cortisol-related energy from marked point process and continuous-valued observations. IEEE Access. 2023;11:137204-47. doi:10.1109/ACCESS.2023.3334974.
