# Supplementary material for: Disrupted fear regulation in insomnia disorder revealed by arousal estimation from skin conductance
Source: PLOS Ment Health. 2026 Oct 14;3(10):e0000720. doi: 10.1371/journal.pmen.0000720 (PMC13667829; doi:10.1371/journal.pmen.0000720)
Supplement: Supplementary file 2 [file pmen.0000720.s003.pdf]

S2 File. Secondary analysis results

In this section, we compare the estimated arousal states between groups (ID and GS) and across phases (conditioning, extinction, and recall). We also examine individual phase-to-phase changes within each group and compare these changes between groups. The features extracted for comparison include the mean arousal state $\bar{x}$, the standard deviation of arousal states $\hat{\sigma}_x$, the mean HAI $\bar{h}$, and the standard deviation of HAI $\hat{\sigma}_h$. Comparisons are performed at three levels:

- **Between-group comparisons for each phase:** Assess if the features differ significantly between the ID and GS groups within the same phase using a two-sample Wilcoxon rank-sum test.
- **Within-group comparisons of phase-to-phase changes:** Assess if the features differ significantly across phases within the same group using a paired Wilcoxon signed-rank test.
- **Between-group comparisons of phase-to-phase changes:** Assess if the changes in features across phases differ significantly between the ID and GS groups using a two-sample Wilcoxon rank-sum test.

To control for overestimating the evidence for group differences from multiple testing, the reported p-values were adjusted using the Bonferroni correction across the four features within each phase. The non-parametric effect sizes (r) for all comparisons are also reported.

Between-group comparisons for each phase

Table A and Table B summarize the extracted features ($\bar{x}$, $\hat{\sigma}_x$, $\bar{h}$, $\hat{\sigma}_h$) under the MPP+Cont and MPP models, respectively. For each model, the median, lower quartile, and upper quartile of these features are reported for the ID and GS groups across the conditioning, extinction, and recall phases. The p-values and effect sizes r from Wilcoxon rank-sum tests comparing feature distributions between the ID and GS groups are also provided. Box plots of the corresponding features under the MPP+Cont and MPP models are shown in Fig. A and Fig. B, respectively.

Table A: **Features under the MPP+Cont model for ID and GS groups during each phase.** Median, lower quartile, and upper quartile values of the features are reported. p-values and effect sizes r from Wilcoxon rank-sum tests comparing features between the ID and GS groups are also provided. Significant differences ($p < 0.05$) and medium effect sizes ($|r| \geq 0.3$) are highlighted in bold.

Conditioning				
Feature	ID ($n = 16$)	GS ($n = 17$)	p-value	Effect Size (r)
$\bar{x}$ (a.u.)	-0.329 [-4.513, 0.326]	-3.257 [-5.704, -0.740]	0.8296	0.2195
$\hat{\sigma}_x$ (a.u.)	1.614 [1.123, 3.529]	1.991 [1.601, 4.299]	1.0000	-0.1568
$\bar{h}$ (a.u.)	0.498 [0.489, 0.504]	0.504 [0.500, 0.507]	0.1467	**-0.3637**
$\hat{\sigma}_h$ (a.u.)	0.473 [0.458, 0.478]	0.467 [0.457, 0.481]	1.0000	0.0752
Extinction				
Feature	ID ($n = 11$)	GS ($n = 14$)	p-value	Effect Size (r)
$\bar{x}$ (a.u.)	-4.034 [-8.190, -3.350]	-4.800 [-9.126, -2.045]	1.0000	-0.0985
$\hat{\sigma}_x$ (a.u.)	3.117 [2.761, 6.601]	3.682 [2.397, 6.733]	1.0000	0.0547
$\bar{h}$ (a.u.)	0.499 [0.497, 0.513]	0.500 [0.500, 0.504]	1.0000	-0.0219
$\hat{\sigma}_h$ (a.u.)	0.479 [0.461, 0.483]	0.474 [0.459, 0.489]	1.0000	-0.0876
Recall				
Feature	ID ($n = 17$)	GS ($n = 15$)	p-value	Effect Size (r)
$\bar{x}$ (a.u.)	-3.918 [-7.581, -2.128]	-7.287 [-9.126, -5.351]	0.1444	**0.3705**
$\hat{\sigma}_x$ (a.u.)	3.442 [1.996, 5.650]	4.782 [3.406, 6.282]	0.4683	-0.2770
$\bar{h}$ (a.u.)	0.500 [0.493, 0.501]	0.501 [0.496, 0.507]	1.0000	-0.1569
$\hat{\sigma}_h$ (a.u.)	0.473 [0.448, 0.487]	0.462 [0.445, 0.485]	1.0000	0.0834

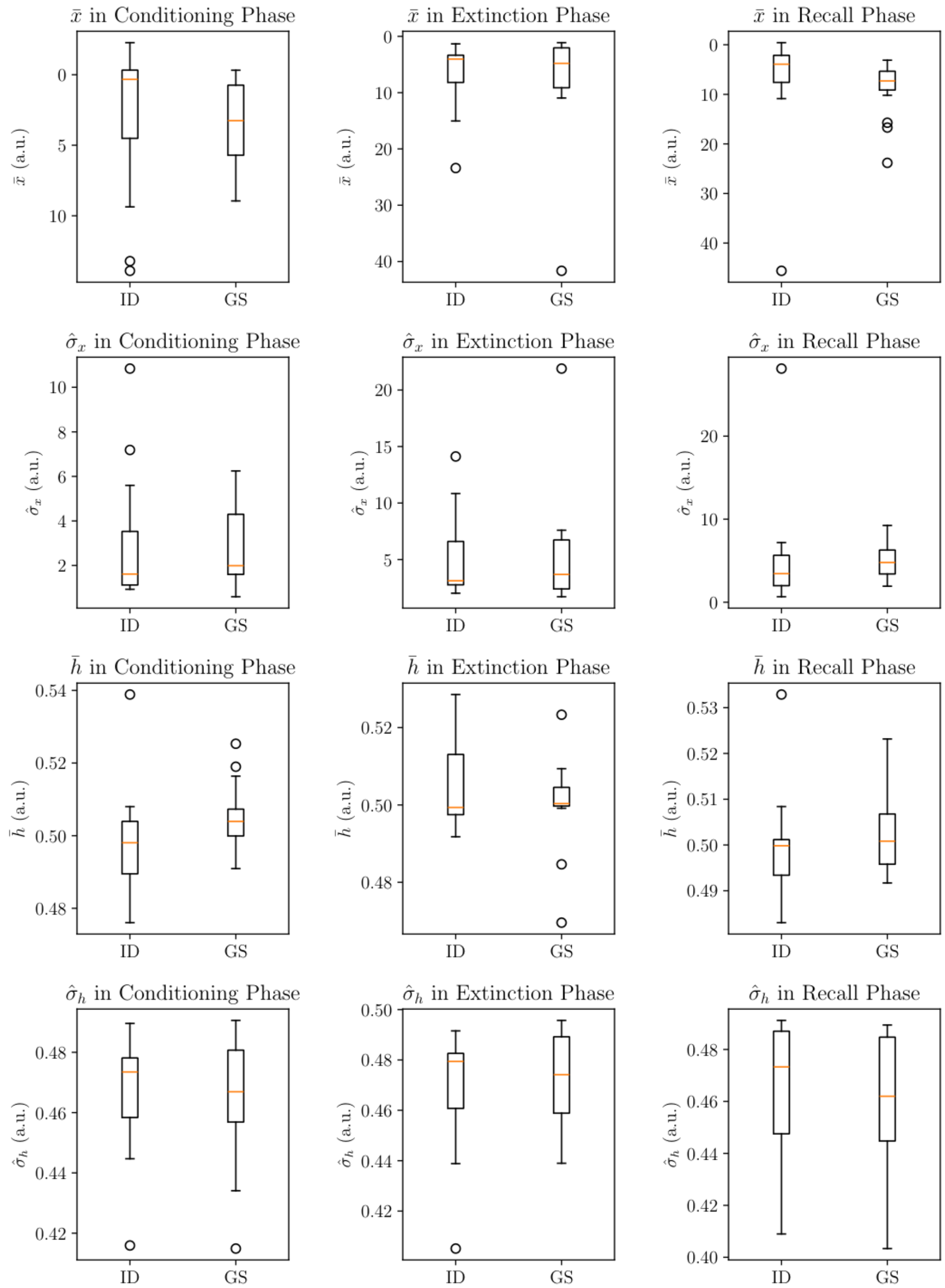

Figure A: **Box plots of features under the MPP+Cont model.** Shown for ID and GS groups during the conditioning (left), extinction (middle), and recall (right) phases.

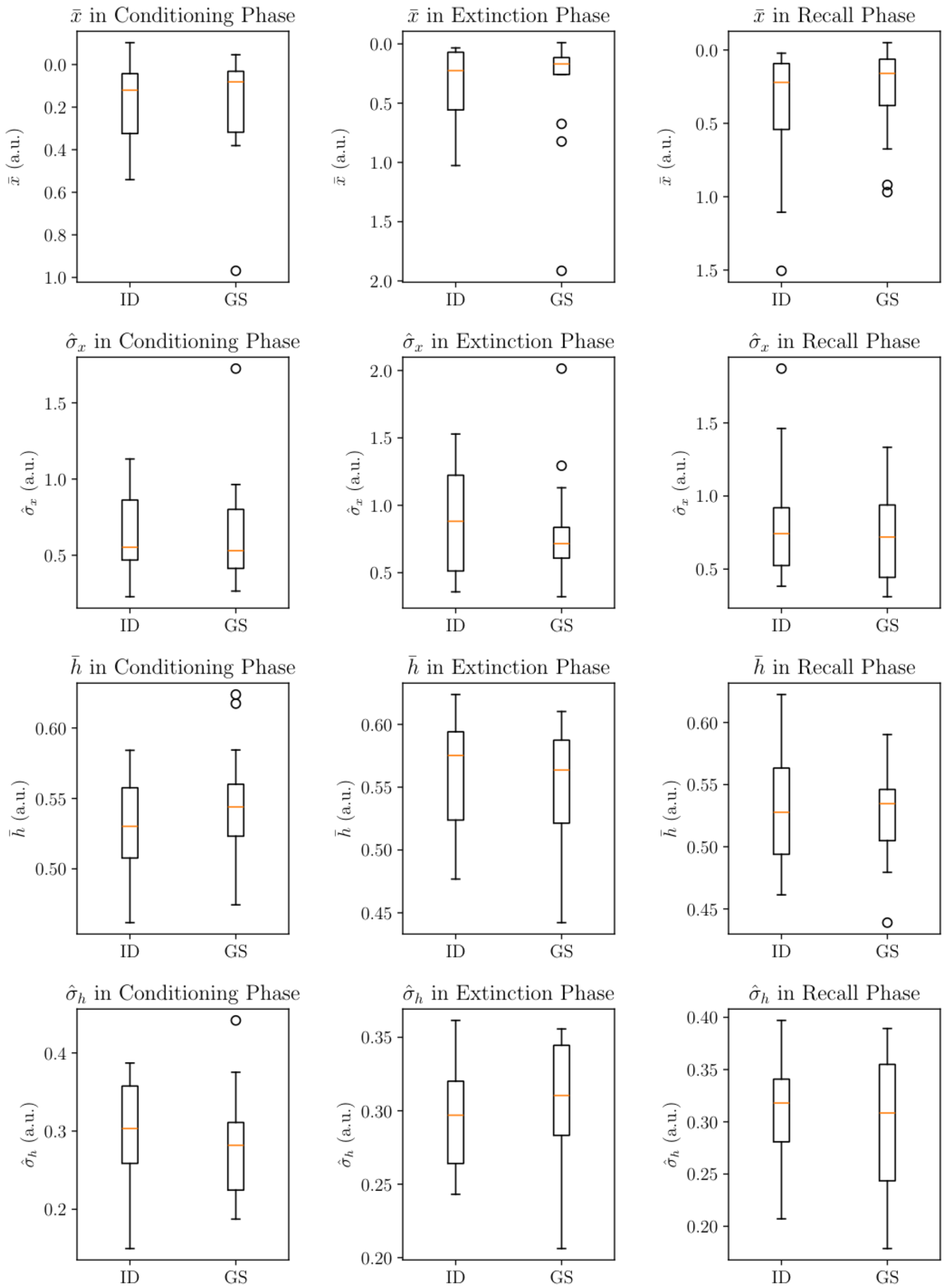

Figure B: **Box plots of features under the MPP model.** Shown for ID and GS groups during the conditioning (left), extinction (middle), and recall (right) phases.

Table B: **Features under the MPP model for ID and GS groups during each phase.** Median, lower quartile, and upper quartile values of the features are reported. p-values and effect sizes r from Wilcoxon rank-sum tests comparing features between the ID and GS groups are also provided. Significant differences ($p < 0.05$) and medium effect sizes ($|r| \geq 0.3$) are highlighted in bold.

Conditioning				
Feature	ID ($n = 16$)	GS ($n = 17$)	p-value	Effect Size (r)
$\bar{x}$ (a.u.)	-0.121 [-0.324, -0.043]	-0.082 [-0.318, -0.032]	1.0000	-0.0878
$\hat{\sigma}_x$ (a.u.)	0.552 [0.468, 0.863]	0.530 [0.414, 0.801]	1.0000	0.0941
$\bar{h}$ (a.u.)	0.530 [0.508, 0.558]	0.544 [0.523, 0.560]	1.0000	-0.1505
$\hat{\sigma}_h$ (a.u.)	0.303 [0.259, 0.358]	0.282 [0.225, 0.311]	1.0000	0.1693
Extinction				
Feature	ID ($n = 11$)	GS ($n = 14$)	p-value	Effect Size (r)
$\bar{x}$ (a.u.)	-0.225 [-0.557, -0.071]	-0.170 [-0.258, -0.116]	1.0000	-0.0876
$\hat{\sigma}_x$ (a.u.)	0.882 [0.513, 1.223]	0.715 [0.608, 0.836]	1.0000	0.1204
$\bar{h}$ (a.u.)	0.575 [0.524, 0.594]	0.564 [0.521, 0.587]	1.0000	0.0985
$\hat{\sigma}_h$ (a.u.)	0.297 [0.264, 0.320]	0.310 [0.283, 0.345]	1.0000	-0.1752
Recall				
Feature	ID ($n = 17$)	GS ($n = 15$)	p-value	Effect Size (r)
$\bar{x}$ (a.u.)	-0.221 [-0.542, -0.092]	-0.159 [-0.379, -0.063]	1.0000	-0.1969
$\hat{\sigma}_x$ (a.u.)	0.743 [0.524, 0.919]	0.719 [0.443, 0.939]	1.0000	0.0634
$\bar{h}$ (a.u.)	0.528 [0.494, 0.563]	0.535 [0.505, 0.546]	1.0000	0.0367
$\hat{\sigma}_h$ (a.u.)	0.318 [0.281, 0.341]	0.308 [0.243, 0.355]	1.0000	0.0968

Within-group and between-group comparisons of phase-to-phase changes

Table C and Table D summarize individual phase-to-phase changes in features ($\Delta\bar{x}$, $\Delta\hat{\sigma}_x$, $\Delta\bar{h}$, $\Delta\hat{\sigma}_h$) under the MPP+Cont and MPP models, respectively. Only subjects with retained data in both phases after exclusion are included in the analysis. For each model, the median, lower quartile, and upper quartile of feature changes are reported for the ID and GS groups across the conditioning-to-extinction, conditioning-to-recall, and extinction-to-recall phase transitions. p-values from paired Wilcoxon signed-rank tests (within-group comparisons) and Wilcoxon rank-sum tests (between-group comparisons) are provided. Effect sizes r from Wilcoxon rank-sum tests (between-group comparisons) are also provided.

Table C: **Changes in features under the MPP+Cont model for ID and GS groups between each phase.** Median, lower quartile, and upper quartile values of the feature changes are reported. p-values from paired Wilcoxon signed-rank tests comparing phase-to-phase changes within the ID and GS groups, as well as p-values and effect sizes r from Wilcoxon rank-sum tests comparing changes between the ID and GS groups, are also provided. Significant differences ($p < 0.05$) and medium effect sizes ($|r| \geq 0.3$) are highlighted in bold.

Conditioning to Extinction				
ΔFeature	ID ($n = 10$)	GS ($n = 14$)	p-value	Effect Size (r)
$\Delta\bar{x}$ (a.u.)	-2.893 [-7.021, 0.136] ($p = 0.9297$)	-2.334 [-5.082, 0.377] ($p = 0.3140$)	1.0000	-0.0478
$\Delta\hat{\sigma}_x$ (a.u.)	1.510 [0.199, 4.533] ($p = 0.7734$)	1.738 [0.226, 3.883] ($p = 0.0811$)	1.0000	0.0000
Δh (a.u.)	0.006 [0.001, 0.025] ($p = 0.0781$)	-0.002 [-0.013, 0.003] ($p = 0.9648$)	**0.0472**	**0.5139**
$\Delta\hat{\sigma}_h$ (a.u.)	0.003 [-0.014, 0.022] ($p = 1.0000$)	0.010 [-0.020, 0.041] ($p = 1.0000$)	1.0000	-0.0837
Conditioning to Recall				
ΔFeature	ID ($n = 14$)	GS ($n = 14$)	p-value	Effect Size (r)
$\Delta\bar{x}$ (a.u.)	-2.020 [-4.392, 0.121] ($p = 1.0000$)	-3.632 [-6.308, -1.868] ($p =$ **0.0005**)	0.4312	**0.3039**
$\Delta\hat{\sigma}_x$ (a.u.)	0.878 [-0.959, 2.269] ($p = 1.0000$)	1.983 [1.339, 2.939] ($p =$ **0.0210**)	0.4729	-0.2952
Δh (a.u.)	0.002 [-0.004, 0.011] ($p = 1.0000$)	-0.004 [-0.011, 0.001] ($p = 0.6123$)	0.6723	0.2605
$\Delta\hat{\sigma}_h$ (a.u.)	-0.002 [-0.016, 0.013] ($p = 1.0000$)	0.004 [-0.028, 0.021] ($p = 1.0000$)	1.0000	-0.0174
Extinction to Recall				
ΔFeature	ID ($n = 10$)	GS ($n = 12$)	p-value	Effect Size (r)
$\Delta\bar{x}$ (a.u.)	-0.001 [-1.721, 3.647] ($p = 1.0000$)	0.336 [-3.076, 1.292] ($p = 1.0000$)	1.0000	0.0843
$\Delta\hat{\sigma}_x$ (a.u.)	-0.441 [-1.323, 1.157] ($p = 1.0000$)	-0.421 [-1.343, 0.924] ($p = 1.0000$)	1.0000	-0.0703
Δh (a.u.)	-0.000 [-0.007, 0.003] ($p = 1.0000$)	0.004 [-0.007, 0.010] ($p = 1.0000$)	1.0000	-0.1968
$\Delta\hat{\sigma}_h$ (a.u.)	0.003 [-0.015, 0.009] ($p = 1.0000$)	-0.015 [-0.028, 0.004] ($p = 1.0000$)	1.0000	0.2249

Table D: **Changes in features under the MPP model for ID and GS groups between each phase.** Median, lower quartile, and upper quartile values of the feature changes are reported. p-values from paired Wilcoxon signed-rank tests comparing phase-to-phase changes within the ID and GS groups, as well as p-values and effect sizes r from Wilcoxon rank-sum tests comparing changes between the ID and GS groups, are also provided. Significant differences ($p < 0.05$) and medium effect sizes ($|r| \geq 0.3$) are highlighted in bold.

Conditioning to Extinction				
ΔFeature	ID ($n = 10$)	GS ($n = 14$)	p-value	Effect Size (r)
$\Delta\bar{x}$ (a.u.)	-0.185 [-0.306, 0.007] ($p = 0.1484$)	-0.102 [-0.150, -0.035] ($p =$ **0.0342**)	1.0000	-0.1315
$\Delta\hat{\sigma}_x$ (a.u.)	0.353 [-0.014, 0.463] ($p = 0.1484$)	0.207 [0.074, 0.311] ($p = 0.3623$)	1.0000	0.1793
Δh (a.u.)	0.040 [0.010, 0.061] ($p = 0.6406$)	0.018 [-0.034, 0.078] ($p = 1.0000$)	1.0000	0.0717
$\Delta\hat{\sigma}_h$ (a.u.)	-0.028 [-0.045, 0.034] ($p = 1.0000$)	0.024 [-0.016, 0.062] ($p = 0.7749$)	1.0000	-0.1912
Conditioning to Recall				
ΔFeature	ID ($n = 14$)	GS ($n = 14$)	p-value	Effect Size (r)
$\Delta\bar{x}$ (a.u.)	-0.090 [-0.304, 0.029] ($p = 0.2705$)	-0.081 [-0.170, 0.037] ($p = 0.9648$)	1.0000	-0.1389
$\Delta\hat{\sigma}_x$ (a.u.)	0.165 [-0.124, 0.328] ($p = 0.9648$)	0.066 [-0.086, 0.222] ($p = 1.0000$)	1.0000	0.0868
Δh (a.u.)	-0.000 [-0.017, 0.036] ($p = 1.0000$)	-0.047 [-0.066, 0.026] ($p = 1.0000$)	0.9289	0.2258
$\Delta\hat{\sigma}_h$ (a.u.)	0.018 [-0.049, 0.038] ($p = 1.0000$)	0.038 [-0.011, 0.063] ($p = 0.6904$)	1.0000	-0.1216
Extinction to Recall				
ΔFeature	ID ($n = 10$)	GS ($n = 12$)	p-value	Effect Size (r)
$\Delta\bar{x}$ (a.u.)	0.061 [-0.117, 0.476] ($p = 1.0000$)	0.088 [-0.053, 0.301] ($p = 0.7051$)	1.0000	-0.0843
$\Delta\hat{\sigma}_x$ (a.u.)	-0.141 [-0.653, 0.083] ($p = 0.7734$)	-0.131 [-0.338, 0.066] ($p = 1.0000$)	1.0000	-0.1265
Δh (a.u.)	-0.059 [-0.082, 0.024] ($p = 1.0000$)	-0.011 [-0.057, 0.001] ($p = 0.4395$)	1.0000	-0.1687
$\Delta\hat{\sigma}_h$ (a.u.)	0.011 [-0.041, 0.037] ($p = 1.0000$)	-0.002 [-0.049, 0.032] ($p = 1.0000$)	1.0000	0.0422
