# Supplementary material for: Disrupted fear regulation in insomnia disorder revealed by arousal estimation from skin conductance
Source: PLOS Ment Health. 2026 Oct 14;3(10):e0000720. doi: 10.1371/journal.pmen.0000720 (PMC13667829; doi:10.1371/journal.pmen.0000720)
Supplement: Supplementary file 3 [file pmen.0000720.s004.pdf]

# S3 File. Estimated arousal states for all subjects

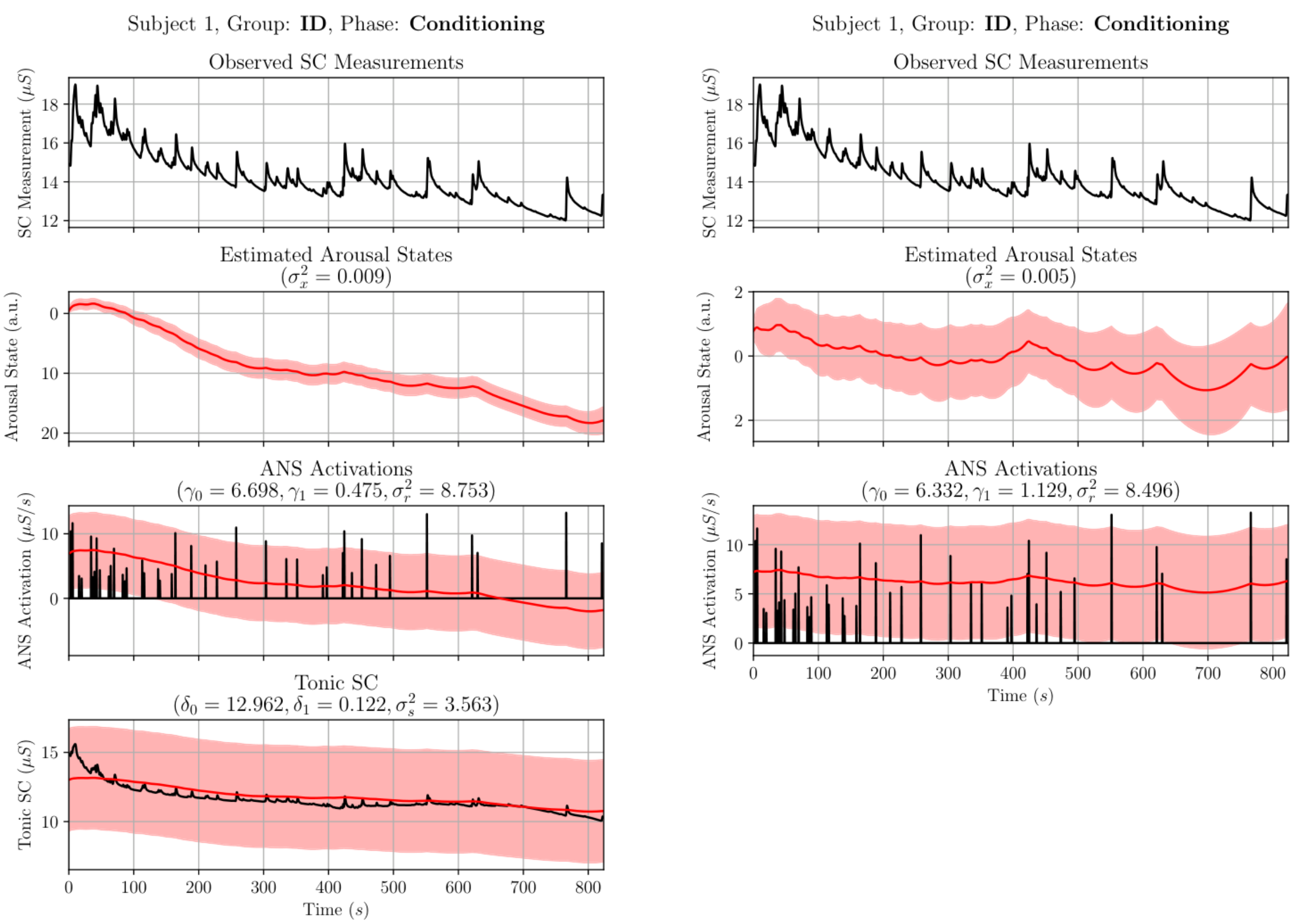


Figure A: **Subject 1 estimated arousal states during the conditioning phase.** Shown for the MPP+Cont model (left) and the MPP model (right). The first panels show the observed SC measurements, $y$ (black). The second panels show the estimated arousal states, $x_{est}$ (red). The third panels show the ANS activations, $r_{obs}$ (black) and the estimated amplitudes, $r_{est}$ (red). The fourth panel shows the tonic component, $s_{obs}$ (black) and the estimated tonic component, $s_{est}$ (red). Estimates are shown with 95% confidence intervals (CI) (red shaded area). Estimated parameters are reported in the title of each panel.

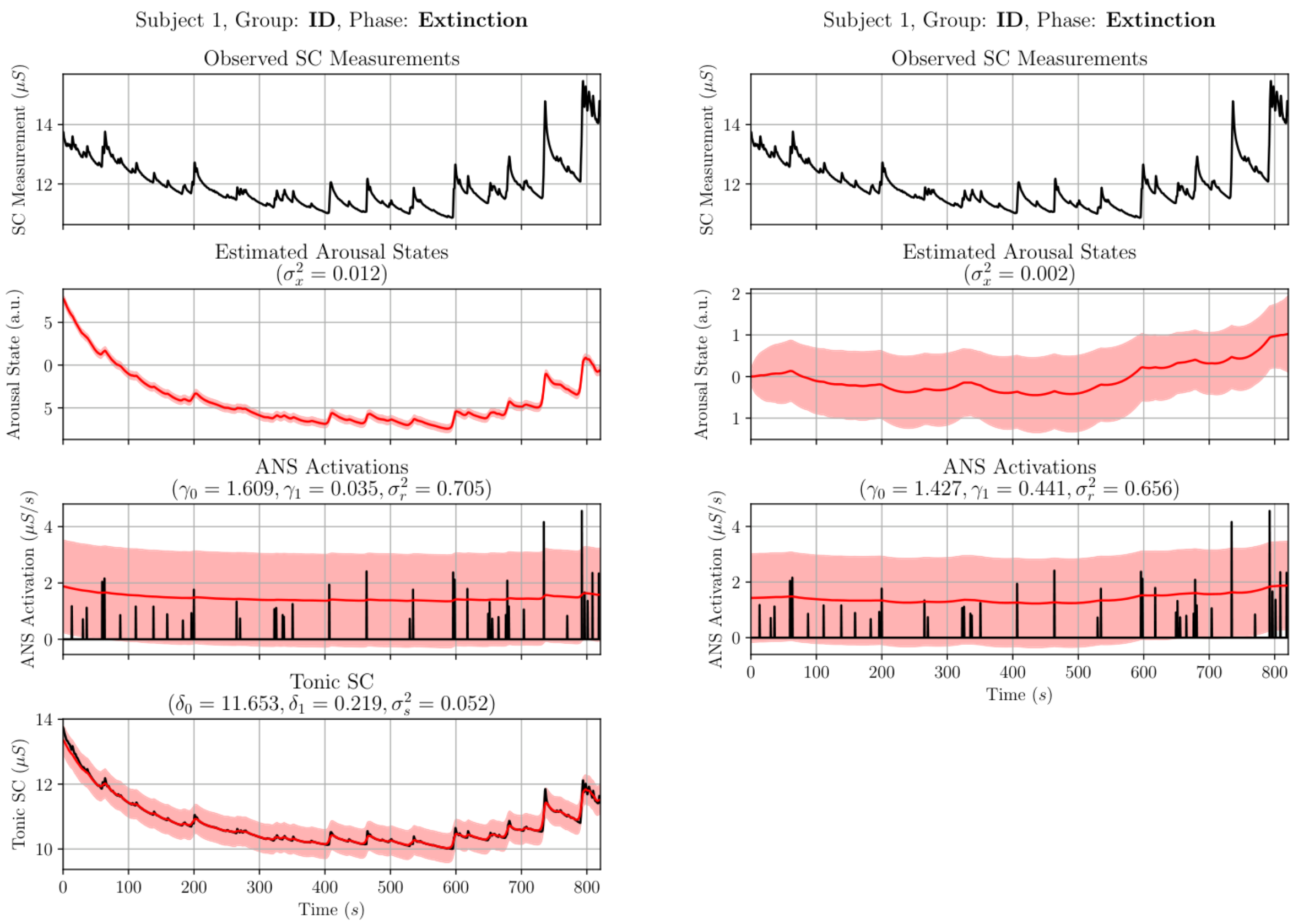


Figure B: **Subject 1 estimated arousal states during the extinction phase.** Shown for the MPP+Cont model (left) and the MPP model (right). The first panels show the observed SC measurements, $y$ (black). The second panels show the estimated arousal states, $x_{est}$ (red). The third panels show the ANS activations, $r_{obs}$ (black) and the estimated amplitudes, $r_{est}$ (red). The fourth panel shows the tonic component, $s_{obs}$ (black) and the estimated tonic component, $s_{est}$ (red). Estimates are shown with 95% confidence intervals (CI) (red shaded area). Estimated parameters are reported in the title of each panel.

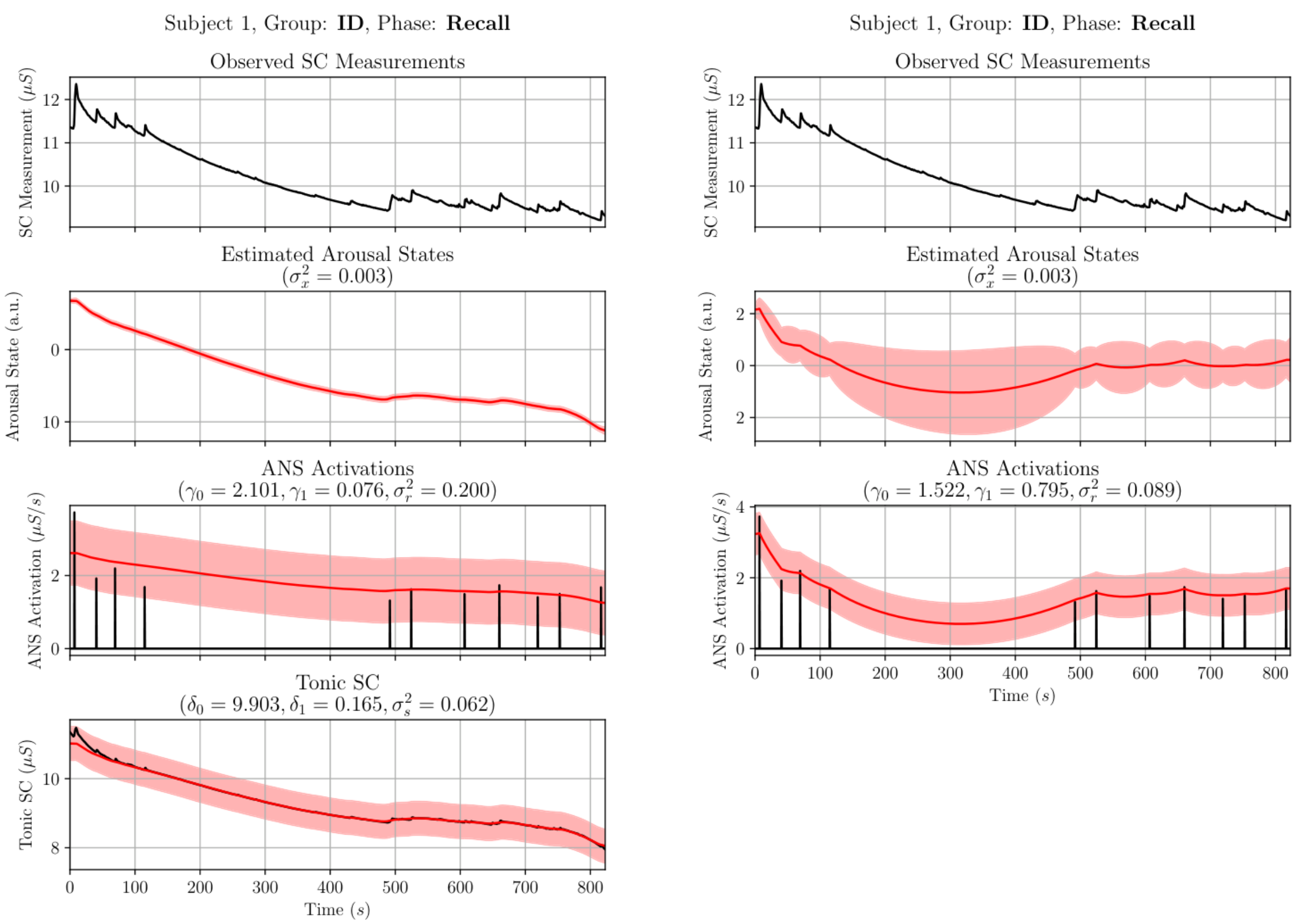


Figure C: **Subject 1 estimated arousal states during the recall phase.** Shown for the MPP+Cont model (left) and the MPP model (right). The first panels show the observed SC measurements, $y$ (black). The second panels show the estimated arousal states, $x_{est}$ (red). The third panels show the ANS activations, $r_{obs}$ (black) and the estimated amplitudes, $r_{est}$ (red). The fourth panel shows the tonic component, $s_{obs}$ (black) and the estimated tonic component, $s_{est}$ (red). Estimates are shown with 95% confidence intervals (CI) (red shaded area). Estimated parameters are reported in the title of each panel.

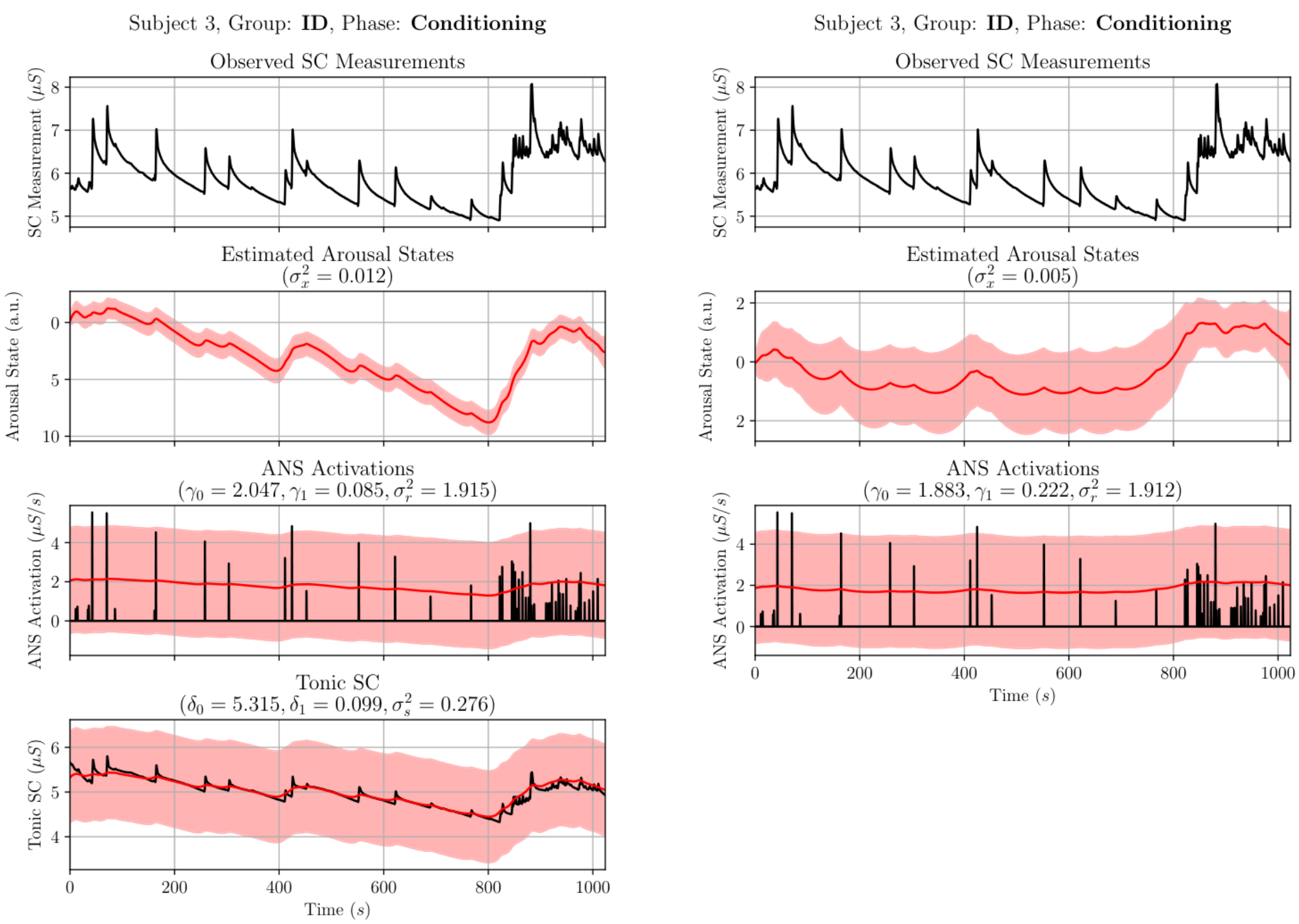


Figure D: **Subject 3 estimated arousal states during the conditioning phase.** Shown for the MPP+Cont model (left) and the MPP model (right). The first panels show the observed SC measurements, $y$ (black). The second panels show the estimated arousal states, $x_{est}$ (red). The third panels show the ANS activations, $r_{obs}$ (black) and the estimated amplitudes, $r_{est}$ (red). The fourth panel shows the tonic component, $s_{obs}$ (black) and the estimated tonic component, $s_{est}$ (red). Estimates are shown with 95% confidence intervals (CI) (red shaded area). Estimated parameters are reported in the title of each panel.

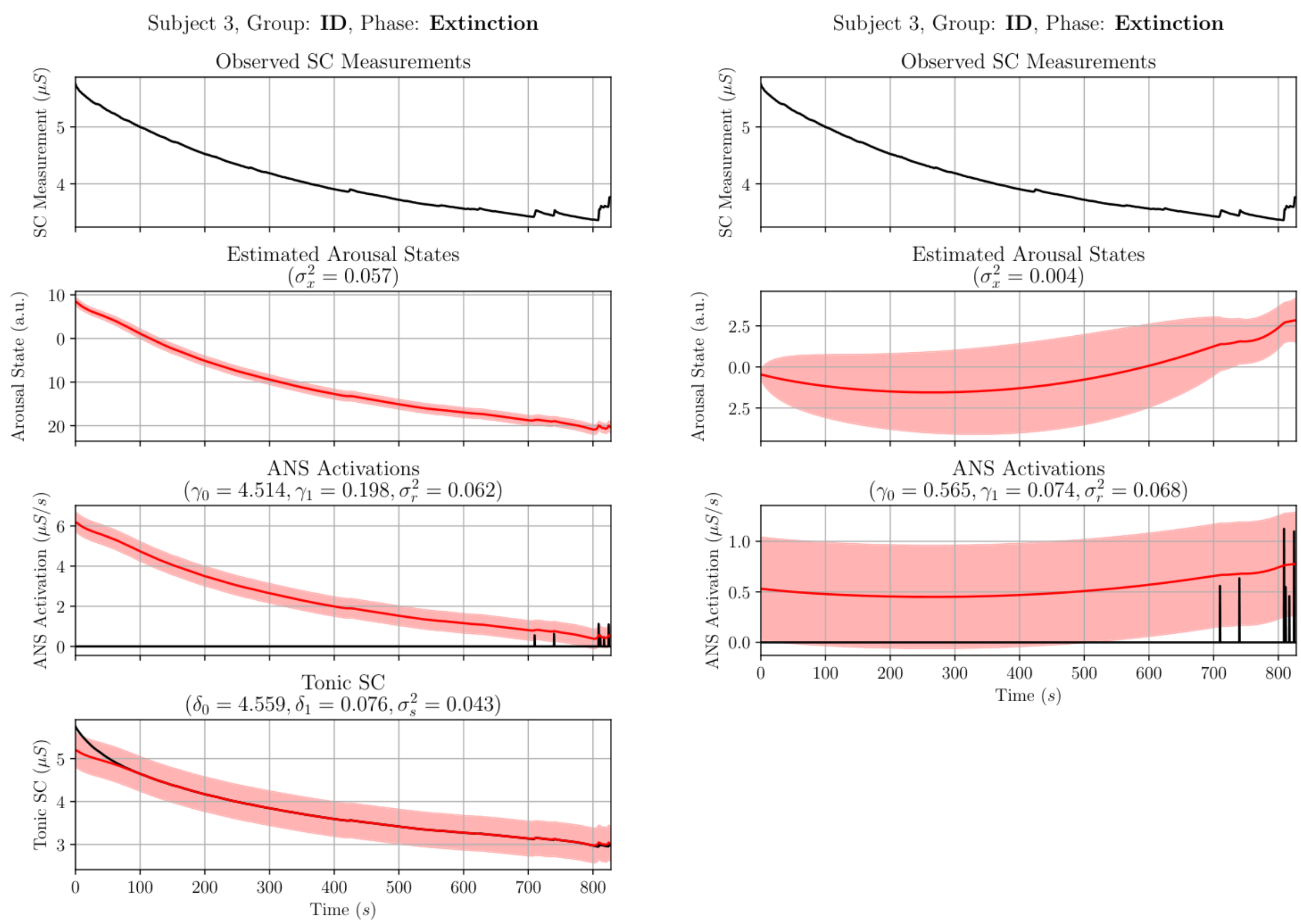


Figure E: **Subject 3 estimated arousal states during the extinction phase.** Shown for the MPP+Cont model (left) and the MPP model (right). The first panels show the observed SC measurements, $y$ (black). The second panels show the estimated arousal states, $x_{est}$ (red). The third panels show the ANS activations, $r_{obs}$ (black) and the estimated amplitudes, $r_{est}$ (red). The fourth panel shows the tonic component, $s_{obs}$ (black) and the estimated tonic component, $s_{est}$ (red). Estimates are shown with 95% confidence intervals (CI) (red shaded area). Estimated parameters are reported in the title of each panel.

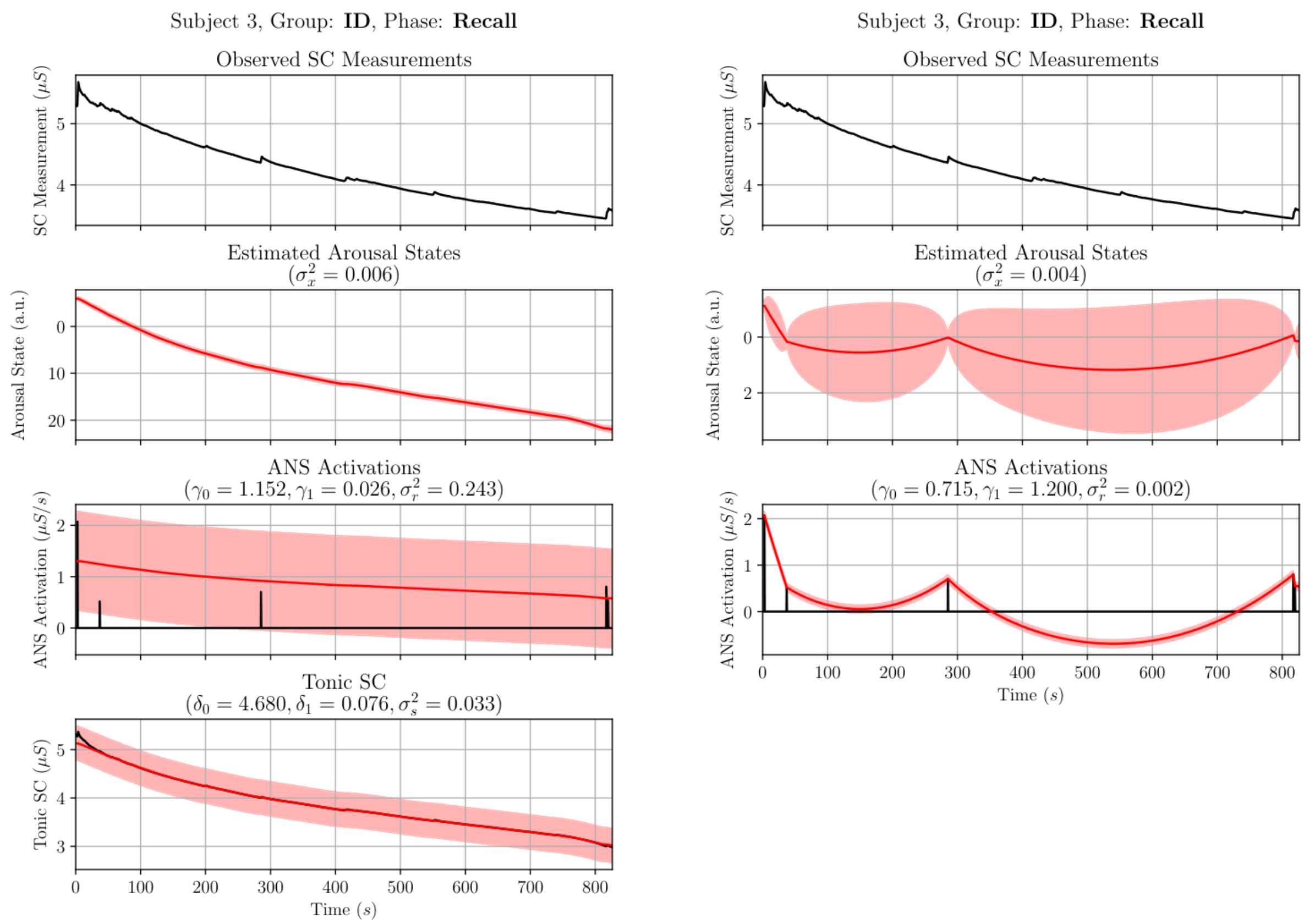


Figure F: **Subject 3 estimated arousal states during the recall phase.** Shown for the MPP+Cont model (left) and the MPP model (right). The first panels show the observed SC measurements, $y$ (black). The second panels show the estimated arousal states, $x_{est}$ (red). The third panels show the ANS activations, $r_{obs}$ (black) and the estimated amplitudes, $r_{est}$ (red). The fourth panel shows the tonic component, $s_{obs}$ (black) and the estimated tonic component, $s_{est}$ (red). Estimates are shown with 95% confidence intervals (CI) (red shaded area). Estimated parameters are reported in the title of each panel.

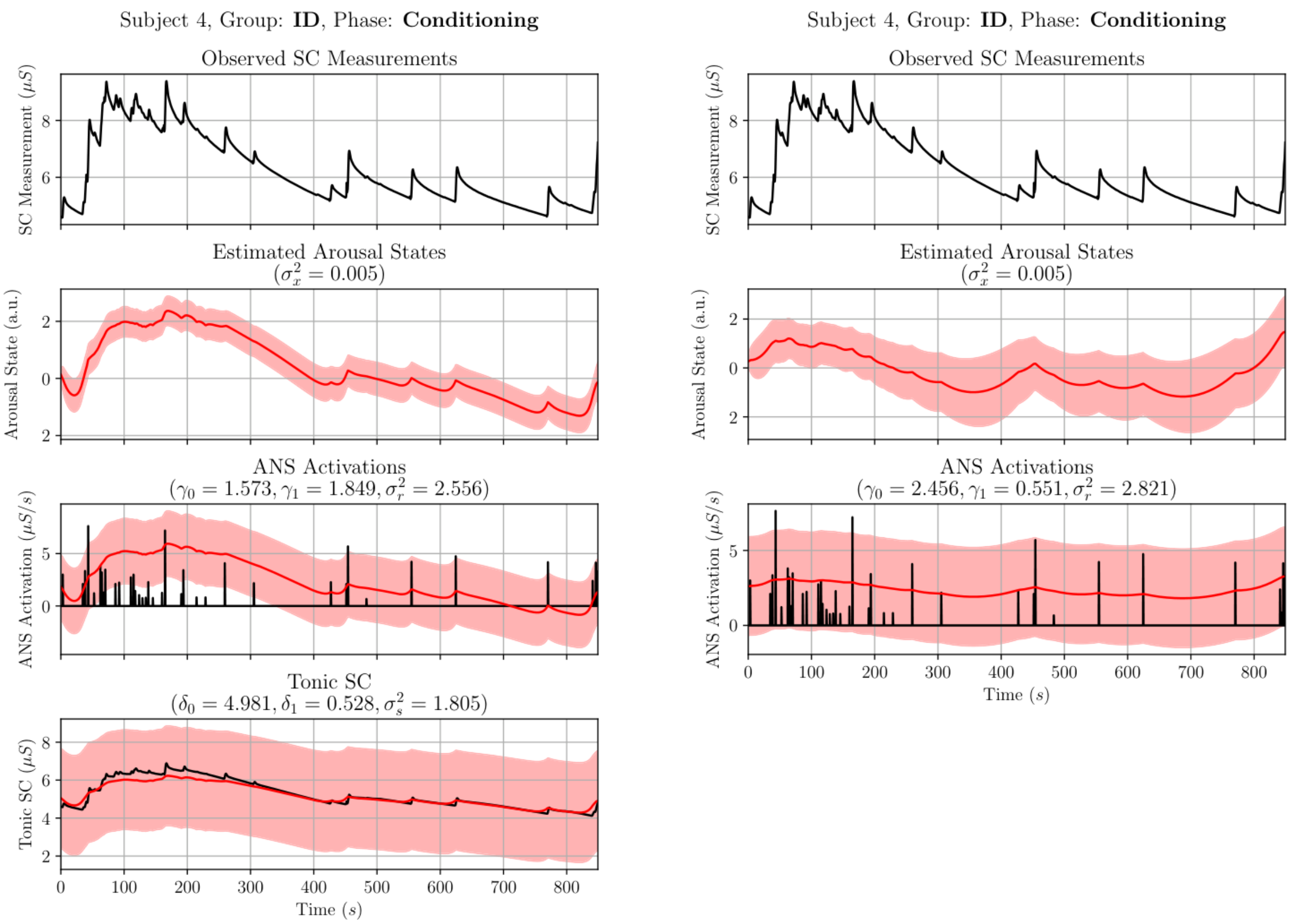


Figure G: **Subject 4 estimated arousal states during the conditioning phase.** Shown for the MPP+Cont model (left) and the MPP model (right). The first panels show the observed SC measurements, $y$ (black). The second panels show the estimated arousal states, $x_{est}$ (red). The third panels show the ANS activations, $r_{obs}$ (black) and the estimated amplitudes, $r_{est}$ (red). The fourth panel shows the tonic component, $s_{obs}$ (black) and the estimated tonic component, $s_{est}$ (red). Estimates are shown with 95% confidence intervals (CI) (red shaded area). Estimated parameters are reported in the title of each panel.

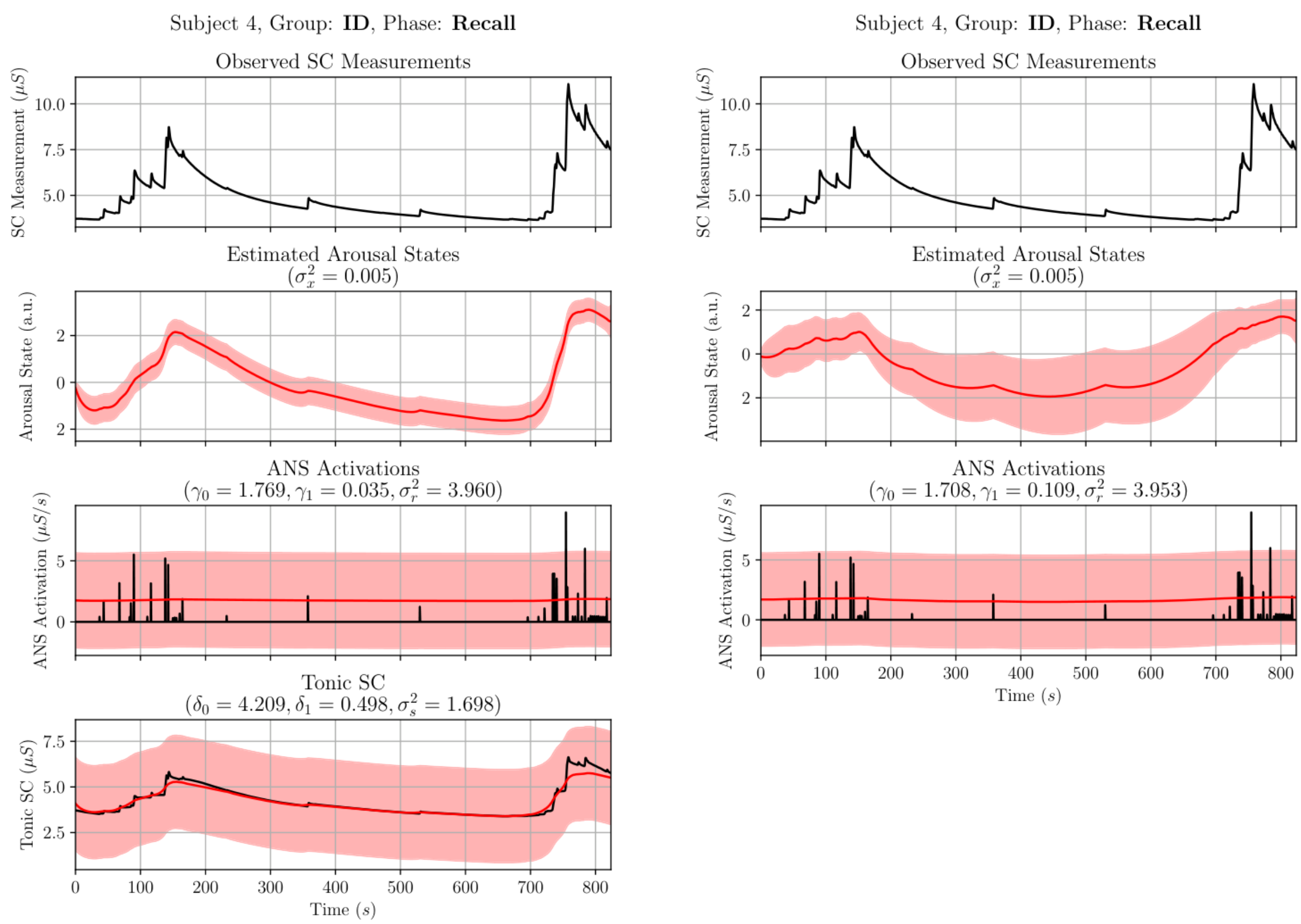


Figure H: **Subject 4 estimated arousal states during the recall phase.** Shown for the MPP+Cont model (left) and the MPP model (right). The first panels show the observed SC measurements, $y$ (black). The second panels show the estimated arousal states, $x_{est}$ (red). The third panels show the ANS activations, $r_{obs}$ (black) and the estimated amplitudes, $r_{est}$ (red). The fourth panel shows the tonic component, $s_{obs}$ (black) and the estimated tonic component, $s_{est}$ (red). Estimates are shown with 95% confidence intervals (CI) (red shaded area). Estimated parameters are reported in the title of each panel.

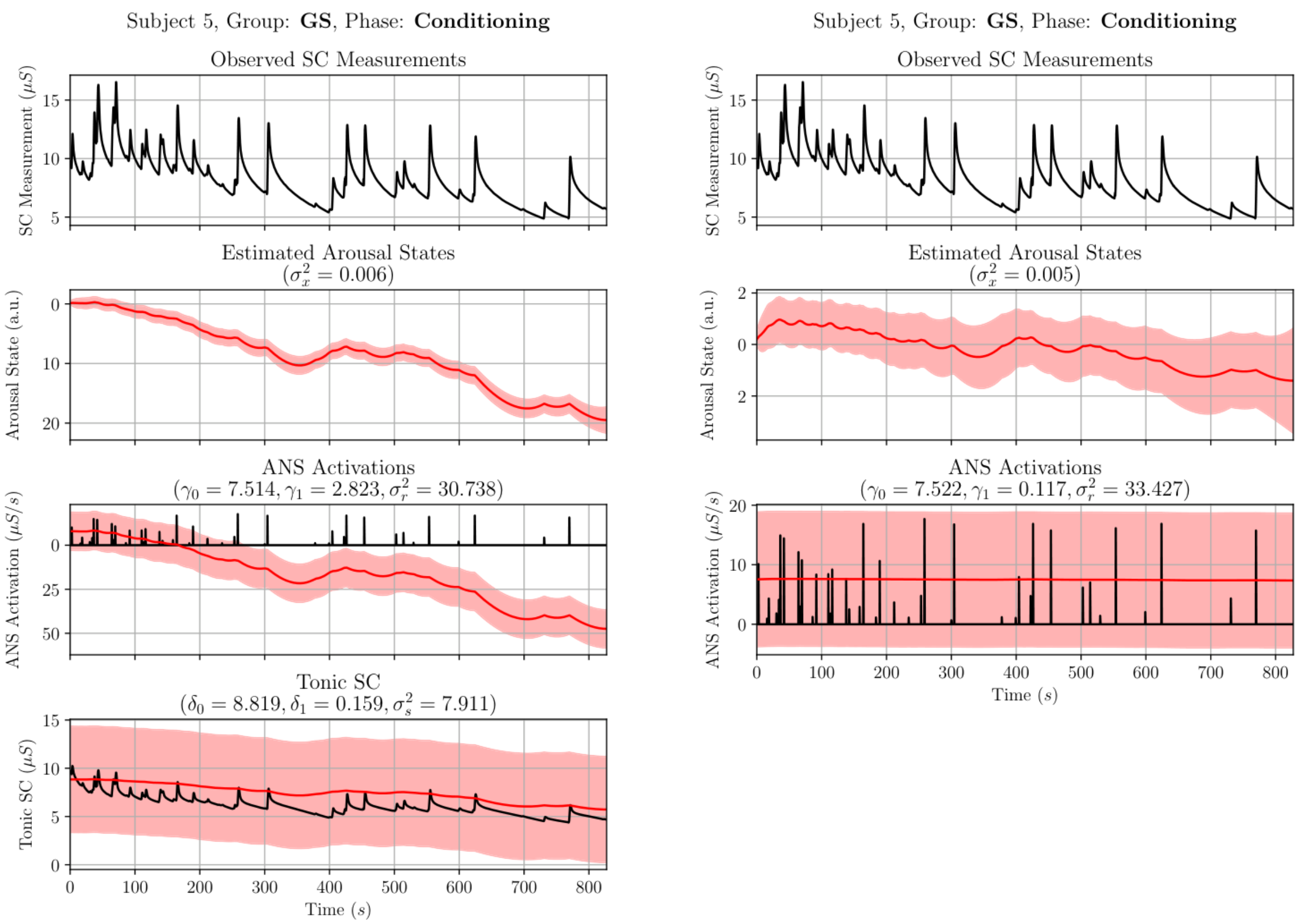


Figure I: **Subject 5 estimated arousal states during the conditioning phase.** Shown for the MPP+Cont model (left) and the MPP model (right). The first panels show the observed SC measurements, $y$ (black). The second panels show the estimated arousal states, $x_{est}$ (red). The third panels show the ANS activations, $r_{obs}$ (black) and the estimated amplitudes, $r_{est}$ (red). The fourth panel shows the tonic component, $s_{obs}$ (black) and the estimated tonic component, $s_{est}$ (red). Estimates are shown with 95% confidence intervals (CI) (red shaded area). Estimated parameters are reported in the title of each panel.

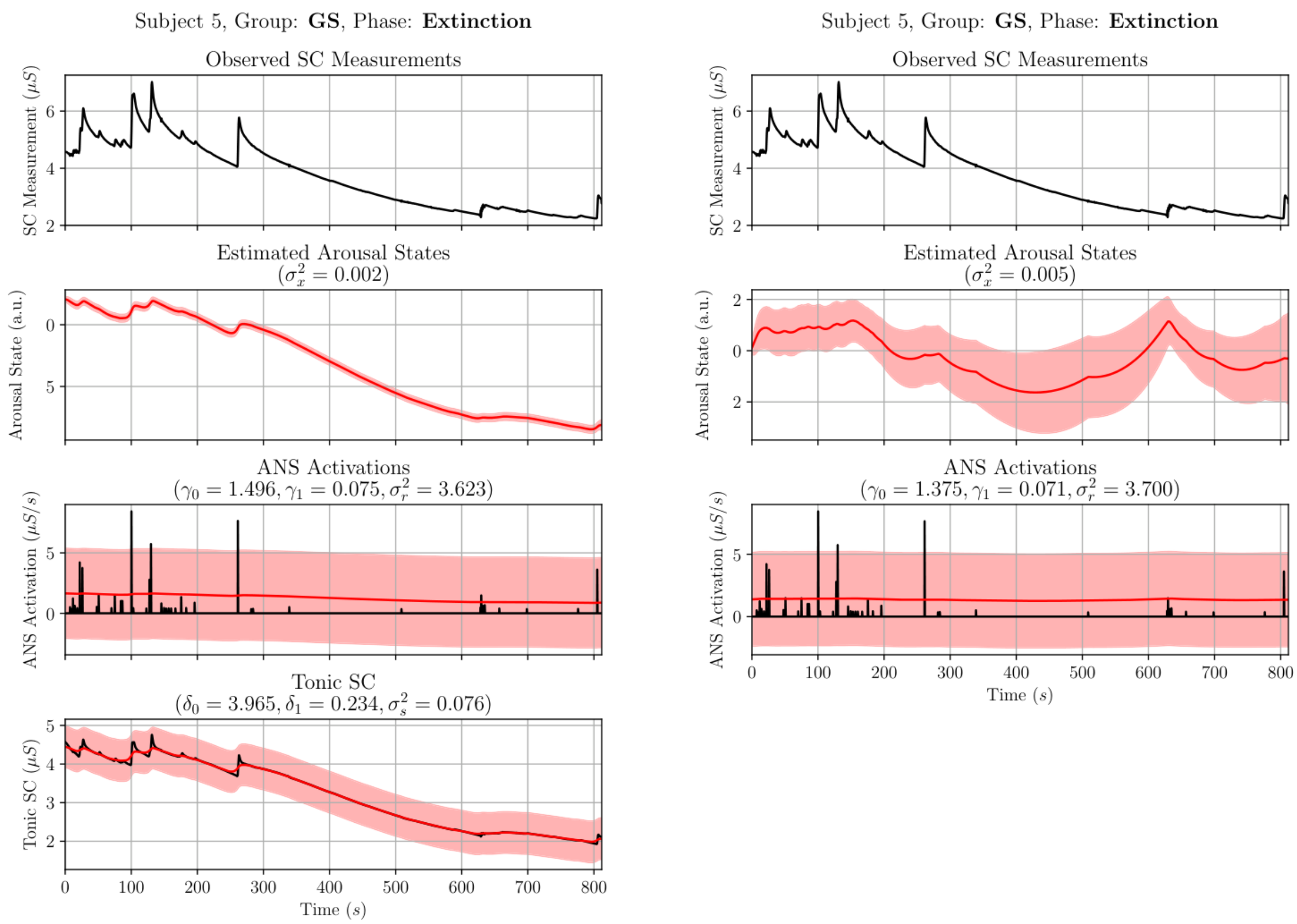


Figure J: **Subject 5 estimated arousal states during the extinction phase.** Shown for the MPP+Cont model (left) and the MPP model (right). The first panels show the observed SC measurements, $y$ (black). The second panels show the estimated arousal states, $x_{est}$ (red). The third panels show the ANS activations, $r_{obs}$ (black) and the estimated amplitudes, $r_{est}$ (red). The fourth panel shows the tonic component, $s_{obs}$ (black) and the estimated tonic component, $s_{est}$ (red). Estimates are shown with 95% confidence intervals (CI) (red shaded area). Estimated parameters are reported in the title of each panel.

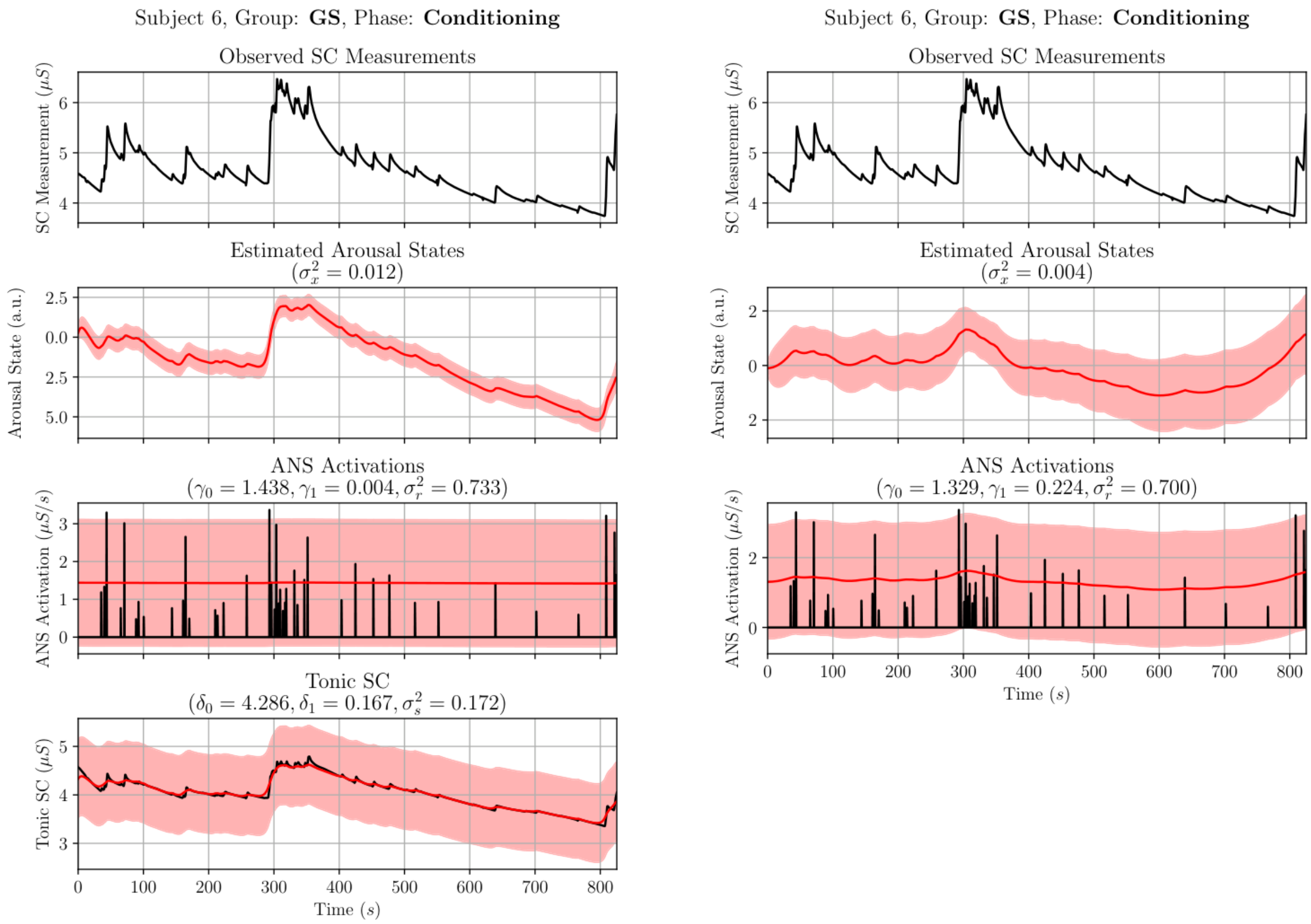


Figure K: **Subject 6 estimated arousal states during the conditioning phase.** Shown for the MPP+Cont model (left) and the MPP model (right). The first panels show the observed SC measurements, $y$ (black). The second panels show the estimated arousal states, $x_{est}$ (red). The third panels show the ANS activations, $r_{obs}$ (black) and the estimated amplitudes, $r_{est}$ (red). The fourth panel shows the tonic component, $s_{obs}$ (black) and the estimated tonic component, $s_{est}$ (red). Estimates are shown with 95% confidence intervals (CI) (red shaded area). Estimated parameters are reported in the title of each panel.

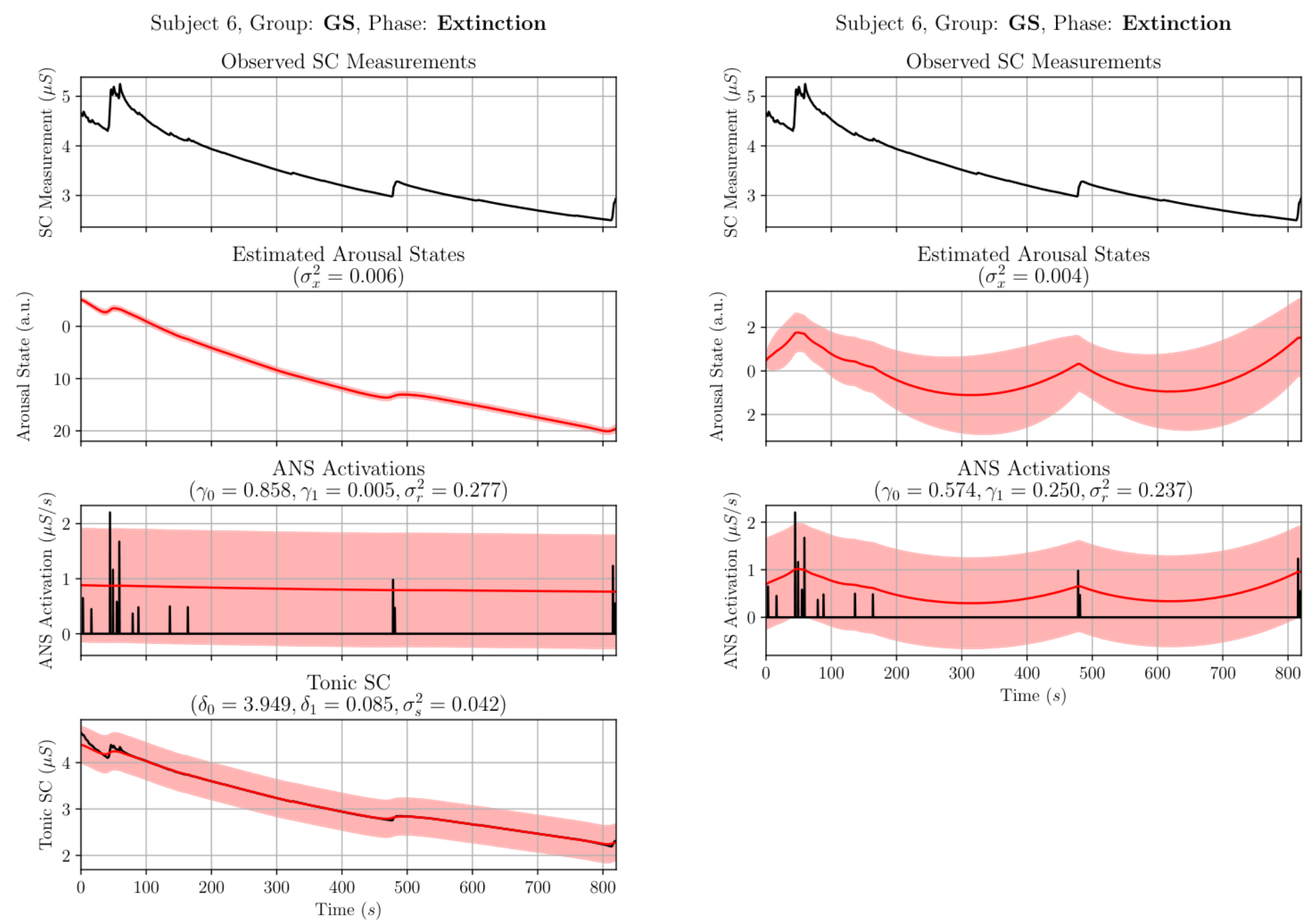


Figure L: **Subject 6 estimated arousal states during the extinction phase.** Shown for the MPP+Cont model (left) and the MPP model (right). The first panels show the observed SC measurements, $y$ (black). The second panels show the estimated arousal states, $x_{est}$ (red). The third panels show the ANS activations, $r_{obs}$ (black) and the estimated amplitudes, $r_{est}$ (red). The fourth panel shows the tonic component, $s_{obs}$ (black) and the estimated tonic component, $s_{est}$ (red). Estimates are shown with 95% confidence intervals (CI) (red shaded area). Estimated parameters are reported in the title of each panel.

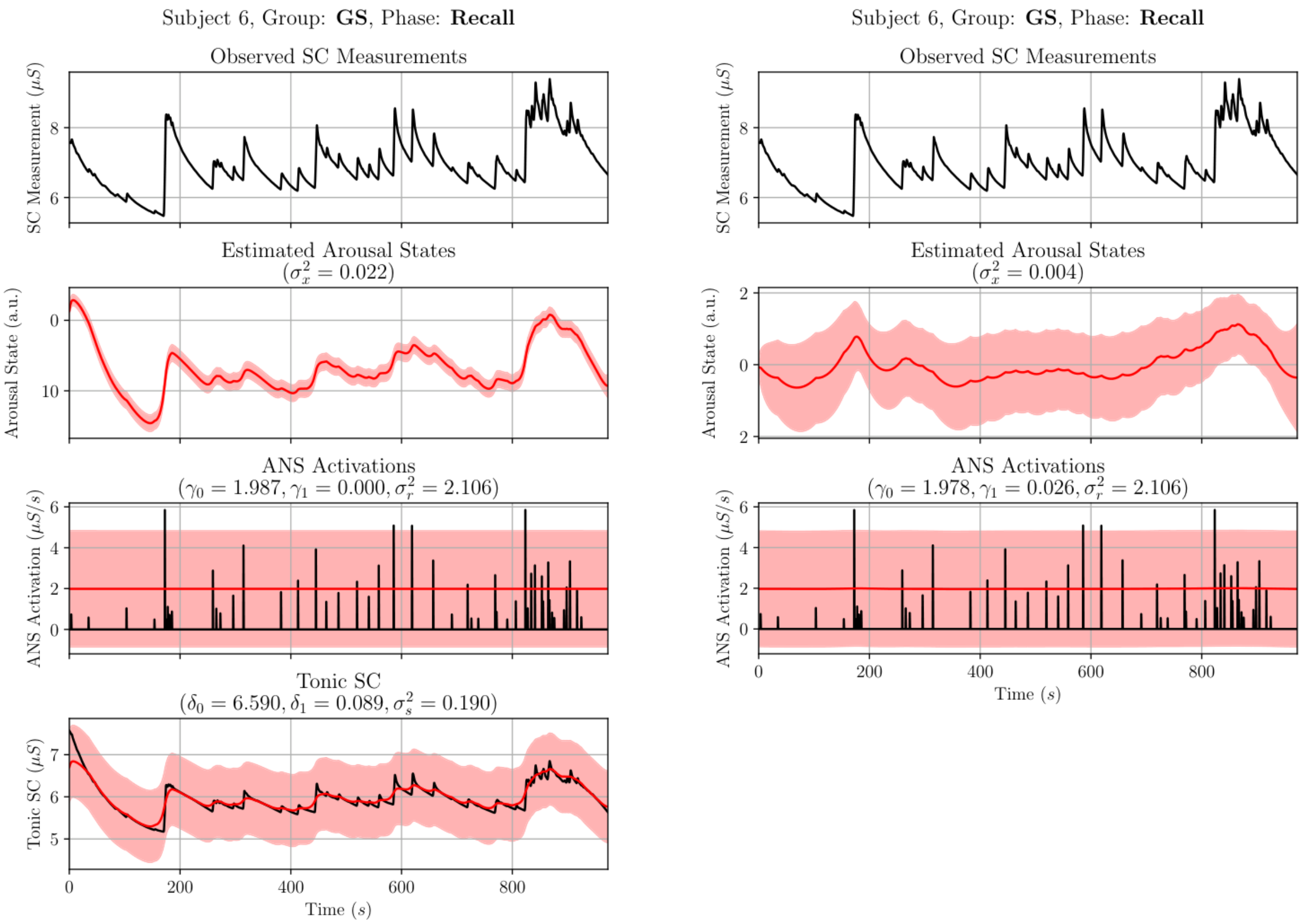


Figure M: **Subject 6 estimated arousal states during the recall phase.** Shown for the MPP+Cont model (left) and the MPP model (right). The first panels show the observed SC measurements, $y$ (black). The second panels show the estimated arousal states, $x_{est}$ (red). The third panels show the ANS activations, $r_{obs}$ (black) and the estimated amplitudes, $r_{est}$ (red). The fourth panel shows the tonic component, $s_{obs}$ (black) and the estimated tonic component, $s_{est}$ (red). Estimates are shown with 95% confidence intervals (CI) (red shaded area). Estimated parameters are reported in the title of each panel.

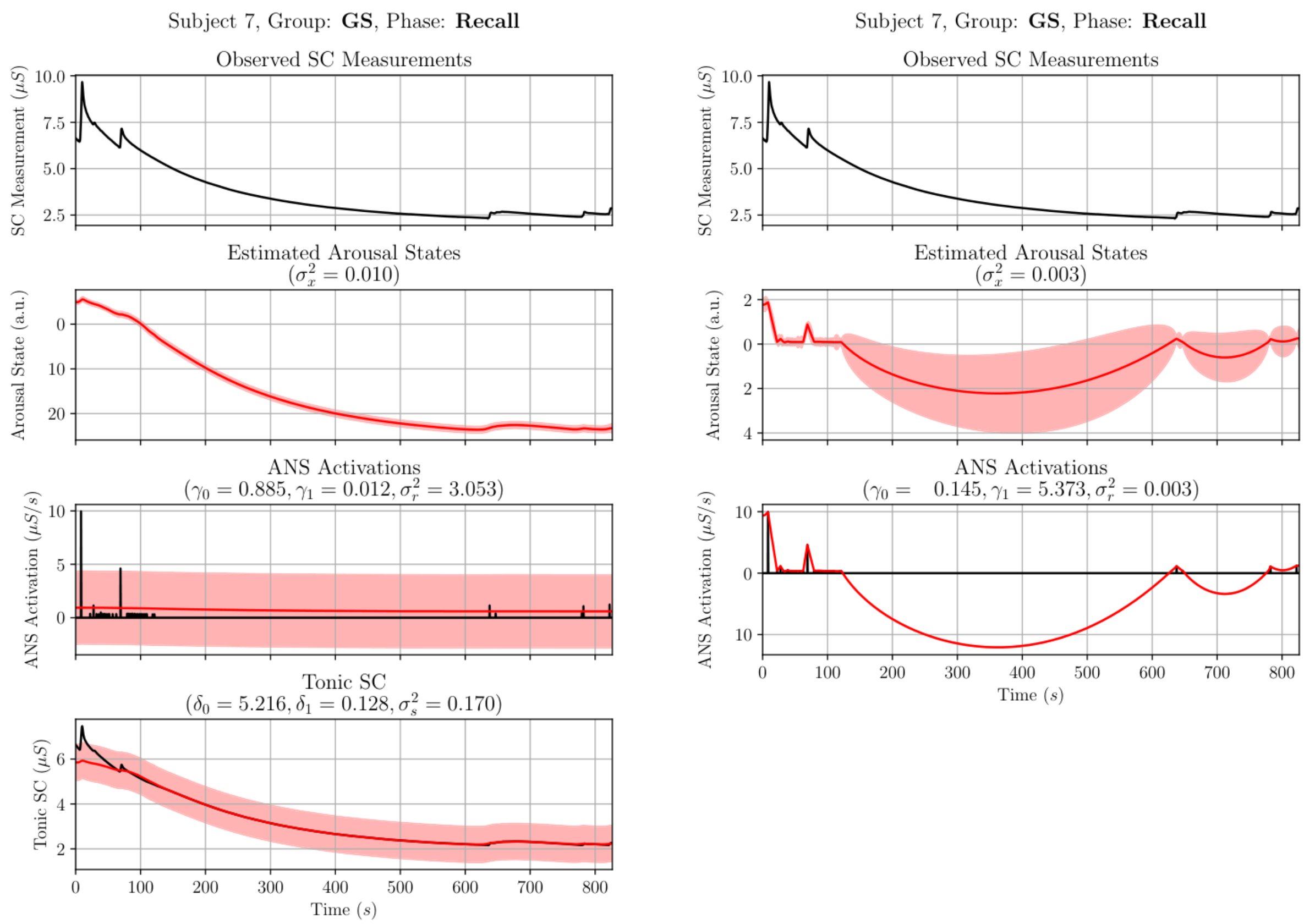


Figure N: **Subject 7 estimated arousal states during the recall phase.** Shown for the MPP+Cont model (left) and the MPP model (right). The first panels show the observed SC measurements, $y$ (black). The second panels show the estimated arousal states, $x_{est}$ (red). The third panels show the ANS activations, $r_{obs}$ (black) and the estimated amplitudes, $r_{est}$ (red). The fourth panel shows the tonic component, $s_{obs}$ (black) and the estimated tonic component, $s_{est}$ (red). Estimates are shown with 95% confidence intervals (CI) (red shaded area). Estimated parameters are reported in the title of each panel.

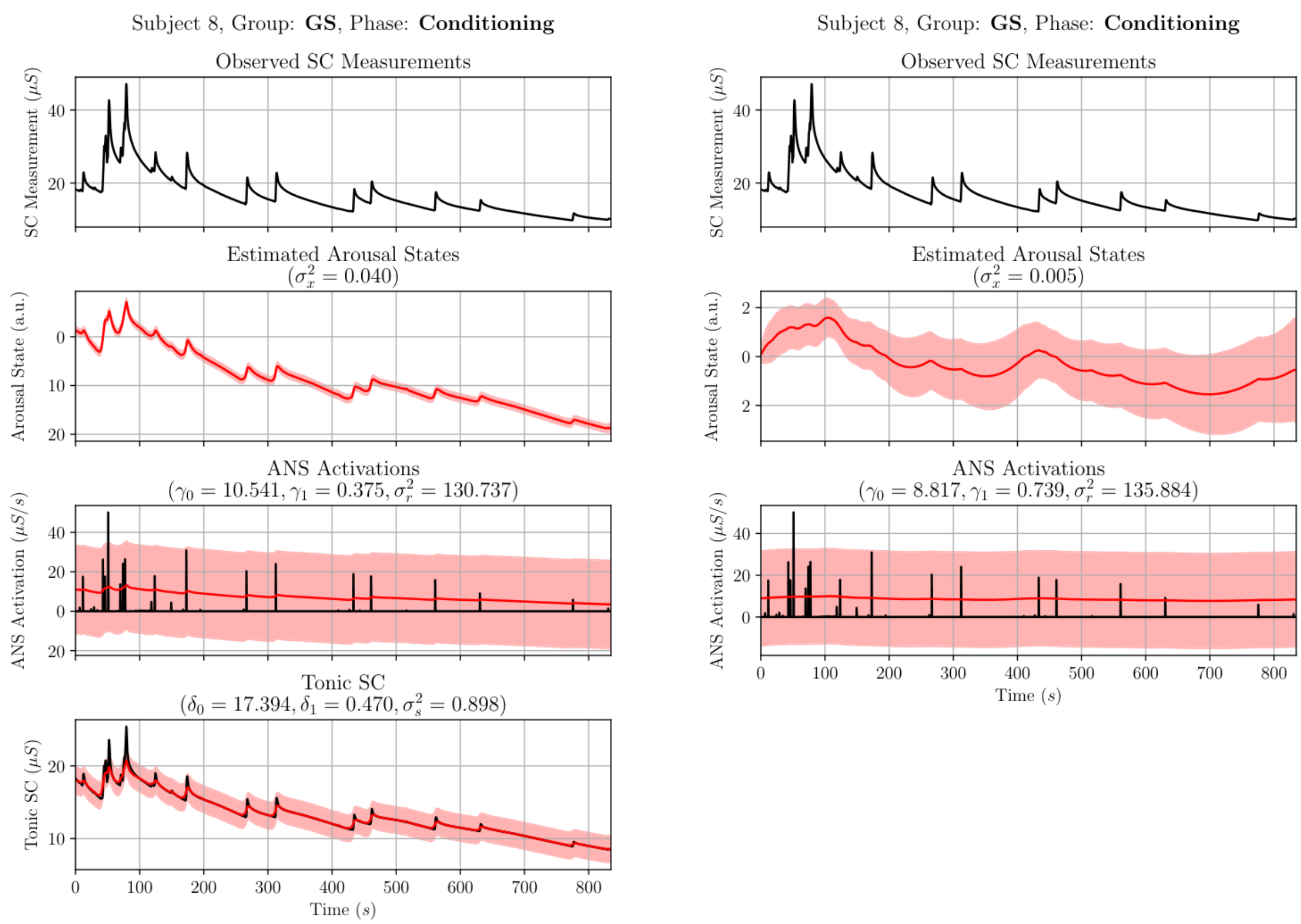


Figure O: **Subject 8 estimated arousal states during the conditioning phase.** Shown for the MPP+Cont model (left) and the MPP model (right). The first panels show the observed SC measurements, $y$ (black). The second panels show the estimated arousal states, $x_{est}$ (red). The third panels show the ANS activations, $r_{obs}$ (black) and the estimated amplitudes, $r_{est}$ (red). The fourth panel shows the tonic component, $s_{obs}$ (black) and the estimated tonic component, $s_{est}$ (red). Estimates are shown with 95% confidence intervals (CI) (red shaded area). Estimated parameters are reported in the title of each panel.

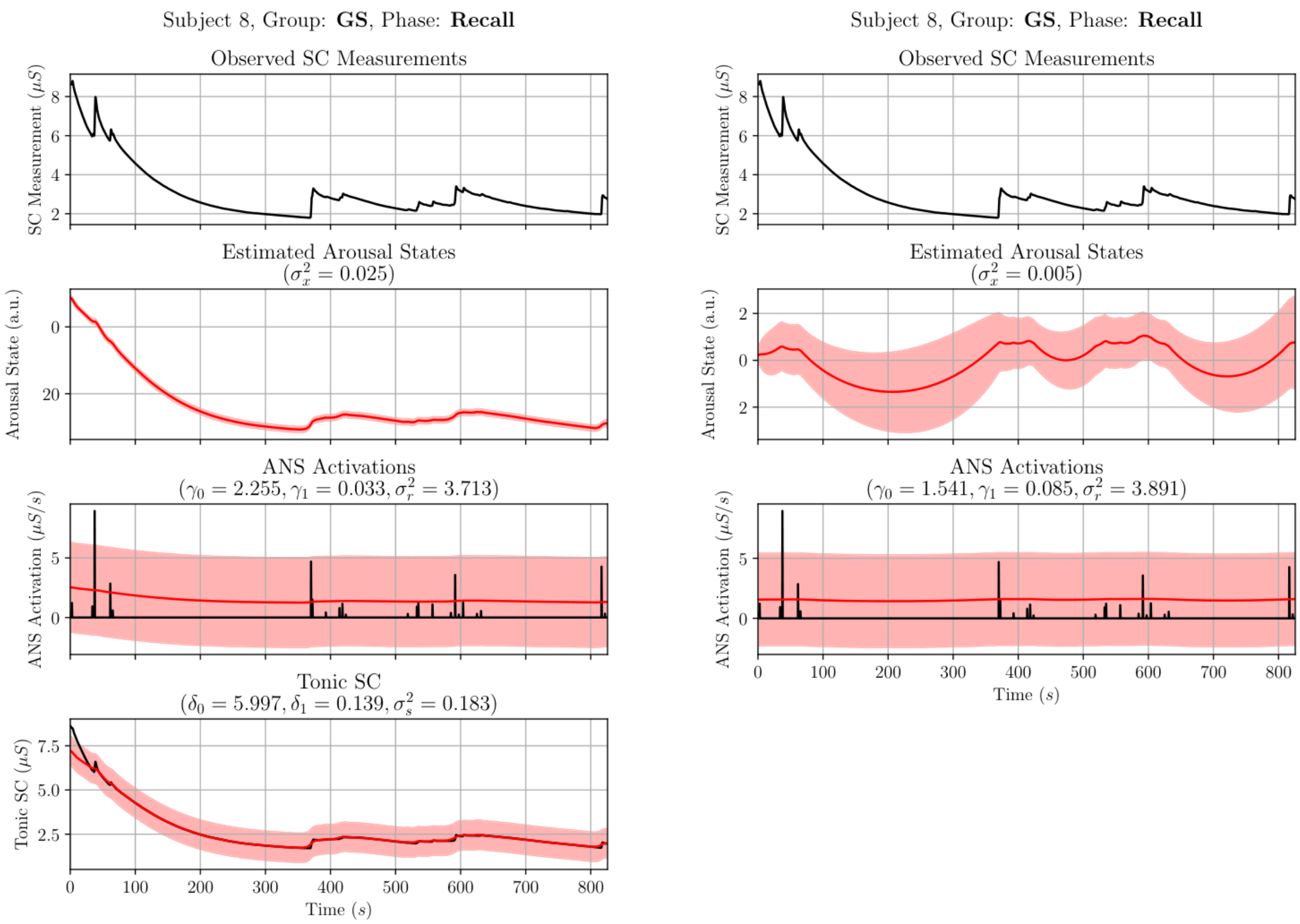


Figure P: **Subject 8 estimated arousal states during the recall phase.** Shown for the MPP+Cont model (left) and the MPP model (right). The first panels show the observed SC measurements, $y$ (black). The second panels show the estimated arousal states, $x_{est}$ (red). The third panels show the ANS activations, $r_{obs}$ (black) and the estimated amplitudes, $r_{est}$ (red). The fourth panel shows the tonic component, $s_{obs}$ (black) and the estimated tonic component, $s_{est}$ (red). Estimates are shown with 95% confidence intervals (CI) (red shaded area). Estimated parameters are reported in the title of each panel.

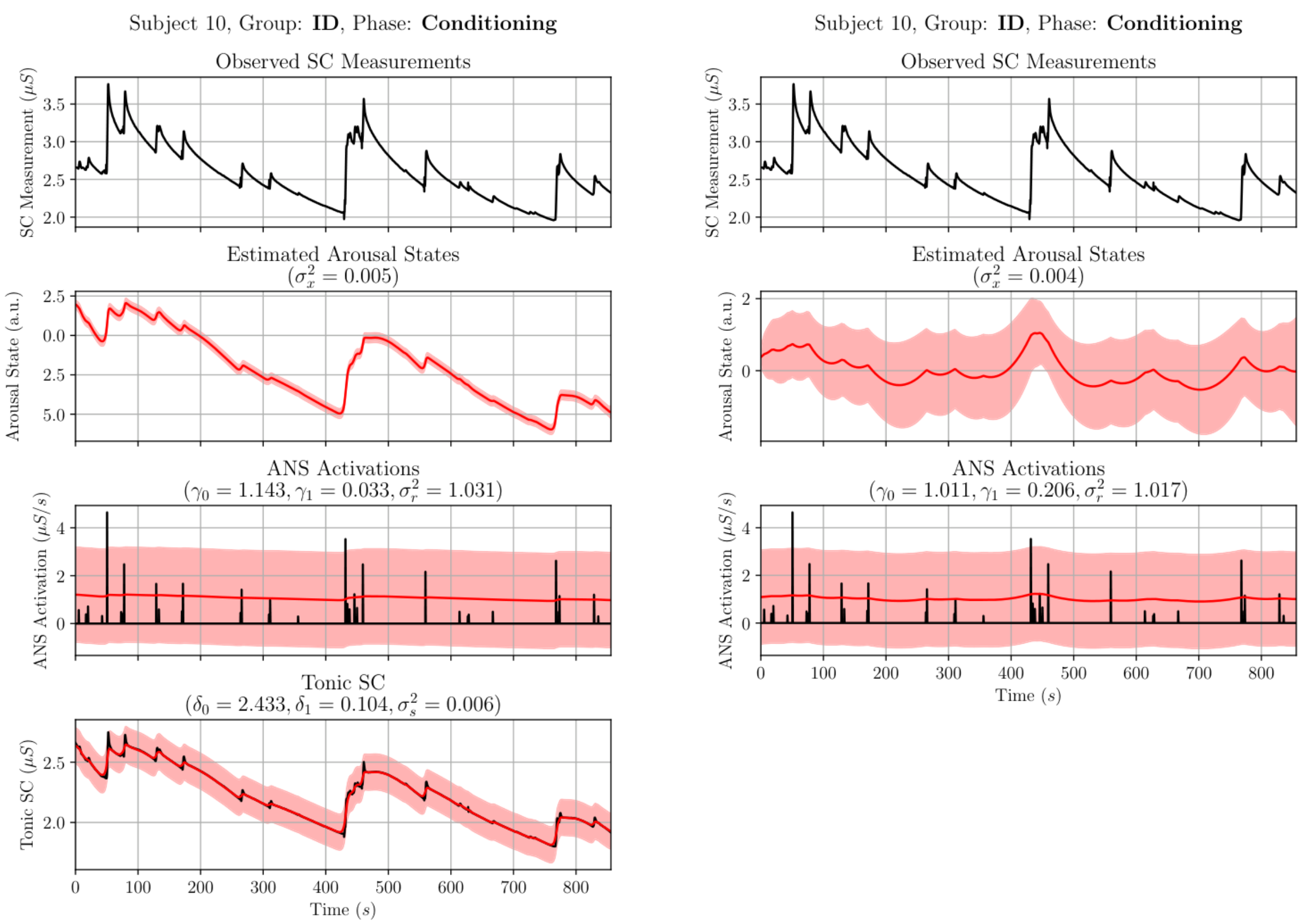


Figure Q: **Subject 10 estimated arousal states during the conditioning phase.** Shown for the MPP+Cont model (left) and the MPP model (right). The first panels show the observed SC measurements, $y$ (black). The second panels show the estimated arousal states, $x_{est}$ (red). The third panels show the ANS activations, $r_{obs}$ (black) and the estimated amplitudes, $r_{est}$ (red). The fourth panel shows the tonic component, $s_{obs}$ (black) and the estimated tonic component, $s_{est}$ (red). Estimates are shown with 95% confidence intervals (CI) (red shaded area). Estimated parameters are reported in the title of each panel.

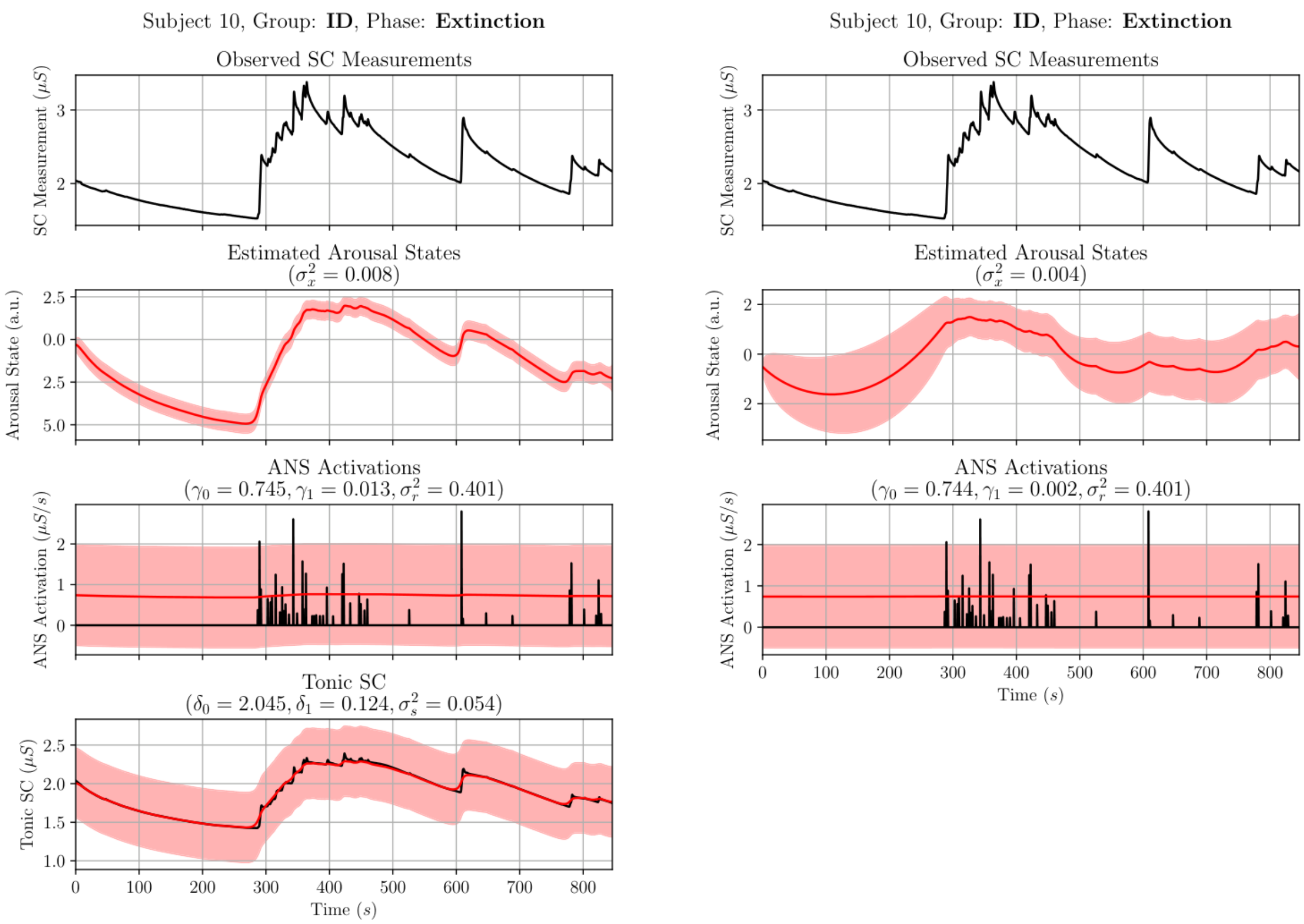


Figure R: **Subject 10 estimated arousal states during the extinction phase.** Shown for the MPP+Cont model (left) and the MPP model (right). The first panels show the observed SC measurements, $y$ (black). The second panels show the estimated arousal states, $x_{est}$ (red). The third panels show the ANS activations, $r_{obs}$ (black) and the estimated amplitudes, $r_{est}$ (red). The fourth panel shows the tonic component, $s_{obs}$ (black) and the estimated tonic component, $s_{est}$ (red). Estimates are shown with 95% confidence intervals (CI) (red shaded area). Estimated parameters are reported in the title of each panel.

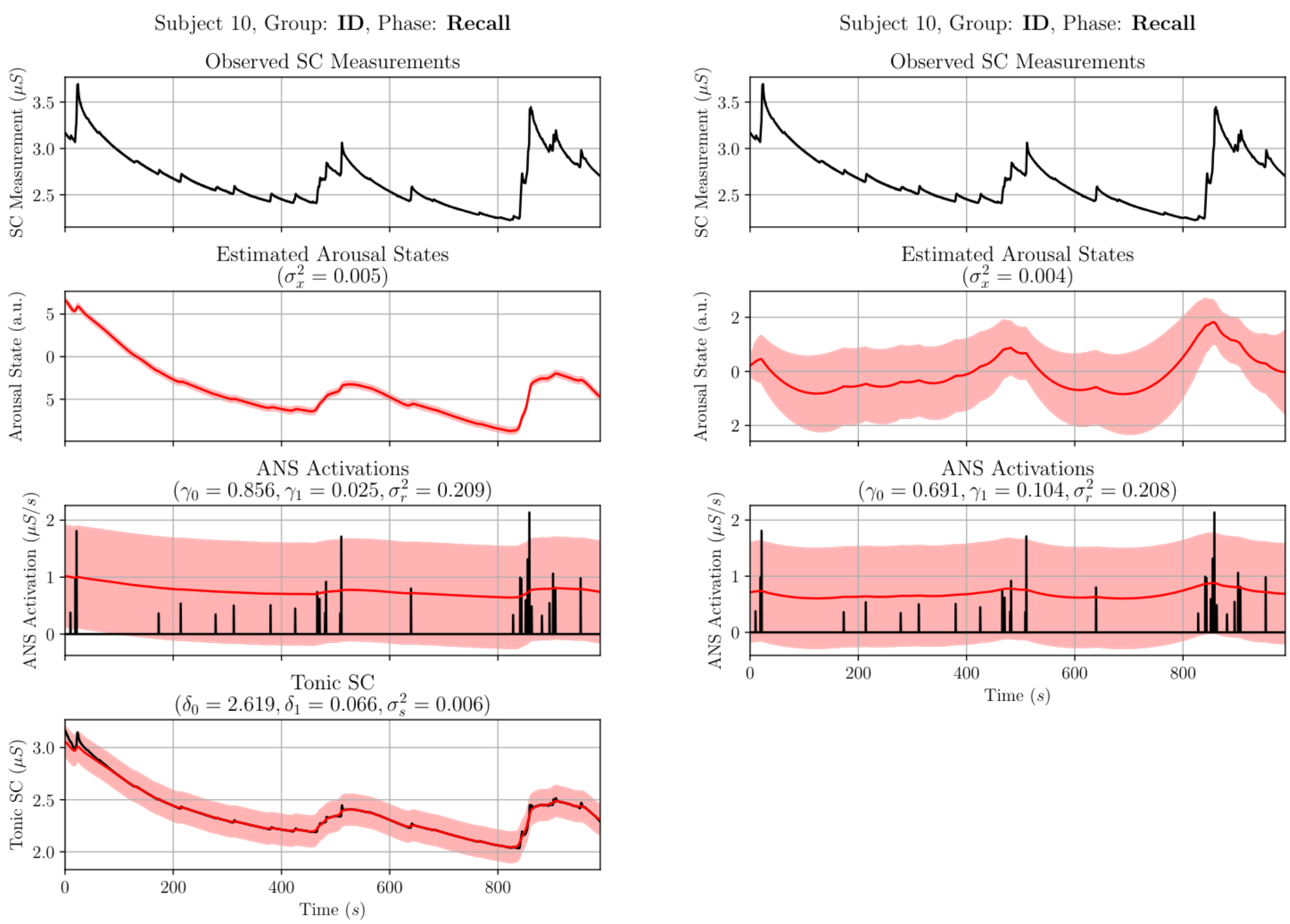


Figure S: **Subject 10 estimated arousal states during the recall phase.** Shown for the MPP+Cont model (left) and the MPP model (right). The first panels show the observed SC measurements, $y$ (black). The second panels show the estimated arousal states, $x_{est}$ (red). The third panels show the ANS activations, $r_{obs}$ (black) and the estimated amplitudes, $r_{est}$ (red). The fourth panel shows the tonic component, $s_{obs}$ (black) and the estimated tonic component, $s_{est}$ (red). Estimates are shown with 95% confidence intervals (CI) (red shaded area). Estimated parameters are reported in the title of each panel.

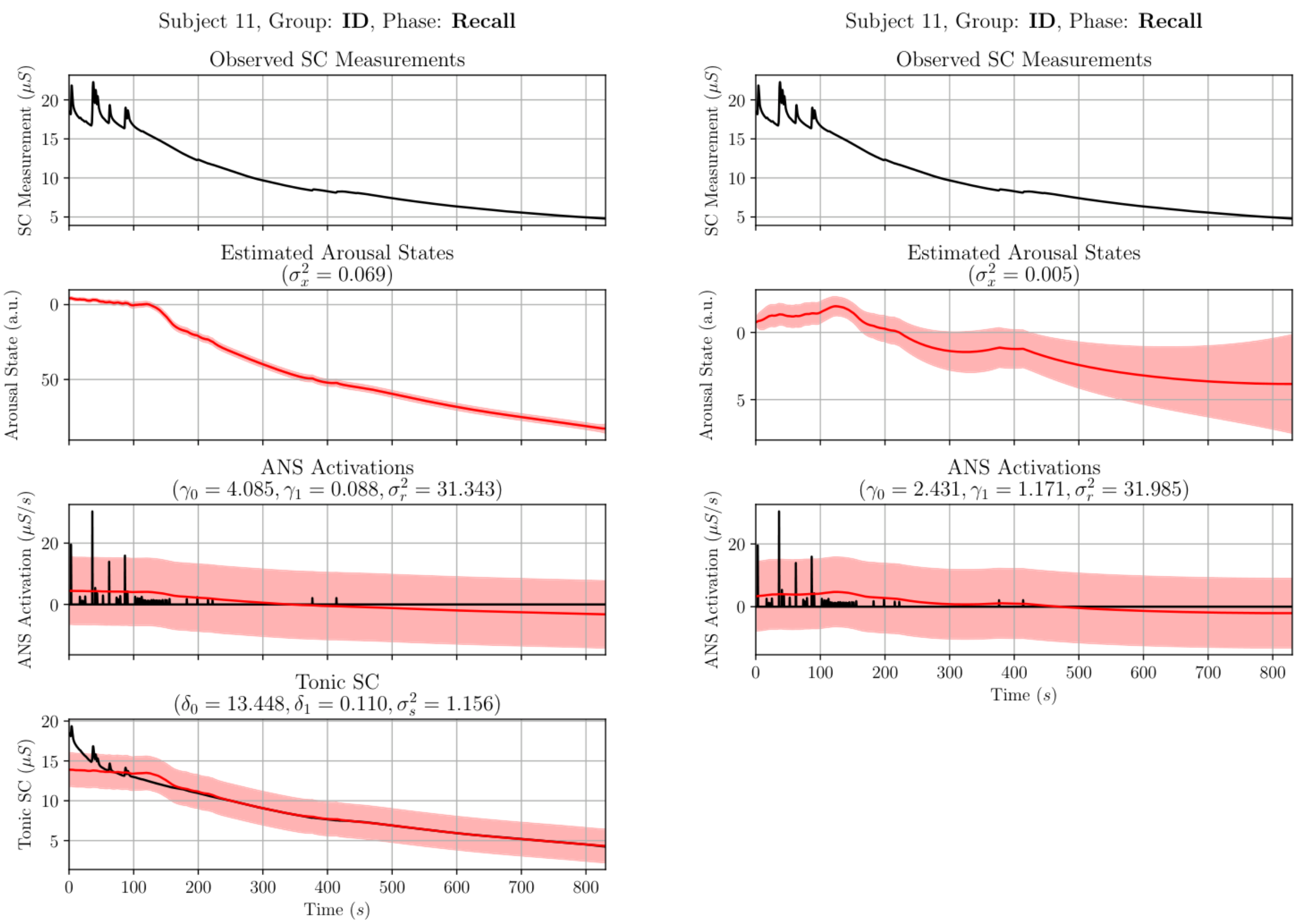


Figure T: **Subject 11 estimated arousal states during the recall phase.** Shown for the MPP+Cont model (left) and the MPP model (right). The first panels show the observed SC measurements, $y$ (black). The second panels show the estimated arousal states, $x_{est}$ (red). The third panels show the ANS activations, $r_{obs}$ (black) and the estimated amplitudes, $r_{est}$ (red). The fourth panel shows the tonic component, $s_{obs}$ (black) and the estimated tonic component, $s_{est}$ (red). Estimates are shown with 95% confidence intervals (CI) (red shaded area). Estimated parameters are reported in the title of each panel.

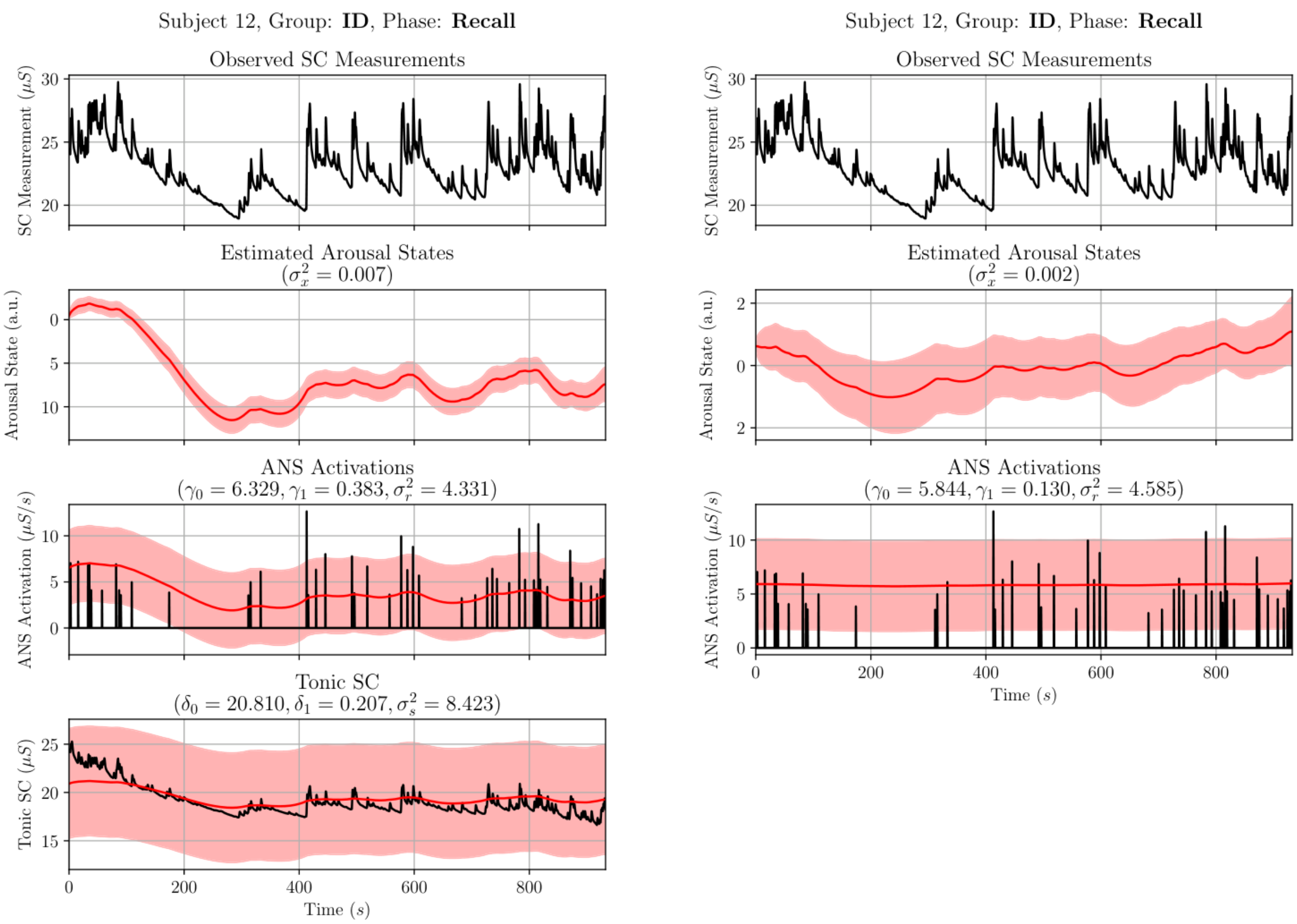


Figure U: **Subject 12 estimated arousal states during the recall phase.** Shown for the MPP+Cont model (left) and the MPP model (right). The first panels show the observed SC measurements, $y$ (black). The second panels show the estimated arousal states, $x_{est}$ (red). The third panels show the ANS activations, $r_{obs}$ (black) and the estimated amplitudes, $r_{est}$ (red). The fourth panel shows the tonic component, $s_{obs}$ (black) and the estimated tonic component, $s_{est}$ (red). Estimates are shown with 95% confidence intervals (CI) (red shaded area). Estimated parameters are reported in the title of each panel.

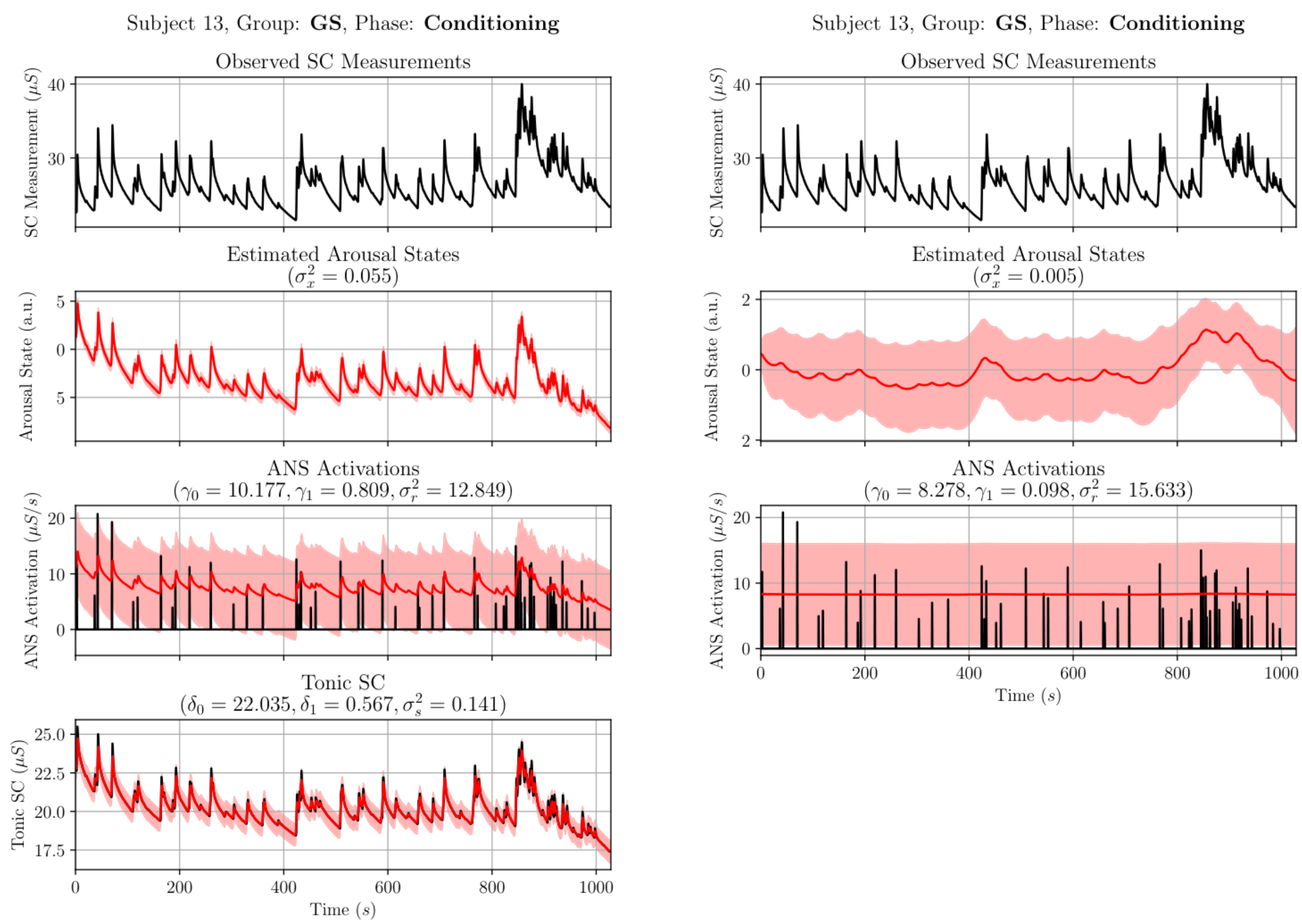


Figure V: **Subject 13 estimated arousal states during the conditioning phase.** Shown for the MPP+Cont model (left) and the MPP model (right). The first panels show the observed SC measurements, $y$ (black). The second panels show the estimated arousal states, $x_{est}$ (red). The third panels show the ANS activations, $r_{obs}$ (black) and the estimated amplitudes, $r_{est}$ (red). The fourth panel shows the tonic component, $s_{obs}$ (black) and the estimated tonic component, $s_{est}$ (red). Estimates are shown with 95% confidence intervals (CI) (red shaded area). Estimated parameters are reported in the title of each panel.

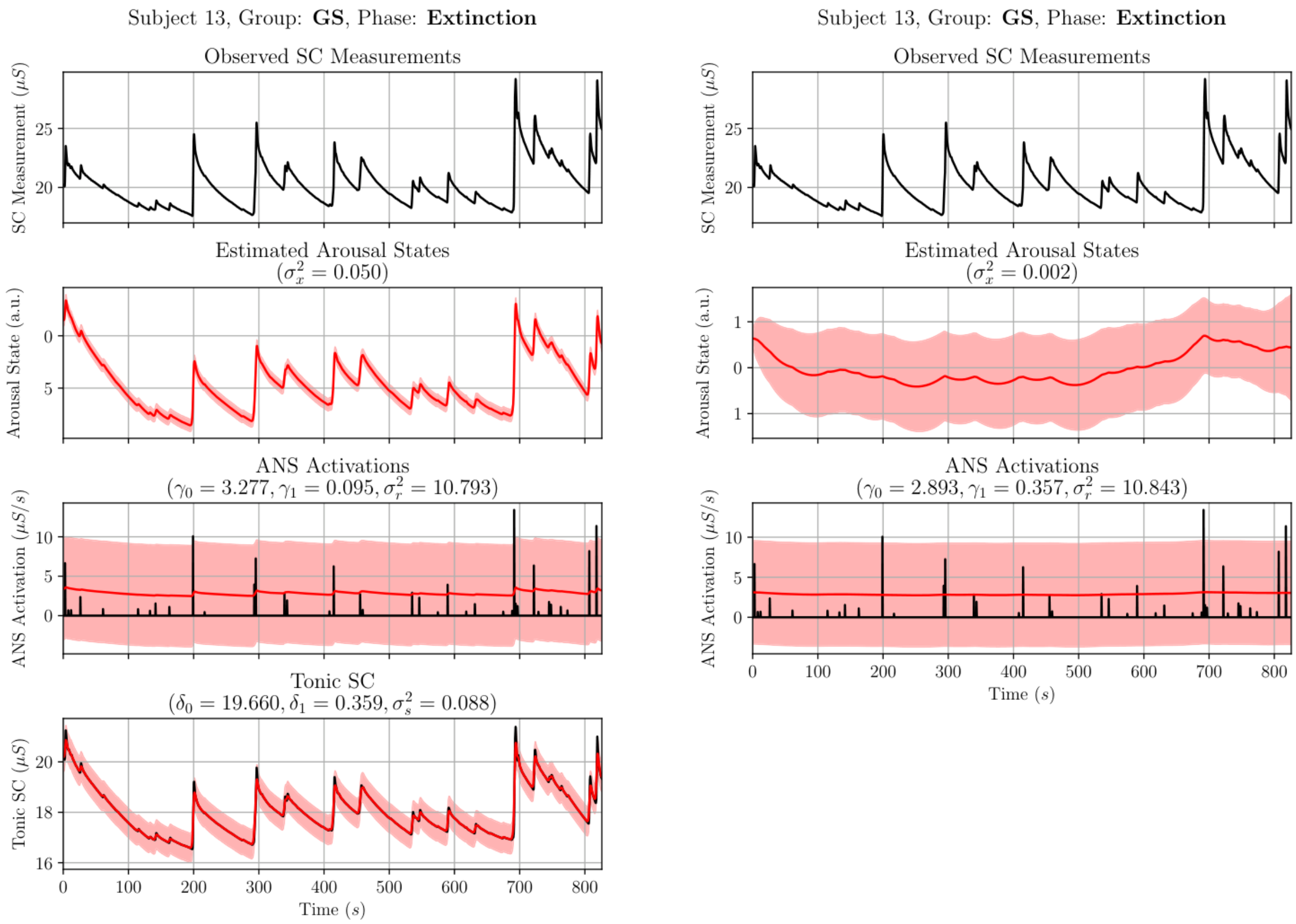


Figure W: **Subject 13 estimated arousal states during the extinction phase.** Shown for the MPP+Cont model (left) and the MPP model (right). The first panels show the observed SC measurements, $y$ (black). The second panels show the estimated arousal states, $x_{est}$ (red). The third panels show the ANS activations, $r_{obs}$ (black) and the estimated amplitudes, $r_{est}$ (red). The fourth panel shows the tonic component, $s_{obs}$ (black) and the estimated tonic component, $s_{est}$ (red). Estimates are shown with 95% confidence intervals (CI) (red shaded area). Estimated parameters are reported in the title of each panel.

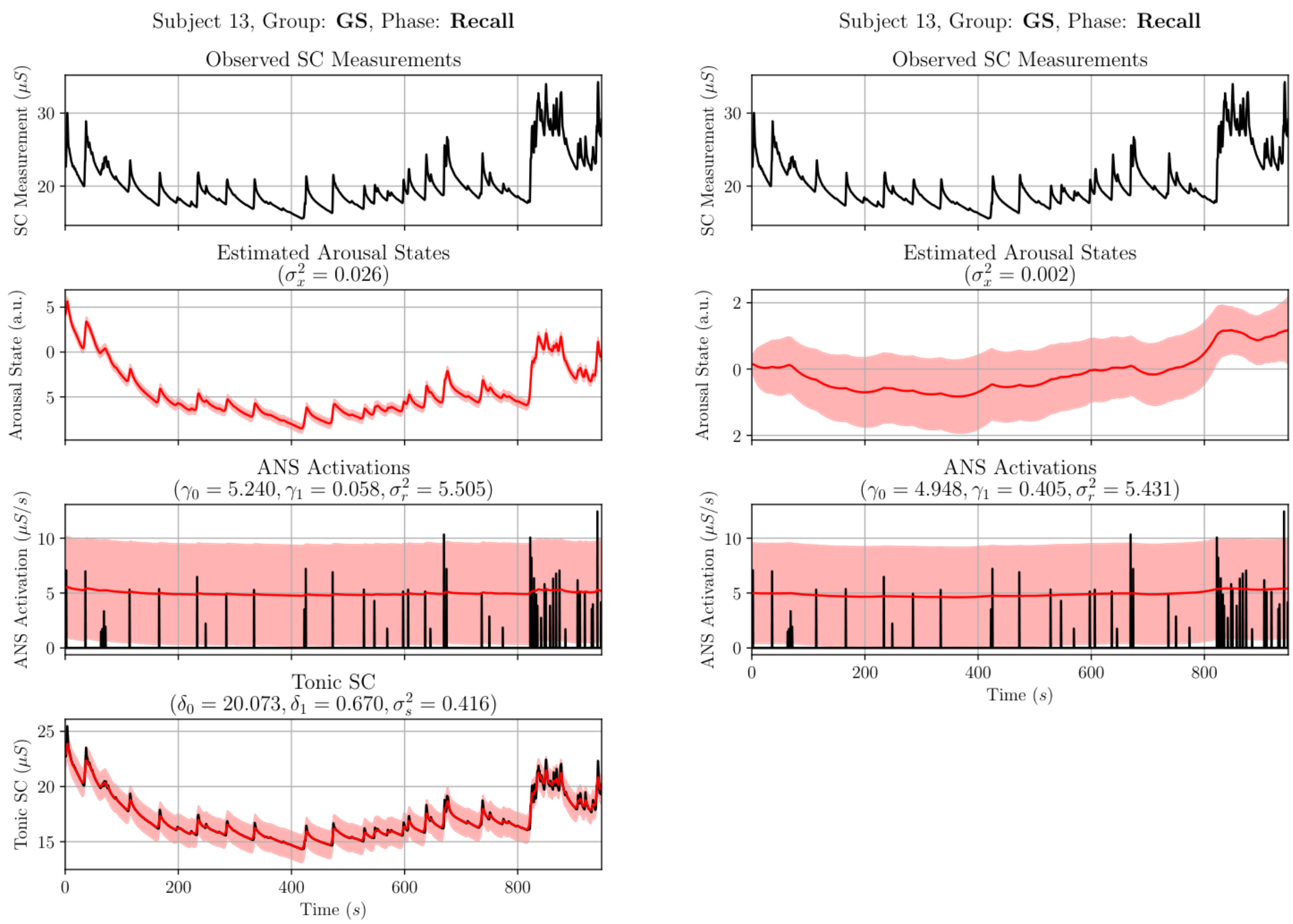


Figure X: **Subject 13 estimated arousal states during the recall phase.** Shown for the MPP+Cont model (left) and the MPP model (right). The first panels show the observed SC measurements, $y$ (black). The second panels show the estimated arousal states, $x_{est}$ (red). The third panels show the ANS activations, $r_{obs}$ (black) and the estimated amplitudes, $r_{est}$ (red). The fourth panel shows the tonic component, $s_{obs}$ (black) and the estimated tonic component, $s_{est}$ (red). Estimates are shown with 95% confidence intervals (CI) (red shaded area). Estimated parameters are reported in the title of each panel.

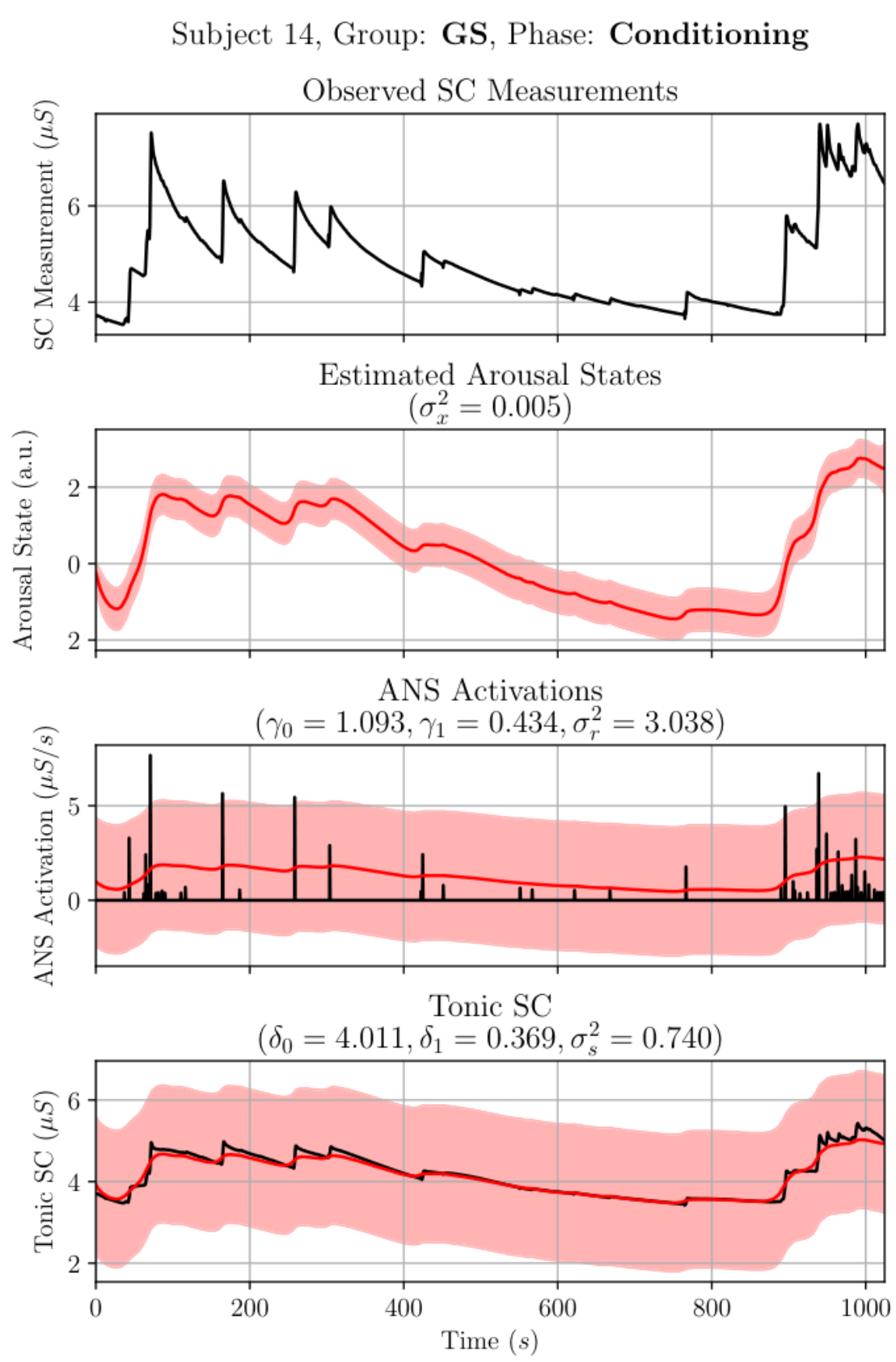


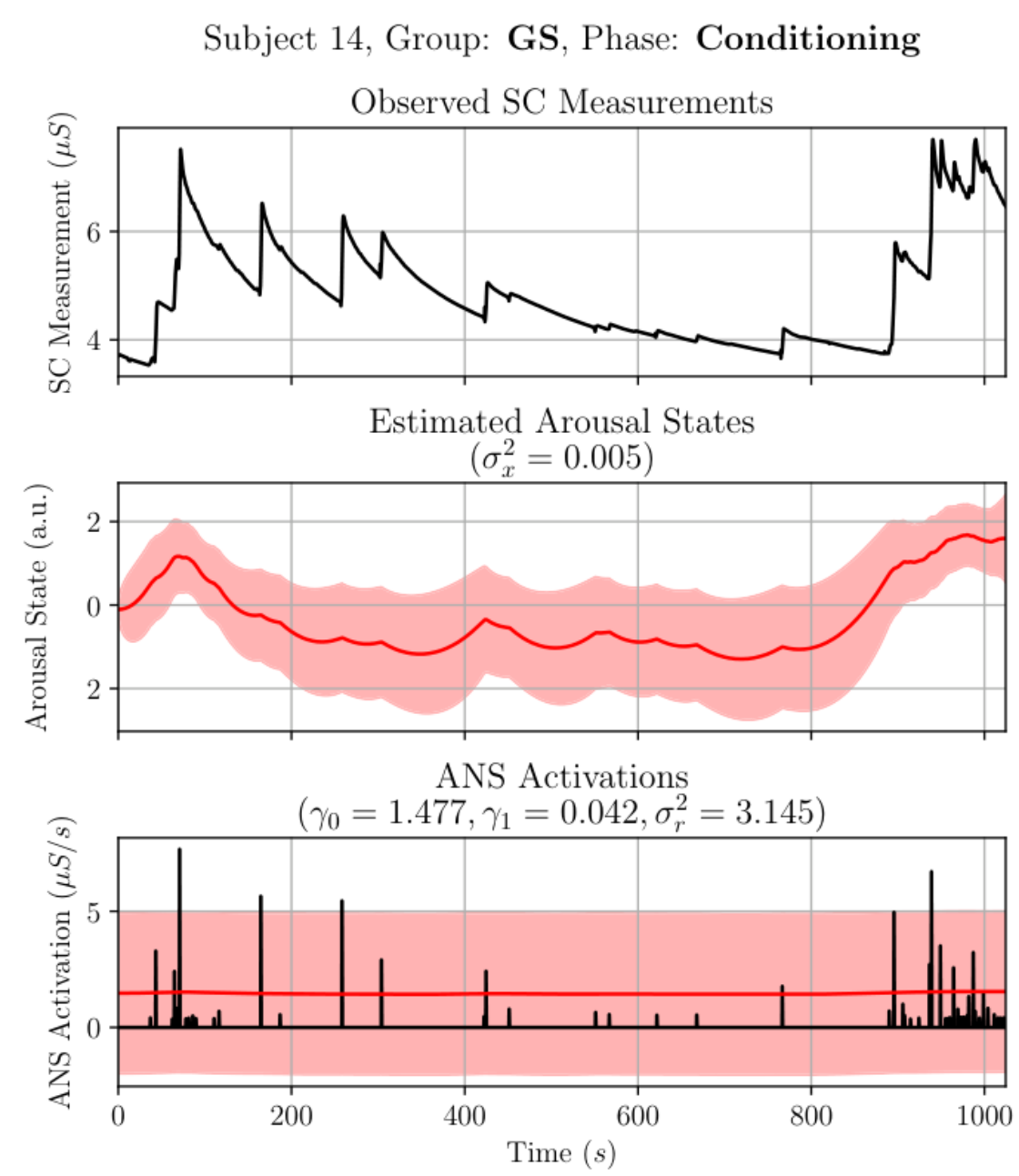


Figure Y: **Subject 14 estimated arousal states during the conditioning phase.** Shown for the MPP+Cont model (left) and the MPP model (right). The first panels show the observed SC measurements, $y$ (black). The second panels show the estimated arousal states, $x_{est}$ (red). The third panels show the ANS activations, $r_{obs}$ (black) and the estimated amplitudes, $r_{est}$ (red). The fourth panel shows the tonic component, $s_{obs}$ (black) and the estimated tonic component, $s_{est}$ (red). Estimates are shown with 95% confidence intervals (CI) (red shaded area). Estimated parameters are reported in the title of each panel.

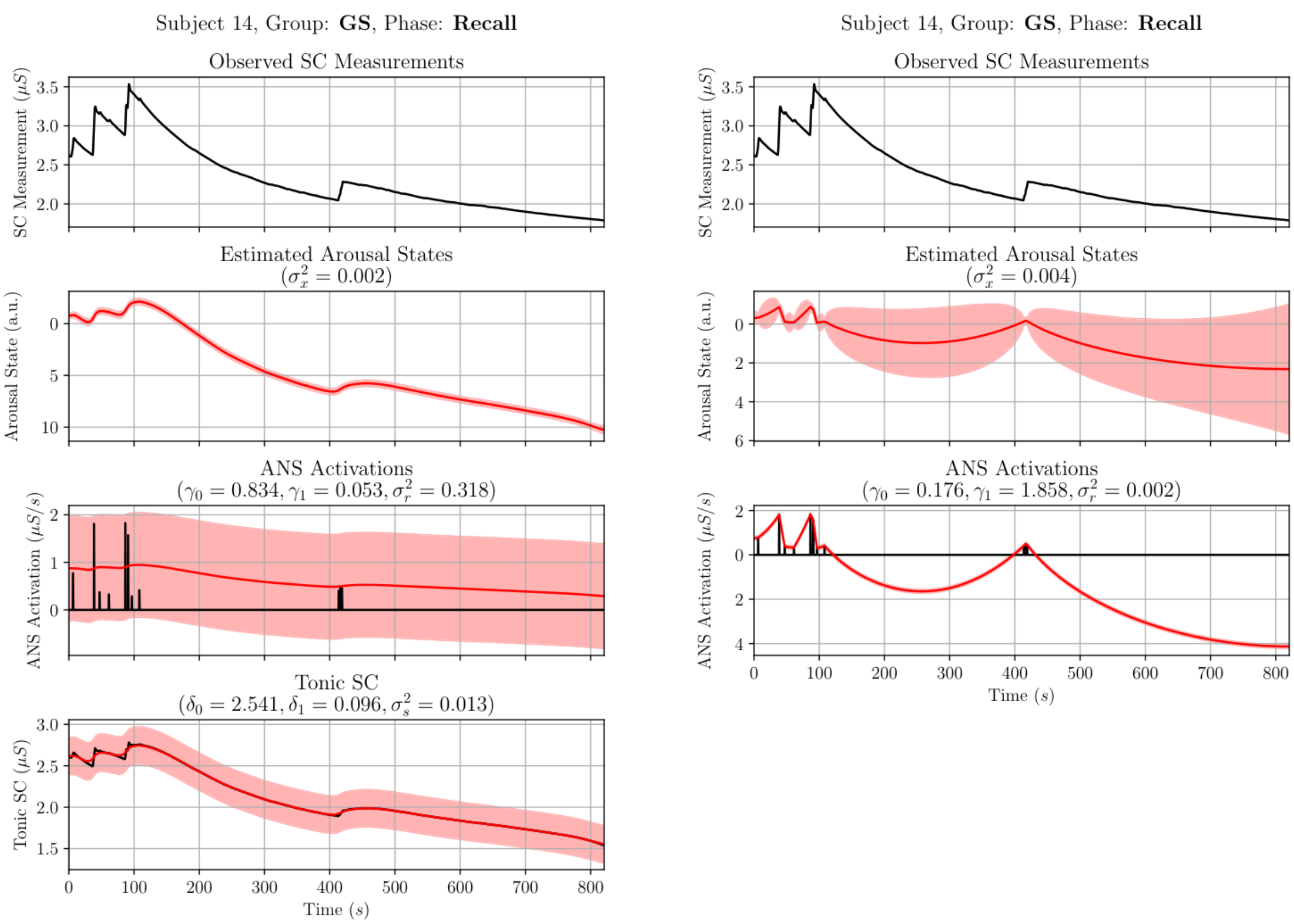


Figure Z: **Subject 14 estimated arousal states during the recall phase.** Shown for the MPP+Cont model (left) and the MPP model (right). The first panels show the observed SC measurements, $y$ (black). The second panels show the estimated arousal states, $x_{est}$ (red). The third panels show the ANS activations, $r_{obs}$ (black) and the estimated amplitudes, $r_{est}$ (red). The fourth panel shows the tonic component, $s_{obs}$ (black) and the estimated tonic component, $s_{est}$ (red). Estimates are shown with 95% confidence intervals (CI) (red shaded area). Estimated parameters are reported in the title of each panel.

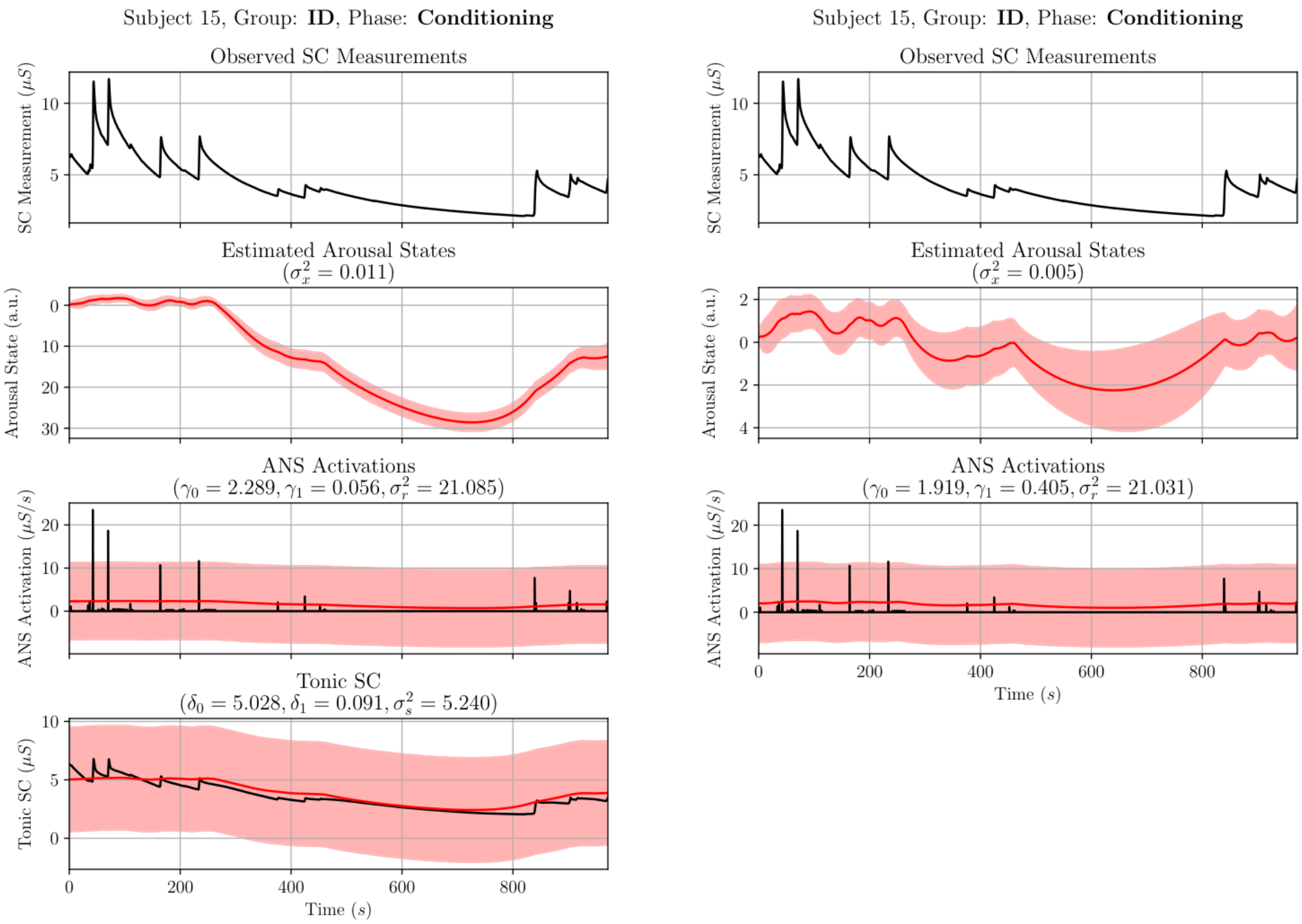


Figure AA: **Subject 15 estimated arousal states during the conditioning phase.** Shown for the MPP+Cont model (left) and the MPP model (right). The first panels show the observed SC measurements, $y$ (black). The second panels show the estimated arousal states, $x_{est}$ (red). The third panels show the ANS activations, $r_{obs}$ (black) and the estimated amplitudes, $r_{est}$ (red). The fourth panel shows the tonic component, $s_{obs}$ (black) and the estimated tonic component, $s_{est}$ (red). Estimates are shown with 95% confidence intervals (CI) (red shaded area). Estimated parameters are reported in the title of each panel.

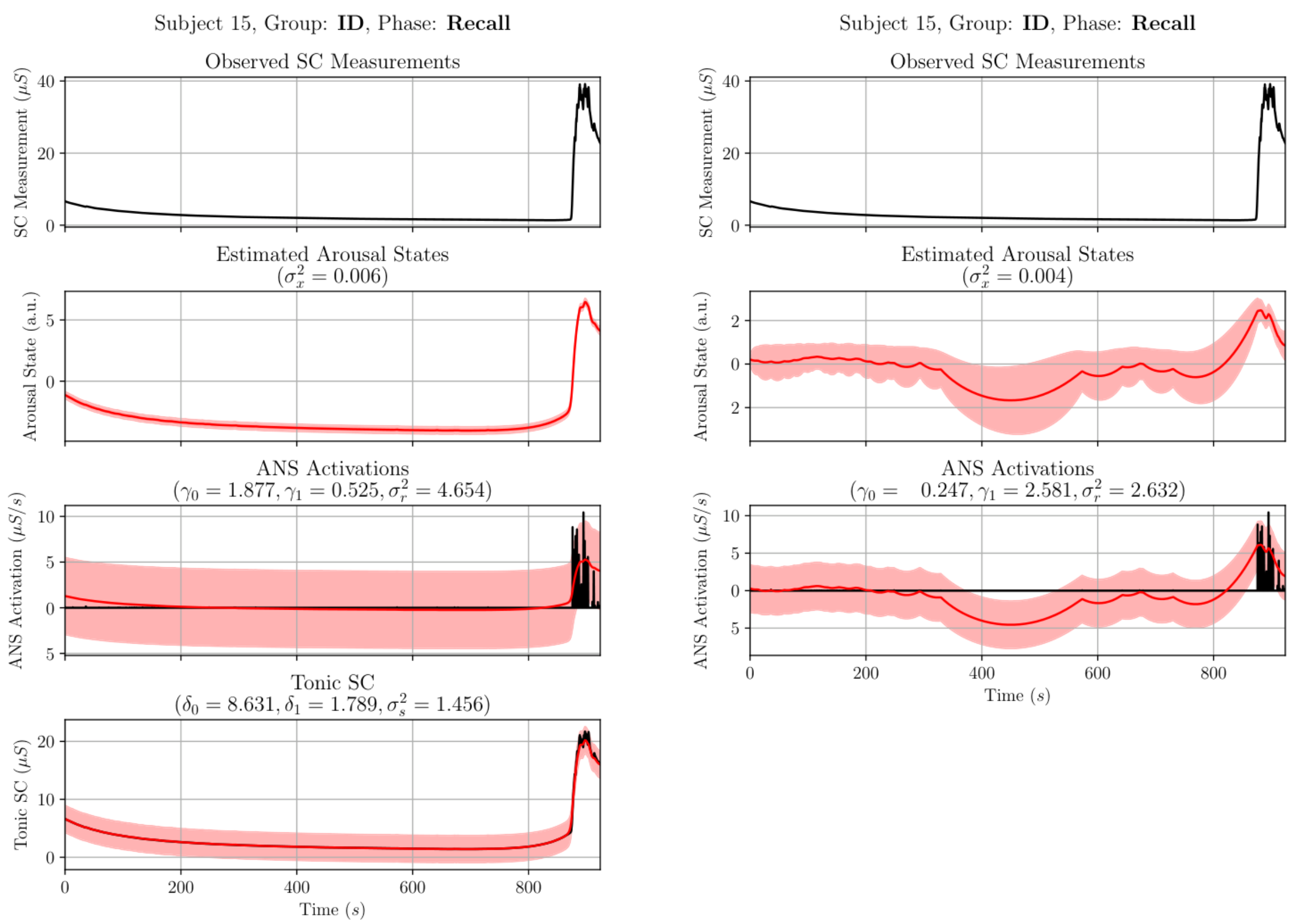


Figure AB: **Subject 15 estimated arousal states during the recall phase.** Shown for the MPP+Cont model (left) and the MPP model (right). The first panels show the observed SC measurements, $y$ (black). The second panels show the estimated arousal states, $x_{est}$ (red). The third panels show the ANS activations, $r_{obs}$ (black) and the estimated amplitudes, $r_{est}$ (red). The fourth panel shows the tonic component, $s_{obs}$ (black) and the estimated tonic component, $s_{est}$ (red). Estimates are shown with 95% confidence intervals (CI) (red shaded area). Estimated parameters are reported in the title of each panel.

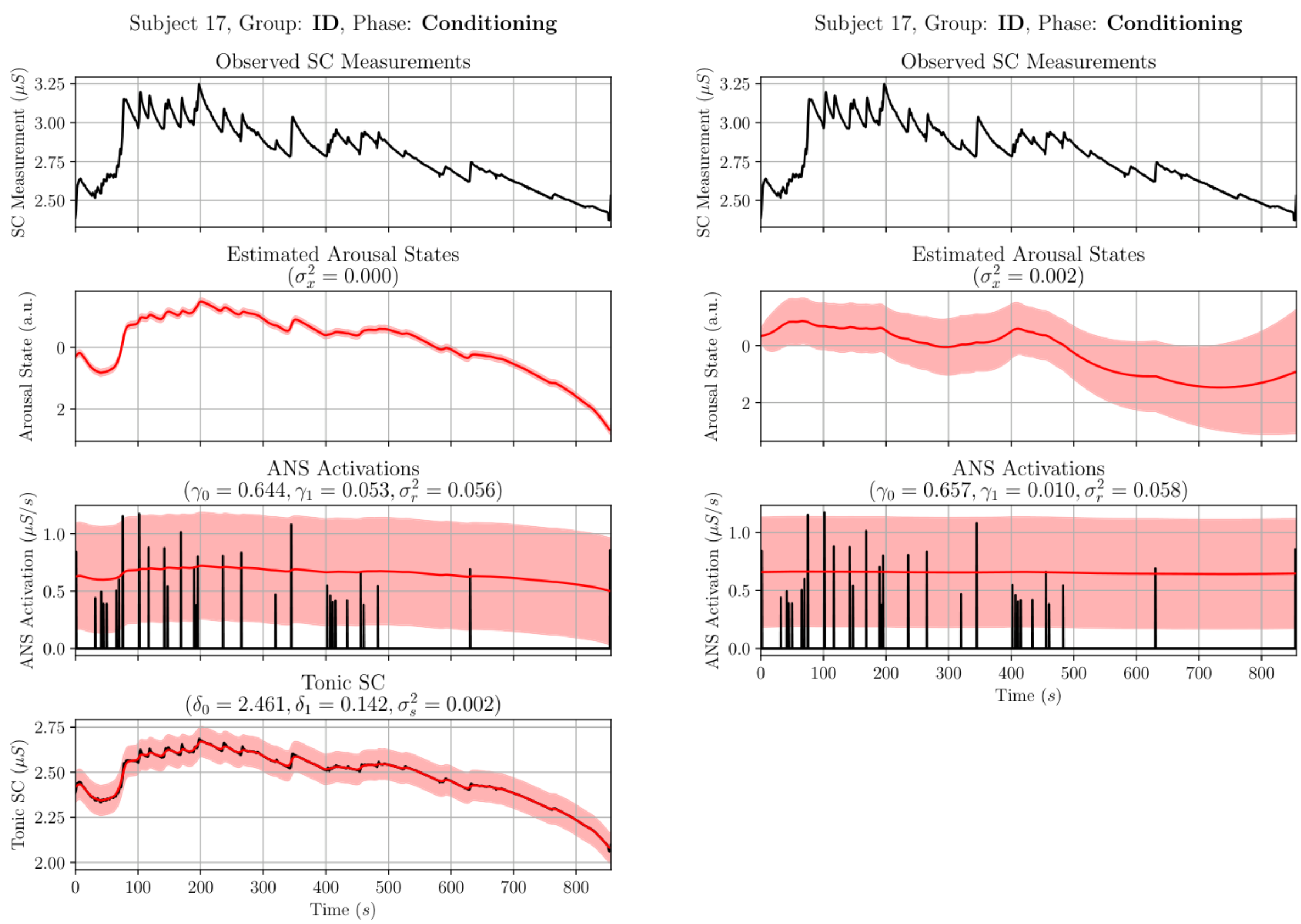


Figure AC: **Subject 17 estimated arousal states during the conditioning phase.** Shown for the MPP+Cont model (left) and the MPP model (right). The first panels show the observed SC measurements, $y$ (black). The second panels show the estimated arousal states, $x_{est}$ (red). The third panels show the ANS activations, $r_{obs}$ (black) and the estimated amplitudes, $r_{est}$ (red). The fourth panel shows the tonic component, $s_{obs}$ (black) and the estimated tonic component, $s_{est}$ (red). Estimates are shown with 95% confidence intervals (CI) (red shaded area). Estimated parameters are reported in the title of each panel.

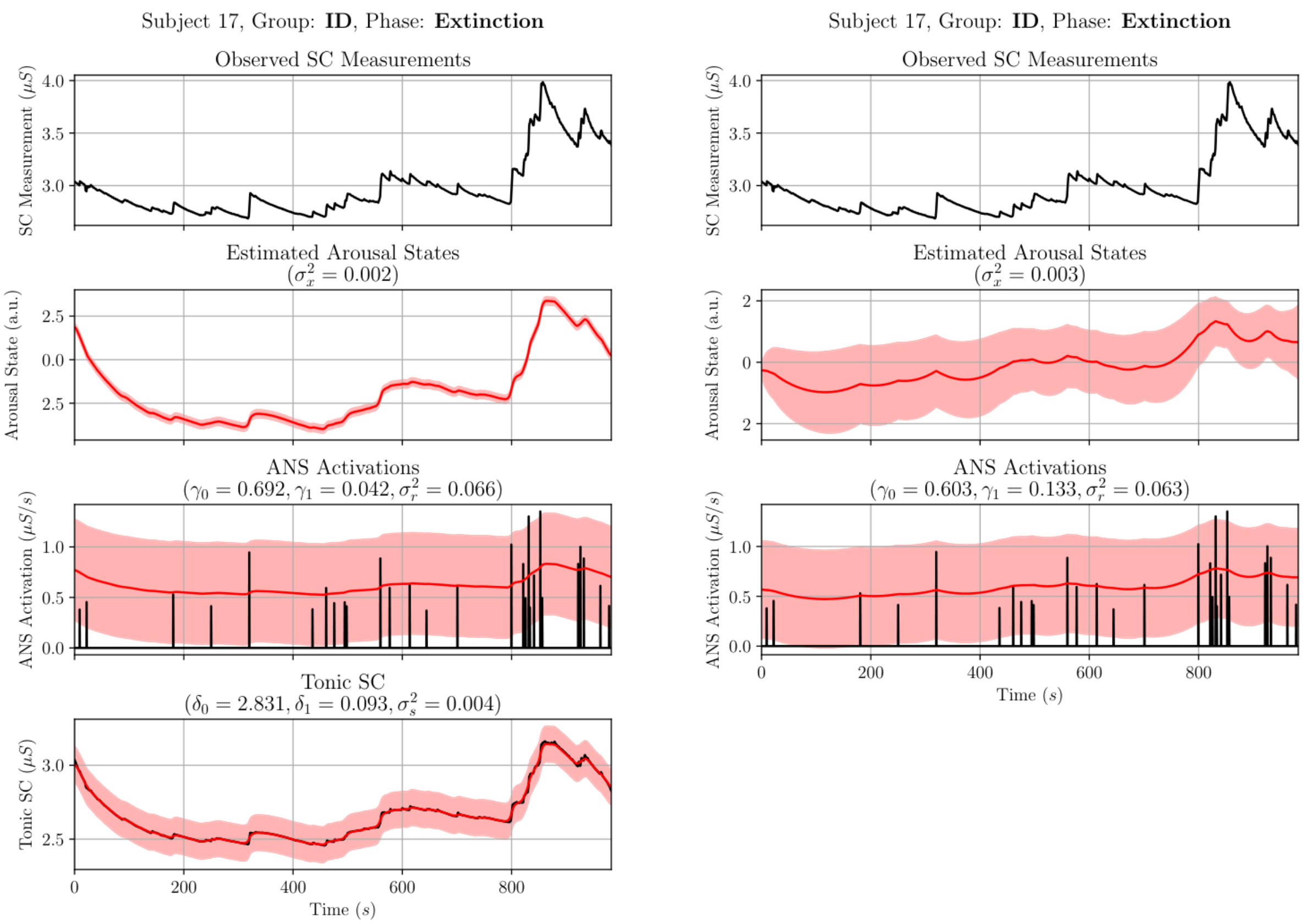


Figure AD: **Subject 17 estimated arousal states during the extinction phase.** Shown for the MPP+Cont model (left) and the MPP model (right). The first panels show the observed SC measurements, $y$ (black). The second panels show the estimated arousal states, $x_{est}$ (red). The third panels show the ANS activations, $r_{obs}$ (black) and the estimated amplitudes, $r_{est}$ (red). The fourth panel shows the tonic component, $s_{obs}$ (black) and the estimated tonic component, $s_{est}$ (red). Estimates are shown with 95% confidence intervals (CI) (red shaded area). Estimated parameters are reported in the title of each panel.

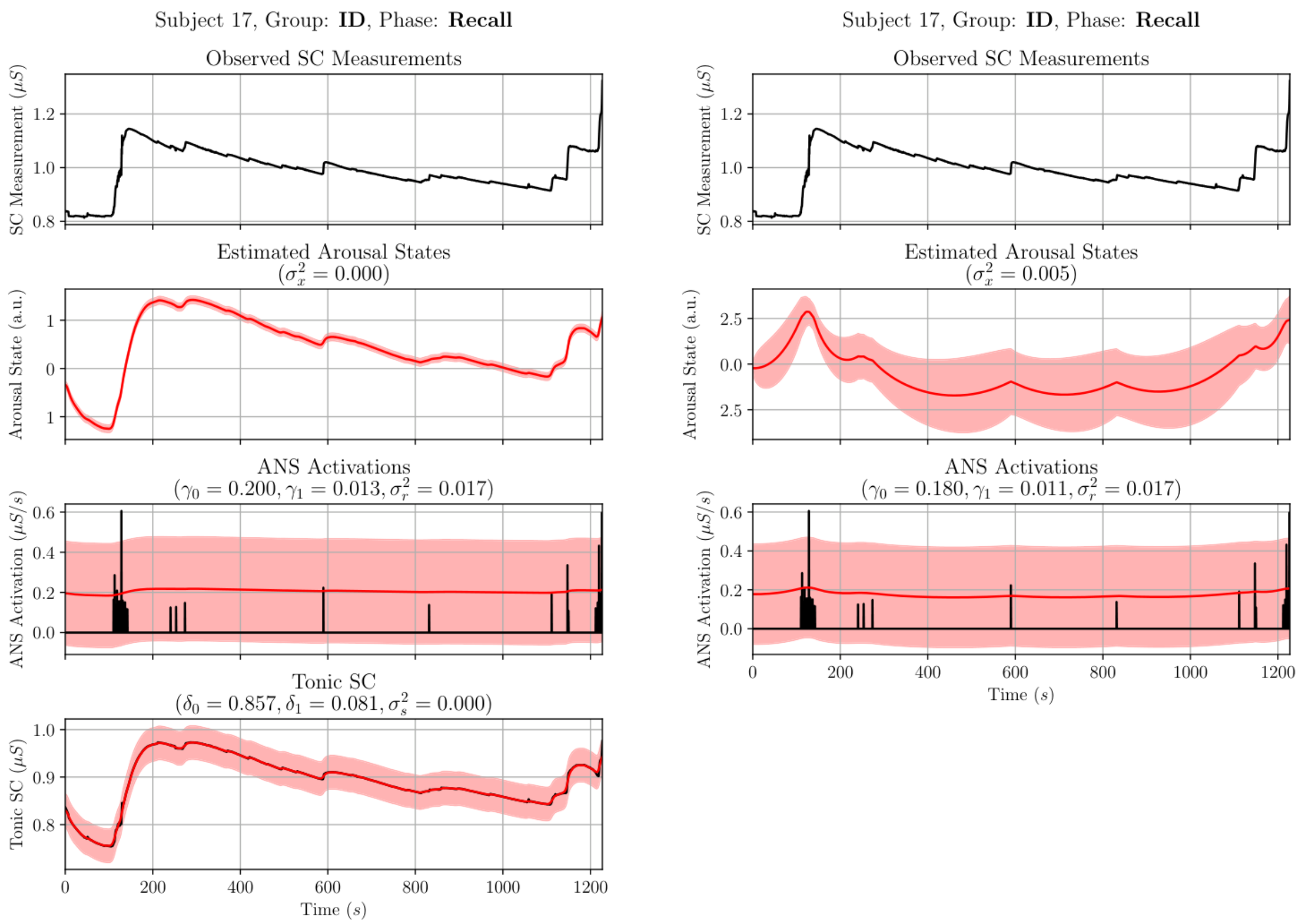


Figure AE: **Subject 17 estimated arousal states during the recall phase.** Shown for the MPP+Cont model (left) and the MPP model (right). The first panels show the observed SC measurements, $y$ (black). The second panels show the estimated arousal states, $x_{est}$ (red). The third panels show the ANS activations, $r_{obs}$ (black) and the estimated amplitudes, $r_{est}$ (red). The fourth panel shows the tonic component, $s_{obs}$ (black) and the estimated tonic component, $s_{est}$ (red). Estimates are shown with 95% confidence intervals (CI) (red shaded area). Estimated parameters are reported in the title of each panel.

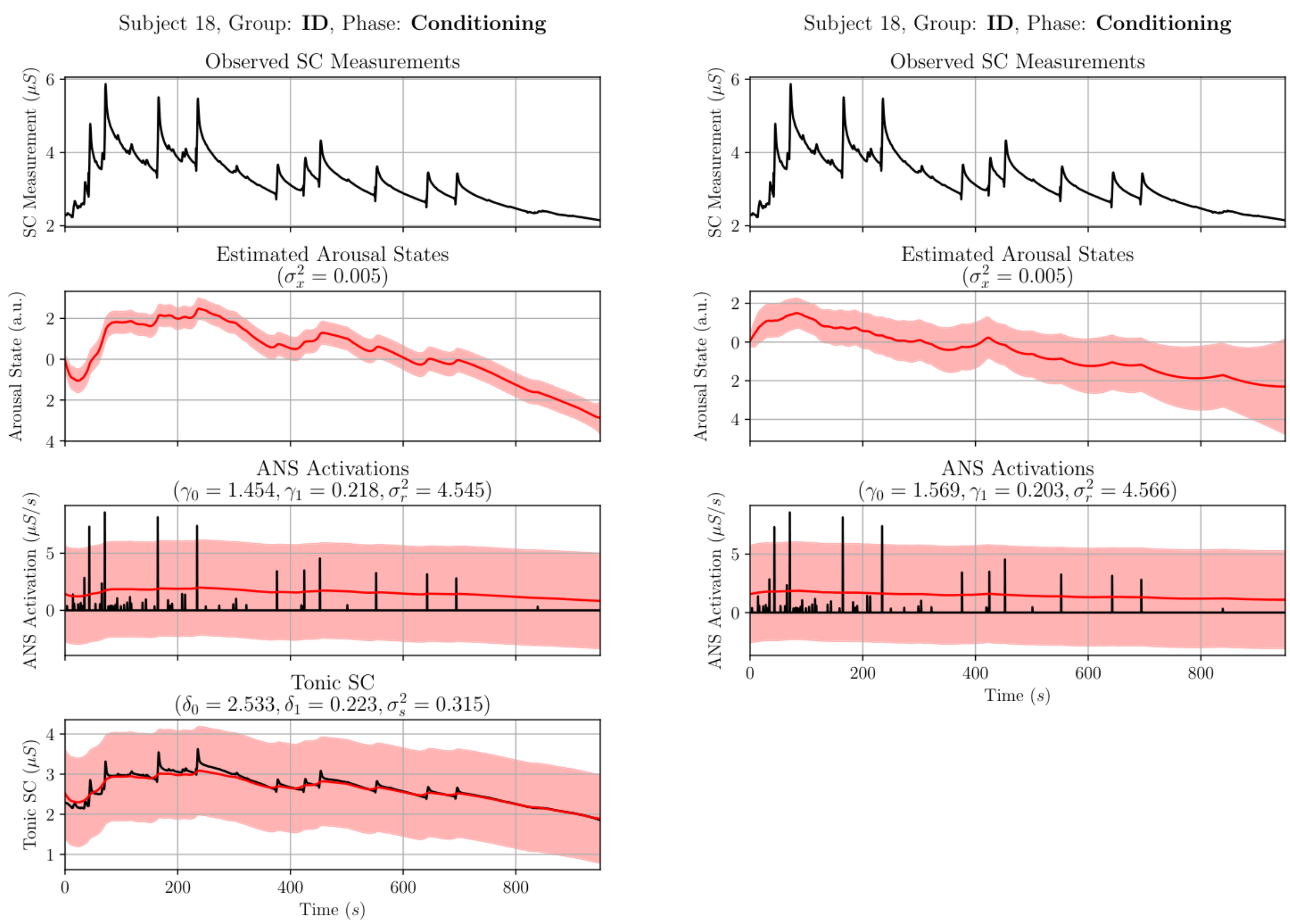


Figure AF: **Subject 18 estimated arousal states during the conditioning phase.** Shown for the MPP+Cont model (left) and the MPP model (right). The first panels show the observed SC measurements, $y$ (black). The second panels show the estimated arousal states, $x_{est}$ (red). The third panels show the ANS activations, $r_{obs}$ (black) and the estimated amplitudes, $r_{est}$ (red). The fourth panel shows the tonic component, $s_{obs}$ (black) and the estimated tonic component, $s_{est}$ (red). Estimates are shown with 95% confidence intervals (CI) (red shaded area). Estimated parameters are reported in the title of each panel.

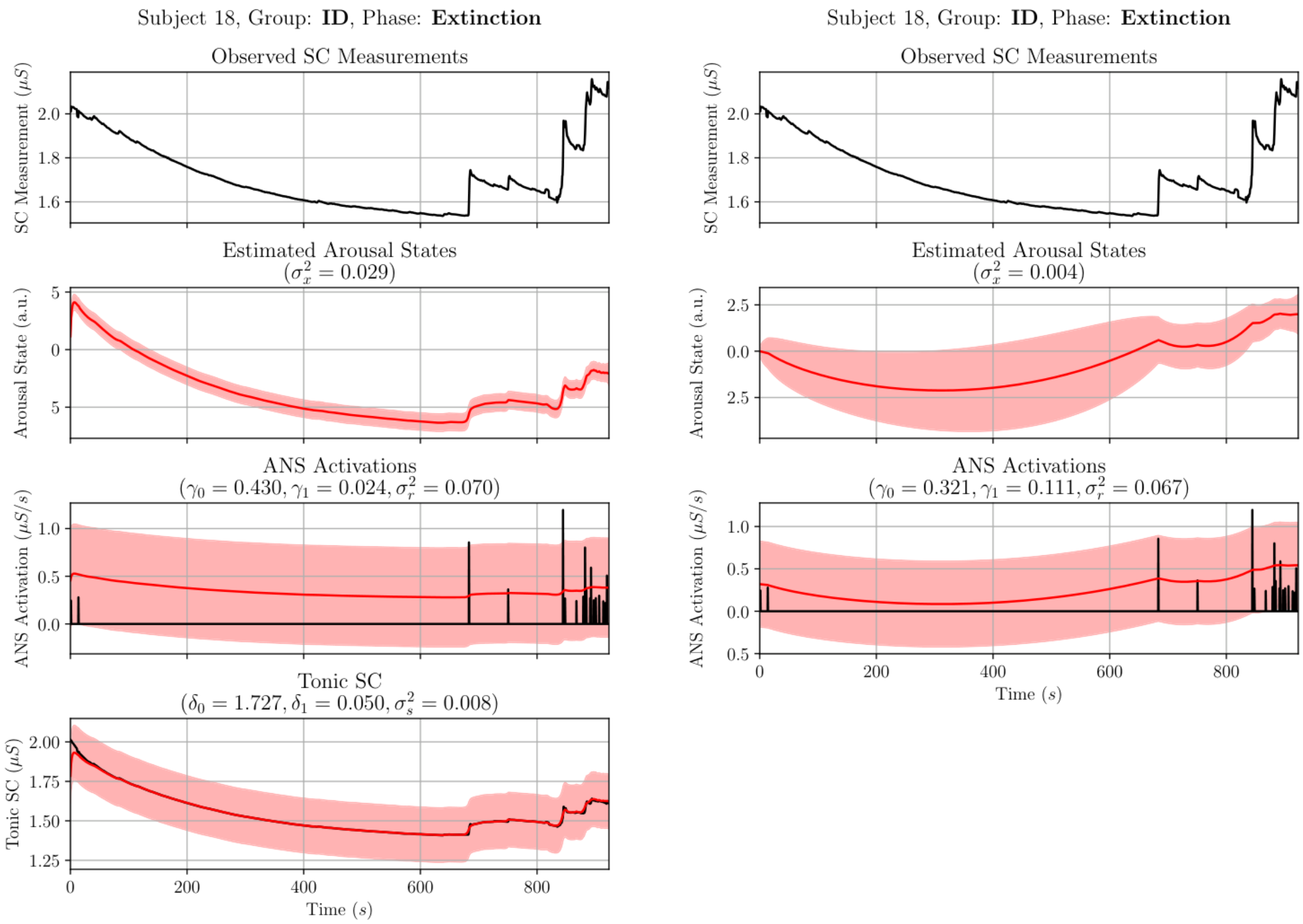


Figure AG: **Subject 18 estimated arousal states during the extinction phase.** Shown for the MPP+Cont model (left) and the MPP model (right). The first panels show the observed SC measurements, $y$ (black). The second panels show the estimated arousal states, $x_{est}$ (red). The third panels show the ANS activations, $r_{obs}$ (black) and the estimated amplitudes, $r_{est}$ (red). The fourth panel shows the tonic component, $s_{obs}$ (black) and the estimated tonic component, $s_{est}$ (red). Estimates are shown with 95% confidence intervals (CI) (red shaded area). Estimated parameters are reported in the title of each panel.

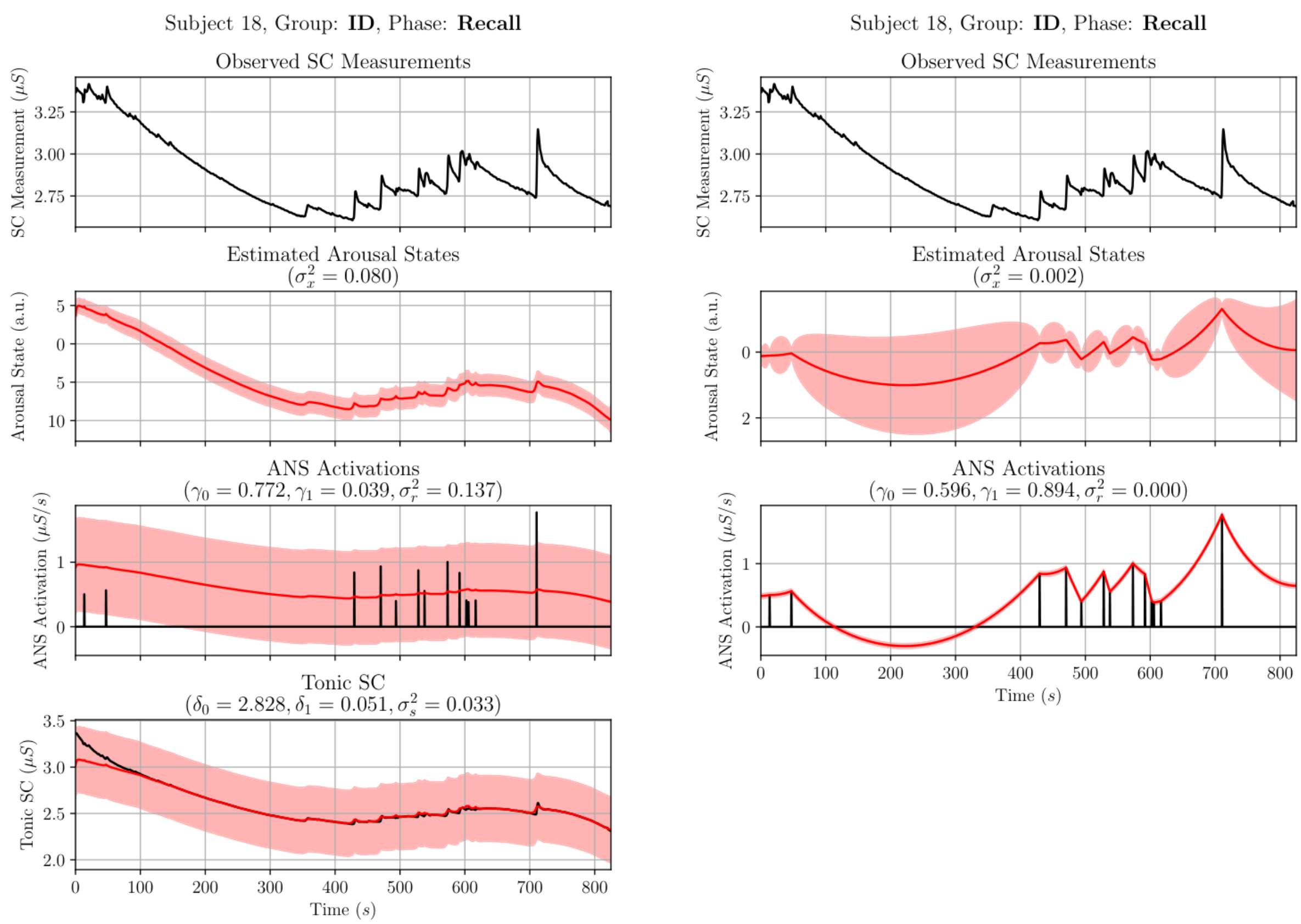


Figure AH: **Subject 18 estimated arousal states during the recall phase.** Shown for the MPP+Cont model (left) and the MPP model (right). The first panels show the observed SC measurements, $y$ (black). The second panels show the estimated arousal states, $x_{est}$ (red). The third panels show the ANS activations, $r_{obs}$ (black) and the estimated amplitudes, $r_{est}$ (red). The fourth panel shows the tonic component, $s_{obs}$ (black) and the estimated tonic component, $s_{est}$ (red). Estimates are shown with 95% confidence intervals (CI) (red shaded area). Estimated parameters are reported in the title of each panel.

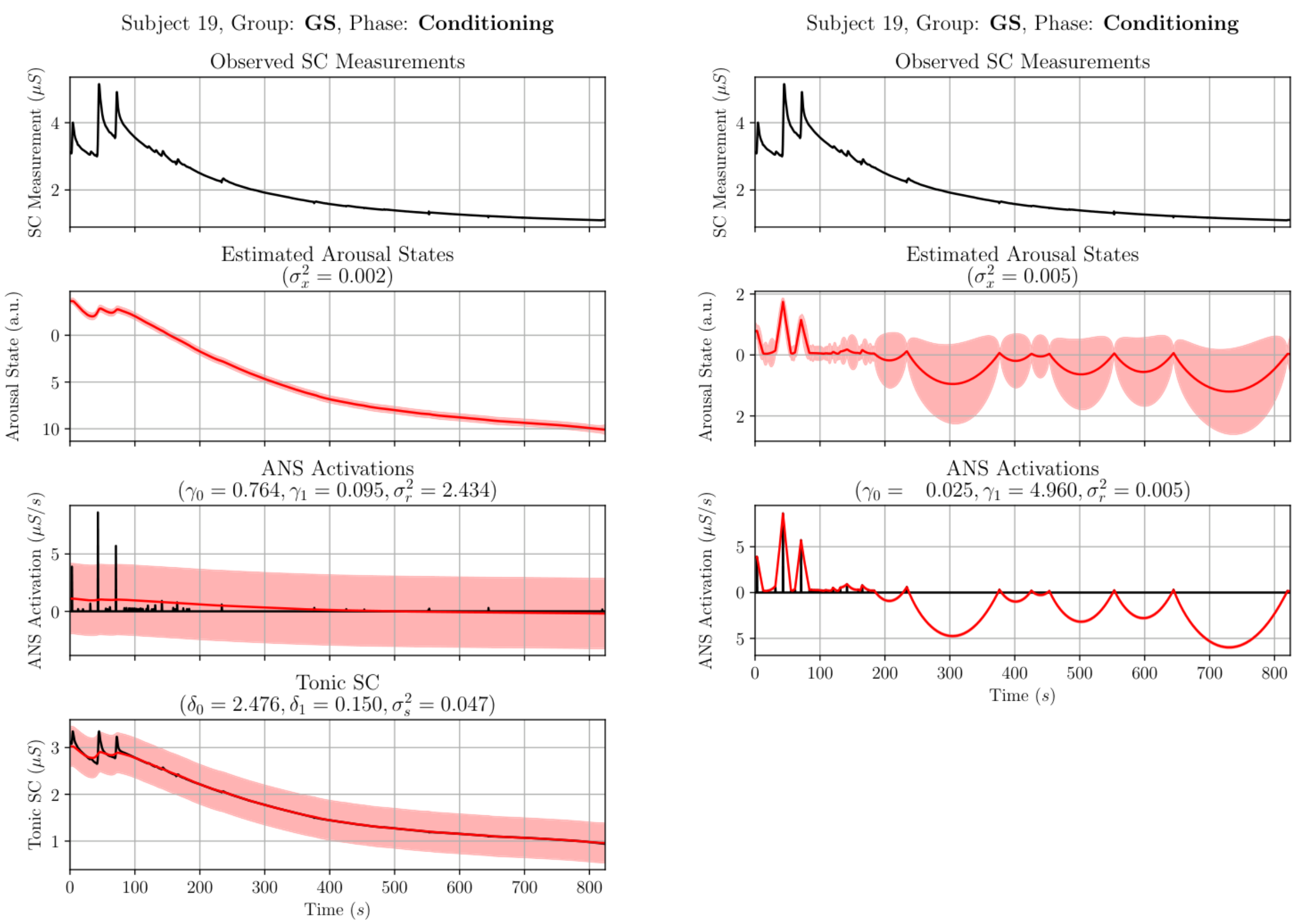


Figure AI: **Subject 19 estimated arousal states during the conditioning phase.** Shown for the MPP+Cont model (left) and the MPP model (right). The first panels show the observed SC measurements, $y$ (black). The second panels show the estimated arousal states, $x_{est}$ (red). The third panels show the ANS activations, $r_{obs}$ (black) and the estimated amplitudes, $r_{est}$ (red). The fourth panel shows the tonic component, $s_{obs}$ (black) and the estimated tonic component, $s_{est}$ (red). Estimates are shown with 95% confidence intervals (CI) (red shaded area). Estimated parameters are reported in the title of each panel.

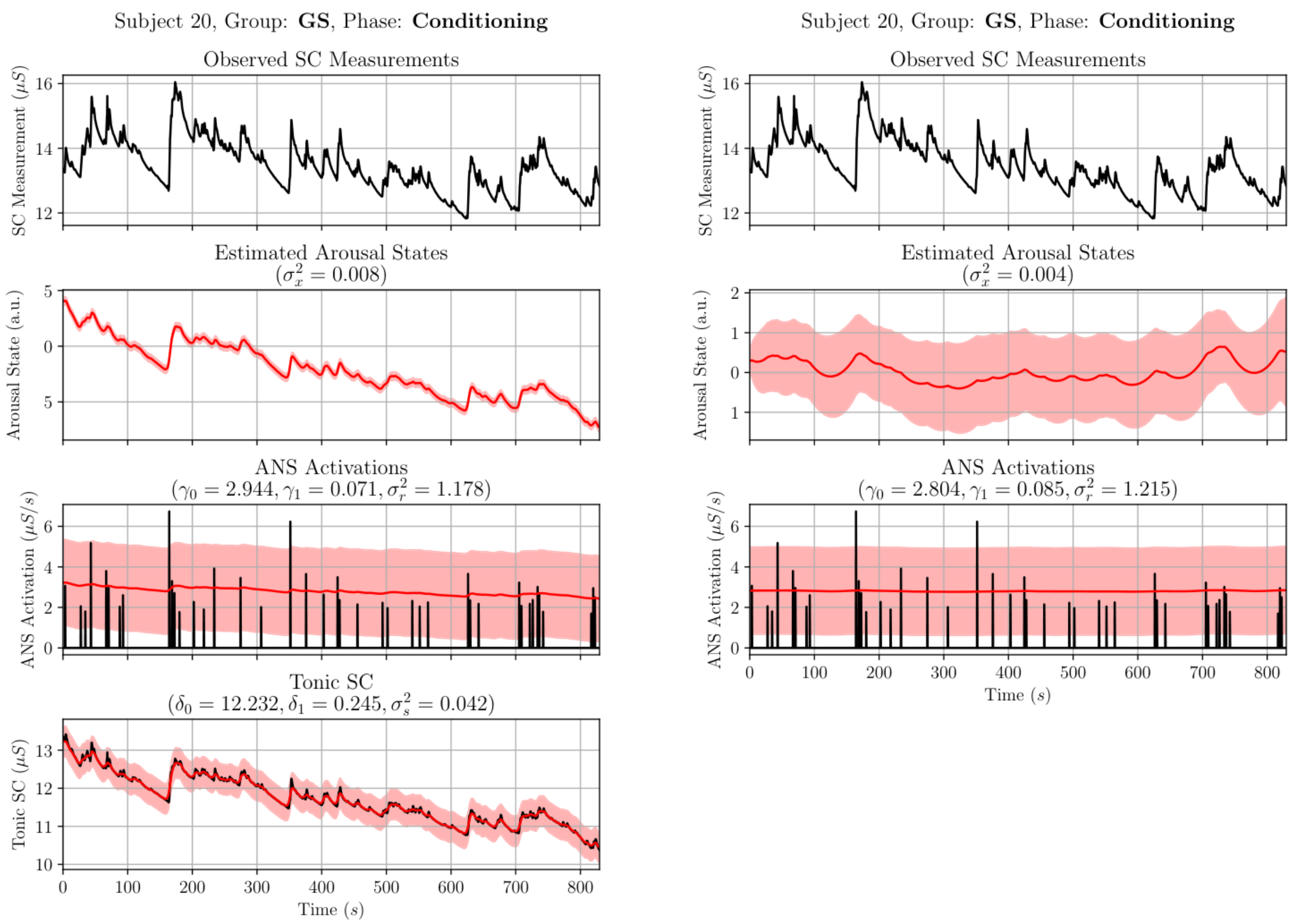


Figure AJ: **Subject 20 estimated arousal states during the conditioning phase.** Shown for the MPP+Cont model (left) and the MPP model (right). The first panels show the observed SC measurements, $y$ (black). The second panels show the estimated arousal states, $x_{est}$ (red). The third panels show the ANS activations, $r_{obs}$ (black) and the estimated amplitudes, $r_{est}$ (red). The fourth panel shows the tonic component, $s_{obs}$ (black) and the estimated tonic component, $s_{est}$ (red). Estimates are shown with 95% confidence intervals (CI) (red shaded area). Estimated parameters are reported in the title of each panel.

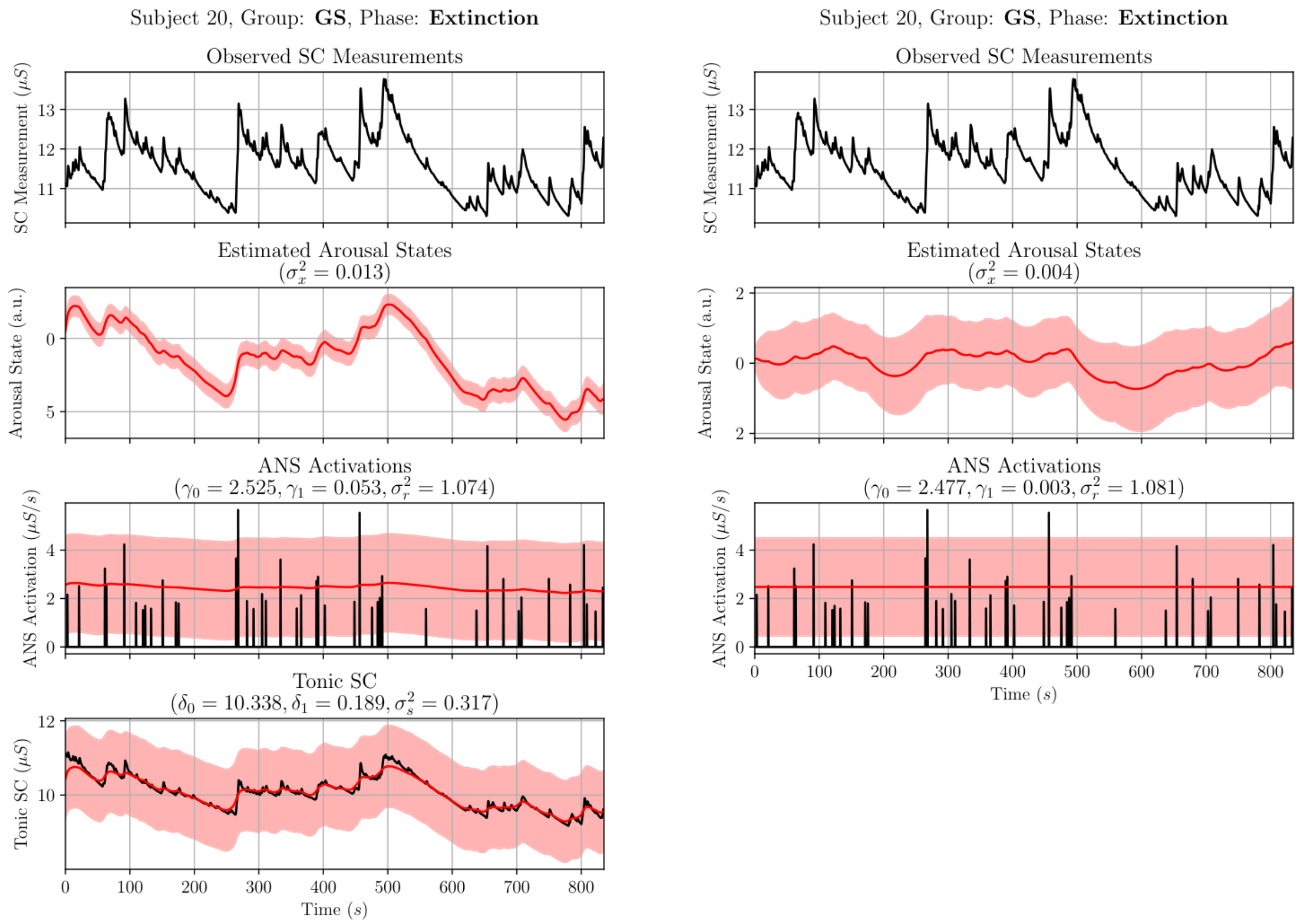


Figure AK: **Subject 20 estimated arousal states during the extinction phase.** Shown for the MPP+Cont model (left) and the MPP model (right). The first panels show the observed SC measurements, $y$ (black). The second panels show the estimated arousal states, $x_{est}$ (red). The third panels show the ANS activations, $r_{obs}$ (black) and the estimated amplitudes, $r_{est}$ (red). The fourth panel shows the tonic component, $s_{obs}$ (black) and the estimated tonic component, $s_{est}$ (red). Estimates are shown with 95% confidence intervals (CI) (red shaded area). Estimated parameters are reported in the title of each panel.

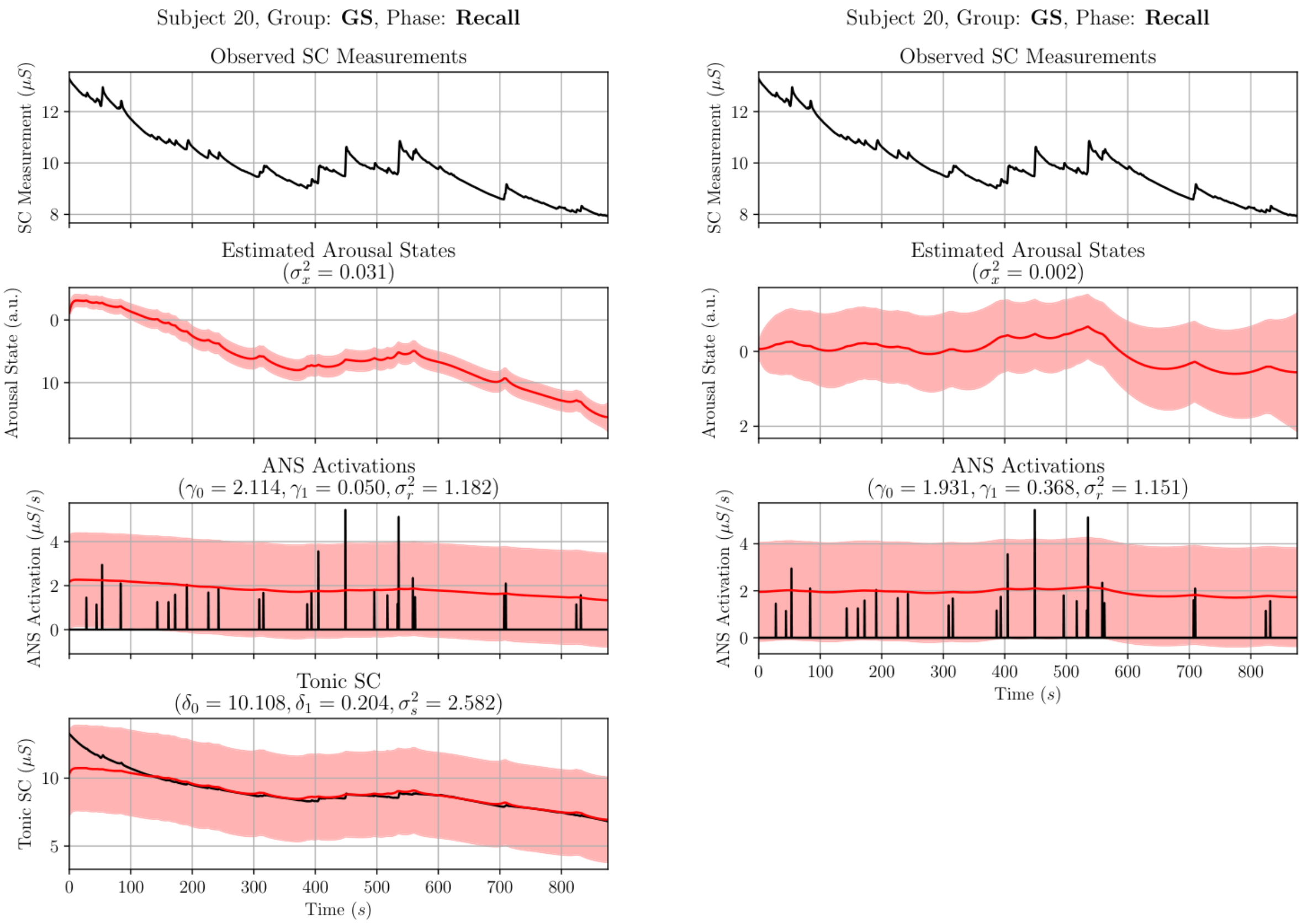


Figure AL: **Subject 20 estimated arousal states during the recall phase.** Shown for the MPP+Cont model (left) and the MPP model (right). The first panels show the observed SC measurements, $y$ (black). The second panels show the estimated arousal states, $x_{est}$ (red). The third panels show the ANS activations, $r_{obs}$ (black) and the estimated amplitudes, $r_{est}$ (red). The fourth panel shows the tonic component, $s_{obs}$ (black) and the estimated tonic component, $s_{est}$ (red). Estimates are shown with 95% confidence intervals (CI) (red shaded area). Estimated parameters are reported in the title of each panel.

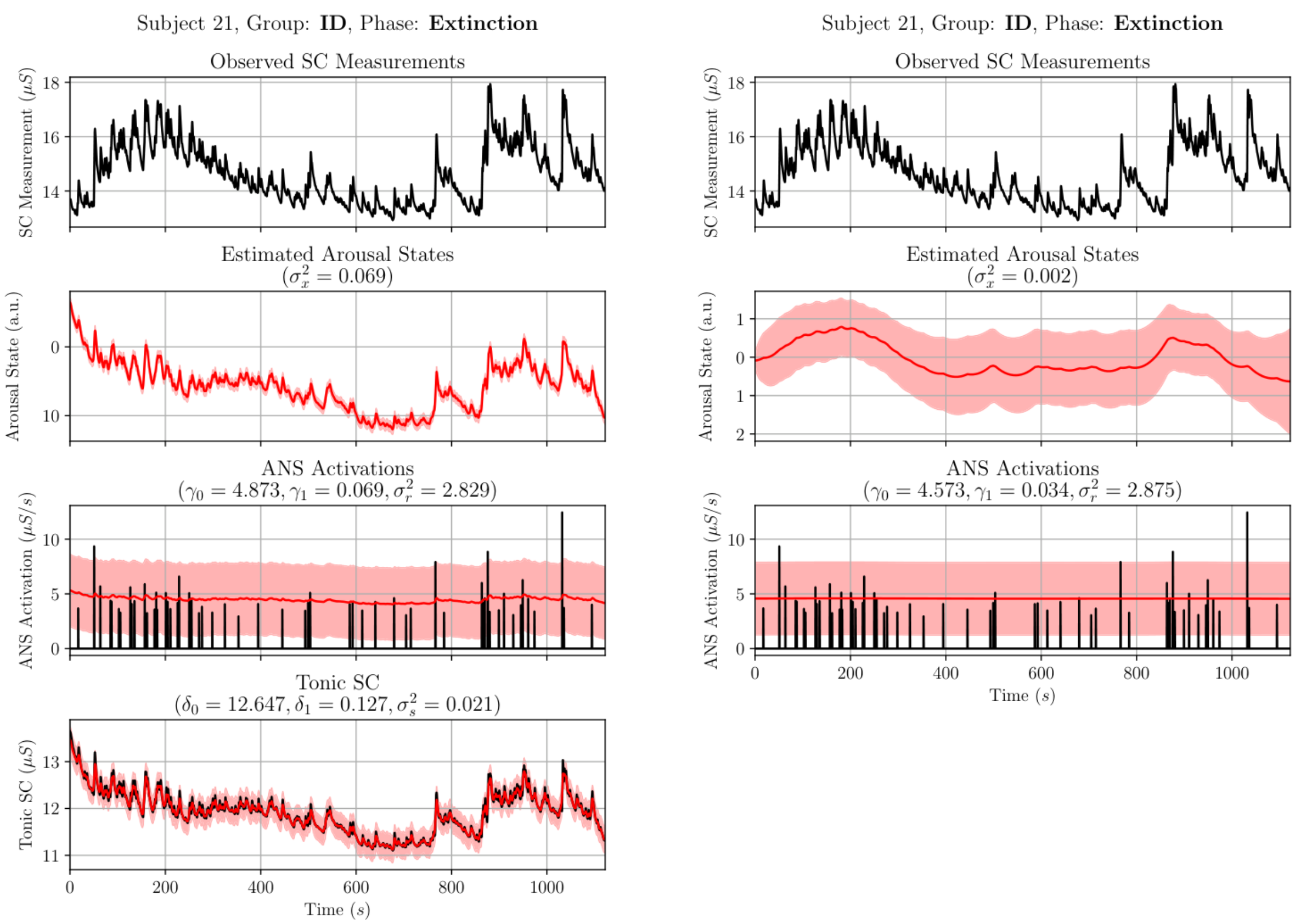


Figure AM: **Subject 21 estimated arousal states during the extinction phase.** Shown for the MPP+Cont model (left) and the MPP model (right). The first panels show the observed SC measurements, $y$ (black). The second panels show the estimated arousal states, $x_{est}$ (red). The third panels show the ANS activations, $r_{obs}$ (black) and the estimated amplitudes, $r_{est}$ (red). The fourth panel shows the tonic component, $s_{obs}$ (black) and the estimated tonic component, $s_{est}$ (red). Estimates are shown with 95% confidence intervals (CI) (red shaded area). Estimated parameters are reported in the title of each panel.

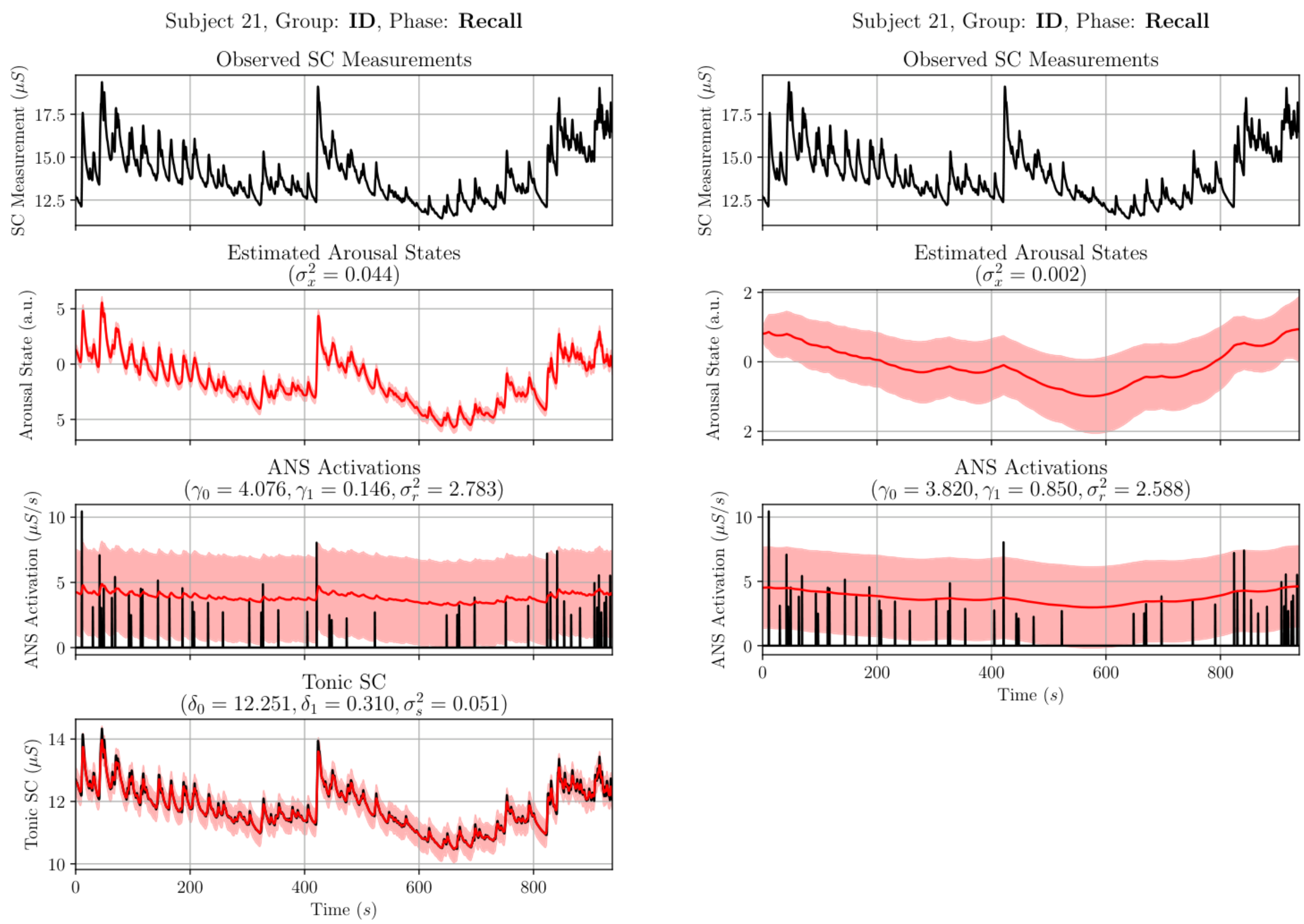


Figure AN: **Subject 21 estimated arousal states during the recall phase.** Shown for the MPP+Cont model (left) and the MPP model (right). The first panels show the observed SC measurements, $y$ (black). The second panels show the estimated arousal states, $x_{est}$ (red). The third panels show the ANS activations, $r_{obs}$ (black) and the estimated amplitudes, $r_{est}$ (red). The fourth panel shows the tonic component, $s_{obs}$ (black) and the estimated tonic component, $s_{est}$ (red). Estimates are shown with 95% confidence intervals (CI) (red shaded area). Estimated parameters are reported in the title of each panel.

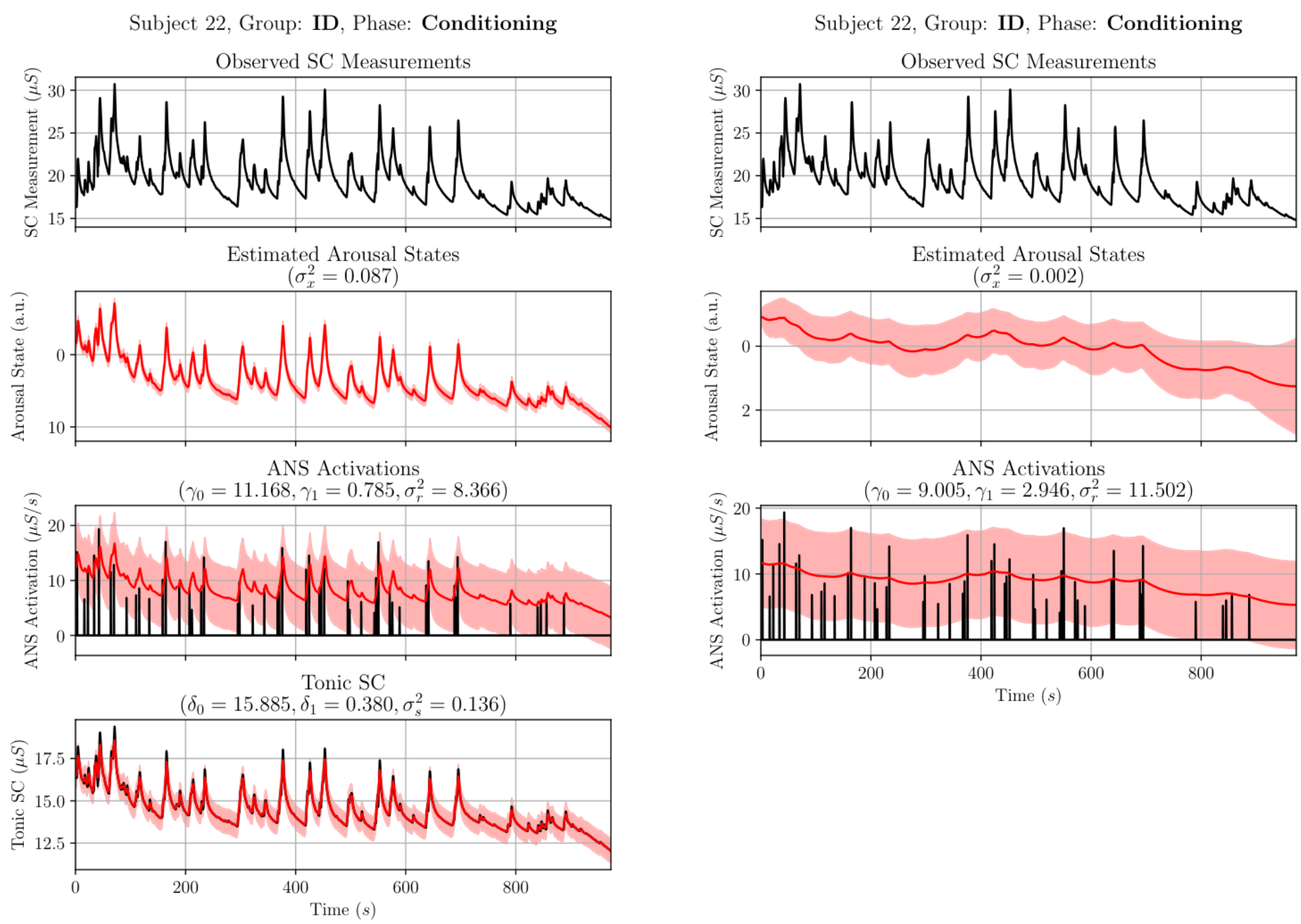


Figure AO: **Subject 22 estimated arousal states during the conditioning phase.** Shown for the MPP+Cont model (left) and the MPP model (right). The first panels show the observed SC measurements, $y$ (black). The second panels show the estimated arousal states, $x_{est}$ (red). The third panels show the ANS activations, $r_{obs}$ (black) and the estimated amplitudes, $r_{est}$ (red). The fourth panel shows the tonic component, $s_{obs}$ (black) and the estimated tonic component, $s_{est}$ (red). Estimates are shown with 95% confidence intervals (CI) (red shaded area). Estimated parameters are reported in the title of each panel.

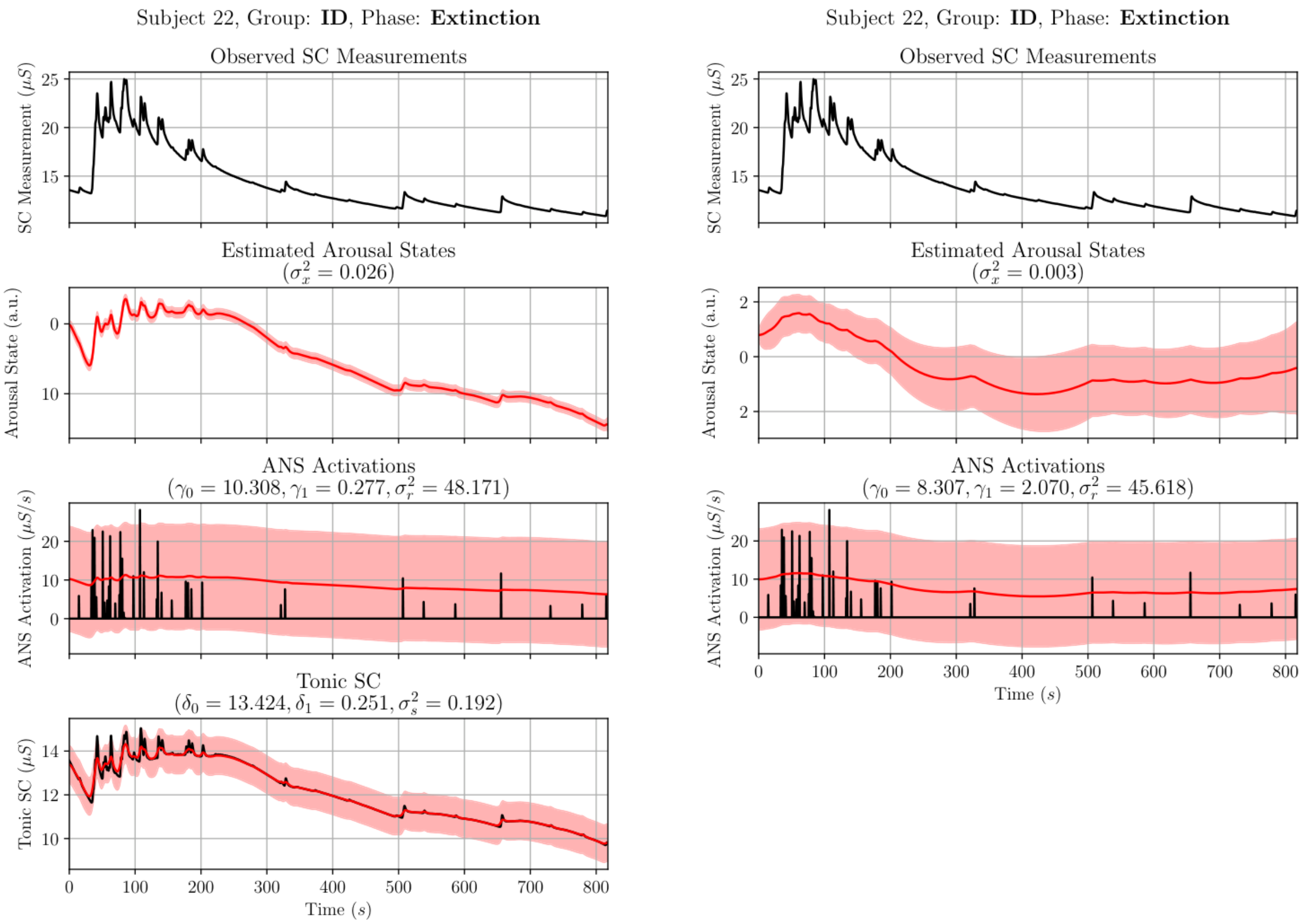


Figure AP: **Subject 22 estimated arousal states during the extinction phase.** Shown for the MPP+Cont model (left) and the MPP model (right). The first panels show the observed SC measurements, $y$ (black). The second panels show the estimated arousal states, $x_{est}$ (red). The third panels show the ANS activations, $r_{obs}$ (black) and the estimated amplitudes, $r_{est}$ (red). The fourth panel shows the tonic component, $s_{obs}$ (black) and the estimated tonic component, $s_{est}$ (red). Estimates are shown with 95% confidence intervals (CI) (red shaded area). Estimated parameters are reported in the title of each panel.

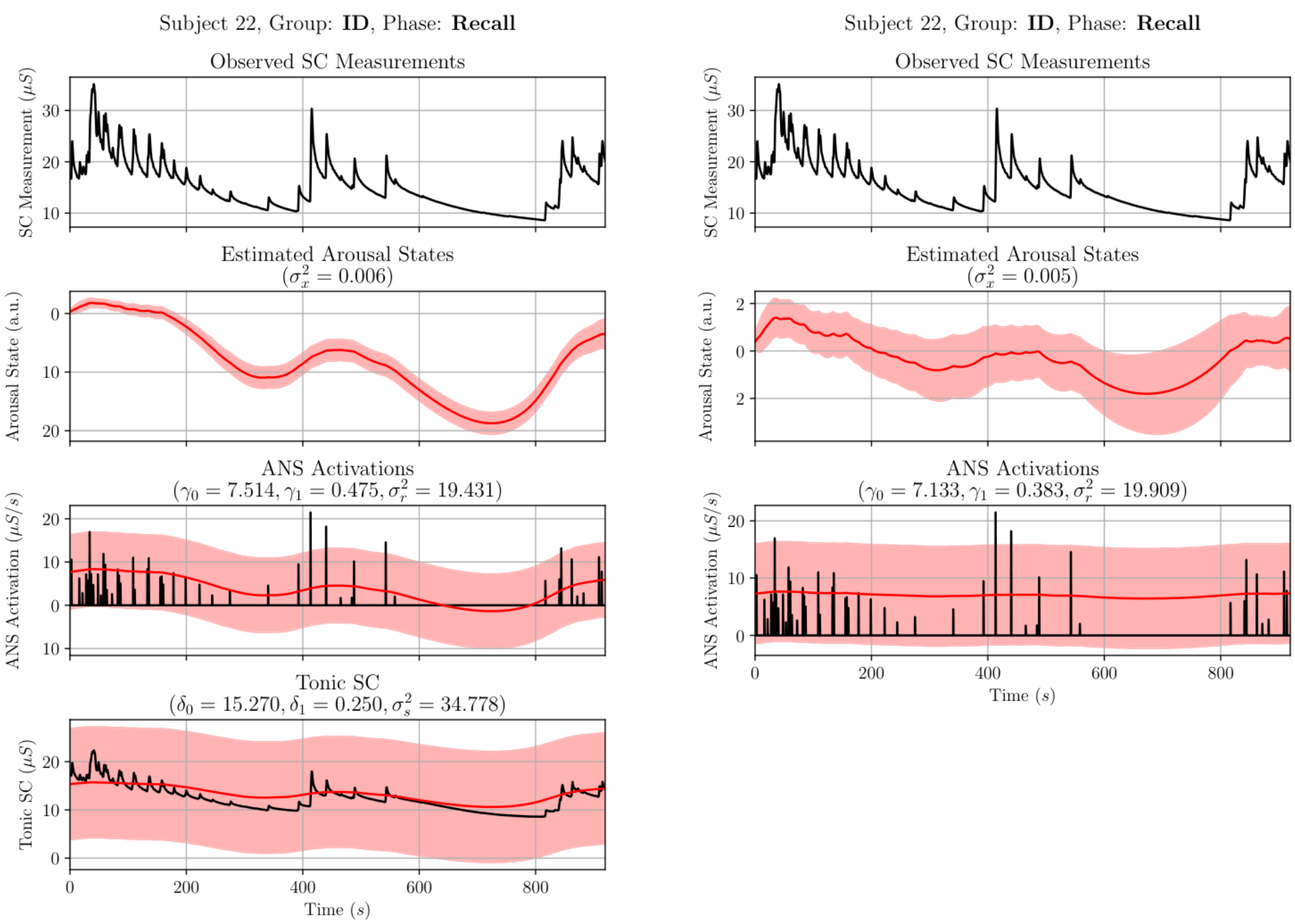


Figure AQ: **Subject 22 estimated arousal states during the recall phase.** Shown for the MPP+Cont model (left) and the MPP model (right). The first panels show the observed SC measurements, $y$ (black). The second panels show the estimated arousal states, $x_{est}$ (red). The third panels show the ANS activations, $r_{obs}$ (black) and the estimated amplitudes, $r_{est}$ (red). The fourth panel shows the tonic component, $s_{obs}$ (black) and the estimated tonic component, $s_{est}$ (red). Estimates are shown with 95% confidence intervals (CI) (red shaded area). Estimated parameters are reported in the title of each panel.

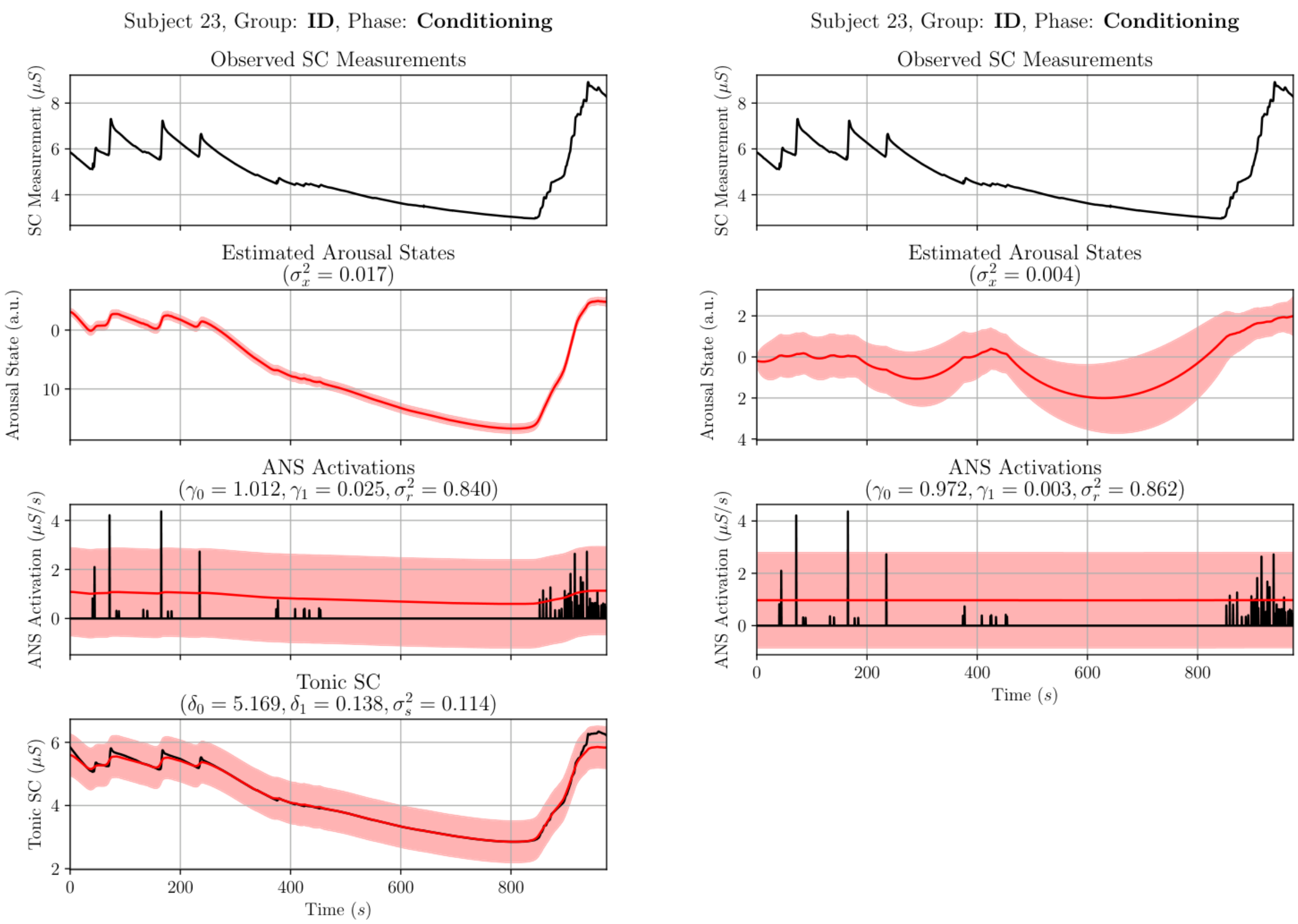


Figure AR: **Subject 23 estimated arousal states during the conditioning phase.** Shown for the MPP+Cont model (left) and the MPP model (right). The first panels show the observed SC measurements, $y$ (black). The second panels show the estimated arousal states, $x_{est}$ (red). The third panels show the ANS activations, $r_{obs}$ (black) and the estimated amplitudes, $r_{est}$ (red). The fourth panel shows the tonic component, $s_{obs}$ (black) and the estimated tonic component, $s_{est}$ (red). Estimates are shown with 95% confidence intervals (CI) (red shaded area). Estimated parameters are reported in the title of each panel.

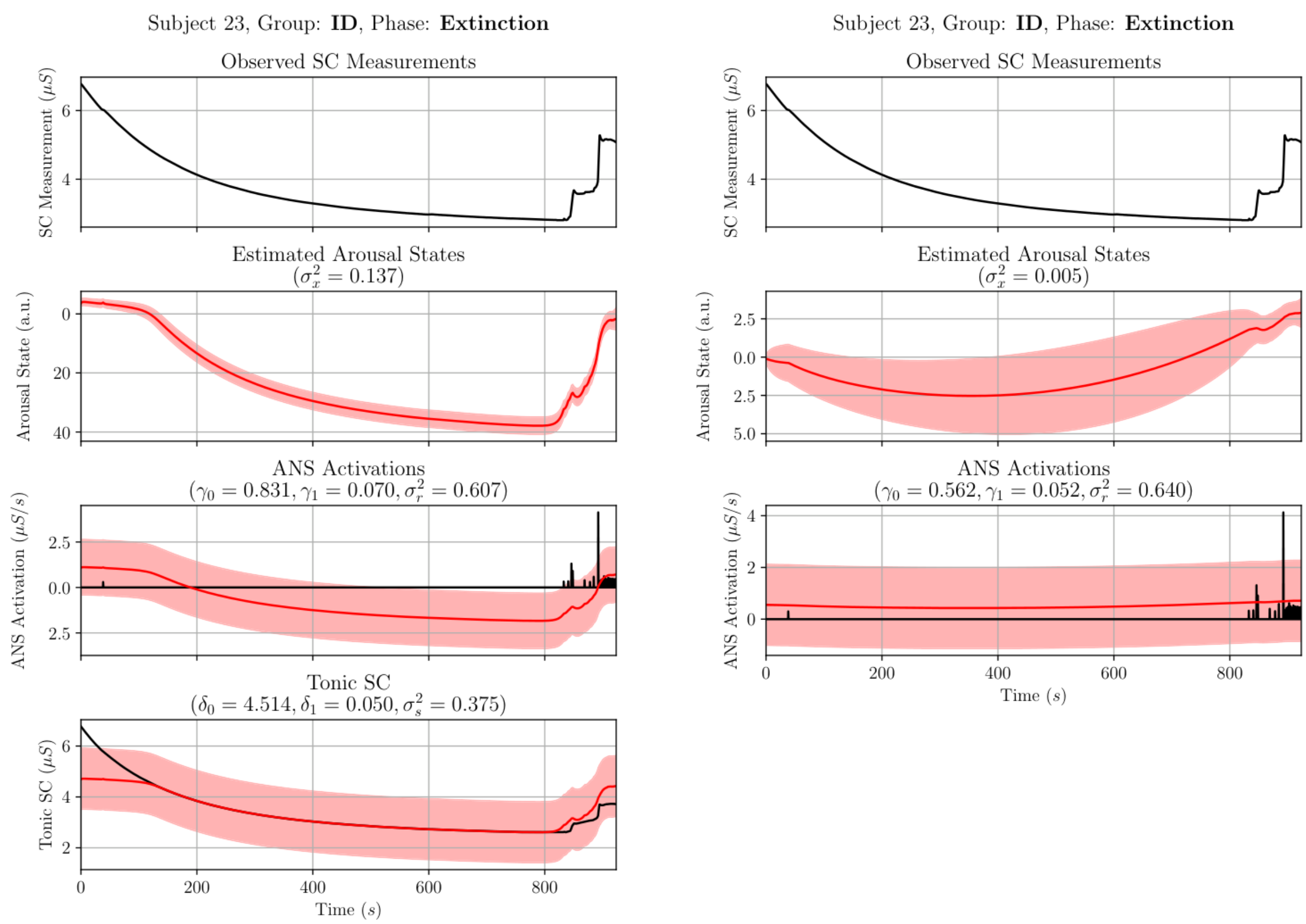


Figure AS: **Subject 23 estimated arousal states during the extinction phase.** Shown for the MPP+Cont model (left) and the MPP model (right). The first panels show the observed SC measurements, $y$ (black). The second panels show the estimated arousal states, $x_{est}$ (red). The third panels show the ANS activations, $r_{obs}$ (black) and the estimated amplitudes, $r_{est}$ (red). The fourth panel shows the tonic component, $s_{obs}$ (black) and the estimated tonic component, $s_{est}$ (red). Estimates are shown with 95% confidence intervals (CI) (red shaded area). Estimated parameters are reported in the title of each panel.

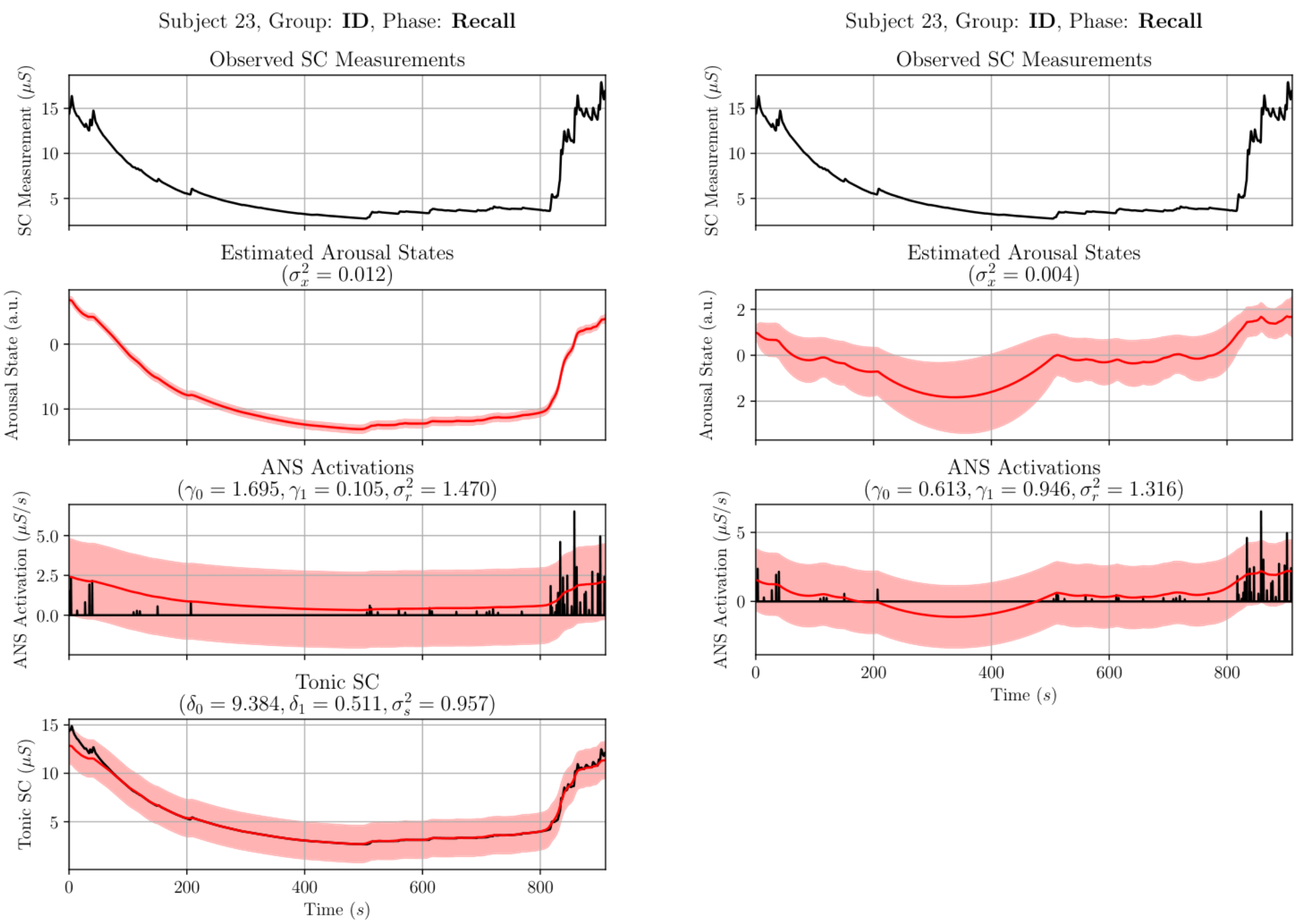


Figure AT: **Subject 23 estimated arousal states during the recall phase.** Shown for the MPP+Cont model (left) and the MPP model (right). The first panels show the observed SC measurements, $y$ (black). The second panels show the estimated arousal states, $x_{est}$ (red). The third panels show the ANS activations, $r_{obs}$ (black) and the estimated amplitudes, $r_{est}$ (red). The fourth panel shows the tonic component, $s_{obs}$ (black) and the estimated tonic component, $s_{est}$ (red). Estimates are shown with 95% confidence intervals (CI) (red shaded area). Estimated parameters are reported in the title of each panel.

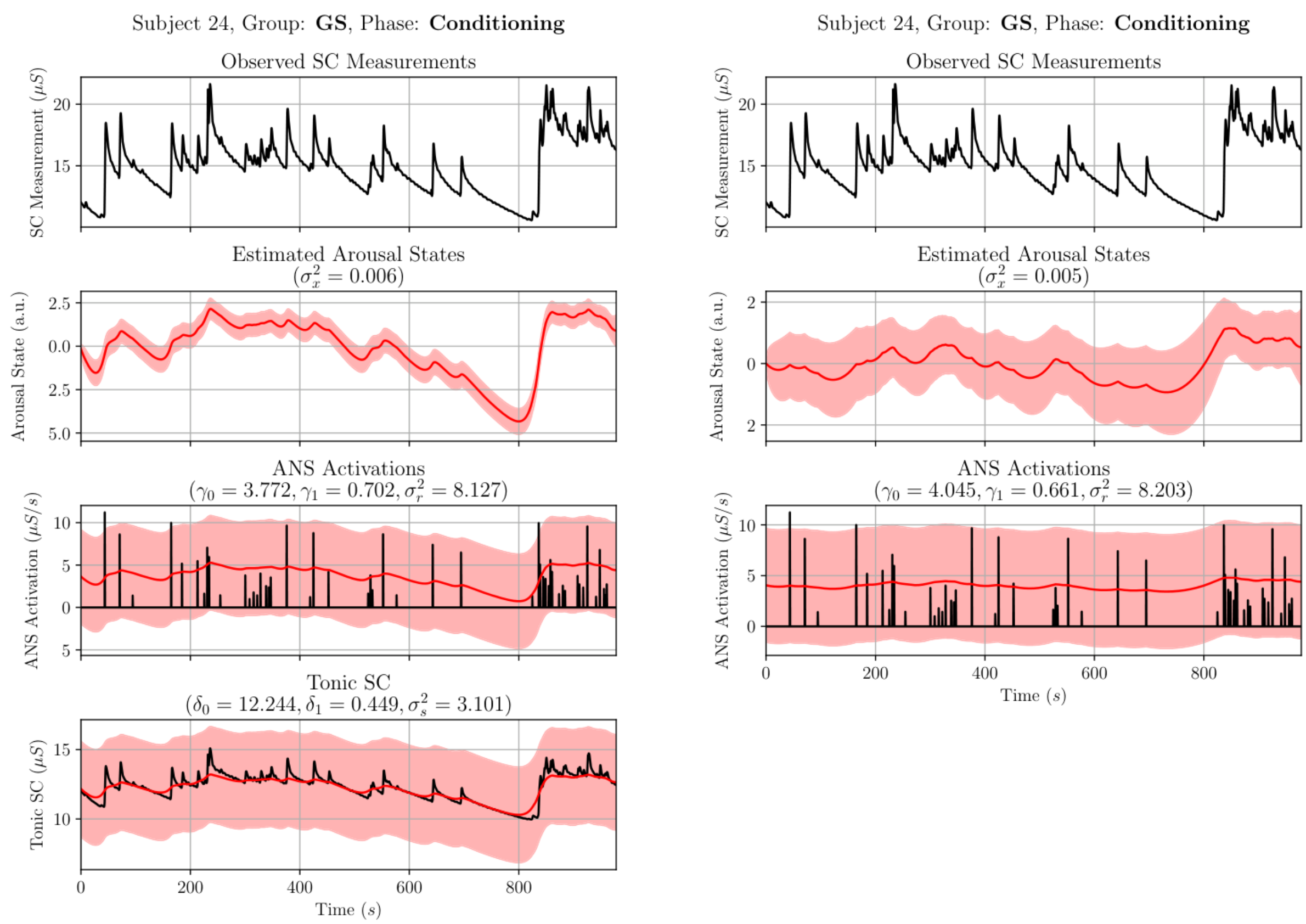


Figure AU: **Subject 24 estimated arousal states during the conditioning phase.** Shown for the MPP+Cont model (left) and the MPP model (right). The first panels show the observed SC measurements, $y$ (black). The second panels show the estimated arousal states, $x_{est}$ (red). The third panels show the ANS activations, $r_{obs}$ (black) and the estimated amplitudes, $r_{est}$ (red). The fourth panel shows the tonic component, $s_{obs}$ (black) and the estimated tonic component, $s_{est}$ (red). Estimates are shown with 95% confidence intervals (CI) (red shaded area). Estimated parameters are reported in the title of each panel.

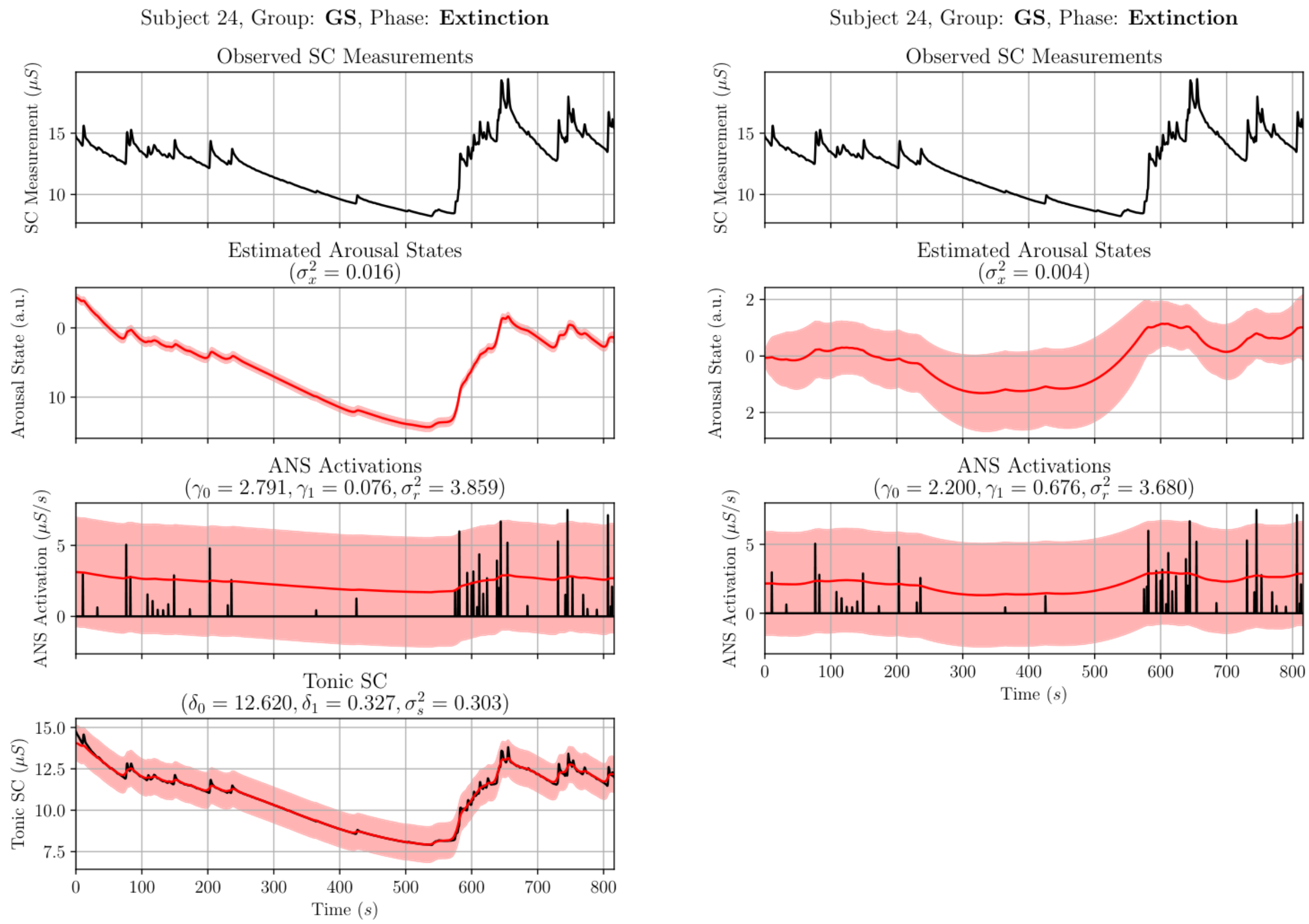


Figure AV: **Subject 24 estimated arousal states during the extinction phase.** Shown for the MPP+Cont model (left) and the MPP model (right). The first panels show the observed SC measurements, $y$ (black). The second panels show the estimated arousal states, $x_{est}$ (red). The third panels show the ANS activations, $r_{obs}$ (black) and the estimated amplitudes, $r_{est}$ (red). The fourth panel shows the tonic component, $s_{obs}$ (black) and the estimated tonic component, $s_{est}$ (red). Estimates are shown with 95% confidence intervals (CI) (red shaded area). Estimated parameters are reported in the title of each panel.

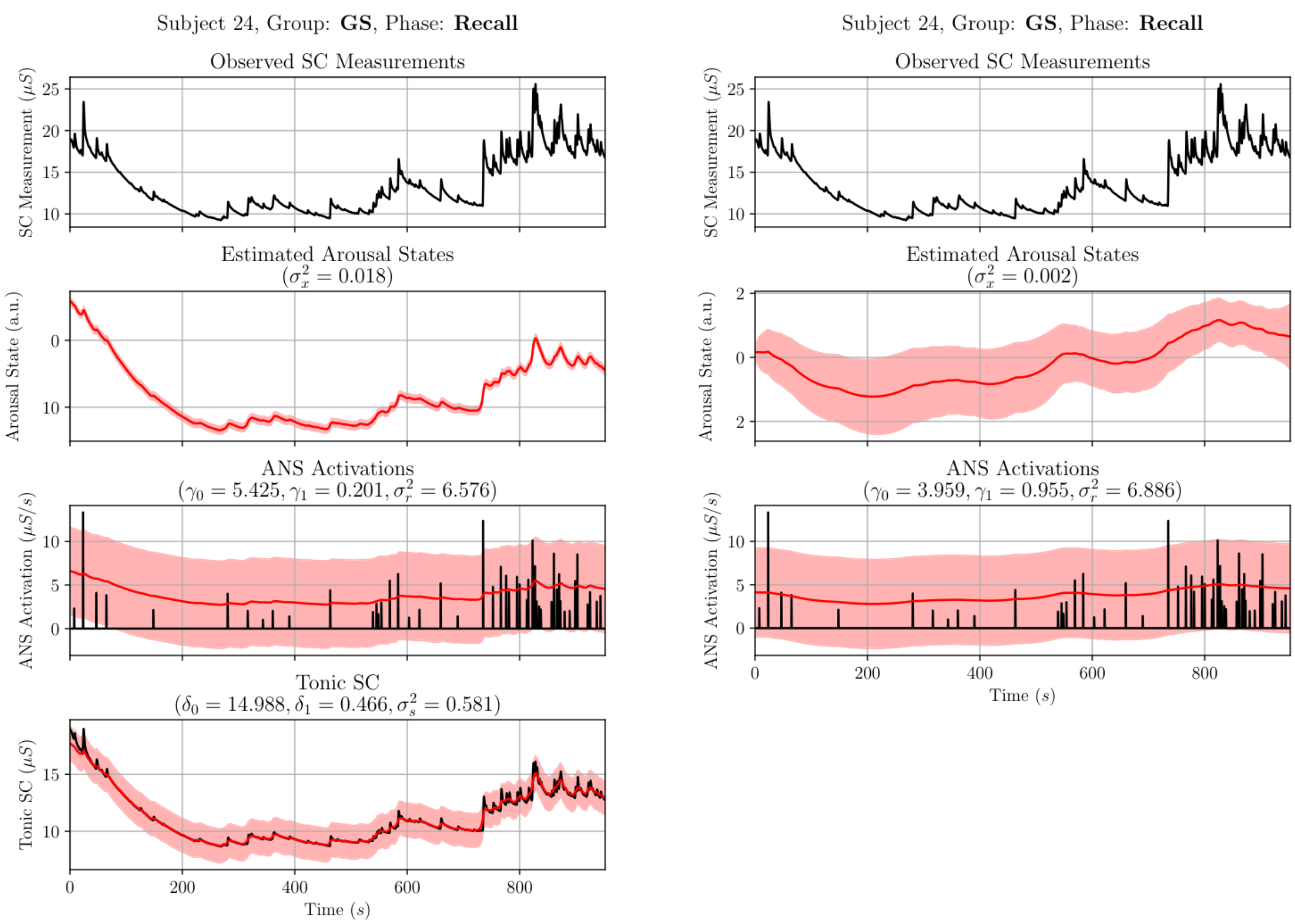


Figure AW: **Subject 24 estimated arousal states during the recall phase.** Shown for the MPP+Cont model (left) and the MPP model (right). The first panels show the observed SC measurements, $y$ (black). The second panels show the estimated arousal states, $x_{est}$ (red). The third panels show the ANS activations, $r_{obs}$ (black) and the estimated amplitudes, $r_{est}$ (red). The fourth panel shows the tonic component, $s_{obs}$ (black) and the estimated tonic component, $s_{est}$ (red). Estimates are shown with 95% confidence intervals (CI) (red shaded area). Estimated parameters are reported in the title of each panel.

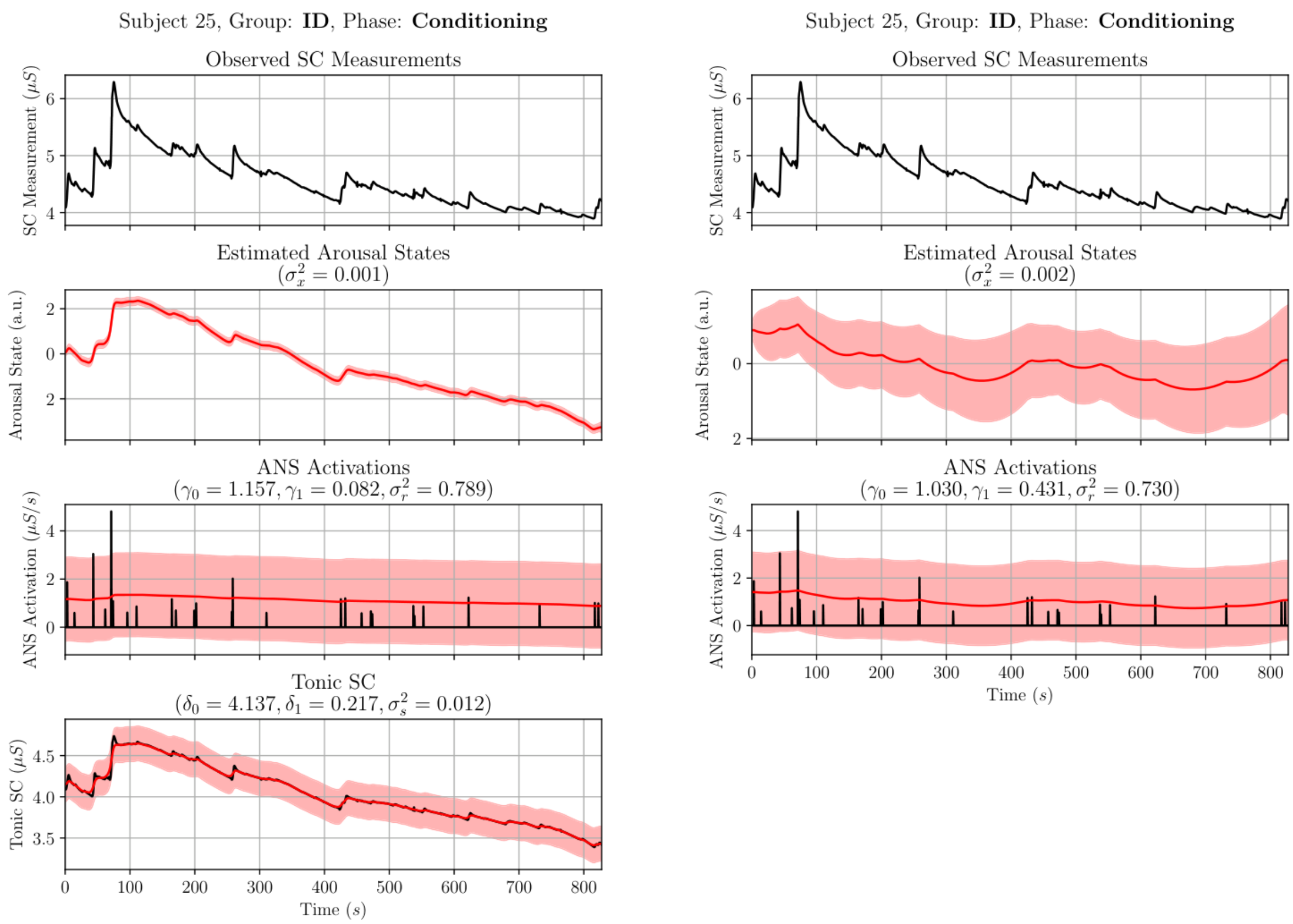


Figure AX: **Subject 25 estimated arousal states during the conditioning phase.** Shown for the MPP+Cont model (left) and the MPP model (right). The first panels show the observed SC measurements, $y$ (black). The second panels show the estimated arousal states, $x_{est}$ (red). The third panels show the ANS activations, $r_{obs}$ (black) and the estimated amplitudes, $r_{est}$ (red). The fourth panel shows the tonic component, $s_{obs}$ (black) and the estimated tonic component, $s_{est}$ (red). Estimates are shown with 95% confidence intervals (CI) (red shaded area). Estimated parameters are reported in the title of each panel.

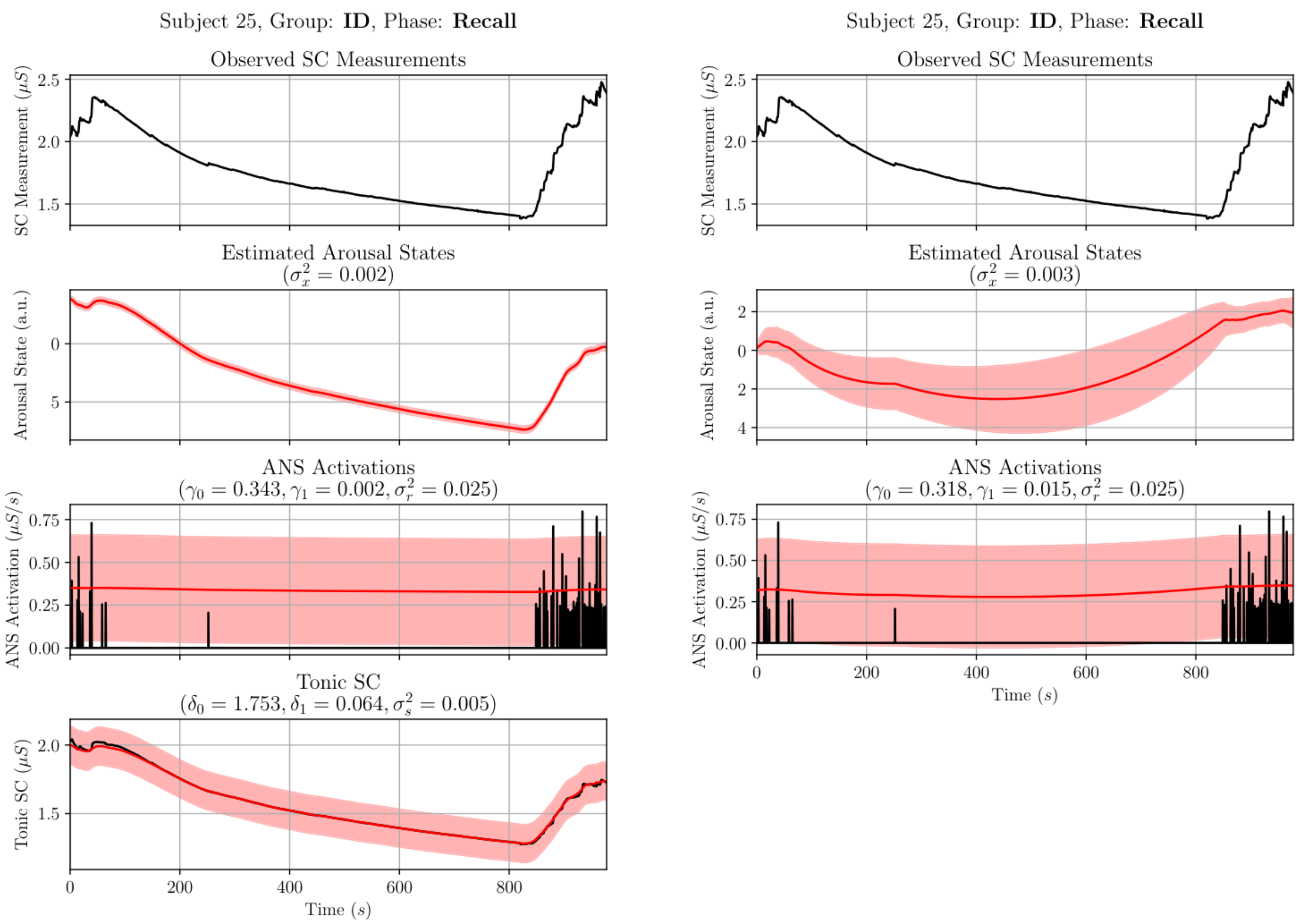


Figure AY: **Subject 25 estimated arousal states during the recall phase.** Shown for the MPP+Cont model (left) and the MPP model (right). The first panels show the observed SC measurements, $y$ (black). The second panels show the estimated arousal states, $x_{est}$ (red). The third panels show the ANS activations, $r_{obs}$ (black) and the estimated amplitudes, $r_{est}$ (red). The fourth panel shows the tonic component, $s_{obs}$ (black) and the estimated tonic component, $s_{est}$ (red). Estimates are shown with 95% confidence intervals (CI) (red shaded area). Estimated parameters are reported in the title of each panel.

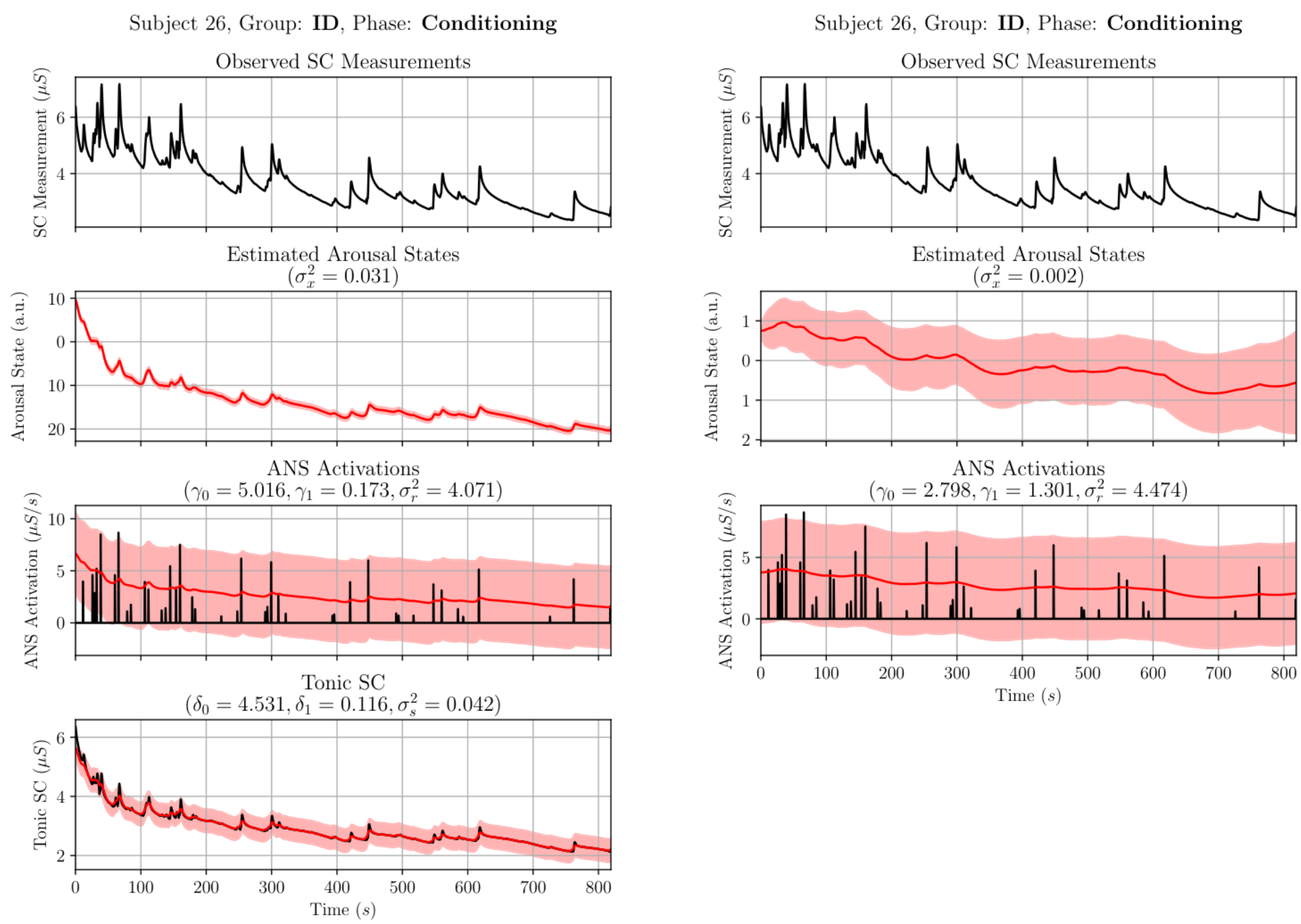


Figure AZ: **Subject 26 estimated arousal states during the conditioning phase.** Shown for the MPP+Cont model (left) and the MPP model (right). The first panels show the observed SC measurements, $y$ (black). The second panels show the estimated arousal states, $x_{est}$ (red). The third panels show the ANS activations, $r_{obs}$ (black) and the estimated amplitudes, $r_{est}$ (red). The fourth panel shows the tonic component, $s_{obs}$ (black) and the estimated tonic component, $s_{est}$ (red). Estimates are shown with 95% confidence intervals (CI) (red shaded area). Estimated parameters are reported in the title of each panel.

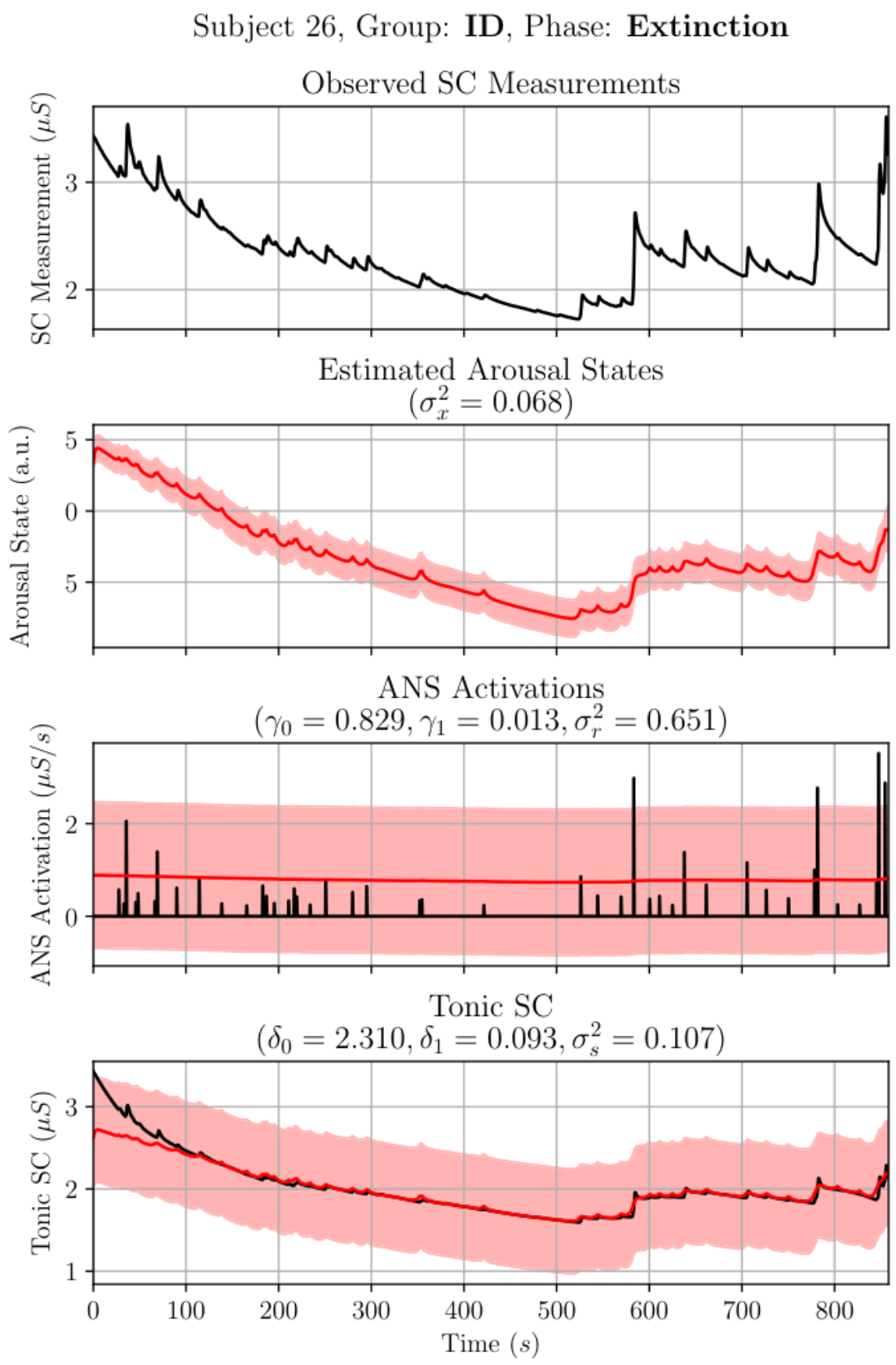


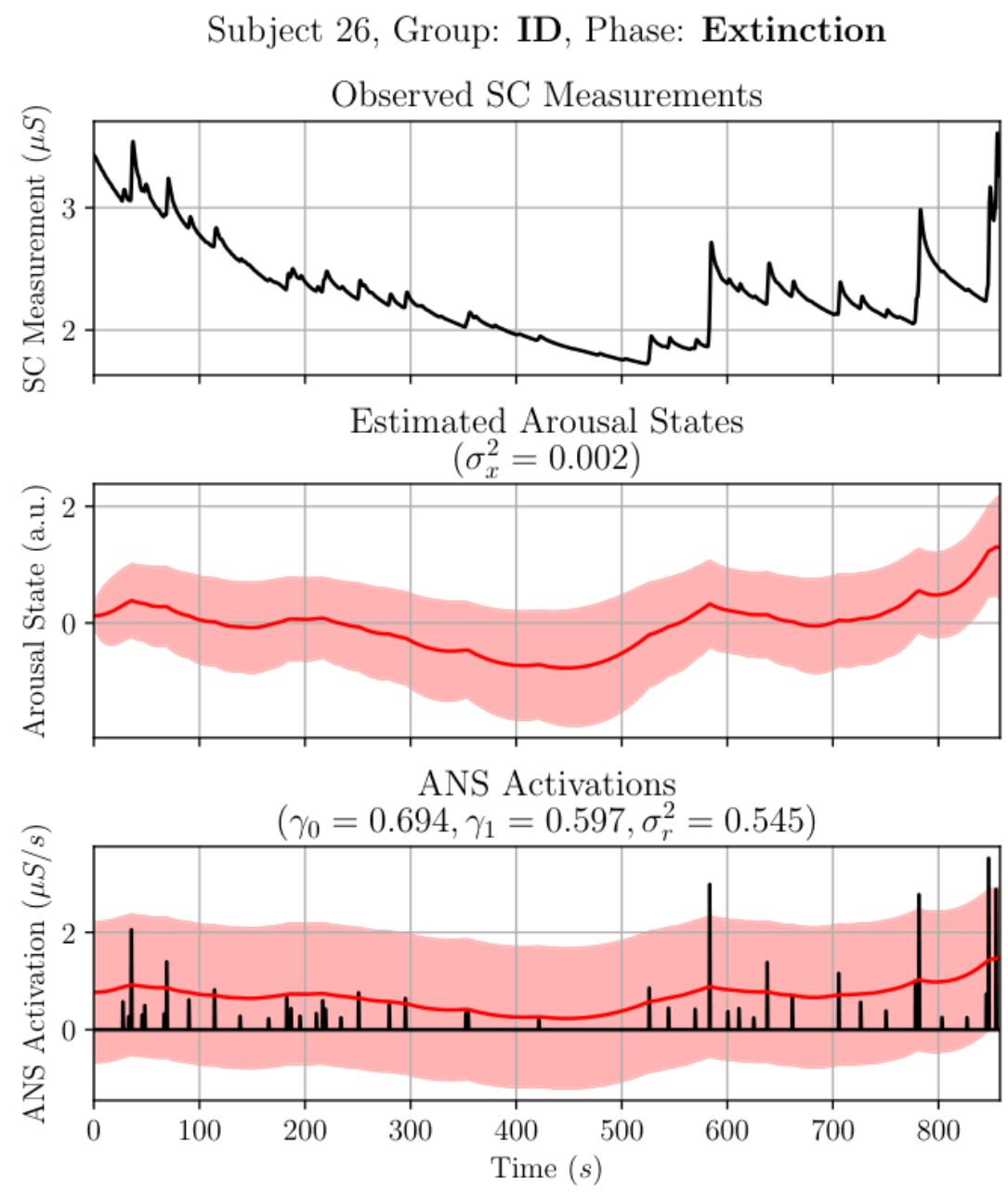


Figure BA: **Subject 26 estimated arousal states during the extinction phase.** Shown for the MPP+Cont model (left) and the MPP model (right). The first panels show the observed SC measurements, $y$ (black). The second panels show the estimated arousal states, $x_{est}$ (red). The third panels show the ANS activations, $r_{obs}$ (black) and the estimated amplitudes, $r_{est}$ (red). The fourth panel shows the tonic component, $s_{obs}$ (black) and the estimated tonic component, $s_{est}$ (red). Estimates are shown with 95% confidence intervals (CI) (red shaded area). Estimated parameters are reported in the title of each panel.

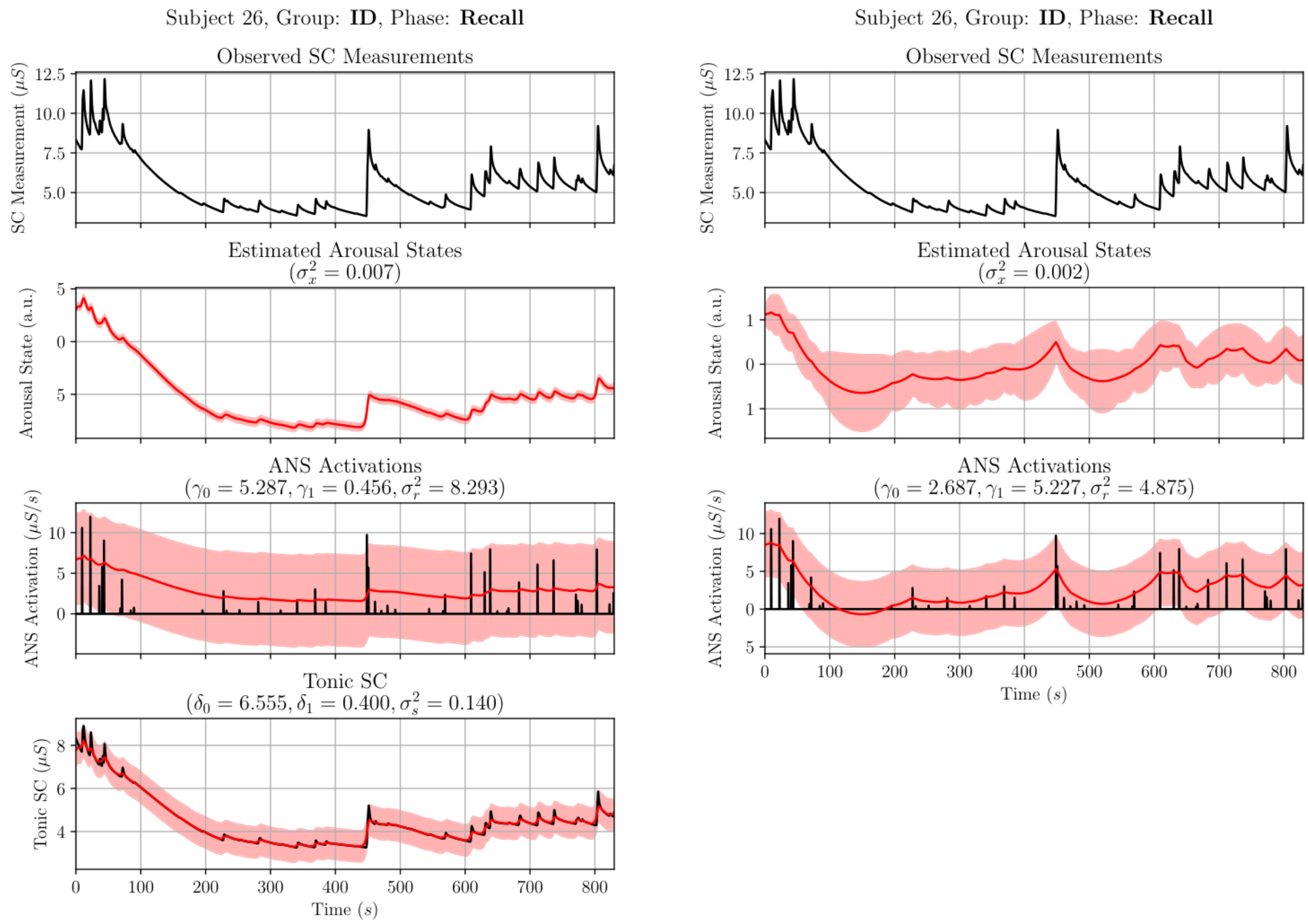


Figure BB: **Subject 26 estimated arousal states during the recall phase.** Shown for the MPP+Cont model (left) and the MPP model (right). The first panels show the observed SC measurements, $y$ (black). The second panels show the estimated arousal states, $x_{est}$ (red). The third panels show the ANS activations, $r_{obs}$ (black) and the estimated amplitudes, $r_{est}$ (red). The fourth panel shows the tonic component, $s_{obs}$ (black) and the estimated tonic component, $s_{est}$ (red). Estimates are shown with 95% confidence intervals (CI) (red shaded area). Estimated parameters are reported in the title of each panel.

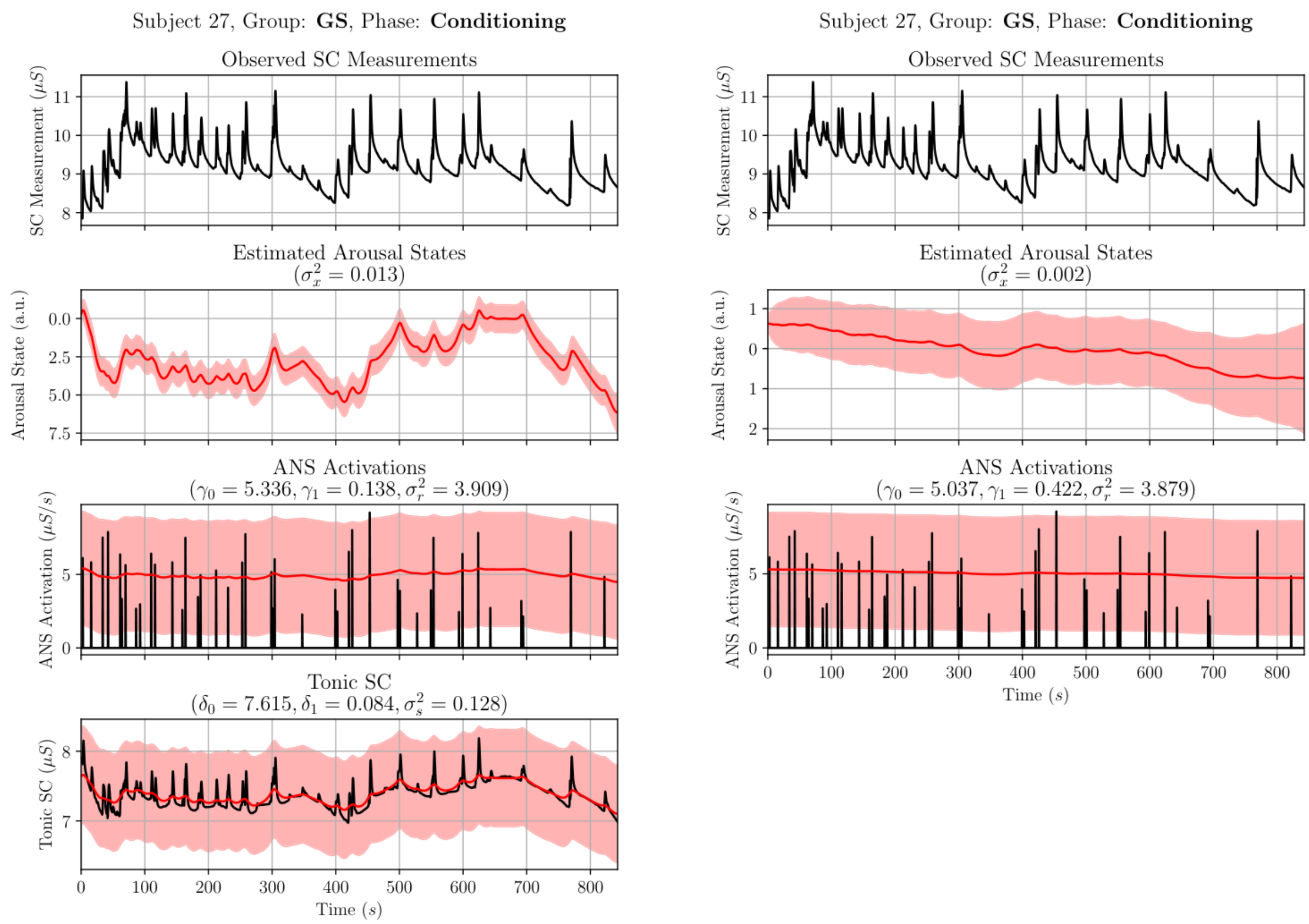


Figure BC: **Subject 27 estimated arousal states during the conditioning phase.** Shown for the MPP+Cont model (left) and the MPP model (right). The first panels show the observed SC measurements, $y$ (black). The second panels show the estimated arousal states, $x_{est}$ (red). The third panels show the ANS activations, $r_{obs}$ (black) and the estimated amplitudes, $r_{est}$ (red). The fourth panel shows the tonic component, $s_{obs}$ (black) and the estimated tonic component, $s_{est}$ (red). Estimates are shown with 95% confidence intervals (CI) (red shaded area). Estimated parameters are reported in the title of each panel.

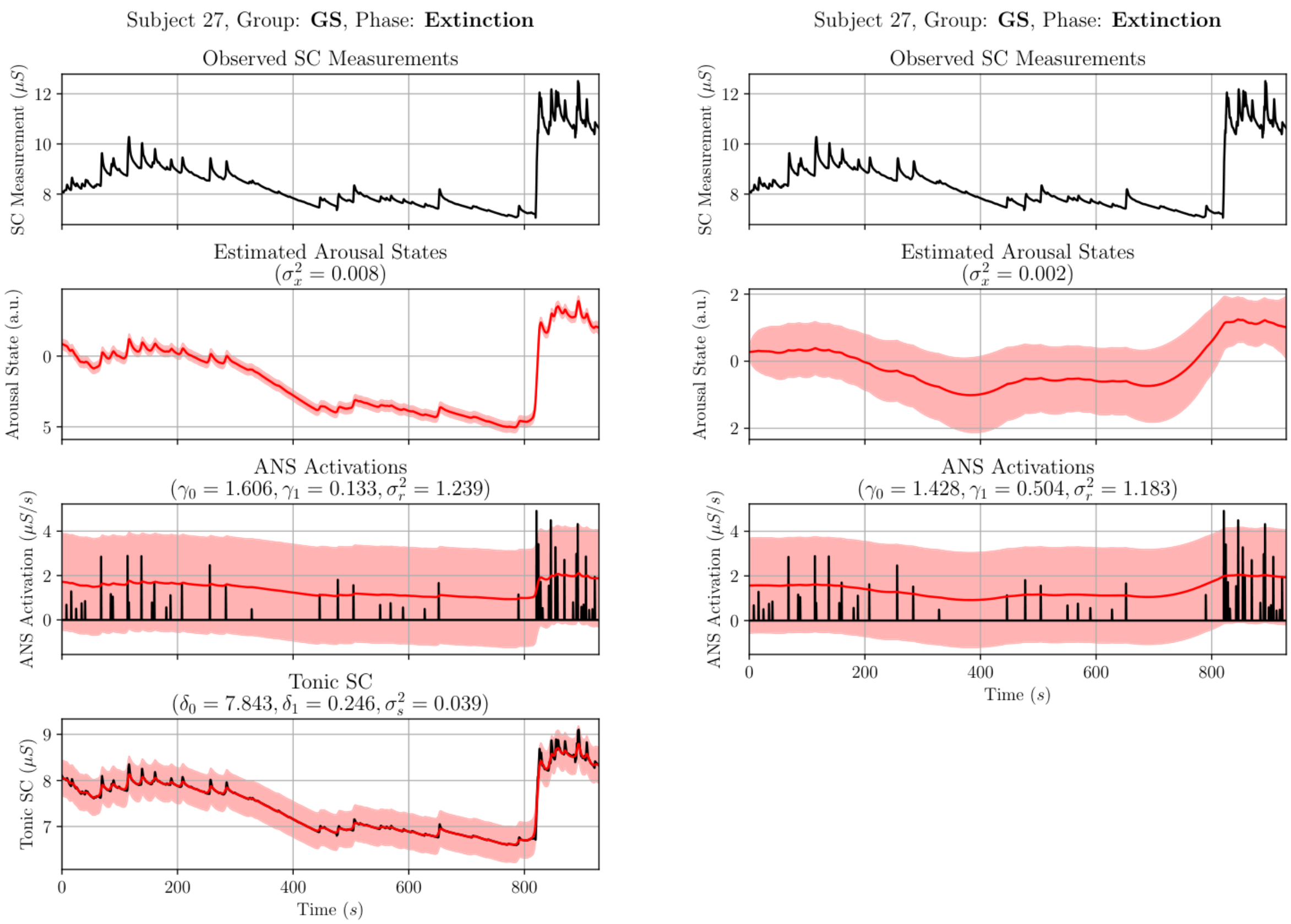


Figure BD: **Subject 27 estimated arousal states during the extinction phase.** Shown for the MPP+Cont model (left) and the MPP model (right). The first panels show the observed SC measurements, $y$ (black). The second panels show the estimated arousal states, $x_{est}$ (red). The third panels show the ANS activations, $r_{obs}$ (black) and the estimated amplitudes, $r_{est}$ (red). The fourth panel shows the tonic component, $s_{obs}$ (black) and the estimated tonic component, $s_{est}$ (red). Estimates are shown with 95% confidence intervals (CI) (red shaded area). Estimated parameters are reported in the title of each panel.

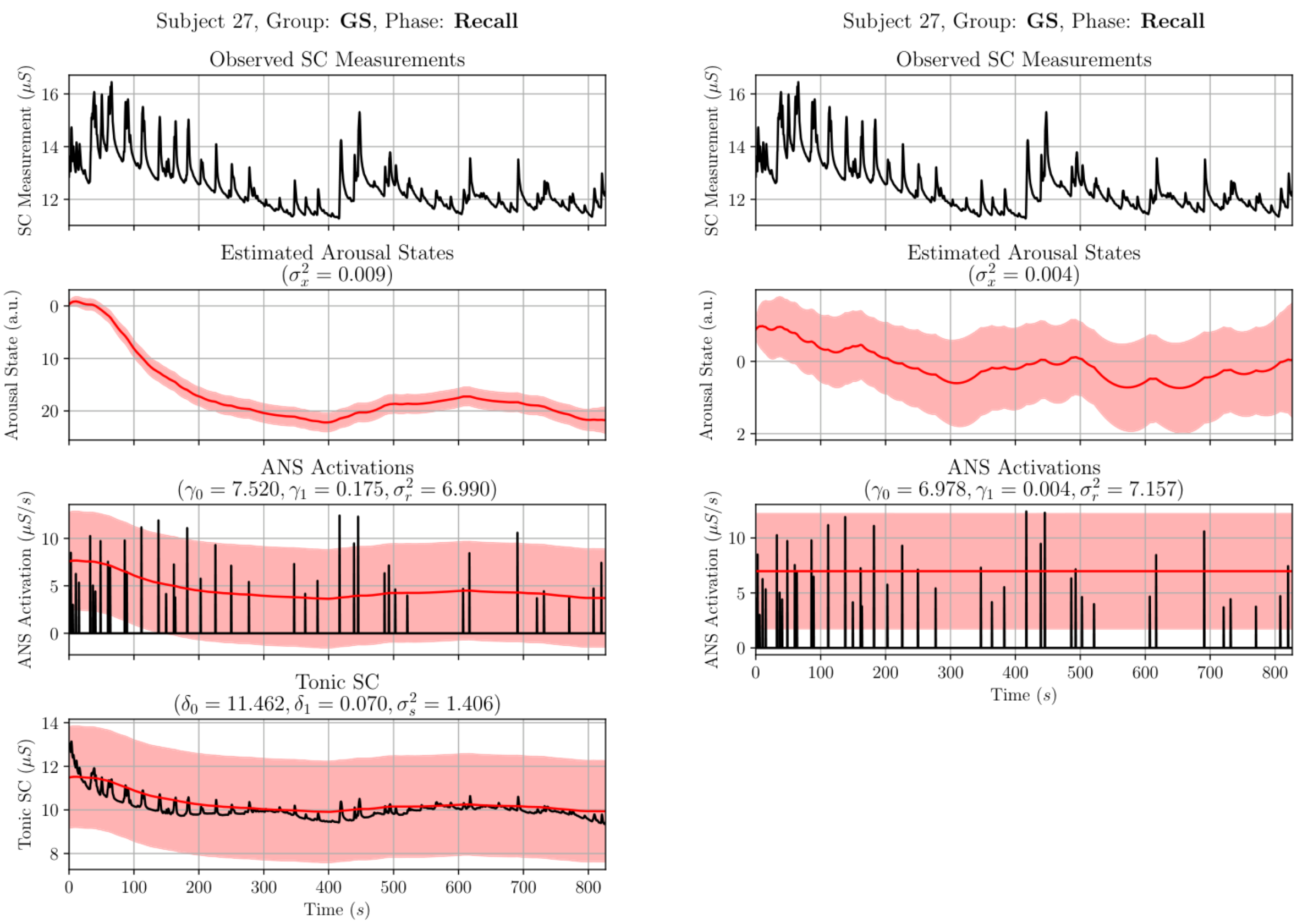


Figure BE: **Subject 27 estimated arousal states during the recall phase.** Shown for the MPP+Cont model (left) and the MPP model (right). The first panels show the observed SC measurements, $y$ (black). The second panels show the estimated arousal states, $x_{est}$ (red). The third panels show the ANS activations, $r_{obs}$ (black) and the estimated amplitudes, $r_{est}$ (red). The fourth panel shows the tonic component, $s_{obs}$ (black) and the estimated tonic component, $s_{est}$ (red). Estimates are shown with 95% confidence intervals (CI) (red shaded area). Estimated parameters are reported in the title of each panel.

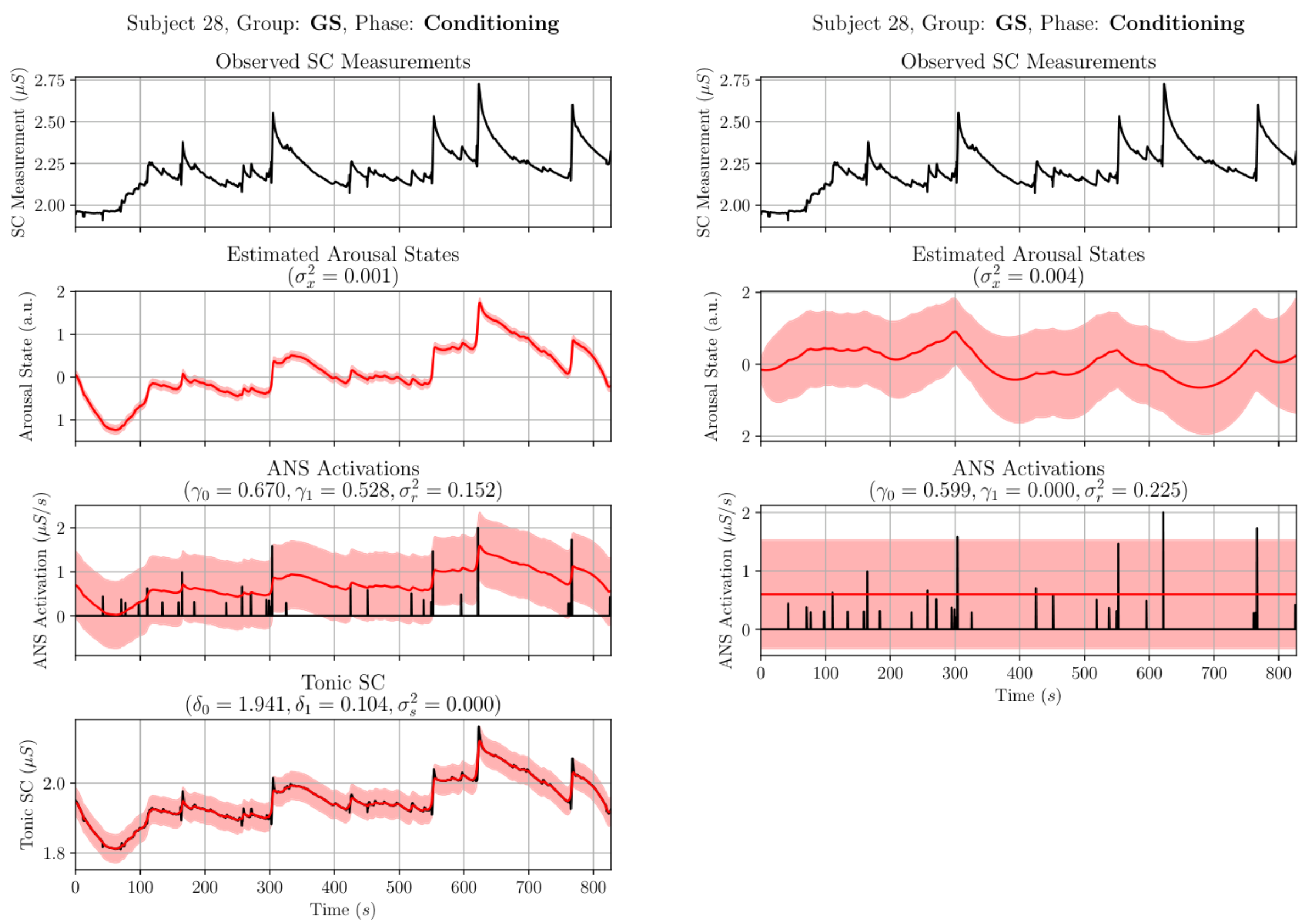


Figure BF: **Subject 28 estimated arousal states during the conditioning phase.** Shown for the MPP+Cont model (left) and the MPP model (right). The first panels show the observed SC measurements, $y$ (black). The second panels show the estimated arousal states, $x_{est}$ (red). The third panels show the ANS activations, $r_{obs}$ (black) and the estimated amplitudes, $r_{est}$ (red). The fourth panel shows the tonic component, $s_{obs}$ (black) and the estimated tonic component, $s_{est}$ (red). Estimates are shown with 95% confidence intervals (CI) (red shaded area). Estimated parameters are reported in the title of each panel.

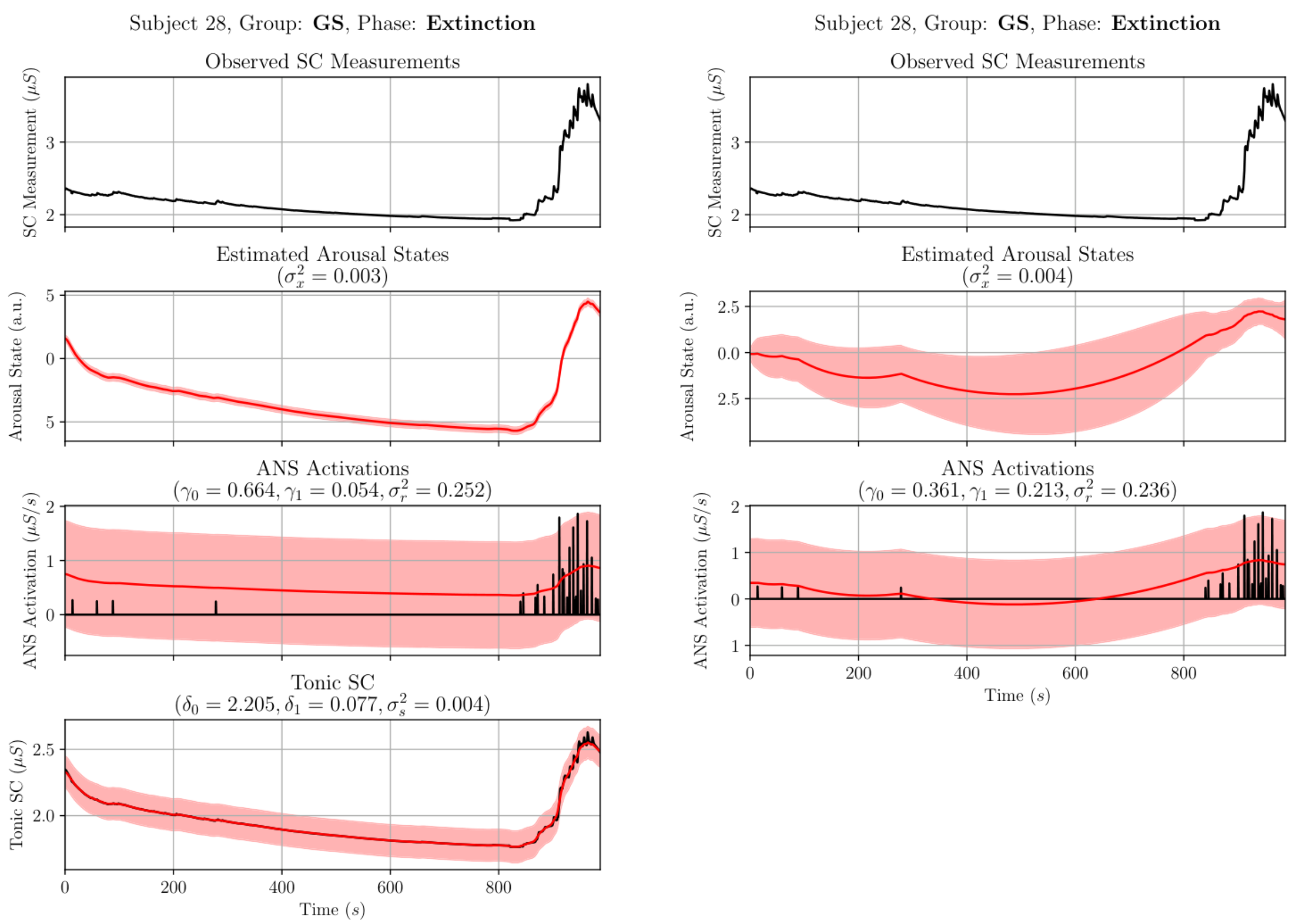


Figure BG: **Subject 28 estimated arousal states during the extinction phase.** Shown for the MPP+Cont model (left) and the MPP model (right). The first panels show the observed SC measurements, $y$ (black). The second panels show the estimated arousal states, $x_{est}$ (red). The third panels show the ANS activations, $r_{obs}$ (black) and the estimated amplitudes, $r_{est}$ (red). The fourth panel shows the tonic component, $s_{obs}$ (black) and the estimated tonic component, $s_{est}$ (red). Estimates are shown with 95% confidence intervals (CI) (red shaded area). Estimated parameters are reported in the title of each panel.

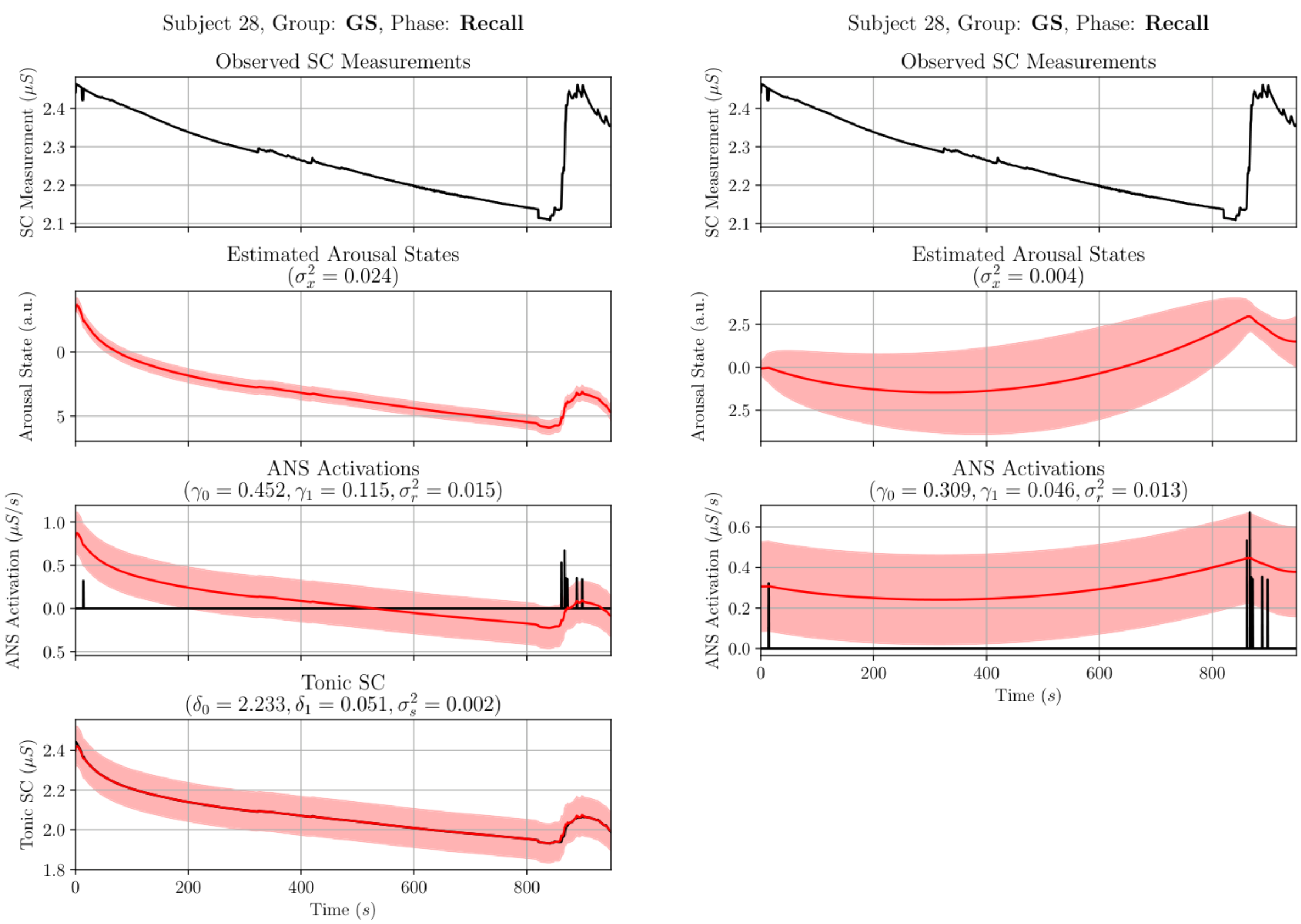


Figure BH: **Subject 28 estimated arousal states during the recall phase.** Shown for the MPP+Cont model (left) and the MPP model (right). The first panels show the observed SC measurements, $y$ (black). The second panels show the estimated arousal states, $x_{est}$ (red). The third panels show the ANS activations, $r_{obs}$ (black) and the estimated amplitudes, $r_{est}$ (red). The fourth panel shows the tonic component, $s_{obs}$ (black) and the estimated tonic component, $s_{est}$ (red). Estimates are shown with 95% confidence intervals (CI) (red shaded area). Estimated parameters are reported in the title of each panel.

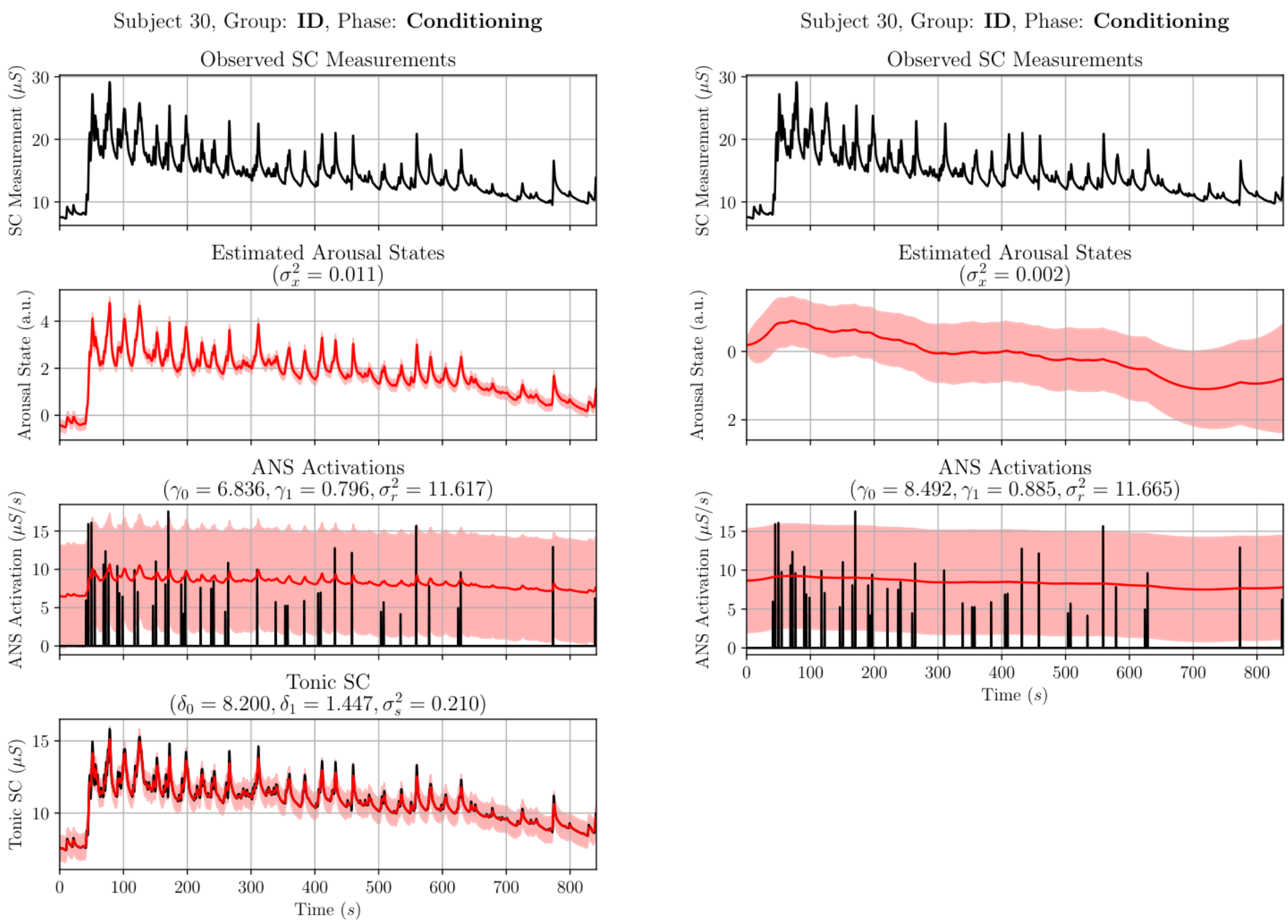


Figure BI: **Subject 30 estimated arousal states during the conditioning phase.** Shown for the MPP+Cont model (left) and the MPP model (right). The first panels show the observed SC measurements, $y$ (black). The second panels show the estimated arousal states, $x_{est}$ (red). The third panels show the ANS activations, $r_{obs}$ (black) and the estimated amplitudes, $r_{est}$ (red). The fourth panel shows the tonic component, $s_{obs}$ (black) and the estimated tonic component, $s_{est}$ (red). Estimates are shown with 95% confidence intervals (CI) (red shaded area). Estimated parameters are reported in the title of each panel.

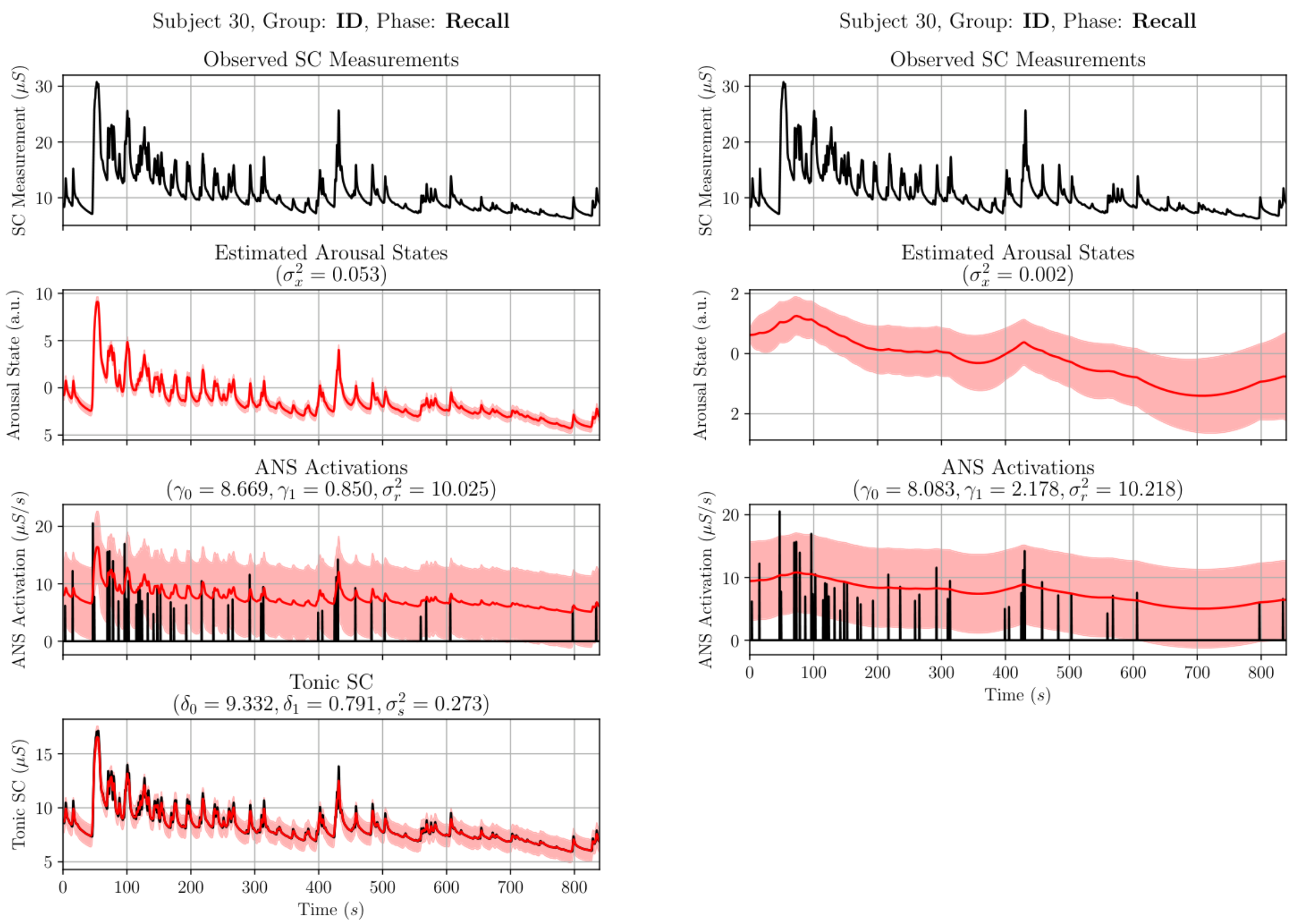


Figure BJ: **Subject 30 estimated arousal states during the recall phase.** Shown for the MPP+Cont model (left) and the MPP model (right). The first panels show the observed SC measurements, $y$ (black). The second panels show the estimated arousal states, $x_{est}$ (red). The third panels show the ANS activations, $r_{obs}$ (black) and the estimated amplitudes, $r_{est}$ (red). The fourth panel shows the tonic component, $s_{obs}$ (black) and the estimated tonic component, $s_{est}$ (red). Estimates are shown with 95% confidence intervals (CI) (red shaded area). Estimated parameters are reported in the title of each panel.

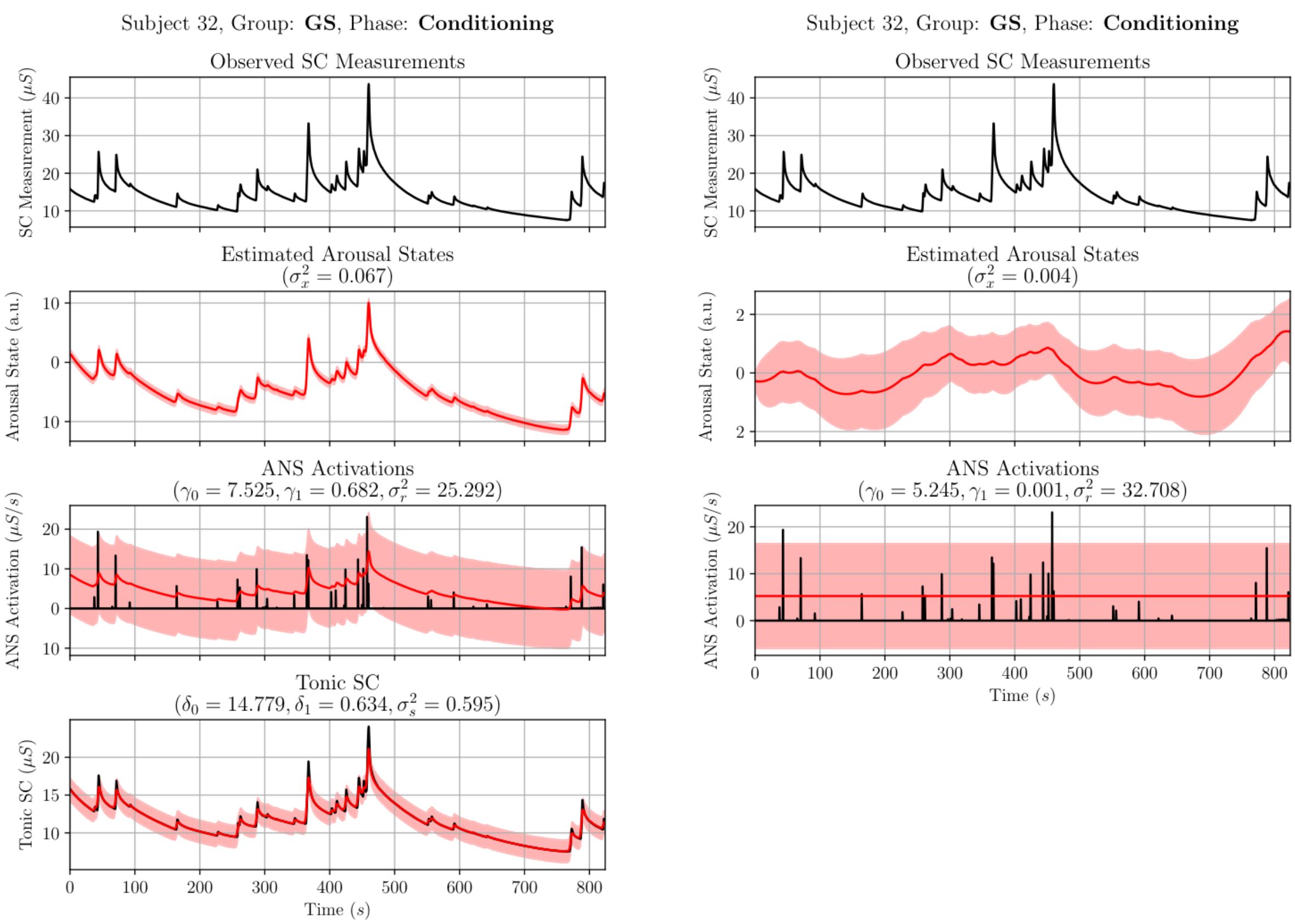


Figure BK: **Subject 32 estimated arousal states during the conditioning phase.** Shown for the MPP+Cont model (left) and the MPP model (right). The first panels show the observed SC measurements, $y$ (black). The second panels show the estimated arousal states, $x_{est}$ (red). The third panels show the ANS activations, $r_{obs}$ (black) and the estimated amplitudes, $r_{est}$ (red). The fourth panel shows the tonic component, $s_{obs}$ (black) and the estimated tonic component, $s_{est}$ (red). Estimates are shown with 95% confidence intervals (CI) (red shaded area). Estimated parameters are reported in the title of each panel.

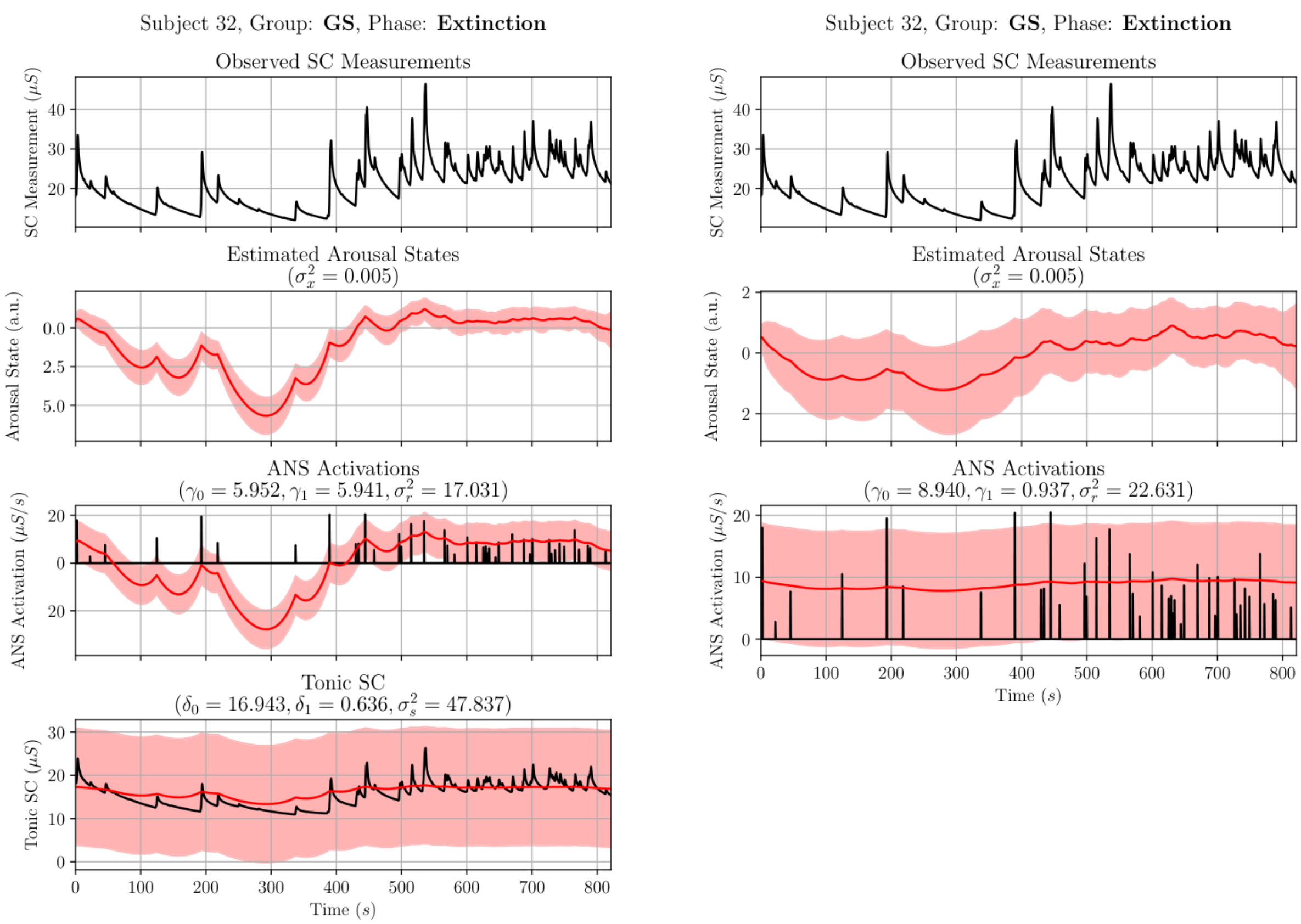


Figure BL: **Subject 32 estimated arousal states during the extinction phase.** Shown for the MPP+Cont model (left) and the MPP model (right). The first panels show the observed SC measurements, $y$ (black). The second panels show the estimated arousal states, $x_{est}$ (red). The third panels show the ANS activations, $r_{obs}$ (black) and the estimated amplitudes, $r_{est}$ (red). The fourth panel shows the tonic component, $s_{obs}$ (black) and the estimated tonic component, $s_{est}$ (red). Estimates are shown with 95% confidence intervals (CI) (red shaded area). Estimated parameters are reported in the title of each panel.

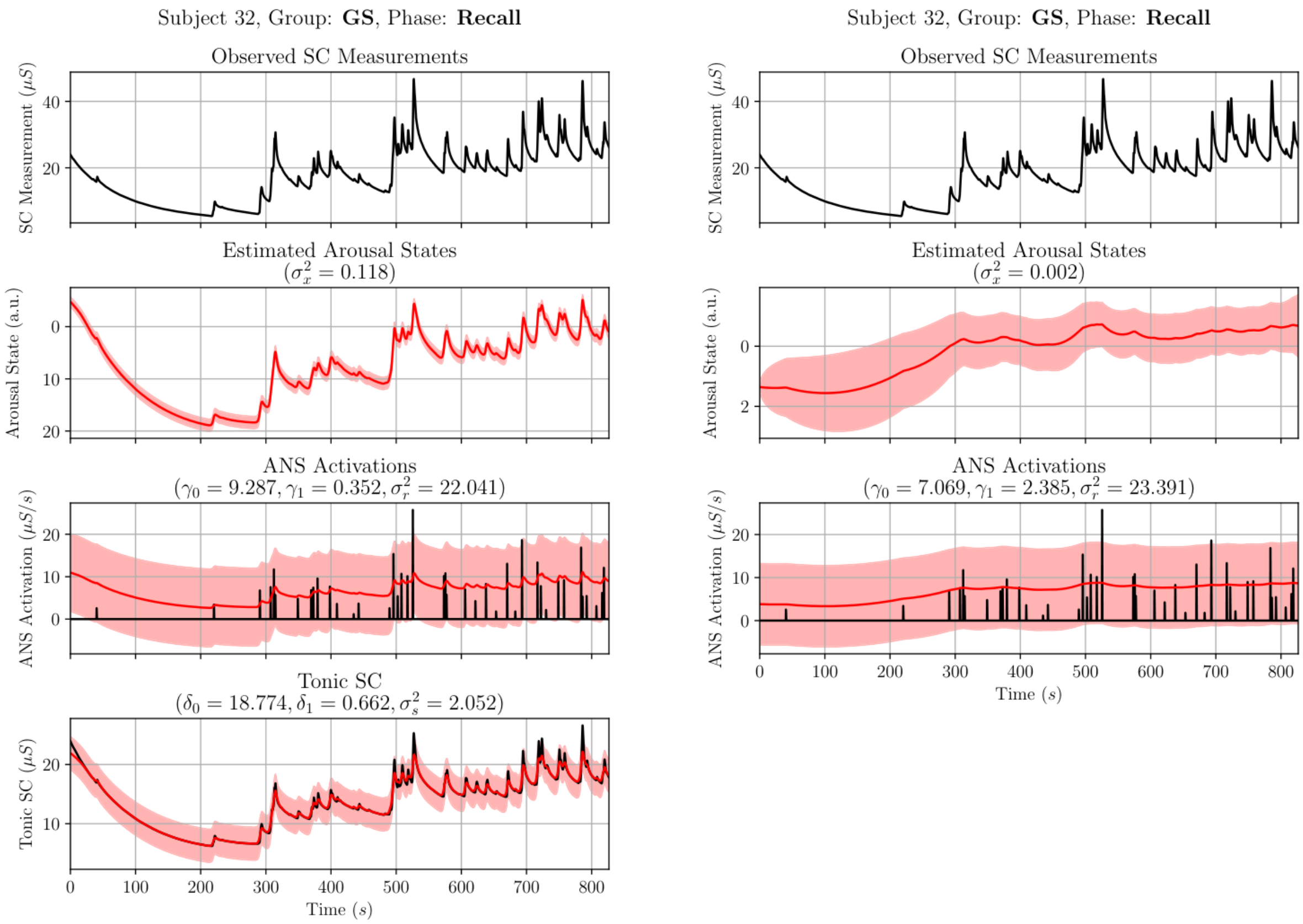


Figure BM: **Subject 32 estimated arousal states during the recall phase.** Shown for the MPP+Cont model (left) and the MPP model (right). The first panels show the observed SC measurements, $y$ (black). The second panels show the estimated arousal states, $x_{est}$ (red). The third panels show the ANS activations, $r_{obs}$ (black) and the estimated amplitudes, $r_{est}$ (red). The fourth panel shows the tonic component, $s_{obs}$ (black) and the estimated tonic component, $s_{est}$ (red). Estimates are shown with 95% confidence intervals (CI) (red shaded area). Estimated parameters are reported in the title of each panel.

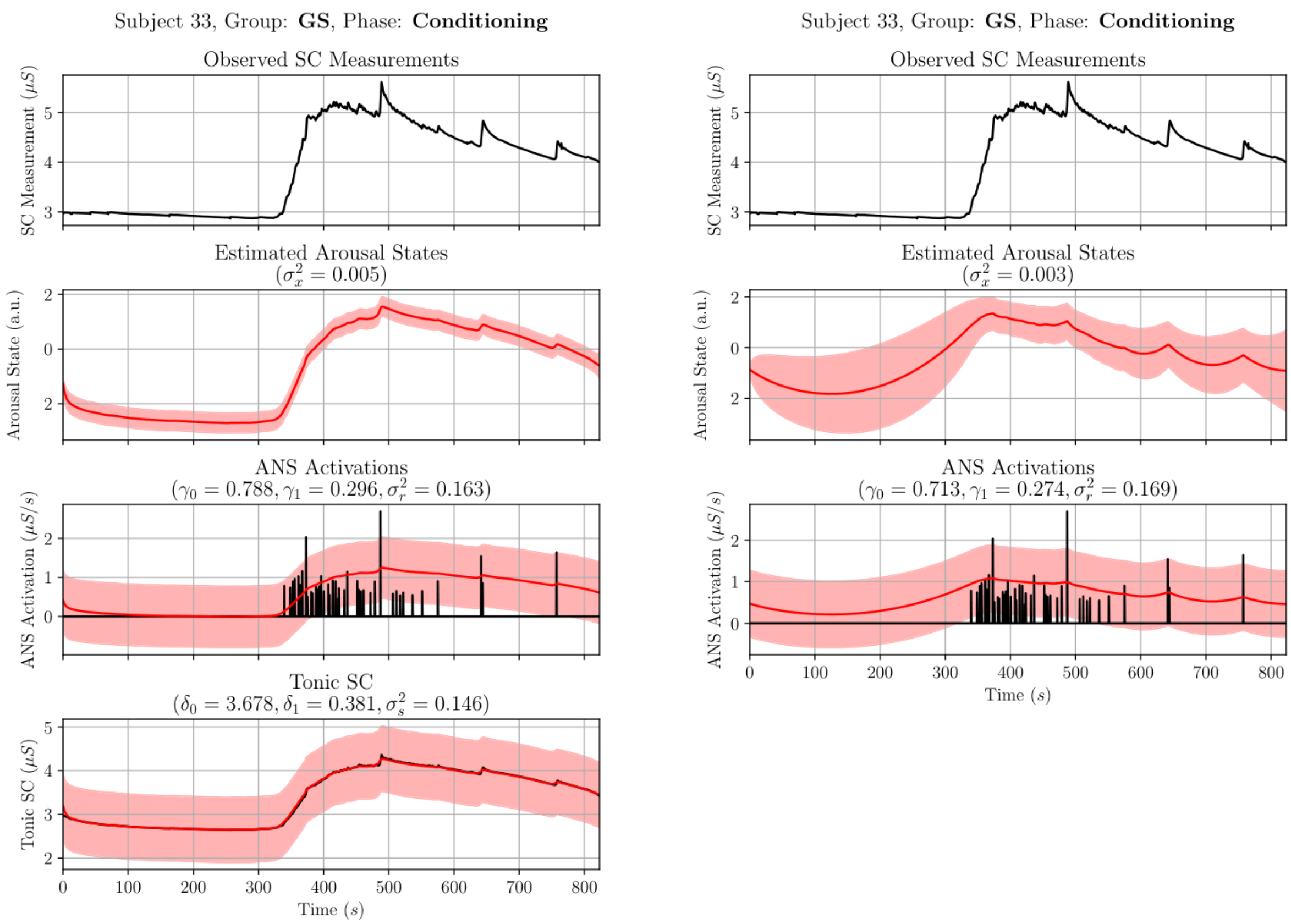


Figure BN: **Subject 33 estimated arousal states during the conditioning phase.** Shown for the MPP+Cont model (left) and the MPP model (right). The first panels show the observed SC measurements, $y$ (black). The second panels show the estimated arousal states, $x_{est}$ (red). The third panels show the ANS activations, $r_{obs}$ (black) and the estimated amplitudes, $r_{est}$ (red). The fourth panel shows the tonic component, $s_{obs}$ (black) and the estimated tonic component, $s_{est}$ (red). Estimates are shown with 95% confidence intervals (CI) (red shaded area). Estimated parameters are reported in the title of each panel.

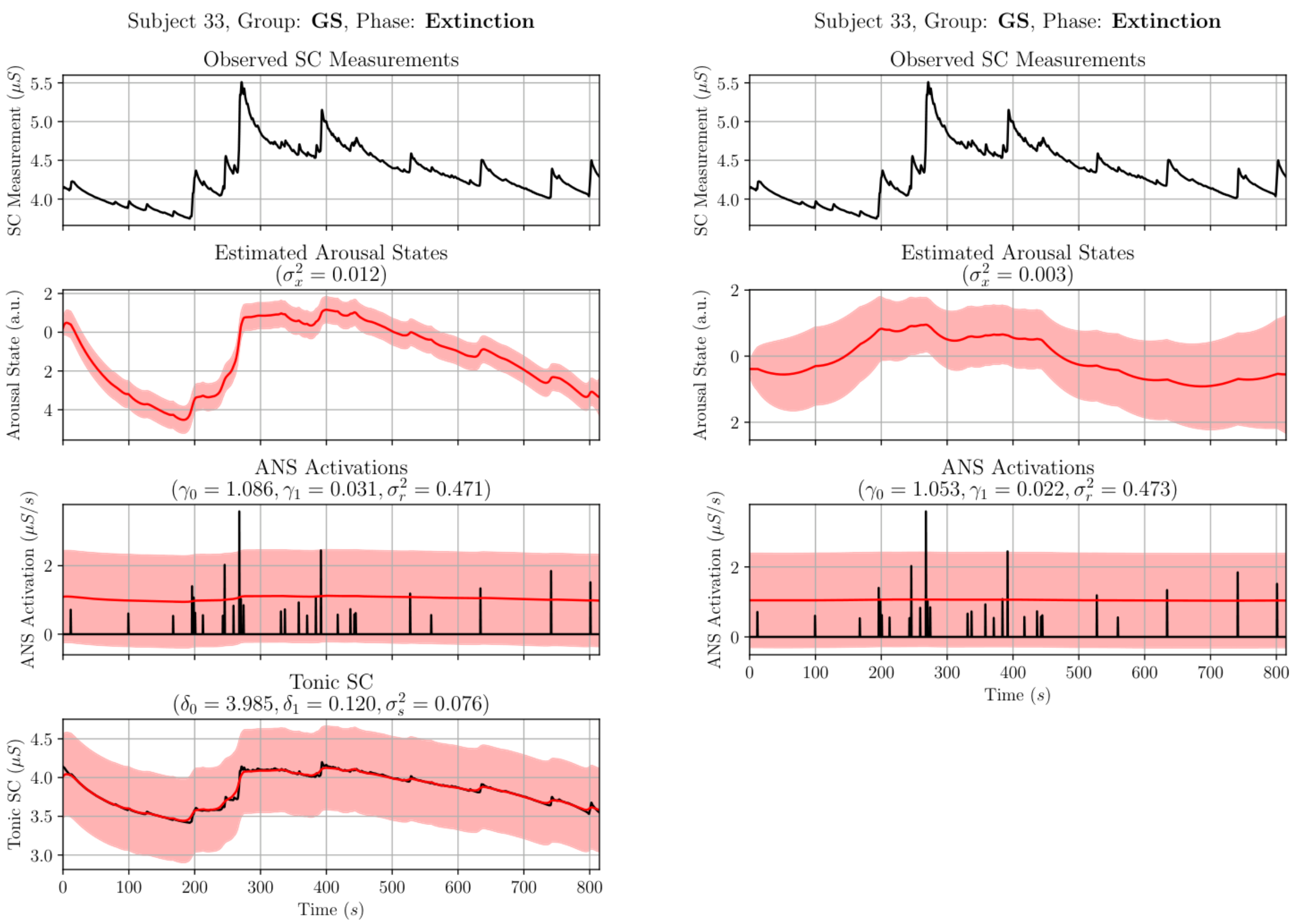


Figure BO: **Subject 33 estimated arousal states during the extinction phase.** Shown for the MPP+Cont model (left) and the MPP model (right). The first panels show the observed SC measurements, $y$ (black). The second panels show the estimated arousal states, $x_{est}$ (red). The third panels show the ANS activations, $r_{obs}$ (black) and the estimated amplitudes, $r_{est}$ (red). The fourth panel shows the tonic component, $s_{obs}$ (black) and the estimated tonic component, $s_{est}$ (red). Estimates are shown with 95% confidence intervals (CI) (red shaded area). Estimated parameters are reported in the title of each panel.

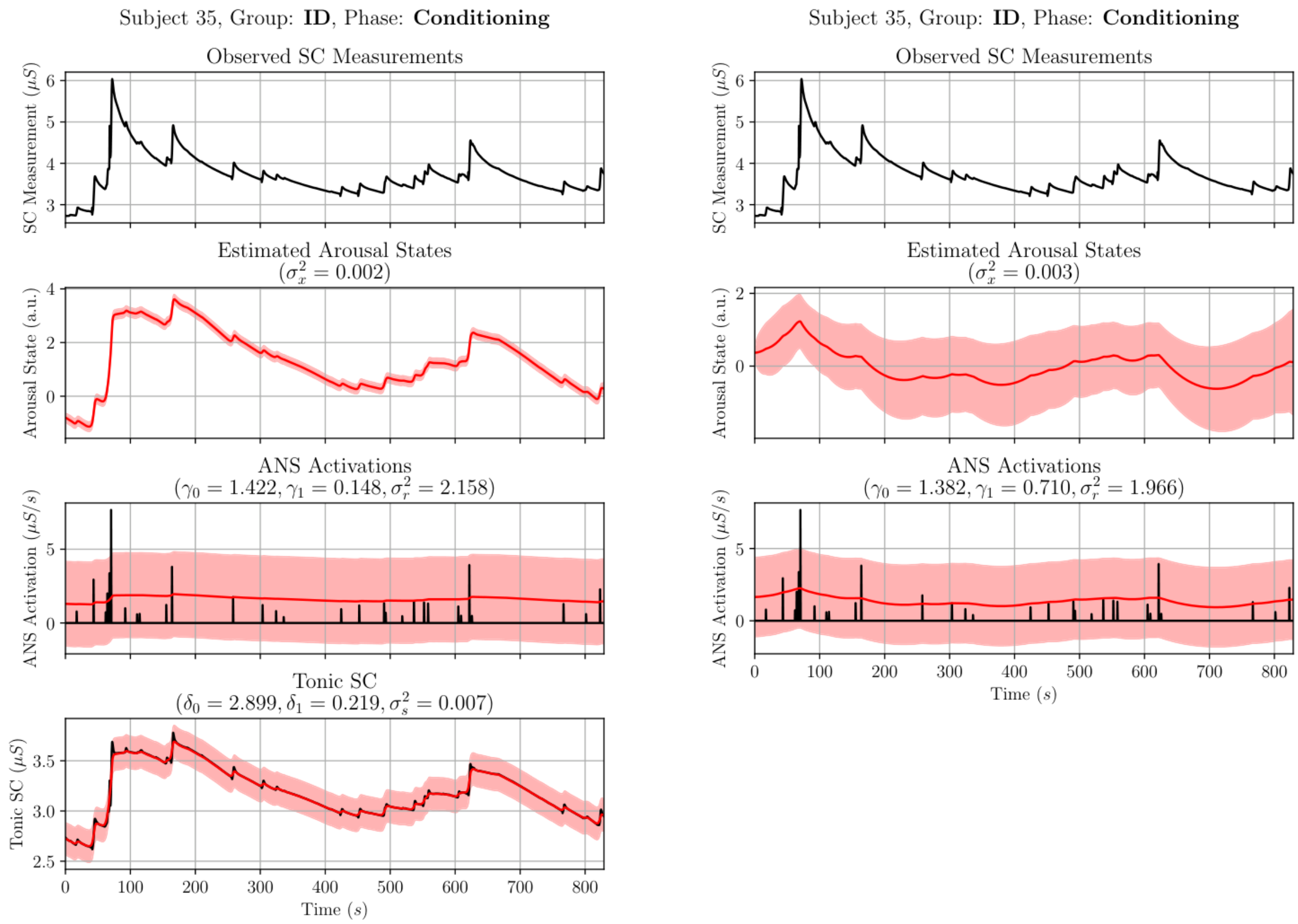


Figure BP: **Subject 35 estimated arousal states during the conditioning phase.** Shown for the MPP+Cont model (left) and the MPP model (right). The first panels show the observed SC measurements, $y$ (black). The second panels show the estimated arousal states, $x_{est}$ (red). The third panels show the ANS activations, $r_{obs}$ (black) and the estimated amplitudes, $r_{est}$ (red). The fourth panel shows the tonic component, $s_{obs}$ (black) and the estimated tonic component, $s_{est}$ (red). Estimates are shown with 95% confidence intervals (CI) (red shaded area). Estimated parameters are reported in the title of each panel.

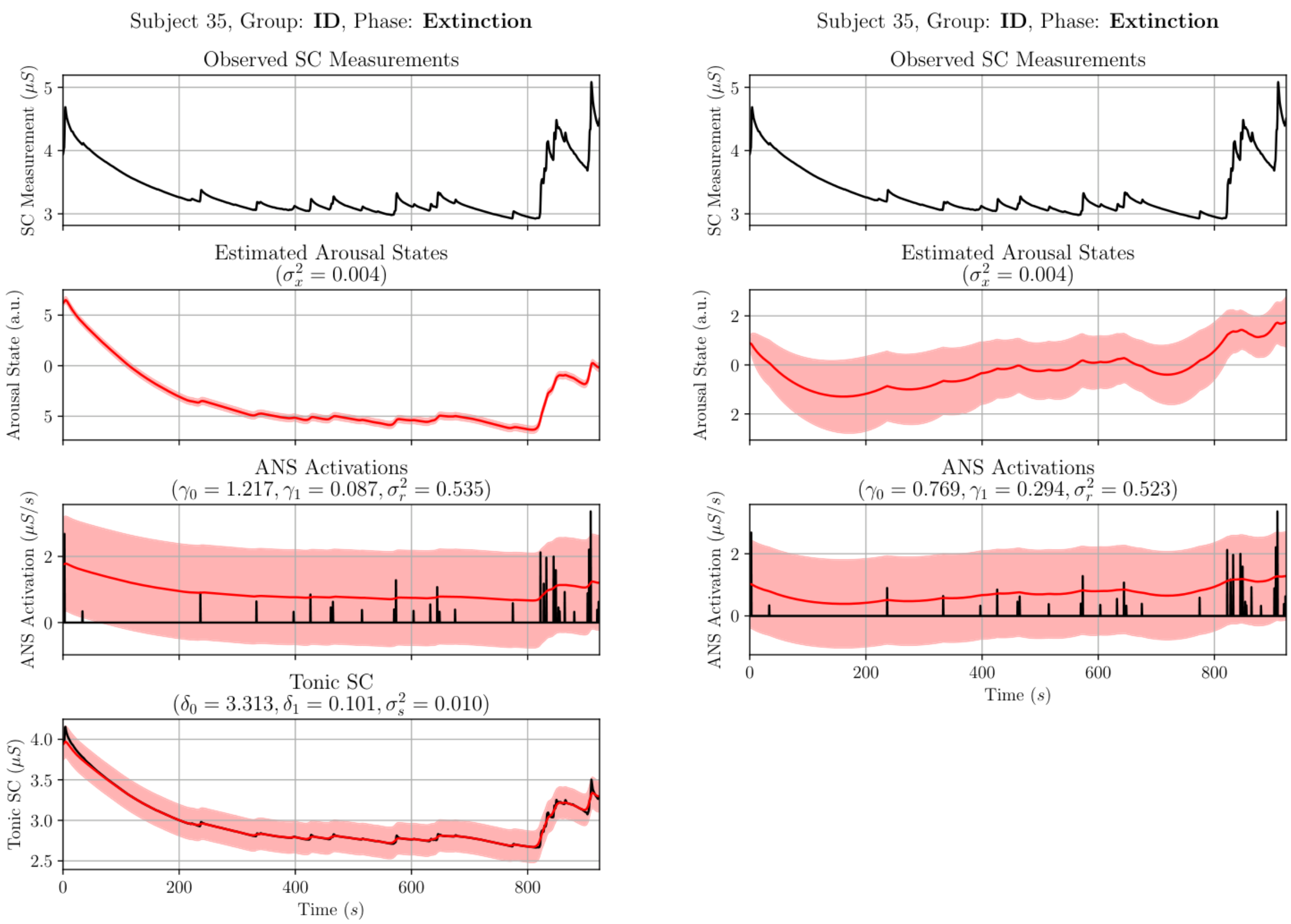


Figure BQ: **Subject 35 estimated arousal states during the extinction phase.** Shown for the MPP+Cont model (left) and the MPP model (right). The first panels show the observed SC measurements, $y$ (black). The second panels show the estimated arousal states, $x_{est}$ (red). The third panels show the ANS activations, $r_{obs}$ (black) and the estimated amplitudes, $r_{est}$ (red). The fourth panel shows the tonic component, $s_{obs}$ (black) and the estimated tonic component, $s_{est}$ (red). Estimates are shown with 95% confidence intervals (CI) (red shaded area). Estimated parameters are reported in the title of each panel.

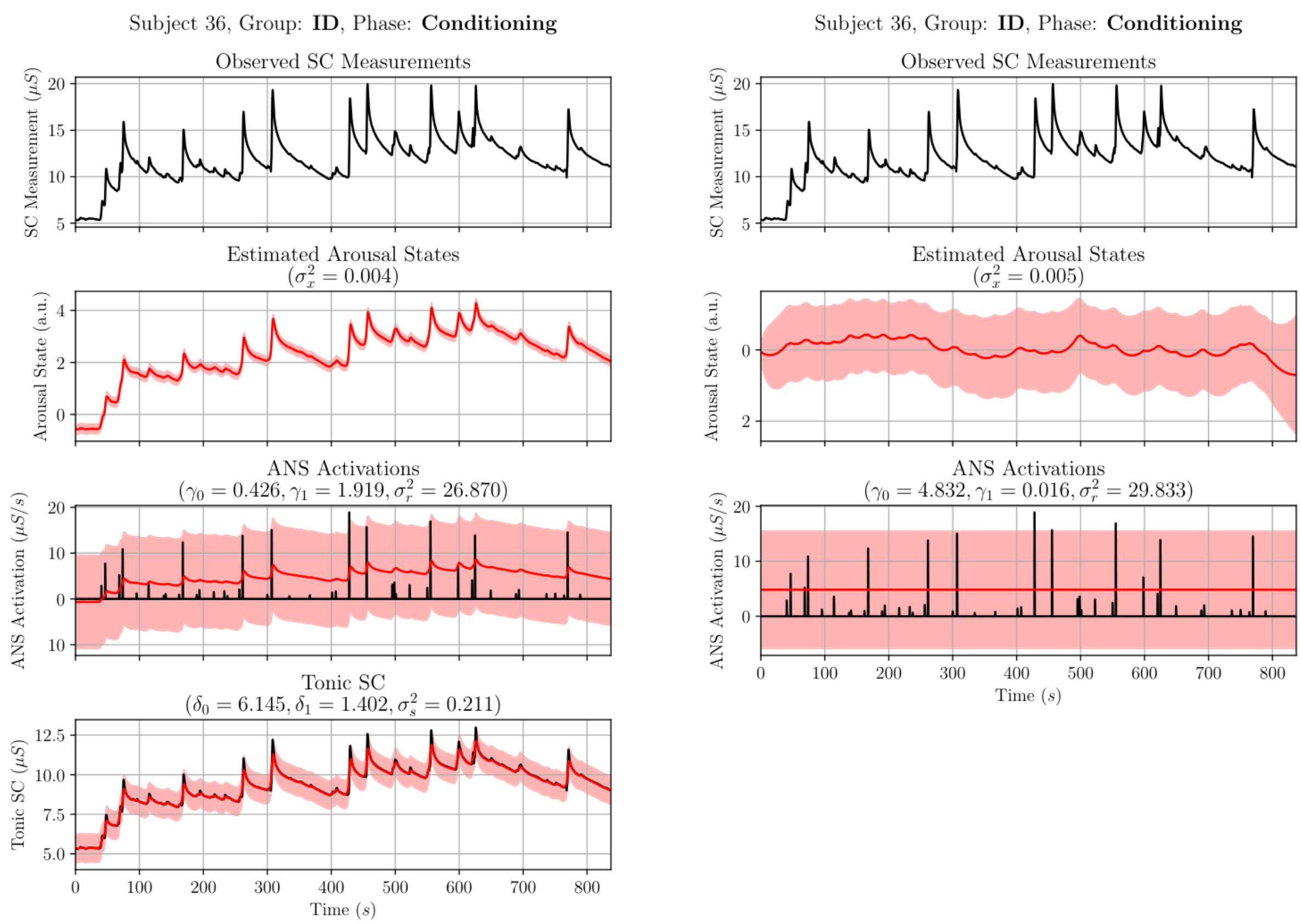


Figure BR: **Subject 36 estimated arousal states during the conditioning phase.** Shown for the MPP+Cont model (left) and the MPP model (right). The first panels show the observed SC measurements, $y$ (black). The second panels show the estimated arousal states, $x_{est}$ (red). The third panels show the ANS activations, $r_{obs}$ (black) and the estimated amplitudes, $r_{est}$ (red). The fourth panel shows the tonic component, $s_{obs}$ (black) and the estimated tonic component, $s_{est}$ (red). Estimates are shown with 95% confidence intervals (CI) (red shaded area). Estimated parameters are reported in the title of each panel.

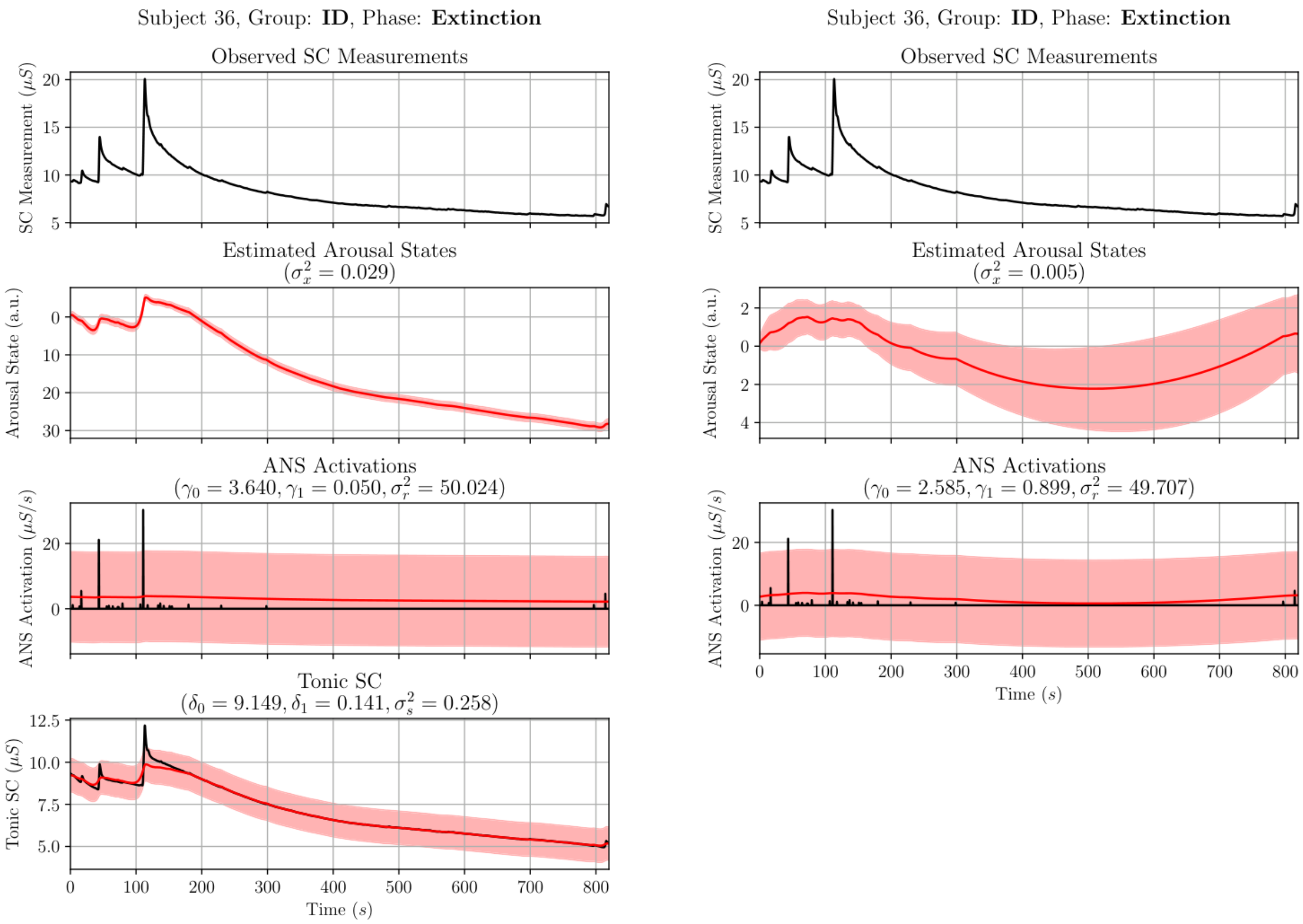


Figure BS: **Subject 36 estimated arousal states during the extinction phase.** Shown for the MPP+Cont model (left) and the MPP model (right). The first panels show the observed SC measurements, $y$ (black). The second panels show the estimated arousal states, $x_{est}$ (red). The third panels show the ANS activations, $r_{obs}$ (black) and the estimated amplitudes, $r_{est}$ (red). The fourth panel shows the tonic component, $s_{obs}$ (black) and the estimated tonic component, $s_{est}$ (red). Estimates are shown with 95% confidence intervals (CI) (red shaded area). Estimated parameters are reported in the title of each panel.

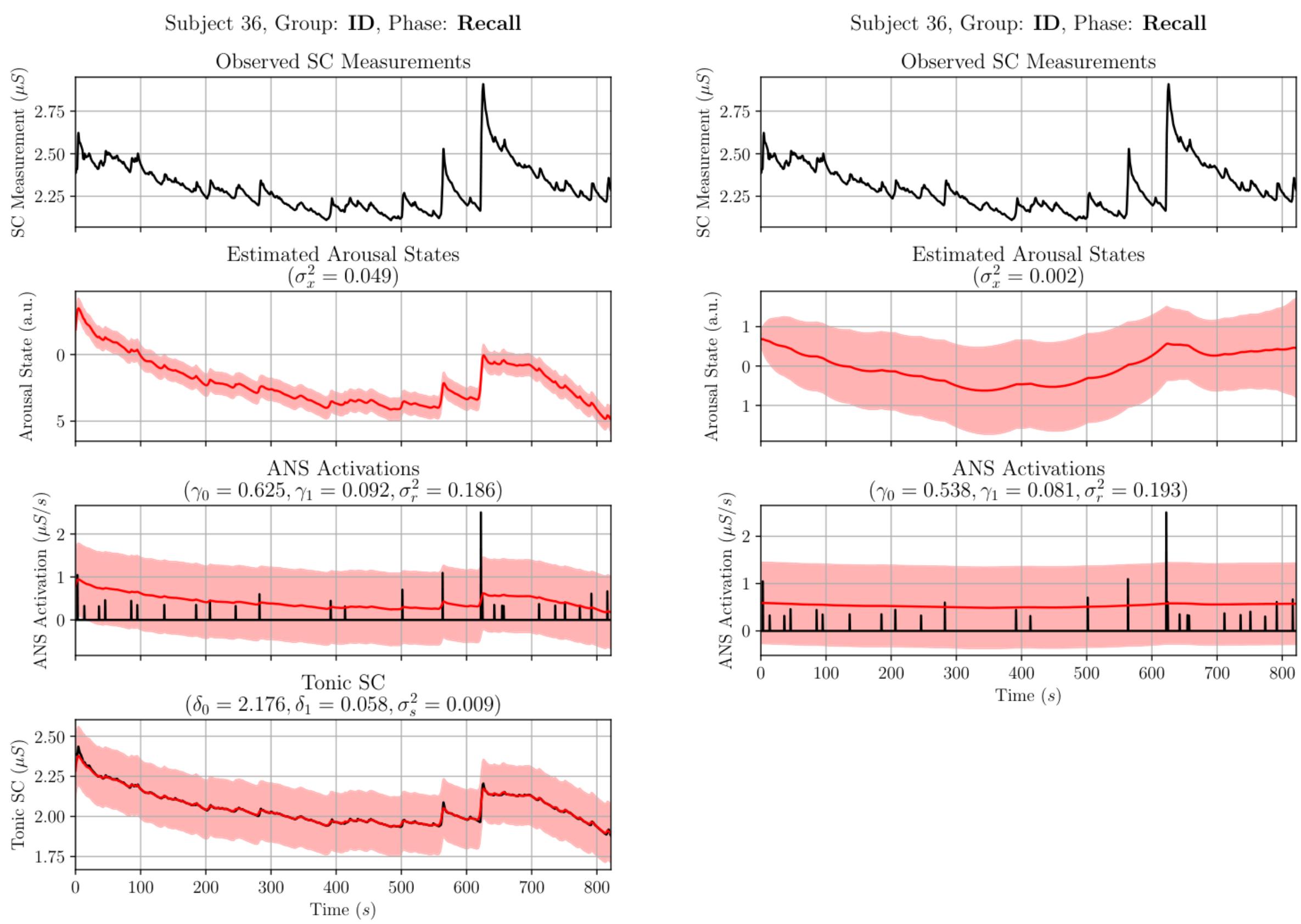


Figure BT: **Subject 36 estimated arousal states during the recall phase.** Shown for the MPP+Cont model (left) and the MPP model (right). The first panels show the observed SC measurements, $y$ (black). The second panels show the estimated arousal states, $x_{est}$ (red). The third panels show the ANS activations, $r_{obs}$ (black) and the estimated amplitudes, $r_{est}$ (red). The fourth panel shows the tonic component, $s_{obs}$ (black) and the estimated tonic component, $s_{est}$ (red). Estimates are shown with 95% confidence intervals (CI) (red shaded area). Estimated parameters are reported in the title of each panel.

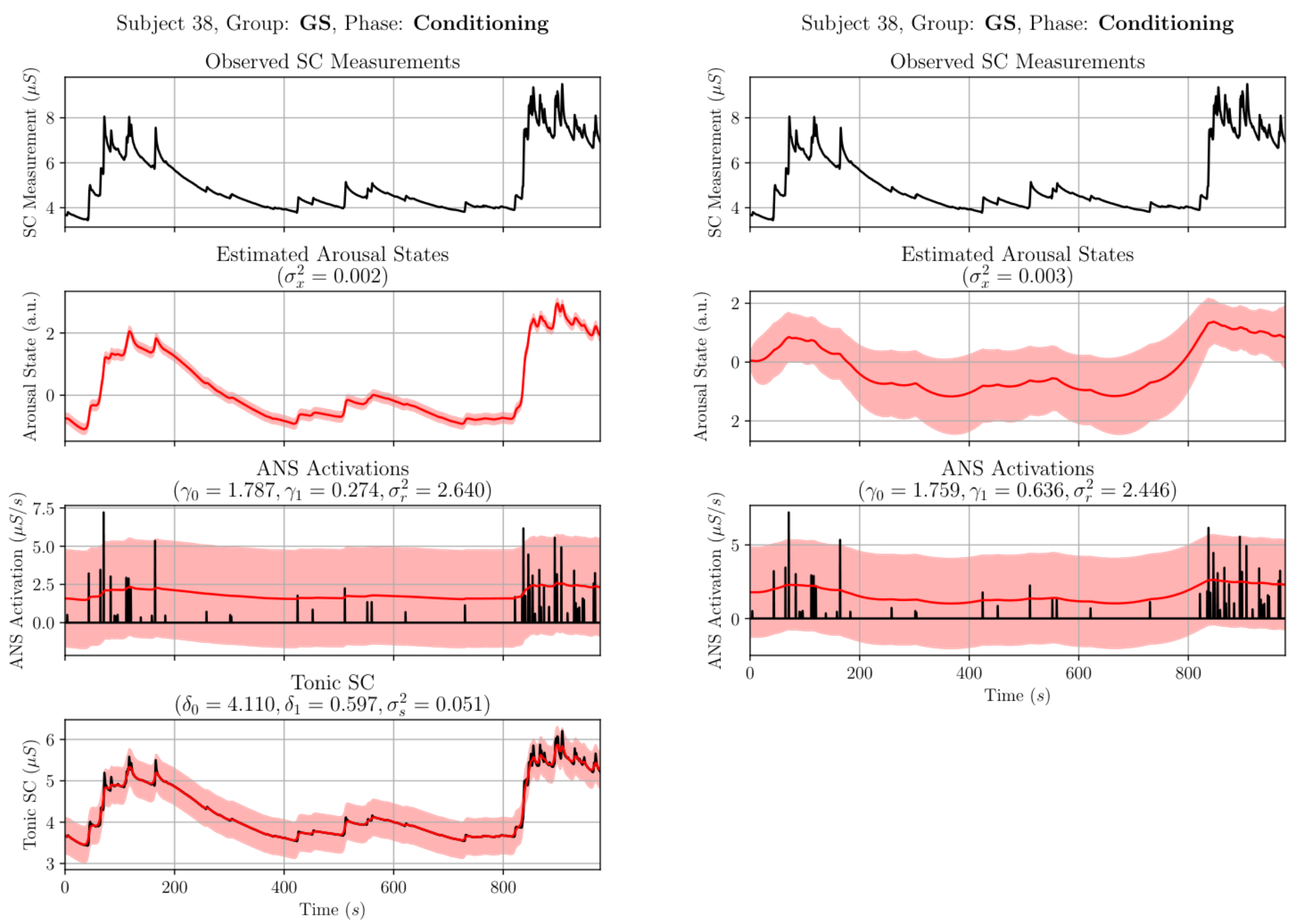


Figure BU: **Subject 38 estimated arousal states during the conditioning phase.** Shown for the MPP+Cont model (left) and the MPP model (right). The first panels show the observed SC measurements, $y$ (black). The second panels show the estimated arousal states, $x_{est}$ (red). The third panels show the ANS activations, $r_{obs}$ (black) and the estimated amplitudes, $r_{est}$ (red). The fourth panel shows the tonic component, $s_{obs}$ (black) and the estimated tonic component, $s_{est}$ (red). Estimates are shown with 95% confidence intervals (CI) (red shaded area). Estimated parameters are reported in the title of each panel.

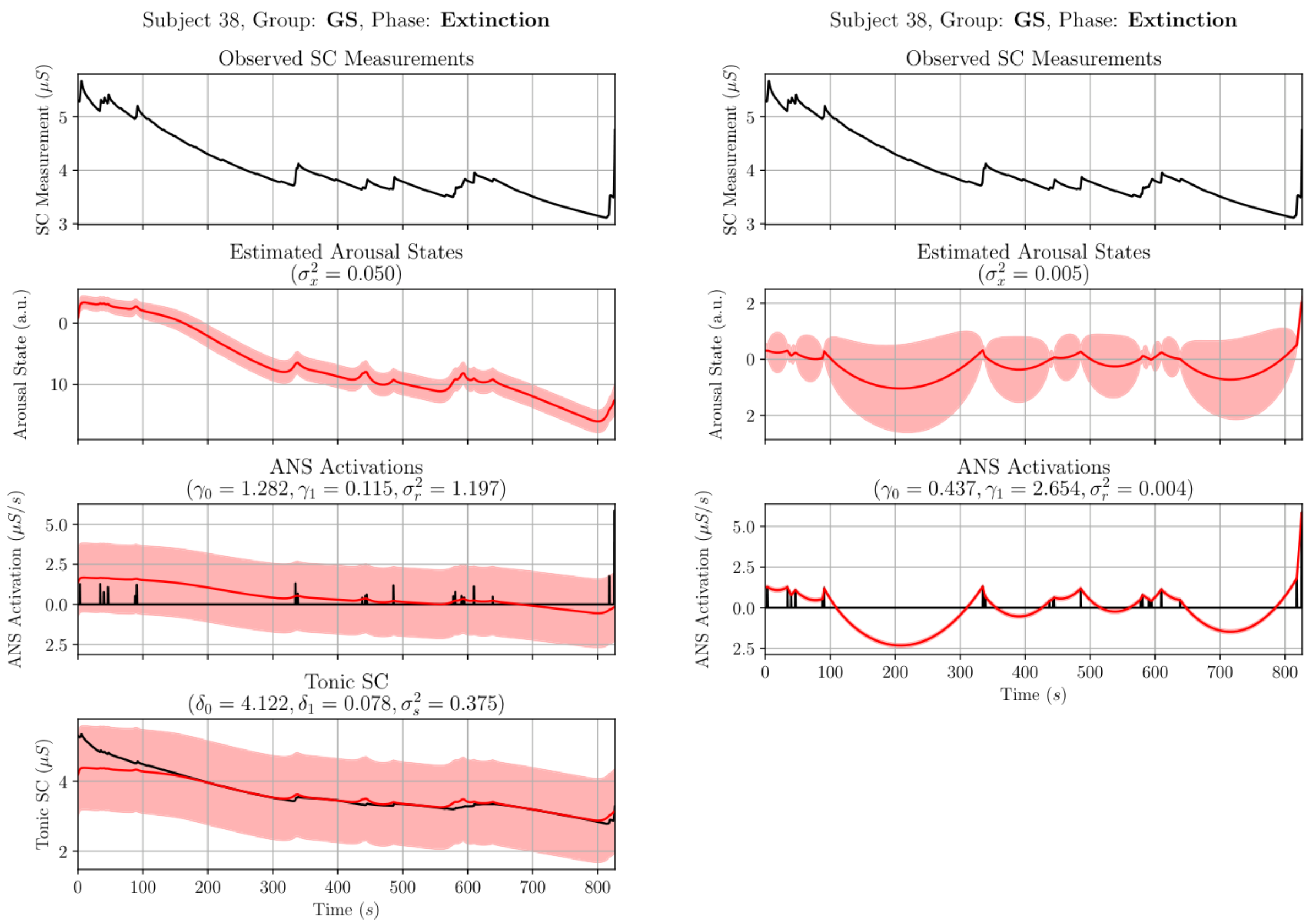


Figure BV: **Subject 38 estimated arousal states during the extinction phase.** Shown for the MPP+Cont model (left) and the MPP model (right). The first panels show the observed SC measurements, $y$ (black). The second panels show the estimated arousal states, $x_{est}$ (red). The third panels show the ANS activations, $r_{obs}$ (black) and the estimated amplitudes, $r_{est}$ (red). The fourth panel shows the tonic component, $s_{obs}$ (black) and the estimated tonic component, $s_{est}$ (red). Estimates are shown with 95% confidence intervals (CI) (red shaded area). Estimated parameters are reported in the title of each panel.

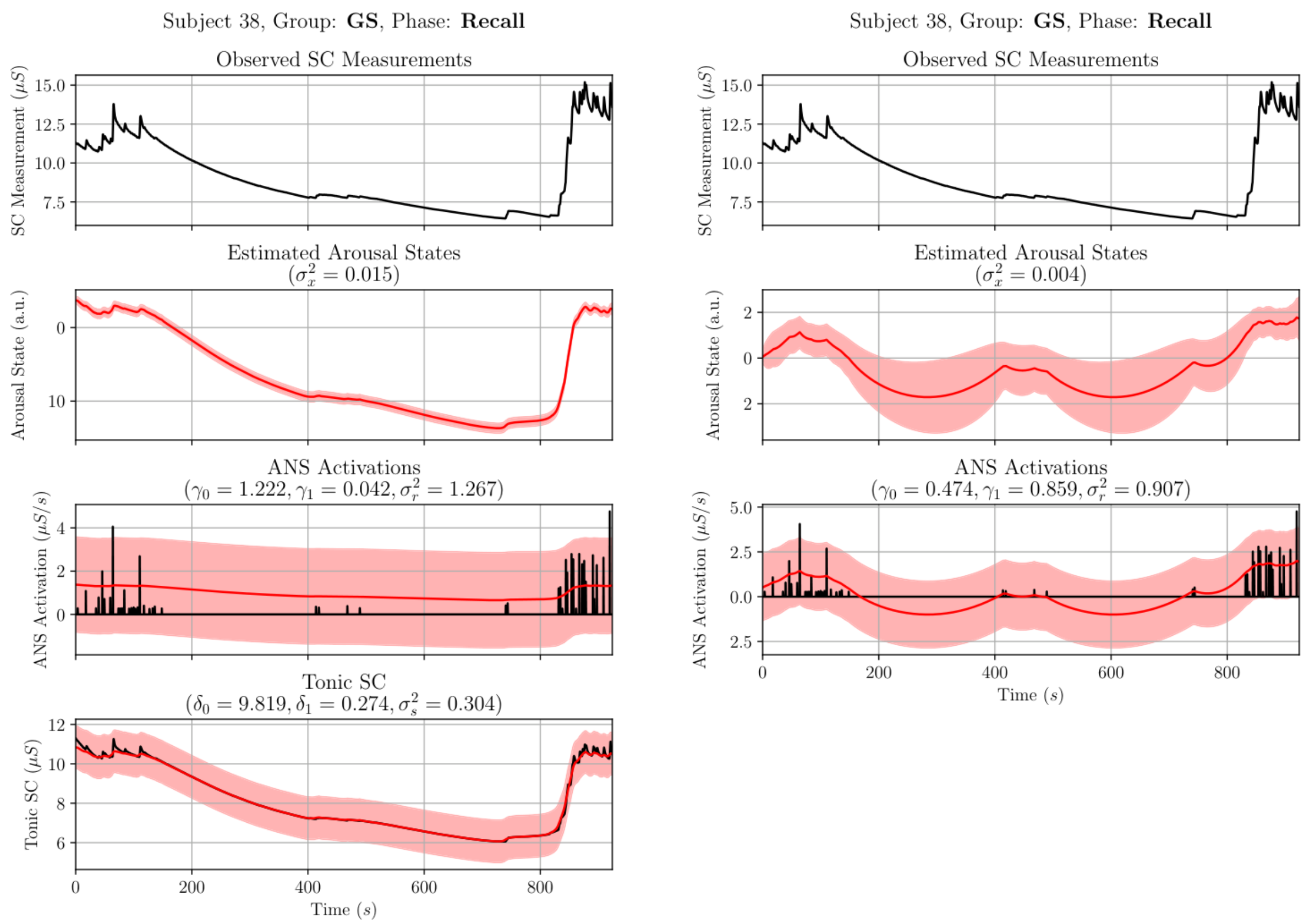


Figure BW: **Subject 38 estimated arousal states during the recall phase.** Shown for the MPP+Cont model (left) and the MPP model (right). The first panels show the observed SC measurements, $y$ (black). The second panels show the estimated arousal states, $x_{est}$ (red). The third panels show the ANS activations, $r_{obs}$ (black) and the estimated amplitudes, $r_{est}$ (red). The fourth panel shows the tonic component, $s_{obs}$ (black) and the estimated tonic component, $s_{est}$ (red). Estimates are shown with 95% confidence intervals (CI) (red shaded area). Estimated parameters are reported in the title of each panel.

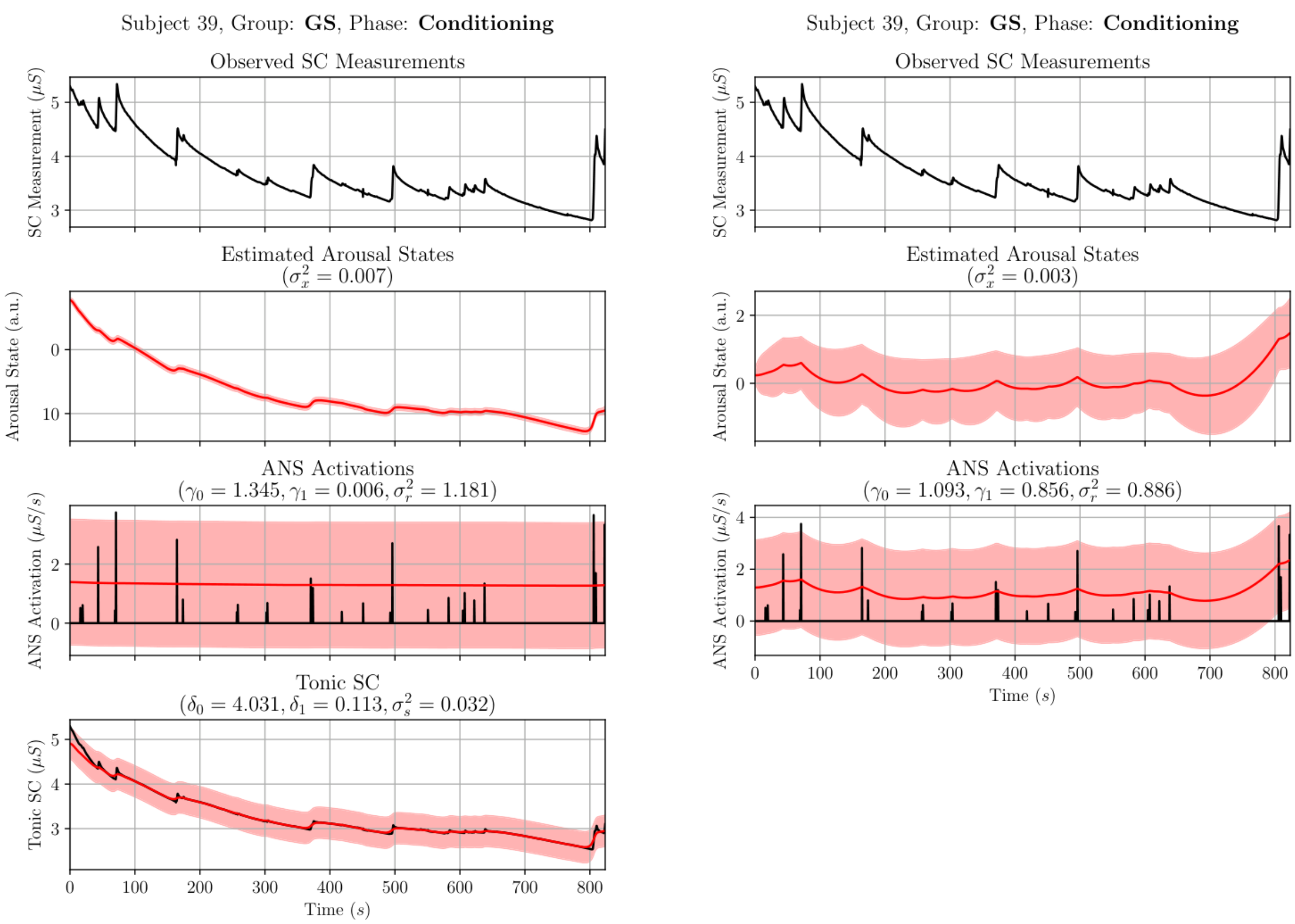


Figure BX: **Subject 39 estimated arousal states during the conditioning phase.** Shown for the MPP+Cont model (left) and the MPP model (right). The first panels show the observed SC measurements, $y$ (black). The second panels show the estimated arousal states, $x_{est}$ (red). The third panels show the ANS activations, $r_{obs}$ (black) and the estimated amplitudes, $r_{est}$ (red). The fourth panel shows the tonic component, $s_{obs}$ (black) and the estimated tonic component, $s_{est}$ (red). Estimates are shown with 95% confidence intervals (CI) (red shaded area). Estimated parameters are reported in the title of each panel.

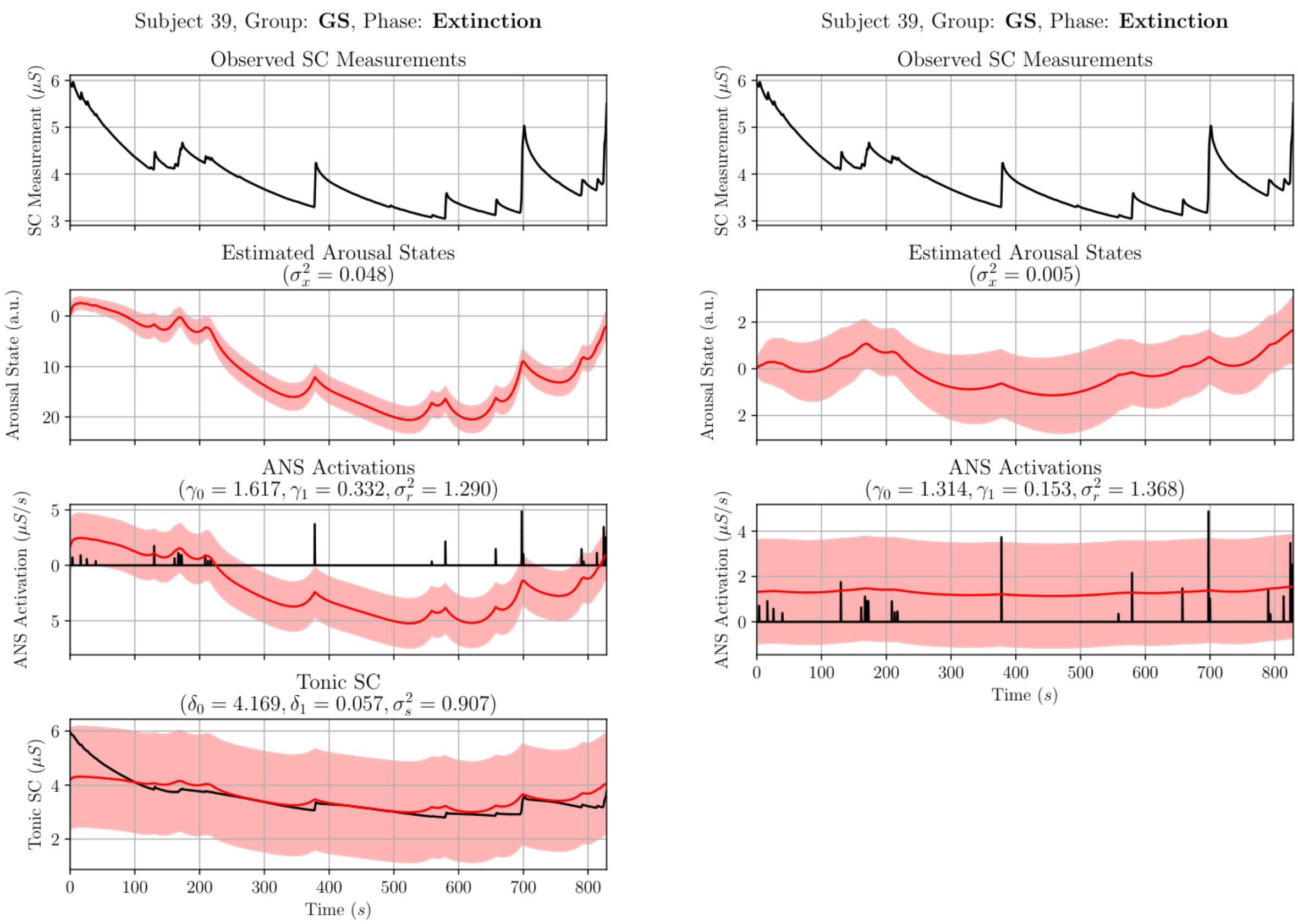


Figure BY: **Subject 39 estimated arousal states during the extinction phase.** Shown for the MPP+Cont model (left) and the MPP model (right). The first panels show the observed SC measurements, $y$ (black). The second panels show the estimated arousal states, $x_{est}$ (red). The third panels show the ANS activations, $r_{obs}$ (black) and the estimated amplitudes, $r_{est}$ (red). The fourth panel shows the tonic component, $s_{obs}$ (black) and the estimated tonic component, $s_{est}$ (red). Estimates are shown with 95% confidence intervals (CI) (red shaded area). Estimated parameters are reported in the title of each panel.

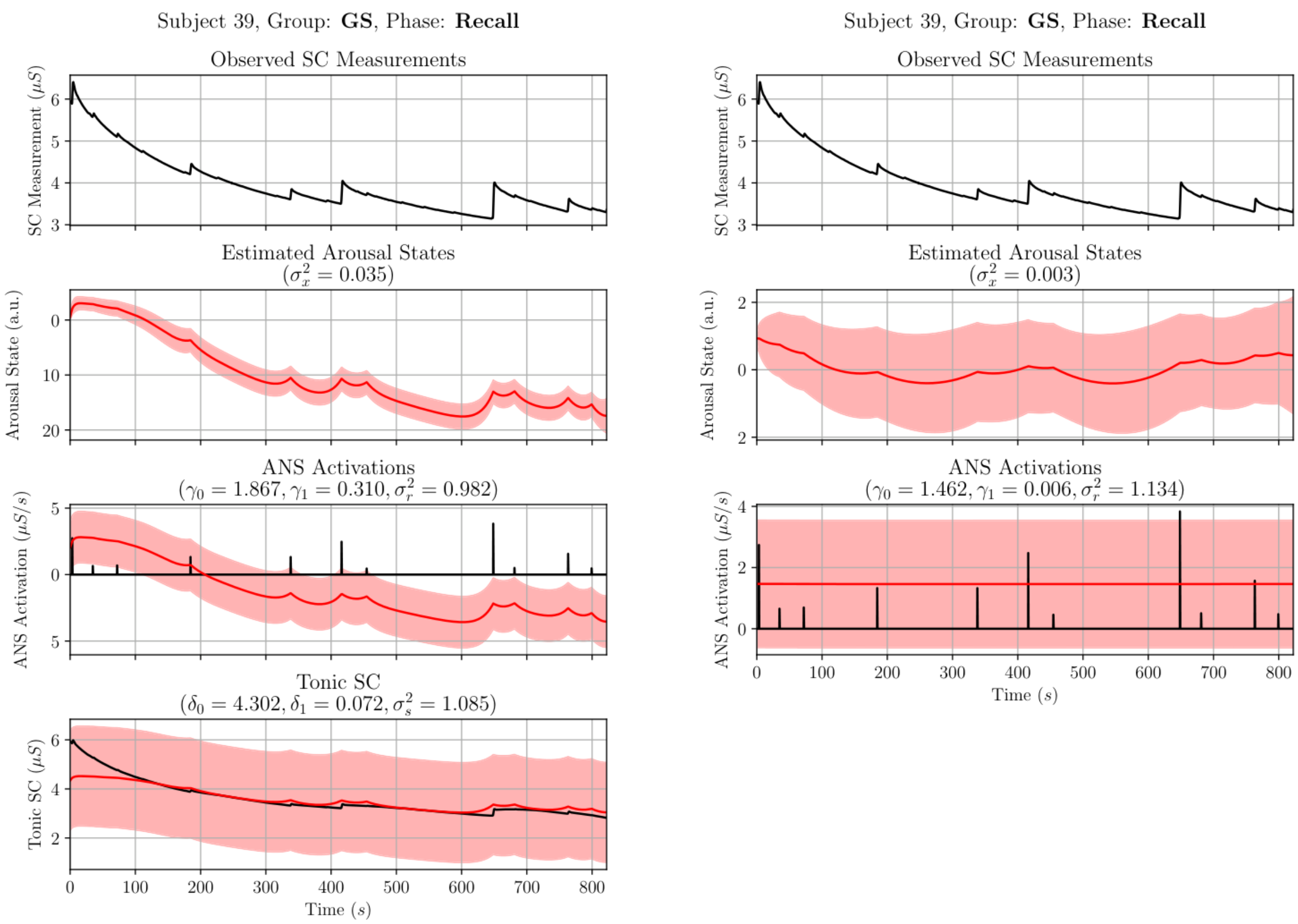


Figure BZ: **Subject 39 estimated arousal states during the recall phase.** Shown for the MPP+Cont model (left) and the MPP model (right). The first panels show the observed SC measurements, $y$ (black). The second panels show the estimated arousal states, $x_{est}$ (red). The third panels show the ANS activations, $r_{obs}$ (black) and the estimated amplitudes, $r_{est}$ (red). The fourth panel shows the tonic component, $s_{obs}$ (black) and the estimated tonic component, $s_{est}$ (red). Estimates are shown with 95% confidence intervals (CI) (red shaded area). Estimated parameters are reported in the title of each panel.

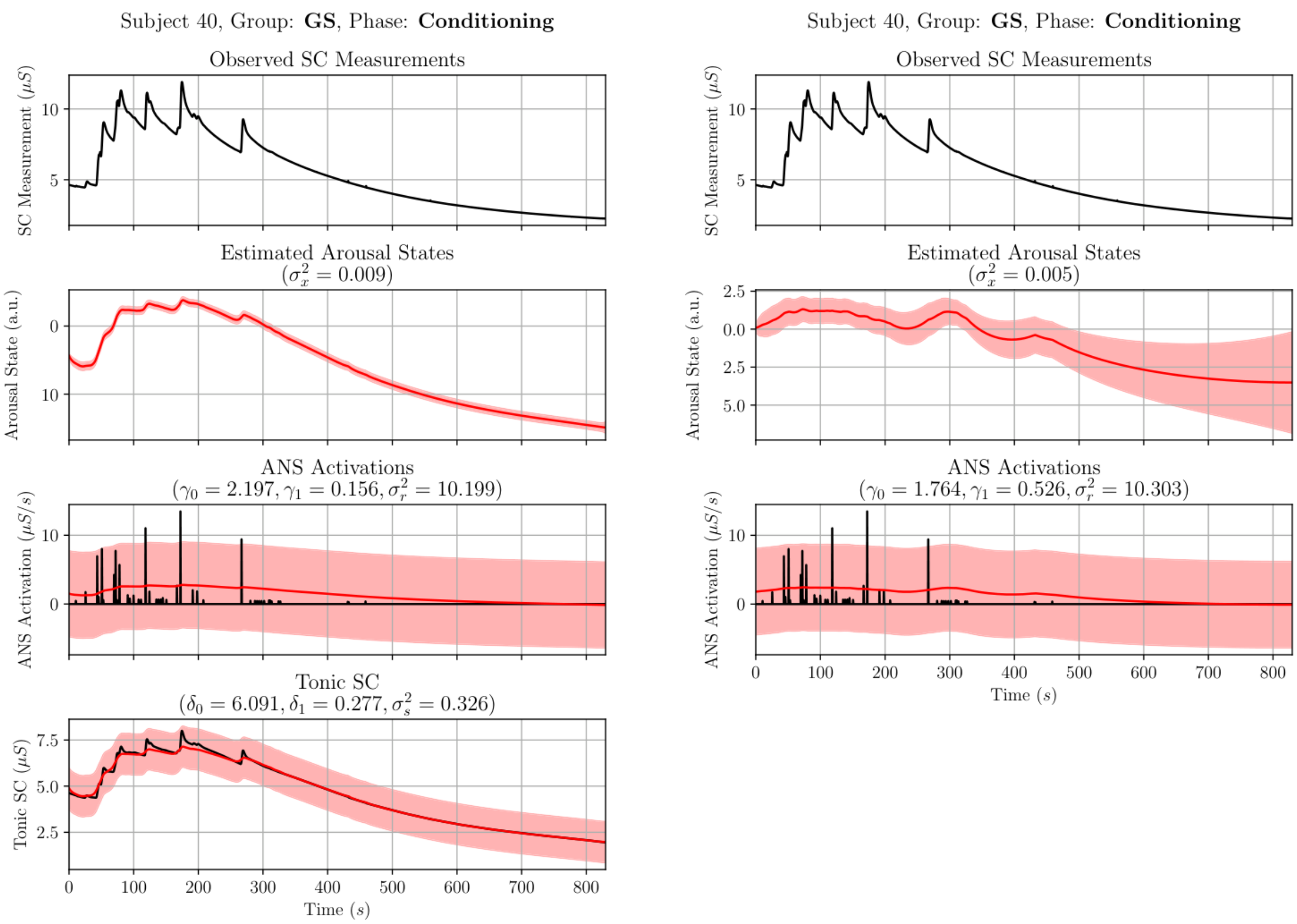


Figure CA: **Subject 40 estimated arousal states during the conditioning phase.** Shown for the MPP+Cont model (left) and the MPP model (right). The first panels show the observed SC measurements, $y$ (black). The second panels show the estimated arousal states, $x_{est}$ (red). The third panels show the ANS activations, $r_{obs}$ (black) and the estimated amplitudes, $r_{est}$ (red). The fourth panel shows the tonic component, $s_{obs}$ (black) and the estimated tonic component, $s_{est}$ (red). Estimates are shown with 95% confidence intervals (CI) (red shaded area). Estimated parameters are reported in the title of each panel.

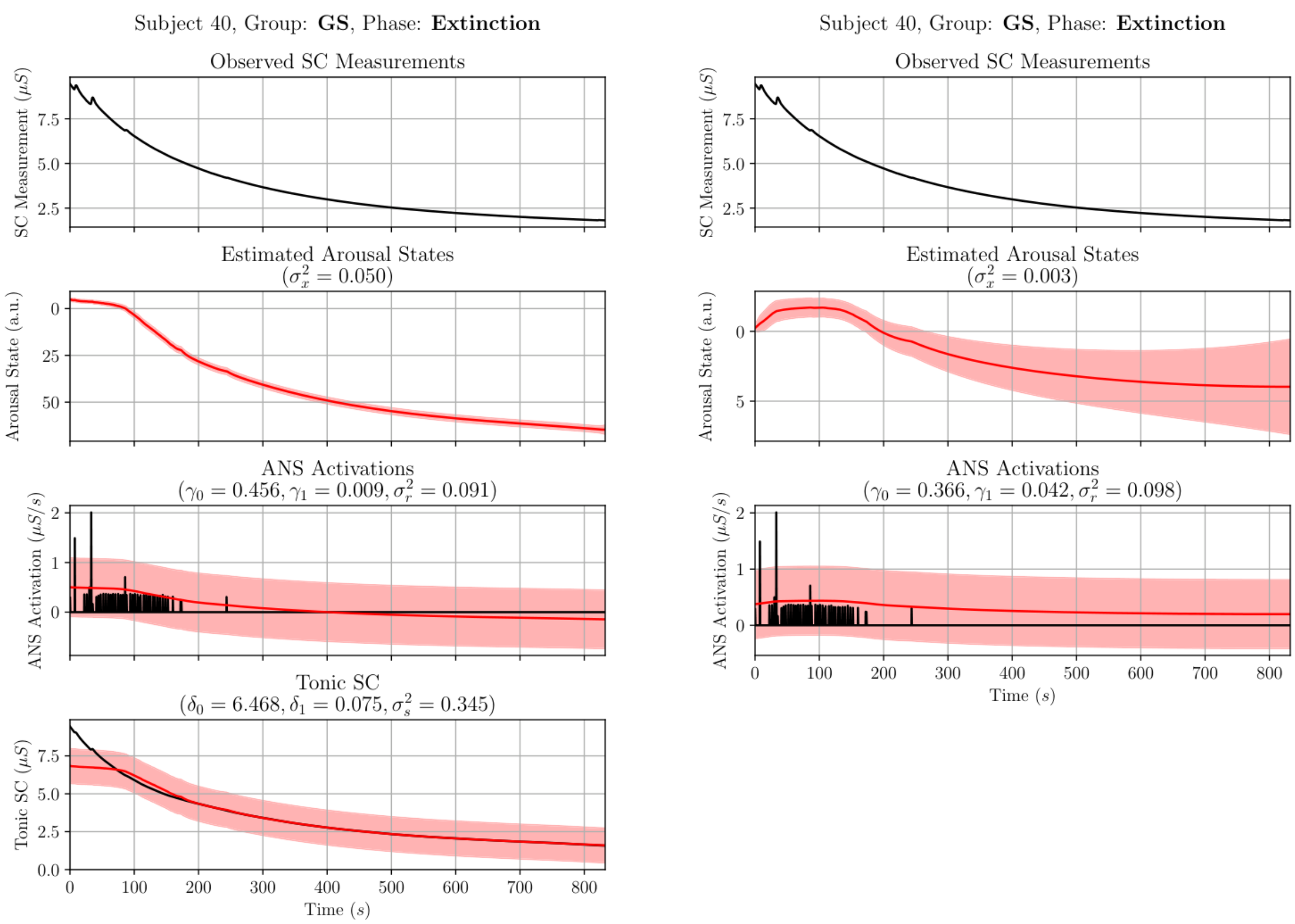


Figure CB: **Subject 40 estimated arousal states during the extinction phase.** Shown for the MPP+Cont model (left) and the MPP model (right). The first panels show the observed SC measurements, $y$ (black). The second panels show the estimated arousal states, $x_{est}$ (red). The third panels show the ANS activations, $r_{obs}$ (black) and the estimated amplitudes, $r_{est}$ (red). The fourth panel shows the tonic component, $s_{obs}$ (black) and the estimated tonic component, $s_{est}$ (red). Estimates are shown with 95% confidence intervals (CI) (red shaded area). Estimated parameters are reported in the title of each panel.

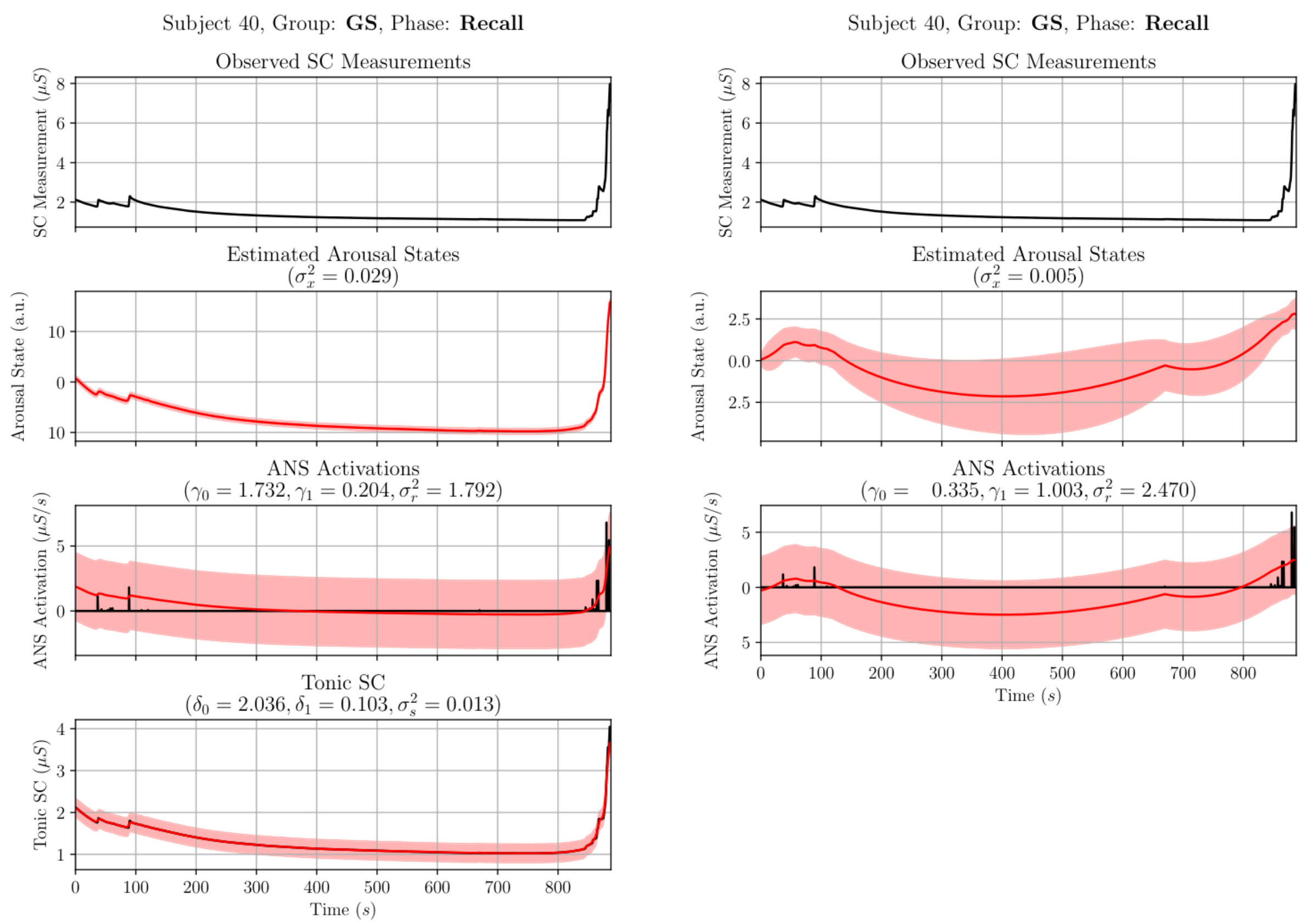


Figure CC: **Subject 40 estimated arousal states during the recall phase.** Shown for the MPP+Cont model (left) and the MPP model (right). The first panels show the observed SC measurements, $y$ (black). The second panels show the estimated arousal states, $x_{est}$ (red). The third panels show the ANS activations, $r_{obs}$ (black) and the estimated amplitudes, $r_{est}$ (red). The fourth panel shows the tonic component, $s_{obs}$ (black) and the estimated tonic component, $s_{est}$ (red). Estimates are shown with 95% confidence intervals (CI) (red shaded area). Estimated parameters are reported in the title of each panel.

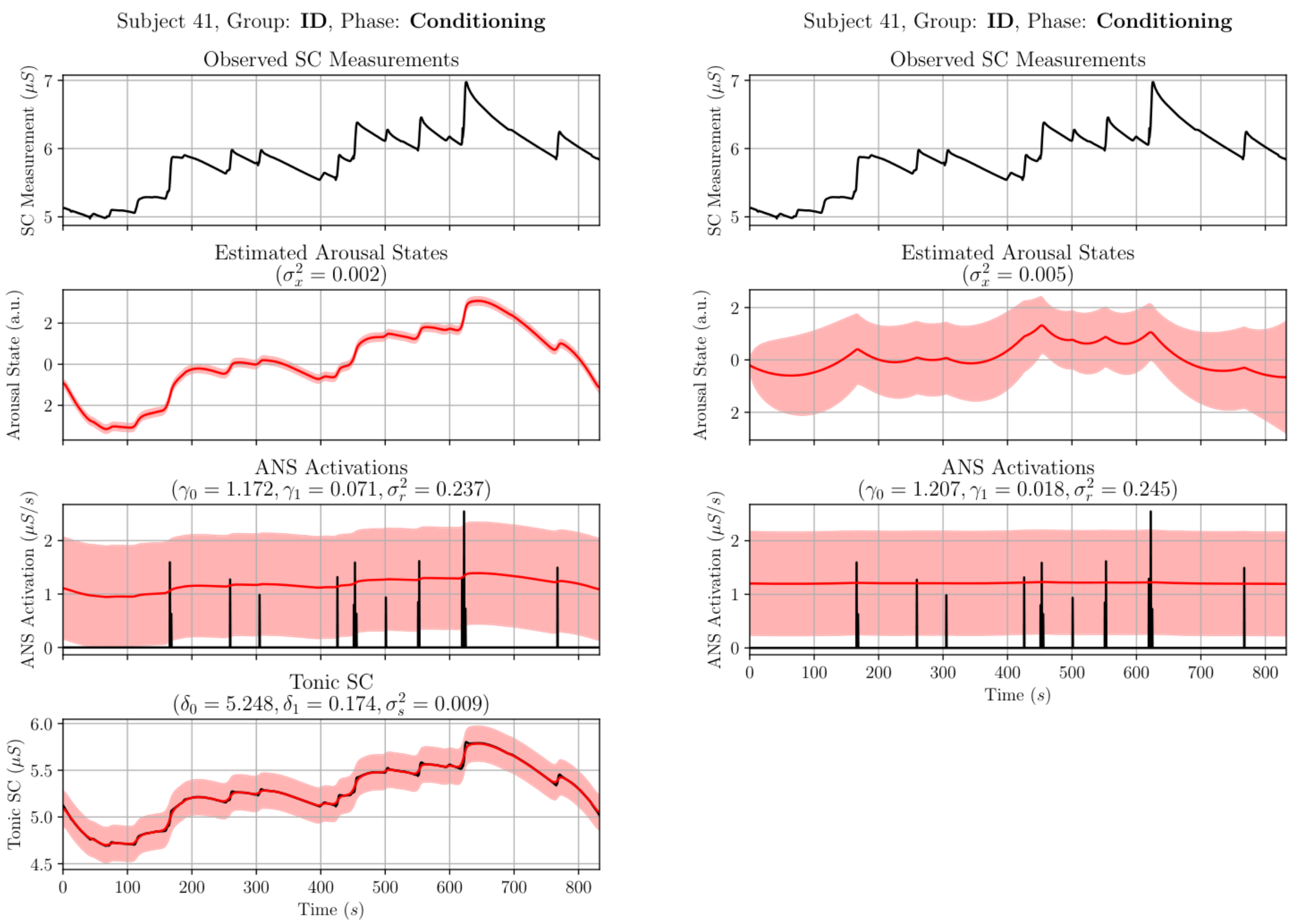


Figure CD: **Subject 41 estimated arousal states during the conditioning phase.** Shown for the MPP+Cont model (left) and the MPP model (right). The first panels show the observed SC measurements, $y$ (black). The second panels show the estimated arousal states, $x_{est}$ (red). The third panels show the ANS activations, $r_{obs}$ (black) and the estimated amplitudes, $r_{est}$ (red). The fourth panel shows the tonic component, $s_{obs}$ (black) and the estimated tonic component, $s_{est}$ (red). Estimates are shown with 95% confidence intervals (CI) (red shaded area). Estimated parameters are reported in the title of each panel.

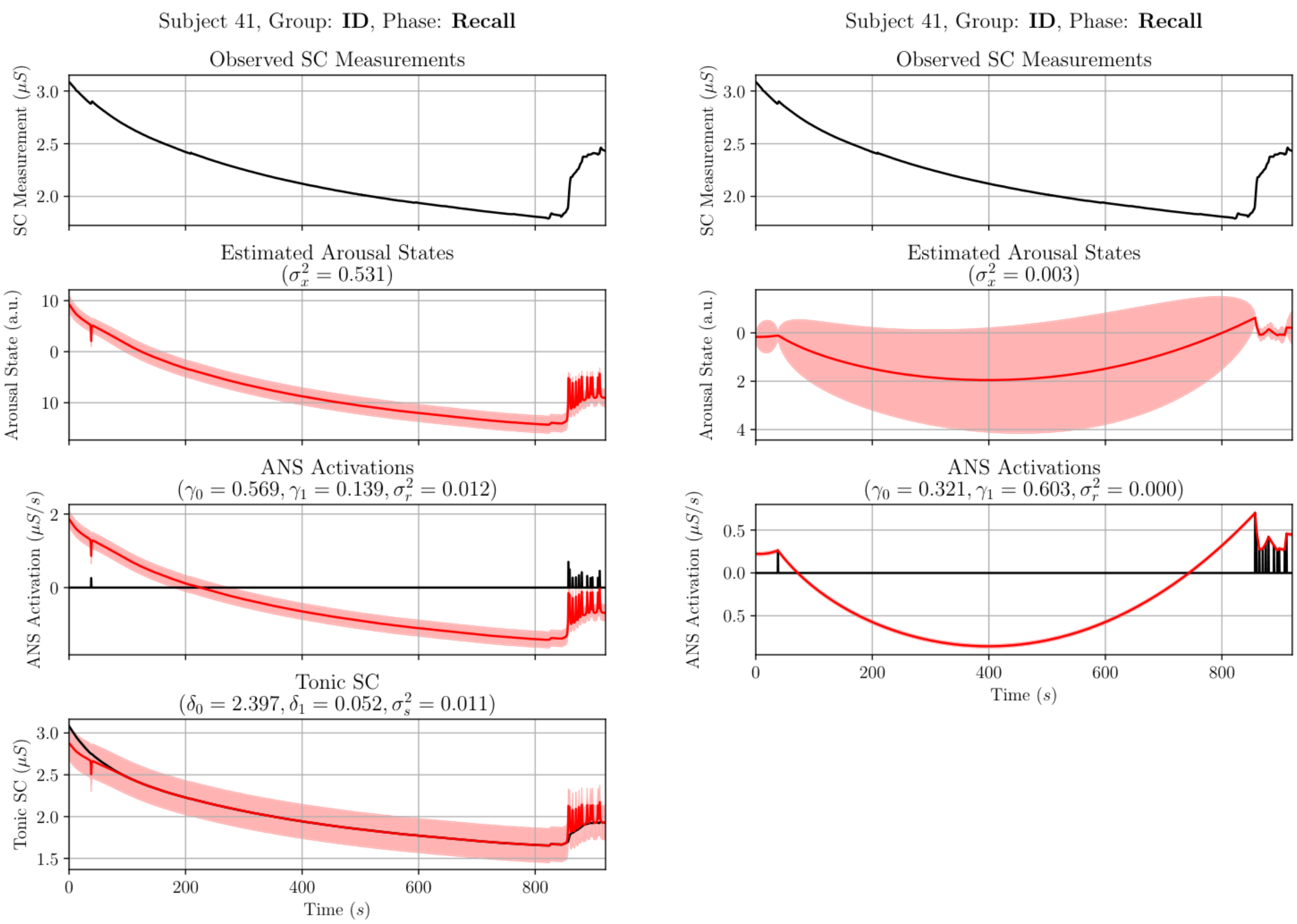


Figure CE: **Subject 41 estimated arousal states during the recall phase.** Shown for the MPP+Cont model (left) and the MPP model (right). The first panels show the observed SC measurements, $y$ (black). The second panels show the estimated arousal states, $x_{est}$ (red). The third panels show the ANS activations, $r_{obs}$ (black) and the estimated amplitudes, $r_{est}$ (red). The fourth panel shows the tonic component, $s_{obs}$ (black) and the estimated tonic component, $s_{est}$ (red). Estimates are shown with 95% confidence intervals (CI) (red shaded area). Estimated parameters are reported in the title of each panel.

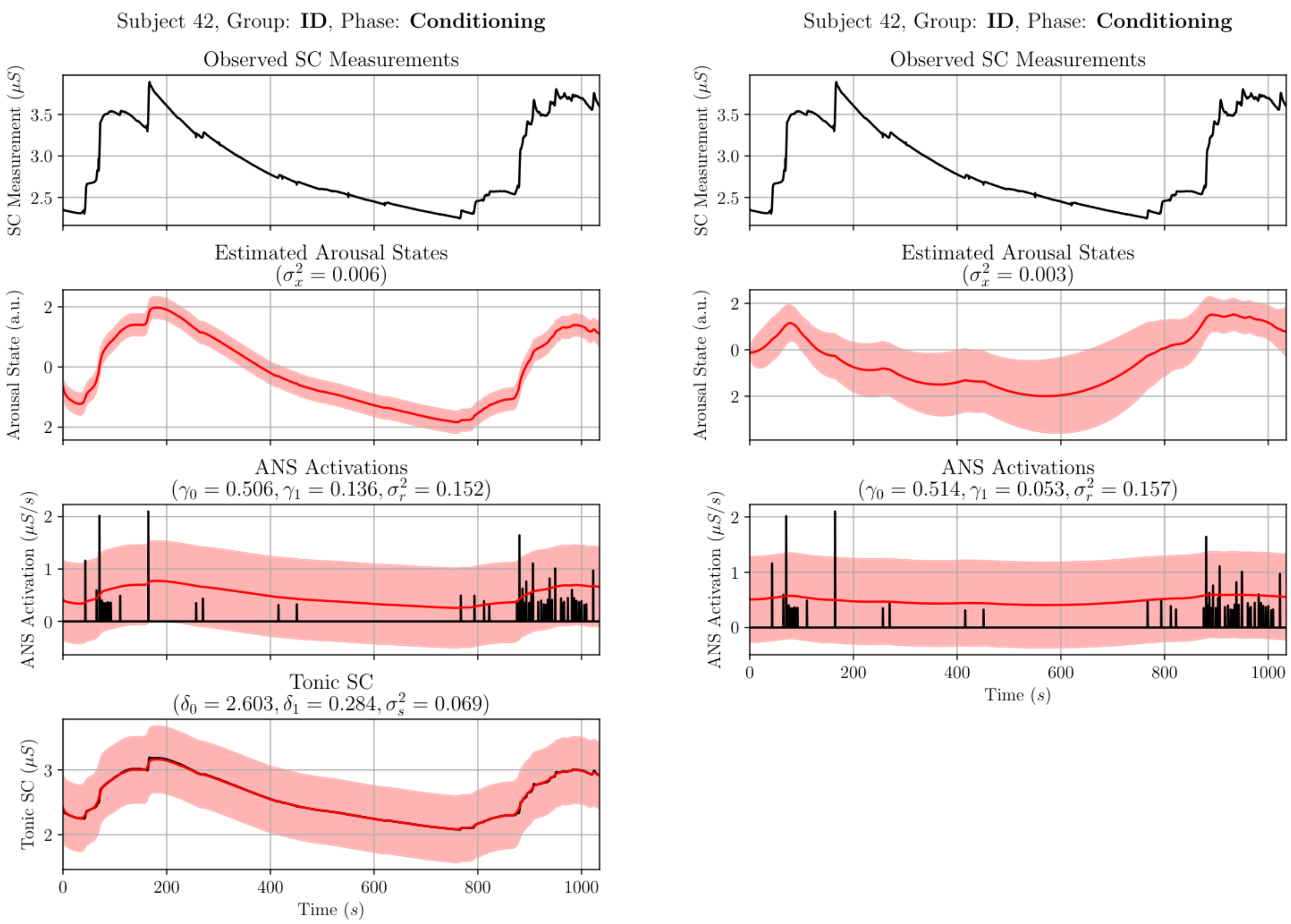


Figure CF: **Subject 42 estimated arousal states during the conditioning phase.** Shown for the MPP+Cont model (left) and the MPP model (right). The first panels show the observed SC measurements, $y$ (black). The second panels show the estimated arousal states, $x_{est}$ (red). The third panels show the ANS activations, $r_{obs}$ (black) and the estimated amplitudes, $r_{est}$ (red). The fourth panel shows the tonic component, $s_{obs}$ (black) and the estimated tonic component, $s_{est}$ (red). Estimates are shown with 95% confidence intervals (CI) (red shaded area). Estimated parameters are reported in the title of each panel.

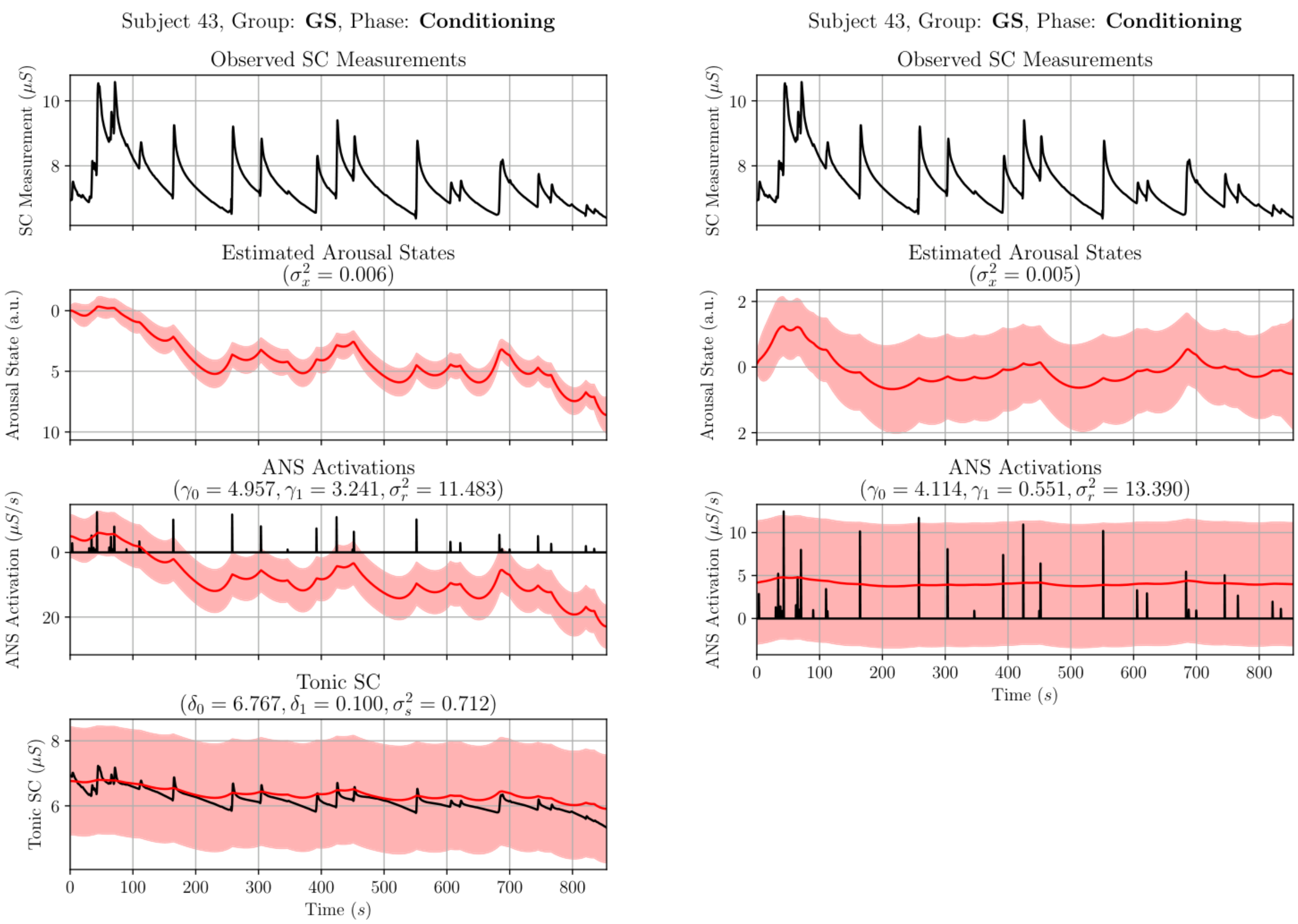


Figure CG: **Subject 43 estimated arousal states during the conditioning phase.** Shown for the MPP+Cont model (left) and the MPP model (right). The first panels show the observed SC measurements, $y$ (black). The second panels show the estimated arousal states, $x_{est}$ (red). The third panels show the ANS activations, $r_{obs}$ (black) and the estimated amplitudes, $r_{est}$ (red). The fourth panel shows the tonic component, $s_{obs}$ (black) and the estimated tonic component, $s_{est}$ (red). Estimates are shown with 95% confidence intervals (CI) (red shaded area). Estimated parameters are reported in the title of each panel.

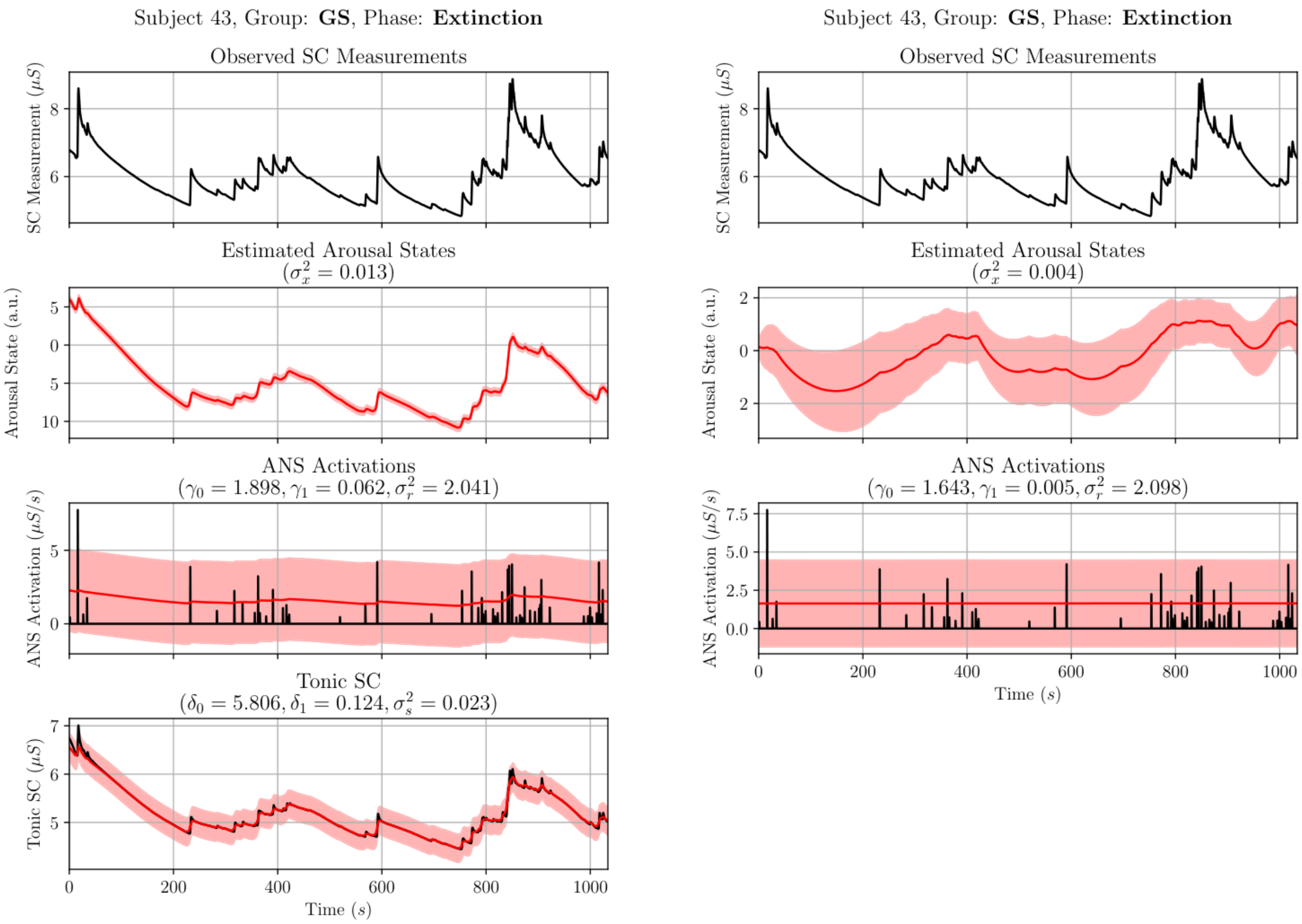


Figure CH: **Subject 43 estimated arousal states during the extinction phase.** Shown for the MPP+Cont model (left) and the MPP model (right). The first panels show the observed SC measurements, $y$ (black). The second panels show the estimated arousal states, $x_{est}$ (red). The third panels show the ANS activations, $r_{obs}$ (black) and the estimated amplitudes, $r_{est}$ (red). The fourth panel shows the tonic component, $s_{obs}$ (black) and the estimated tonic component, $s_{est}$ (red). Estimates are shown with 95% confidence intervals (CI) (red shaded area). Estimated parameters are reported in the title of each panel.

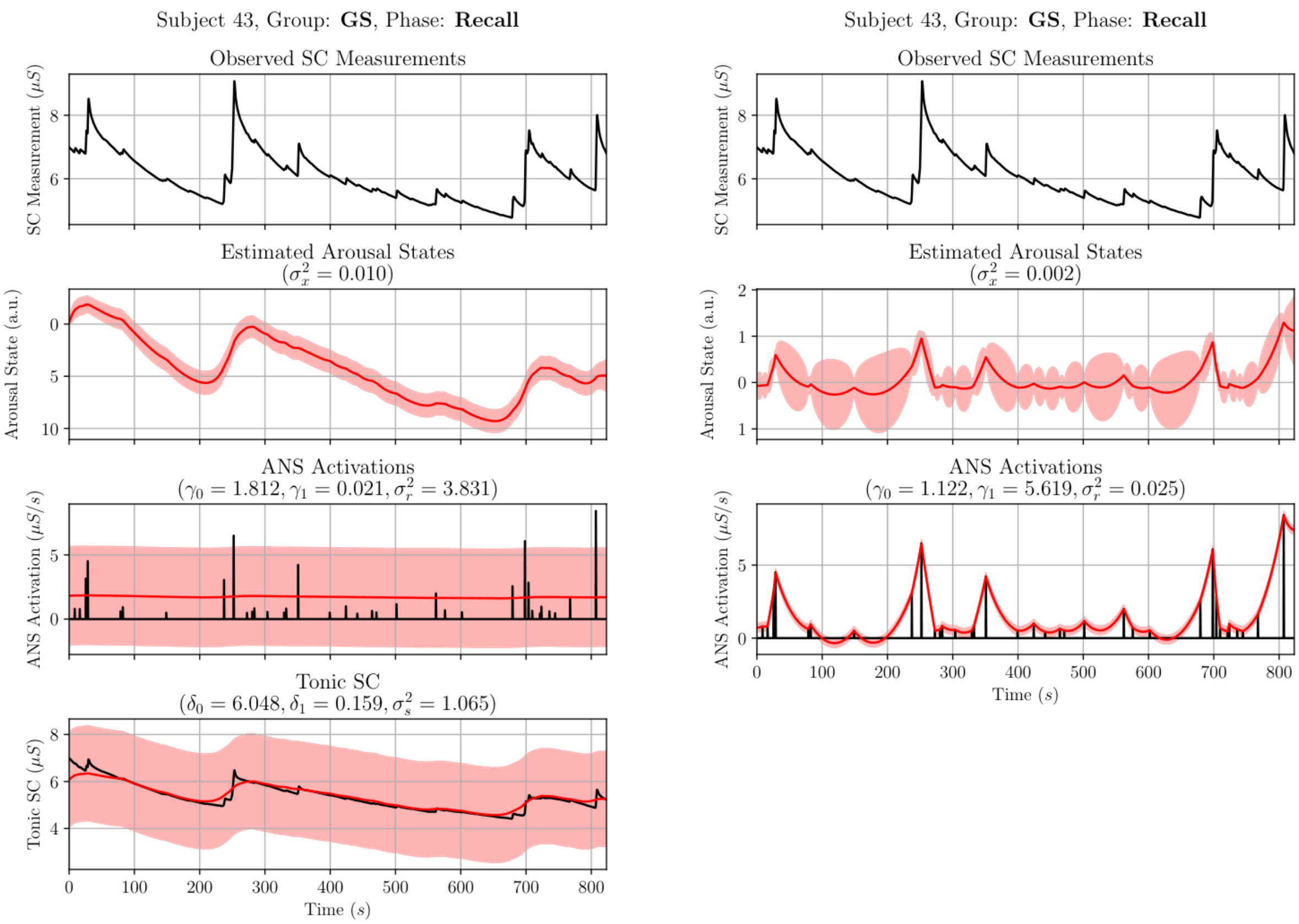


Figure CI: **Subject 43 estimated arousal states during the recall phase.** Shown for the MPP+Cont model (left) and the MPP model (right). The first panels show the observed SC measurements, $y$ (black). The second panels show the estimated arousal states, $x_{est}$ (red). The third panels show the ANS activations, $r_{obs}$ (black) and the estimated amplitudes, $r_{est}$ (red). The fourth panel shows the tonic component, $s_{obs}$ (black) and the estimated tonic component, $s_{est}$ (red). Estimates are shown with 95% confidence intervals (CI) (red shaded area). Estimated parameters are reported in the title of each panel.

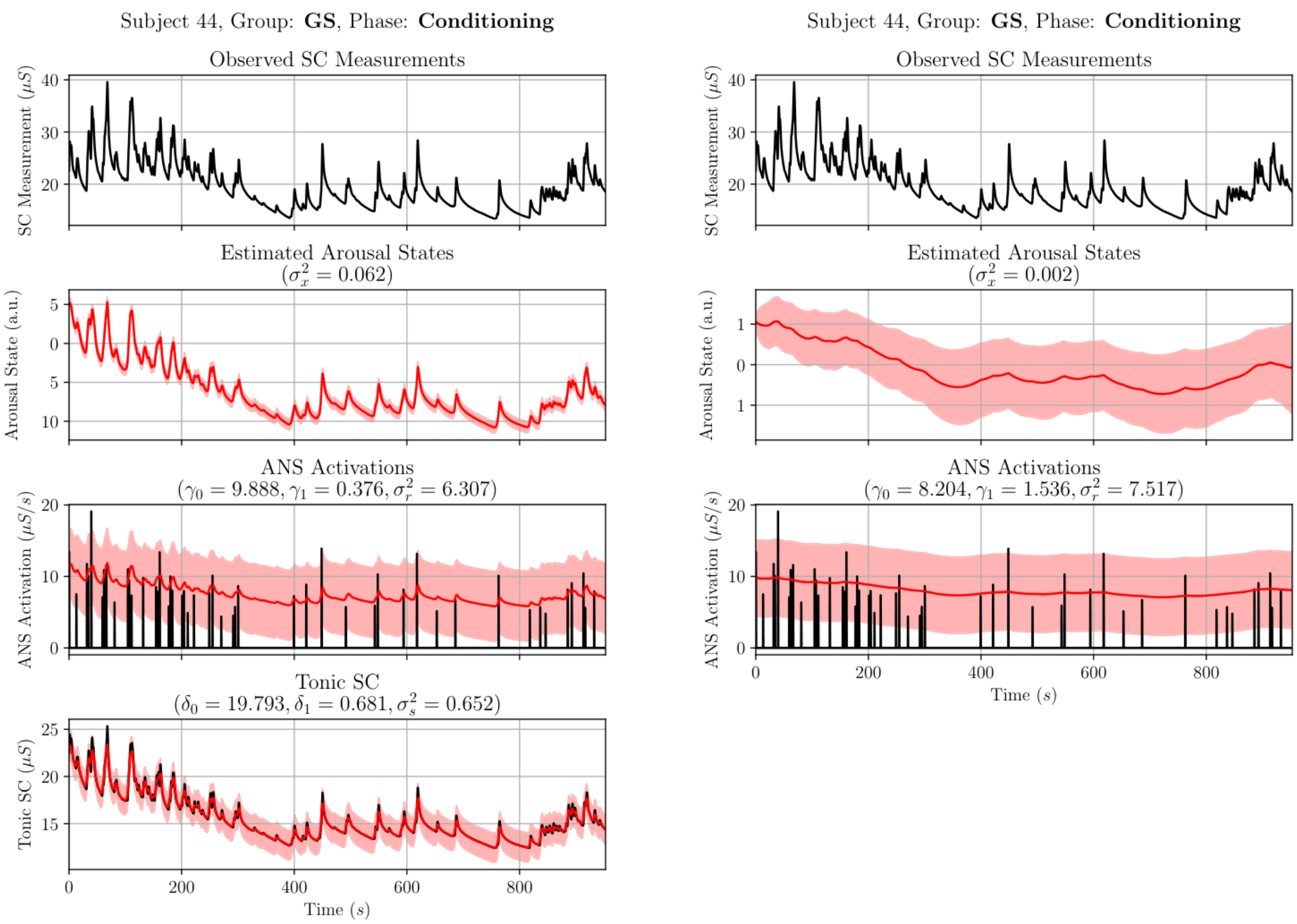


Figure CJ: **Subject 44 estimated arousal states during the conditioning phase.** Shown for the MPP+Cont model (left) and the MPP model (right). The first panels show the observed SC measurements, $y$ (black). The second panels show the estimated arousal states, $x_{est}$ (red). The third panels show the ANS activations, $r_{obs}$ (black) and the estimated amplitudes, $r_{est}$ (red). The fourth panel shows the tonic component, $s_{obs}$ (black) and the estimated tonic component, $s_{est}$ (red). Estimates are shown with 95% confidence intervals (CI) (red shaded area). Estimated parameters are reported in the title of each panel.

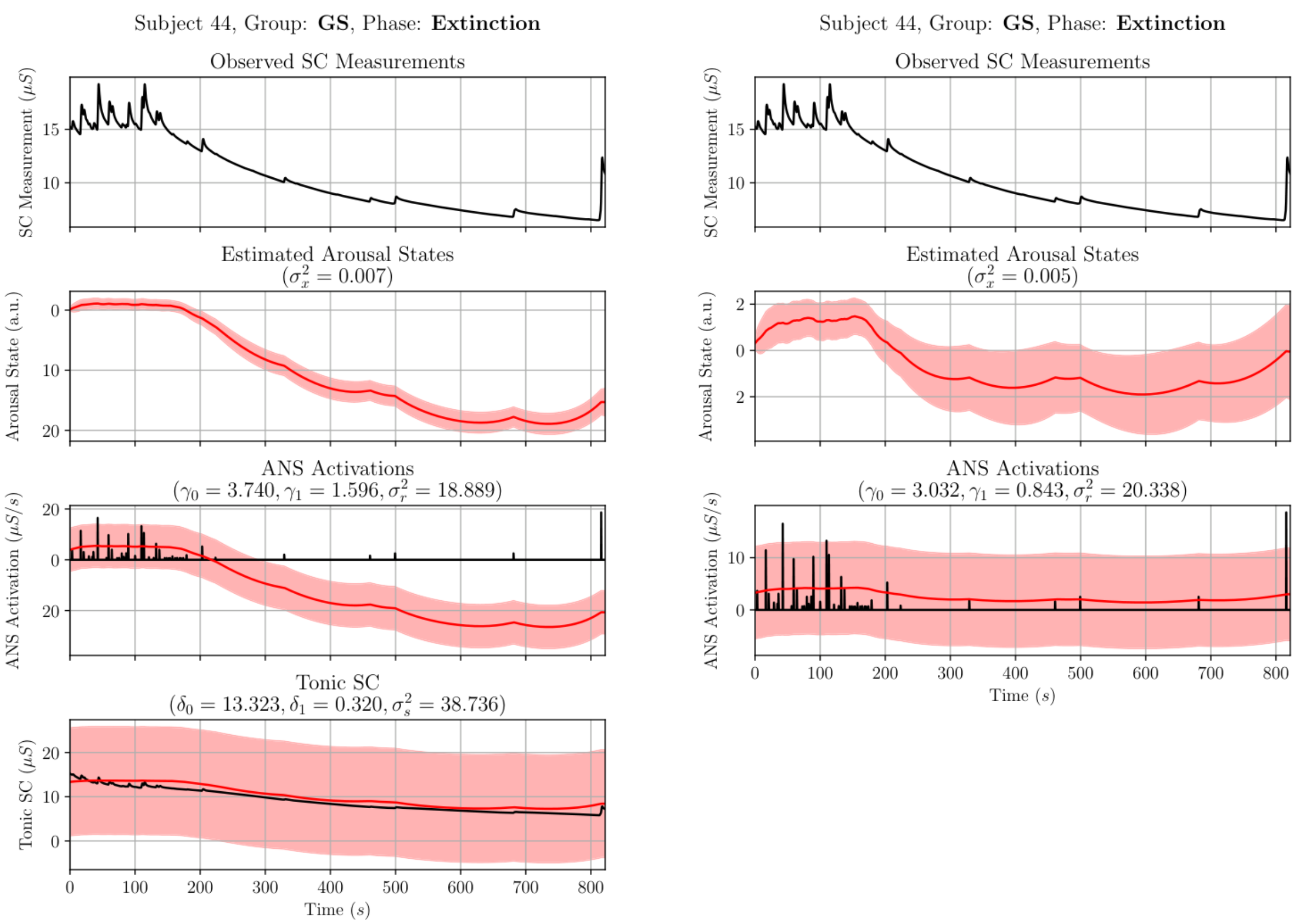


Figure CK: **Subject 44 estimated arousal states during the extinction phase.** Shown for the MPP+Cont model (left) and the MPP model (right). The first panels show the observed SC measurements, $y$ (black). The second panels show the estimated arousal states, $x_{est}$ (red). The third panels show the ANS activations, $r_{obs}$ (black) and the estimated amplitudes, $r_{est}$ (red). The fourth panel shows the tonic component, $s_{obs}$ (black) and the estimated tonic component, $s_{est}$ (red). Estimates are shown with 95% confidence intervals (CI) (red shaded area). Estimated parameters are reported in the title of each panel.

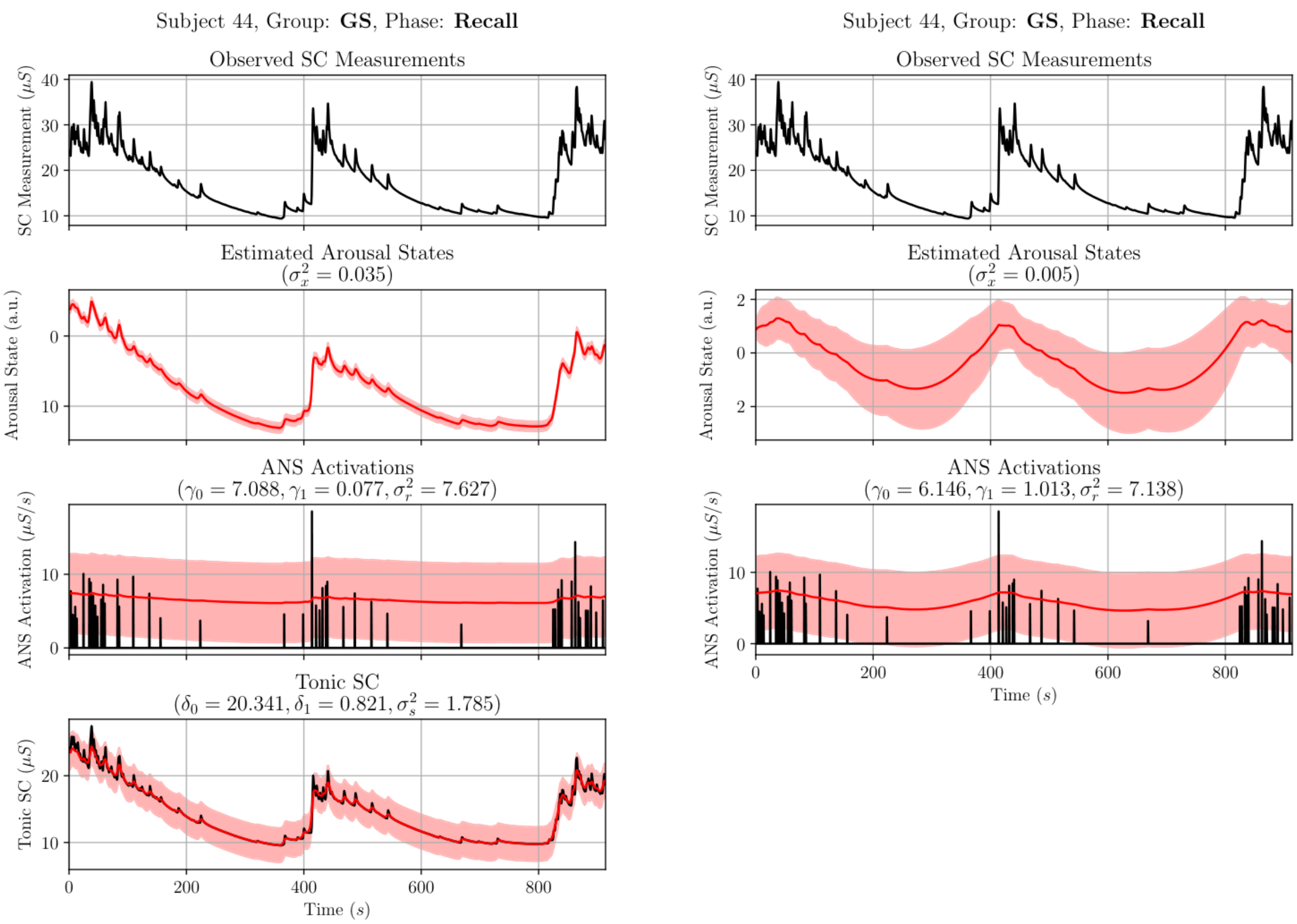


Figure CL: **Subject 44 estimated arousal states during the recall phase.** Shown for the MPP+Cont model (left) and the MPP model (right). The first panels show the observed SC measurements, $y$ (black). The second panels show the estimated arousal states, $x_{est}$ (red). The third panels show the ANS activations, $r_{obs}$ (black) and the estimated amplitudes, $r_{est}$ (red). The fourth panel shows the tonic component, $s_{obs}$ (black) and the estimated tonic component, $s_{est}$ (red). Estimates are shown with 95% confidence intervals (CI) (red shaded area). Estimated parameters are reported in the title of each panel.
